# Dispersive Fluorine/Bromine Interactions as Key Selectivity Determinant: Asymmetric Cyclopropanations with 3,3,3-Trifluoro-2-diazopropionate Catalyzed by a Heterochiral-at-The-Metal Centers Dirhodium Paddlewheel Complex

**DOI:** 10.1021/jacs.5c17842

**Published:** 2025-12-18

**Authors:** Matthias Peeters, Lucas Marchal, Sofia Lerda, Giovanni Bistoni, Alois Fürstner

**Affiliations:** † 28314 Max-Planck-Institut für Kohlenforschung, 45470 Mülheim/Ruhr, Germany; ‡ Department of Chemistry, Biology, and Biotechnology, 9309 University of Perugia, I-06123 Perugia, Italy

## Abstract

Asymmetric cyclopropanation reactions of 3,3,3-trifluoro-2-diazopropionate have so far been largely beyond the scope of dirhodium catalysts. This significant gap in coverage is now closed with the aid of a new class of paddlewheel complex with “backbone chirality”, in which an array of only three different achiral ligands (acetamidate, trifluoroacetate, calix[4]­arene dicarboxylate) renders the two rhodium centers inequivalent and chiral-at-the-metal each. The –NH group of the acetamidate engages the ester carbonyl of the metal carbene intermediate into favorable interligand hydrogen bonding, which in turn controls the trajectory of the incoming olefinic reaction partner. In addition, a dispersive interaction between the −CF_3_ group flanking the carbene center and a strategically placed bromine substituent on the calixarene dicarboxylate locks the conformation of the reactive intermediate in place within the chiral binding site, which translates into high diastereoselectivity and optical purity alike. The same type of interhalogen London dispersion interaction can also benefit [2 + 1] cycloadditions of other fluorinated carbene donors.

## Graphical abstract

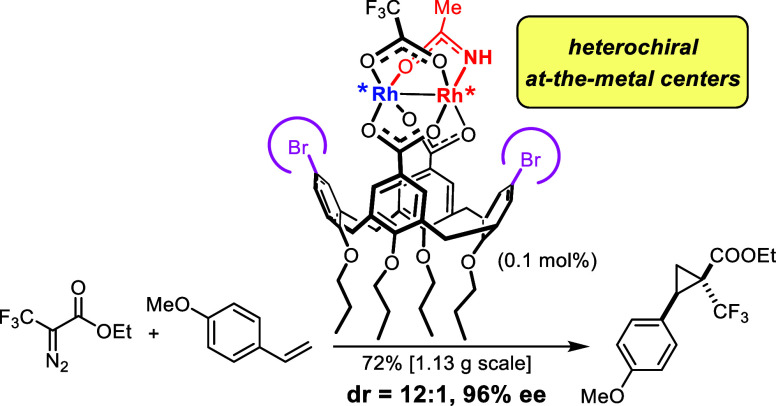

## Introduction

Our group has recently disclosed complex *P* **-1** and its enantiomer *M* **-1** as the prototype of an entirely new class of dirhodium paddlewheel complexes for asymmetric catalysis (Scheme 1A).^1^ Unlike virtually all other dirhodium catalysts known in the literature, which consist of a dimetallic lantern core framed by four (identical) chiral ligands,^2–6^ our new design capitalizes on complexes of type **A** comprised of two inequivalent rhodium centers, each of which is chiral-at-the-metal itself.^1,7^ Conceptually speaking, three different achiral μ-bridging equatorial ligands suffice to craft such heterochiral compounds. In practice, however, it is essential that one of them is a primary carboxamidate, as the –NH group exerts a number of critically important functions. First, it helps to distinguish the two inequivalent Rh centers and, in doing so, determines the site at which the actual catalytic transformation will take place.^8,9^ Upon reaction with a diazoester, a “super-electrophilic” rhodium carbene **B** is formed at the Rh­[O_3_N] face that is locked in place within the chiral environment by an interligand hydrogen bond between this protic site and the ester carbonyl;^8,10^ such a restricted conformation is arguably beneficial for asymmetric induction. Equally relevant is the fact that this noncovalent structural motif plays a key role in controlling the trajectories by which an olefin can approach the reactive carbene center;^10^ computational data and spectroscopic evidence suggest that any attack that disturbs the stabilizing hydrogen bonding array is highly disfavored, even if the corresponding quadrant is sterically wide open.

**Scheme 1:**
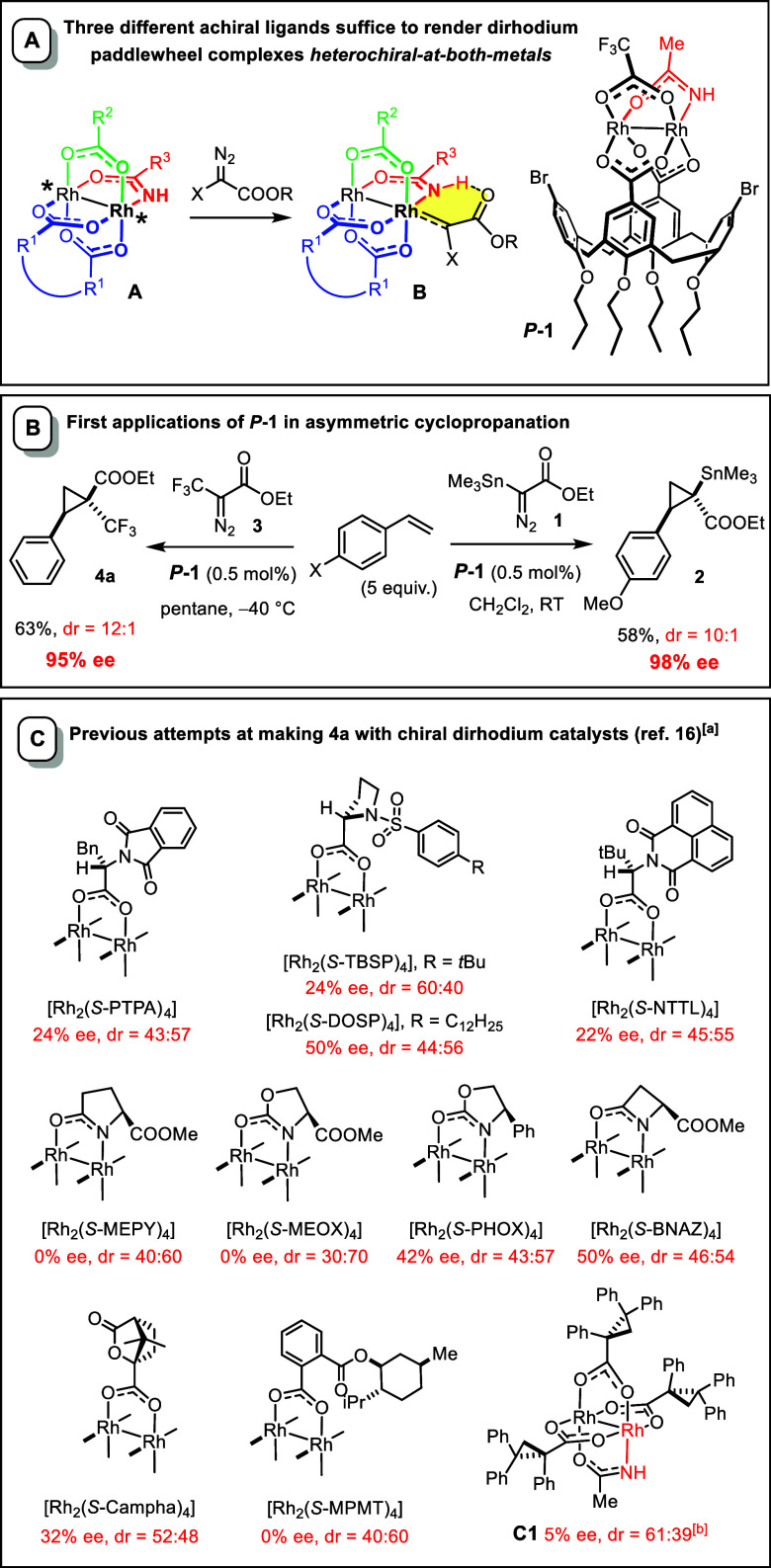
Archetype of a New Class of Chiral-at-Metal Dirhodium Catalysts; The Challenge of Engaging Ethyl 3,3,3-Trifluoro-2-diazopropionate (**3**) in Catalytic Asymmetric Cyclopropanation

a Only the ee of *trans*-**4a** is shown; the ee’s of the *cis*-isomer was even lower in all cases (0–18% ee); the reactions were performed with 5 mol % of the respective catalyst in CH_2_Cl_2_ at room temperature; note: only one of the four identical ligands of the catalysts shown in panel C is drawn for the sake of clarity, except of **C1**, which is a heteroleptic complex that is drawn in full.
b Unpublished results from this laboratory; *p*-methoxystyrene was used as the substrate.

The design of our archetypical complex *P* **-1** mirrors two additional facets that are also relevant. If only *three* chemically different achiral ligands are chosen to fill the *four* μ_2_-bridging equatorial sites about the Rh–Rh axis, the two identical ligands must be placed *cis* to each other to obtain a complex **A** that is chiral-at-both-metals. From the synthetic viewpoint, this precondition is best met by tethering them together, e.g. in form of a dicarboxylate.^1^ Of the many conceivable candidates, a well accessible calix[4]­arene dicarboxylic acid^11^ was chosen because its cone-shape was expected to favor formation of the targeted *cis*-chelate complex over competing dimerization/oligomerization; at the same time, the bromo-substituents on the two other alternating phenyl rings reside in proximity of the axial coordination sites of *P* **-1** where the actual reaction will take place and are hence able to steer the catalytic transformation without preventing it from happening.^12,13^

This unorthodox design paid valuably dividends in that complex *P* **-1** was found to catalyze reactions of the α-stannylated α-diazoester **1** with an assortment of alkenes very well, furnishing valuable stannylated cyclopropanes such as **2** with outstanding optical purity, which allow for diverse downstream functionalization (Scheme 1B).^1^ Particularly rewarding is the fact that the *cis*-isomer was formed selectively; this outcome complements the highly *trans*-selective course of the reaction when catalyzed by heteroleptic complexes such as **C1** previously developed in our laboratory.^10,14,15^ A detailed computational analysis confirmed that interligand hydrogen bonding between the protic −NH group of the equatorial acetamidate ligand and the ester carbonyl of the stannylated rhodium carbene of type **B** (X = SnMe_3_) derived from *P* **-1** and **1** controls the stereochemical outcome;^1^ in any case, the *cis*-configured cyclopropane (*R,R*)-**2** was computed to be formed as the—by far—major product isomer, in perfect agreement with the experimental finding.

Complex *P* **-1** was also shown to catalyze the reaction of ethyl 3,3,3-trifluoro-2-diazopropionate (**3**) with styrene to give the trifluoromethylated cyclopropane **4a** with a similarly high level of asymmetric induction (dr = 12:1, 95% ee) (Scheme 1B).^1^ This result is all the more remarkable given that an earlier study was unable to identify any rhodium catalyst capable of supplying **4a** with a meaningful ee, even though no less than 10 different chiral paddlewheel complexes had been screened (Scheme 1C).^16^ In several cases, the product was racemic, and even the best catalysts gave very modest results ([Rh_2_((*S*)-DOSP)_4_]: 18% ee (*cis*), 50% ee (*trans*); [Rh_2_((*S*)-BNAZ)_4_]: 4% ee (*cis*), 50% ee (*trans*)); moreover, all of them reacted diastereo-unselectively (dr ≈ 1:1). The heteroleptic complex **C1**,^10^ which is a highly effective catalyst for reactions with stannylated diazoesters, proved equally disappointing. Despite the massive advances in asymmetric cyclopropanation chemistry,^17–23^ catalytic asymmetric reactions of 3,3,3-trifluoro-2-diazopropionate are apparently a largely unsolved problem.^24,25^ Only recently has a single report been published, describing three examples of highly asymmetric cyclopropanations of **3** with a particular type of allyl phenyl sulfone bearing an electron withdrawing group (ketone, ester) on the internal position of the olefin; styrene derivatives and/or other terminal olefins have not been revisited in this study.^26^ For these types of substrates commonly used in asymmetric cyclopropanation chemistry, the results summarized in Scheme 1C still represent the state of the art.^16^

When seen against this backdrop and in consideration of the ever-growing importance of trifluoromethylated building blocks in general,^27–32^ we felt encouraged to pursue this lead finding and investigate asymmetric cyclopropanations with 3,3,3-trifluoro-2-diazopropionate **3** catalyzed by *P* **-1** in more detail. In this context, however, a striking and even discomforting aspect required particular attention: note that *P* **-1** furnished the *trans*-isomer of the trifluoromethylated cyclopropane **4a** but the *cis*-isomer of its trimethylstannylated analogue **2** with high diastereoselectivity each.^1^ As the stereochemical assignment of these compounds is unambiguous and the formal CIP-priorities of a −CF_3_ and −SnMe_3_ group over −COOR are identical, this opposing outcome was not only unexpected but is obviously difficult to explain. The question as to why the reactions leading to **2** and **4** take the opposite stereochemical course although they are effected by the same catalyst is arguably of *fundamental* importance for the understanding of how the heterochiral-at-the-metal centers complex *P* **-1** operates.

## Results and Discussion

### Computational Study

This key issue was addressed by detailed DFT calculations. For a system of this size, a reliable computational protocol must combine thorough exploration of the conformational space with a careful treatment of all relevant electronic effects. To this end, an approach was chosen that had previously allowed us to reproduce or even predict key experimental findings in related catalytic systems.^8,10^ First, the structure of the carbene intermediate **B**_**CF**_3__ formed on reaction of **3** with *P* **-1** at the [O_3_N]-face of the catalyst was computed. To this end, a conformational sampling was performed without any constraint using the CREST program^33^ and the GFN2-xTB semiempirical method.^34,35^ An in-house code was then used to compare RMSDs across the conformational space; the selected conformers were refined using the B3LYP-D3­(BJ) functional and the def2-SVP basis set^36^ with the RIJCOSX approximation.^37–39^ Implicit solvation effects at the CPCM level for pentane were included to reproduce the experimental conditions as closely as possible.^40,41^ Free energies were obtained by adding thermal corrections computed at the B3LYP-D3BJ/def2-SVP + RIJCOSX + CPCM­(pentane) level of theory to electronic energies calculated at B3LYP-D3BJ/def2-TZVP+RIJCOSX + CPCM­(pentane).^42^

As expected, **B**_**CF**_3__ shows the bespoken interligand hydrogen bonding. Importantly, however, the lowest energy conformer apparently benefits from an additional stabilizing interaction between the −CF_3_ group and the −Br substituents on the calixarene; as a result, the −CF_3_ substituent is oriented toward the calixarene (Figure 1). The synergistic influence of both stabilizing interactions is evident not least in the fact that the second and third most stable conformers, in which either the hydrogen bond or the presumed fluorine/bromine interaction is missing, are 1.1 and 1.8 kcal·mol^–1^ higher in energy.

**Figure 1:**
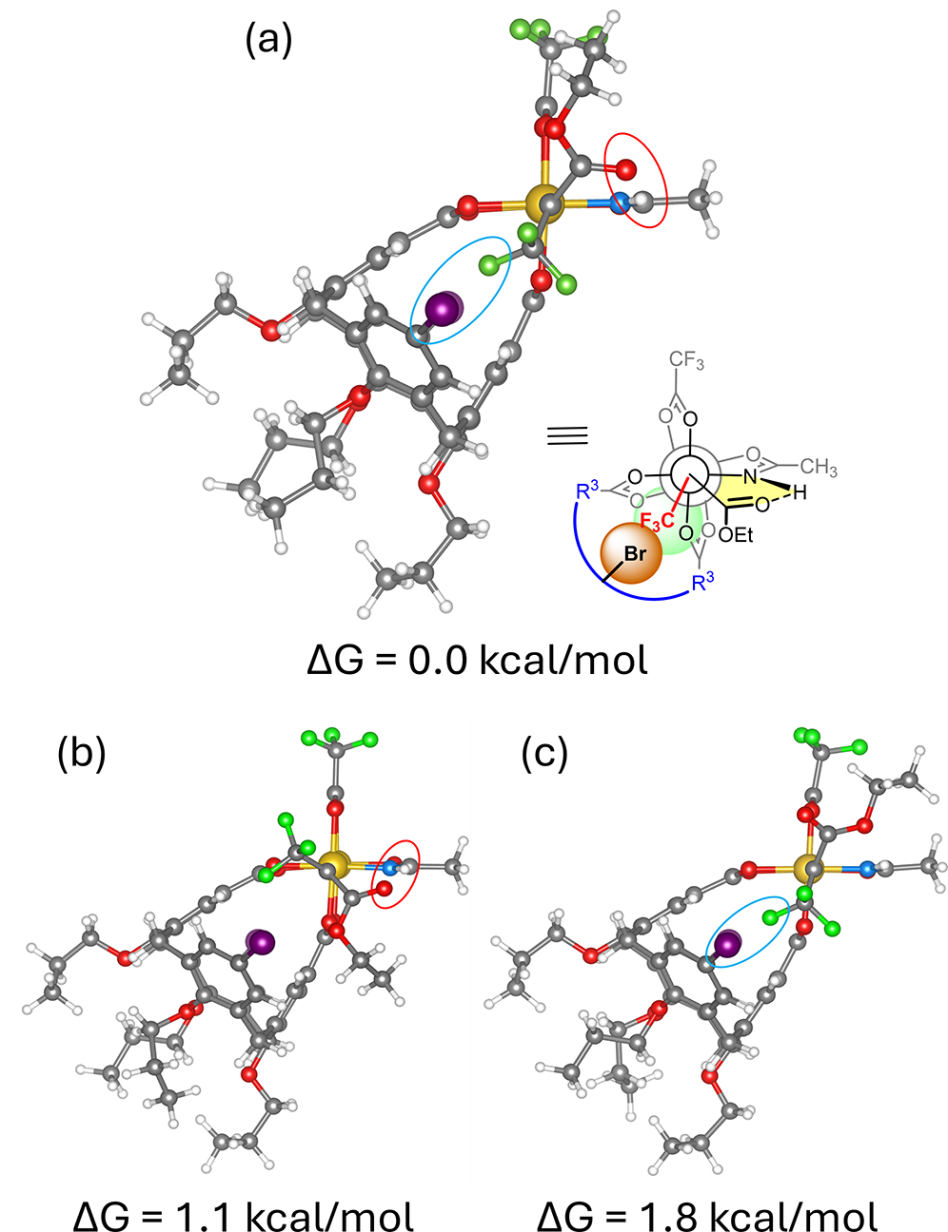
The three most stable conformers of the trifluoromethyl substituted carbene **B**_**CF**_3__ derived from *P* **-1** and diazoester **3**.

The actual nature of this fluorine/bromine interaction was investigated with a model system comprised of phenyl bromide and trifluoroethane held in a spatial arrangement mimicking the array in the carbene intermediate **B**_**CF**_3__ (Figure 2). Single-point electronic energy calculations showed that the heterodimer was 0.81 kcal·mol^–1^ lower in energy than the sum of the individual components, thus indicating a stabilizing contact. Atomic Decomposition of London Dispersion energy (ADLD)^43^ computations on the dimer model confirmed that the main dispersive contributions result from the contact between the bromine atom in the phenyl bromide and the fluorine atoms of trifluoroethane (for details, see the Supporting Information). Note that the heterodimeric array was found to be 1.53 kcal·mol^–1^ higher in energy than its two constituents taken apart when the same calculations were performed without accounting for dispersive forces. Moreover, the computed electrostatic potential map of the dimeric model showed that the electron-rich regions of the fluorine atoms are not oriented toward the σ-hole of the bromine substituent (Figure 2).^44^ Therefore, it can be safely concluded that the observed attractive force in **B**_**CF**_3__ originates from dispersive rather than electrostatic interactions.^45,46^

**Figure 2:**
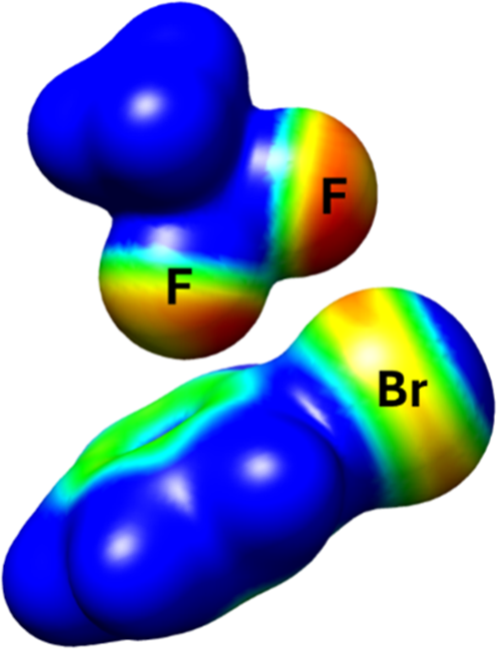
Map of the electrostatic potential of the dimer model consisting of C_6_H_5_Br and CH_3_CF_3_, displayed at an isosurface value of 0.008. The color scale ranges from −0.02 (red, electron-rich regions) to +0.02 (blue, electron-deficient regions).

Such an additional stabilizing effect is missing in the stannylated analogue **B**_**SnMe**_3__. While the peripheral hydrogen bonding between the ester carbonyl and the ligand’s –NH group is equally prominent, the carbene itself can populate a larger conformational space as it is not locked in place by a dispersive interaction. In the most stable conformer, the –SnMe_3_ group actually points away from (rather than toward) the calixarene.^47^ Reactant complex (RC) optimizations showed that the different orientation of the substituents in **B**_**CF**_3__ and **B**_**SnMe**_3__ is the very reason why the subsequent cyclopropanations ultimately take the opposite stereochemical course (Scheme 2). As mentioned above, a front side attack is rendered unfavorable in both cases as it would disturb or even disrupt the stabilizing interligand hydrogen bond between the −NH group of the equatorial acetamidate ligand and the carbene ester carbonyl; the lower quadrant in the back is shielded by the bulky calixarene scaffold. When styrene approaches **B**_**SnMe**_3__ via the top-left backside quadrant, the “upward” orientation of the −SnMe_3_ group away from the calixarene is retained in **RC**_**SnMe**_3__; this geometry eventually translates into the *cis*-configured stannylated cyclopropane **2**.^1^ In case of **B**_**CF**_3__, however, the attractive force resulting from the dispersive fluorine/bromine contact prevents an analogous conformation from being reached; rather, the −CF_3_ substituent is faithfully kept in close vicinity of the calixarene. This arrangement (**RC**_**CF**_3__) leads to the *trans*-configured trifluoro­methylated cyclopropane **4a** as the major product, in line with the experimental results. The catalytic cyclopropanations effected by *P* **-1** are hence a striking illustration for how dispersive interligand forces can even “invert” the stereochemical outcome of a catalytic asymmetric transformation, although the pathway itself is highly conserved in mechanistic terms.

**Scheme 2:**
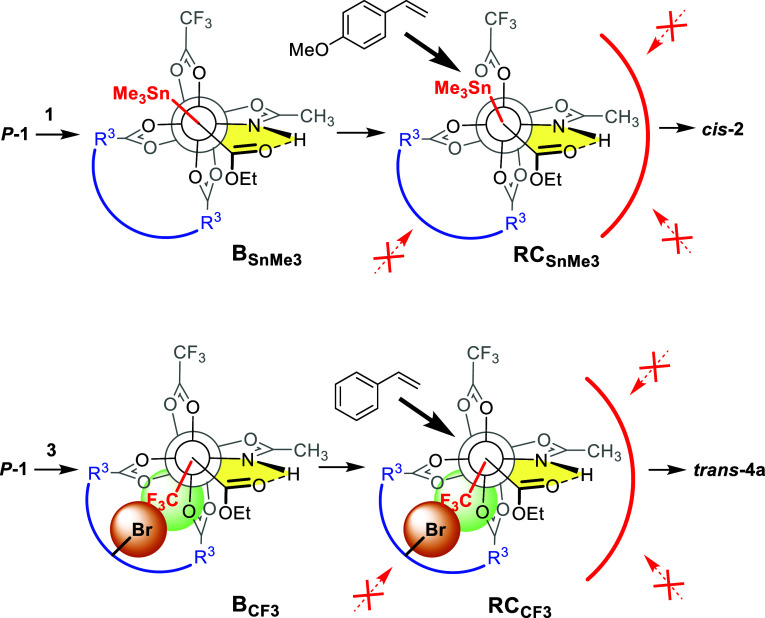
Different Stereochemical Course of the Cyclopropanation Reactions Using the Trimethylstannyl Substituted Diazoester **1** (Top) and Its Trifluoromethyl Substituted Analogue **3** (Bottom) Catalyzed by Complex *P* **-1** with “Backbone Chirality”

Yet another result of the DFT calculations merits explicit mentioning. Whereas the reaction of the stannylated carbene **B**_**SnMe**_3__ with styrene leading to **2** has to overcome a small but distinct transition state, a continuous decrease in energy was observed when the distance between this olefin and **B**_**CF**_3__ was decreased from 5.53 Å as the starting point of the calculations to 1.51 Å corresponding to the bond length in the final cyclopropane product **4a**.^48^ The fact that the reaction is barrierless is deemed to reflect the massively increased electrophilicity of **B**_**CF**_3__ caused by the strong inductive effect of the −CF_3_ substituent on an already highly electron deficient rhodium carbene center.^49^ Such massively upregulated reactivity has been blamed for the fact that it was previously impossible to carry out these reactions with any meaningful level of asymmetric induction, even with the help of well-established rhodium catalysts.^16^

### Catalytic Asymmetric Trifluoromethylcyclopropanation

The newly gained mechanistic understanding for what would otherwise be a perplexing stereochemical pattern made us explore the scope of the trifluoromethylcyclopropanation reaction catalyzed by complex *P* **-1** with confidence. First and foremost, confirmation was sought for the assumption that pervades all considerations so far, namely that peripheral hydrogen bonding between the −NH group of the equatorial carboxamidate ligand and the ester carbonyl group of the carbene formed at the axial coordination site is a key reactivity and selectivity determinant. To this end, a control experiment was carried out with the analogous complex *P* **-2** ^1^ comprising an *N*-methylated acetamidate (Table 1, entry 2); for the poor solubility of *P* **-2** in pure pentane, a pentane/CH_2_Cl_2_ (15:1 *v/v*) mixture had to be used. The reaction was hardly diastereoselective and the ee of *trans*-**4a** dropped to 28%, which is much lower than that obtained with our archetype catalyst *P* **-1** independent of the chosen solvent (compare entries 1, 6–13). This comparison proves that the protic site in *P* **-1** is quintessential for high asymmetric induction.

**Table 1:**
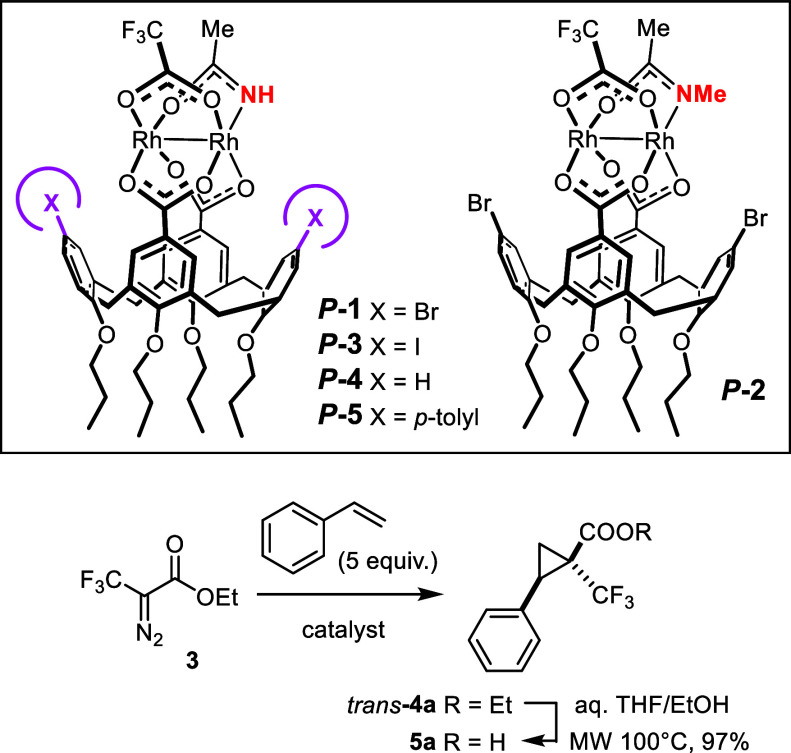
Reaction Optimization

entry	catalyst	mol %	solvent	*T* [°C]	yield [%]^a^ [NMR]	*trans*/*cis*	ee [%]
1	*P* **-1**	0.5	CH_2_Cl_2_/pentane	–20	65	3:1	89
2	*P* **-2**	0.5	CH_2_Cl_2_/pentane	–20	55 [64]	1.5:1	28
3	*P* **-3**	1.0	CH_2_Cl_2_	RT	59 [78]	4.5:1	73
4	*P* **-4**	1.0	CH_2_Cl_2_	RT	[<5]	2.5:1	n.d.
5	*P* **-5**	1.0	CH_2_Cl_2_	RT	[<5]	4.5:1	n.d.
6	*P* **-1**	1.0	CH_2_Cl_2_	RT	79 [86]	4:1	75
7	*P* **-1**	1.0	CH_2_Cl_2_	–20	[66]	6:1	78
8	*P* **-1**	1.0	C_6_H_5_CF_3_	RT	60 [69]	4.5:1	78
9	*P* **-1**	1.0	DCE	RT	36 [56]	4:1	73
10	*P* **-1**	1.0	pentane	RT	75 [87]	6:1	85
11	*P* **-1**	1.0	pentane	–20	77 [89]	8.5:1	93
12	*P* **-1**	0.5	pentane	–40	75 [85]	12:1	95
13	*P* **-1**	0.1	pentane	–40	72 (1.13 g)^b^	12:1	96

a Combined yield of the *trans* and *cis* isomers.

b Amount of analytically pure *trans*-**4a** after flash chromatography.

Additional control experiments revealed the equally pivotal role of the bromine substituents on the calixarene ligand. While formal replacement by iodide as manifested in complex *P* **-3** (X = I) hardly affected the outcome (entry 3), the use of *P* **-4** (X = H) as catalyst essentially failed to afford the desired product (entry 4);^50^ equally poor conversion was observed with complex *P* **-5** carrying *p*-tolyl substituents in lieu of the bromine atoms (entry 5). These data nicely support and even extend the conclusions drawn from the DFT studies that dispersive interactions between the –CF_3_ group flanking the carbene center and the halide atoms on the ligand scaffold are of paramount importance for reactivity and selectivity alike.

A short screening showed that *P* **-1** works best in pentane at low temperatures where the interligand hydrogen bond is presumably very tight (entries 6–12). Under the optimized conditions, the reaction scaled well, furnishing 1.13 g of analytically pure *trans*-**4a** with impeccable 96% ee, even though the catalyst loading was reduced to 0.1 mol % in this case (entry 13). The ester group of **4a** can be hydrolyzed by microwave irradiation of the sample in aqueous EtOH/THF under essentially neutral conditions to prevent epimerization of the base-labile benzylic center. As shown by our group, acids such as **5a** are valuable building blocks that could previously only be made in optically active form by an indirect route.^24^

Other styrene derivatives performed equally well, largely independent of whether they carry electron-withdrawing or —donating substituents on the phenyl ring (Scheme 3). The actual substitution pattern does not seem to matter very much, as witnessed by the *para*-, *meta*-, or *ortho*-substituted products **4b**-**g**, all of which have optical purities of 90–96% ee. Indene performed even better, in that the minor diastereomer was at the limit of detection (dr ≈ 50:1, NMR) and the ee of the *trans*-isomer **4h** was truly excellent. *N*-Vinylcarbazole as well as phenyl vinyl ether were found to be equally adequate reaction partners, affording cyclopropanes **4i** and **4j** functionalized with a heteroatom handle in high optical purity. Very respectable results were also obtained with an ordinary 1,3-diene, a dienyl silyl ether, as well as a 1,3-enyne, all of which reacted exclusively at the less substituted double bond to furnish products **4m**–**o**. Even the cyclopropenation of phenylacetylene was possible, although the corresponding product **4p** was obtained with a more modest ee of 72%.

**Scheme 3:**
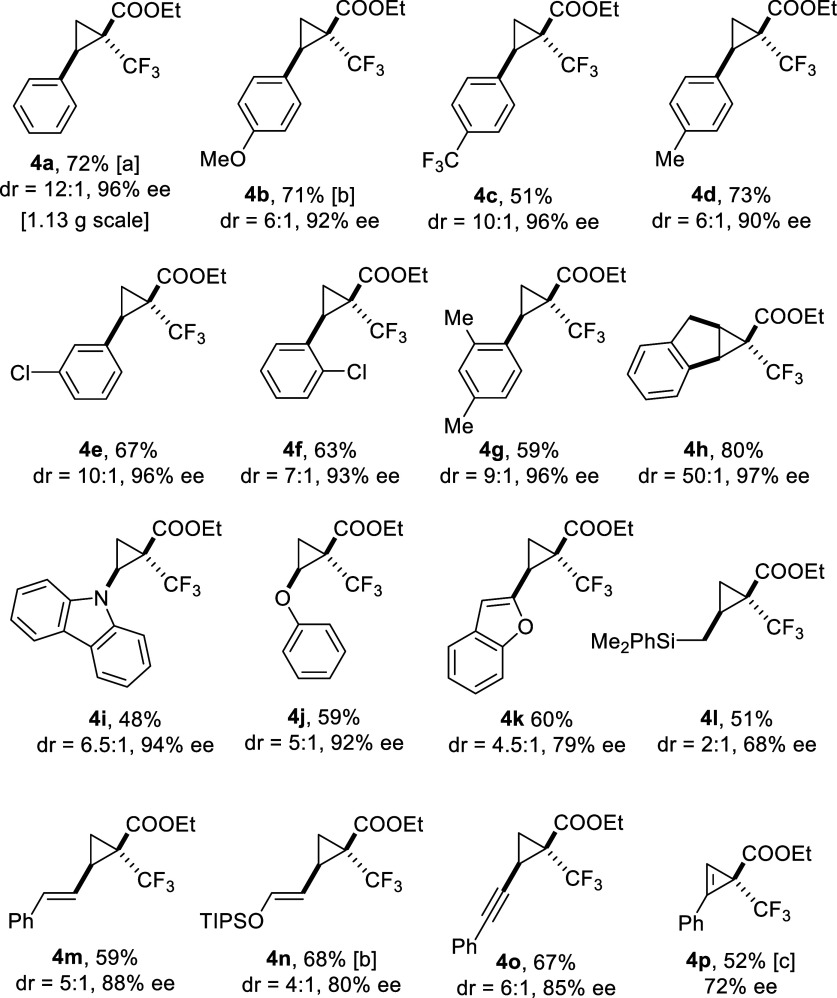
Scope of the Asymmetric Cyclopropanation with Ethyl 3,3,3-Trifluoro-2-diazopropionate (**3**) Catalyzed by the Heterochiral-at-The-Metals Complex *P* **-1** (0.5 mol %); All Reactions Were Performed in Pentane at −40 °C^a^

a Unless stated otherwise, the yields refer to pure *trans*-isomer obtained after flash chromatographic purification, the dr (^19^F NMR) to the product ratio in the crude material; [a] using 0.1 mol % of *P* **-1**; [b] a *cis*/*trans* mixture was isolated; [c] at RT.

Aliphatic alkenes are less adequate substrates; allyl­(dimethylphenyl)­silane, is representative in that the diastereo- and enantioselectivity of the derived product **4l** are lower. The reasons why styrene derivatives perform significantly better were investigated by ADLD analysis^43^ on a representative structure along the minimum-energy path toward product formation. Specifically, we selected the geometry where the forming C–C bond length (3.13 Å) closely matches that observed in the transition state of the stannylated carbene (for details, see the Supporting Information). Atomic dispersion energies were computed for the full structure, for the styrene fragment in its fixed geometry, and for the carbene intermediate in the same geometry. Subtracting the latter two from the complete system isolates the styrene–carbene dispersive interaction. Summing the atomic values for the benzene ring yields a stabilizing interaction of 5.1 kcal mol^–1^. This substantial dispersive effect rationalizes the reduced stereoselectivity observed with olefins bearing aliphatic substituents, which are expected to lack analogous stabilization.

Another limitation is encountered with substrates that are insoluble in pentane at low temperature, which cannot be processed with the aid of this “first generation” catalyst *P* **-1** (e.g., N-vinylphthalimide, 2-vinylthiophene, 2-vinylnaphthalene). We are exploring ligand modifications that might eventually allow the catalyst to operate in somewhat more polar media without loss in selectivity.

Numerous ways can be envisaged to leverage the reactivity of the ester, the substituted arene (or lateral double (triple) bond), or the heteroelement displayed on the cyclopropyl ring of compounds of type **4**. For example, a Curtius rearrangement of acid **5d** affords the corresponding trifluoromethylated carbamate; this reaction has already previously been shown to proceed with full retention of the relative and absolute configuration (Scheme 4).^51^ The same is true for the incorporation of **5d** into a nicely crystalline pseudodipeptide, which allowed the relative and absolute stereostructure assigned to the trifluoromethylated cyclopropyl building block to be confirmed by X-ray diffraction analysis.^51^

**Scheme 4:**
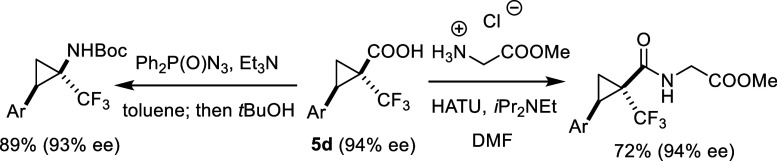
Exemplary Downstream Functionalization Reactions with Retention of Configuration; Ar = *p*-Tolyl^51^

### Extension

It seemed possible to harness dispersive interactions between fluorinated substrates and the strategically placed bromine substituents in the backbone-chiral complex also in other settings. Ordinary donor/acceptor carbenes might provide such an opportunity, because our new catalyst performed poorly when applied to a sterically unencumbered diazoesters such as **6** (the enantiomer *M* **-1** was used in this case) (Scheme 5).^52^ Therefore, it was tempting to probe whether formal perfluorination of the phenyl ring leads to better results.

**Scheme 5:**
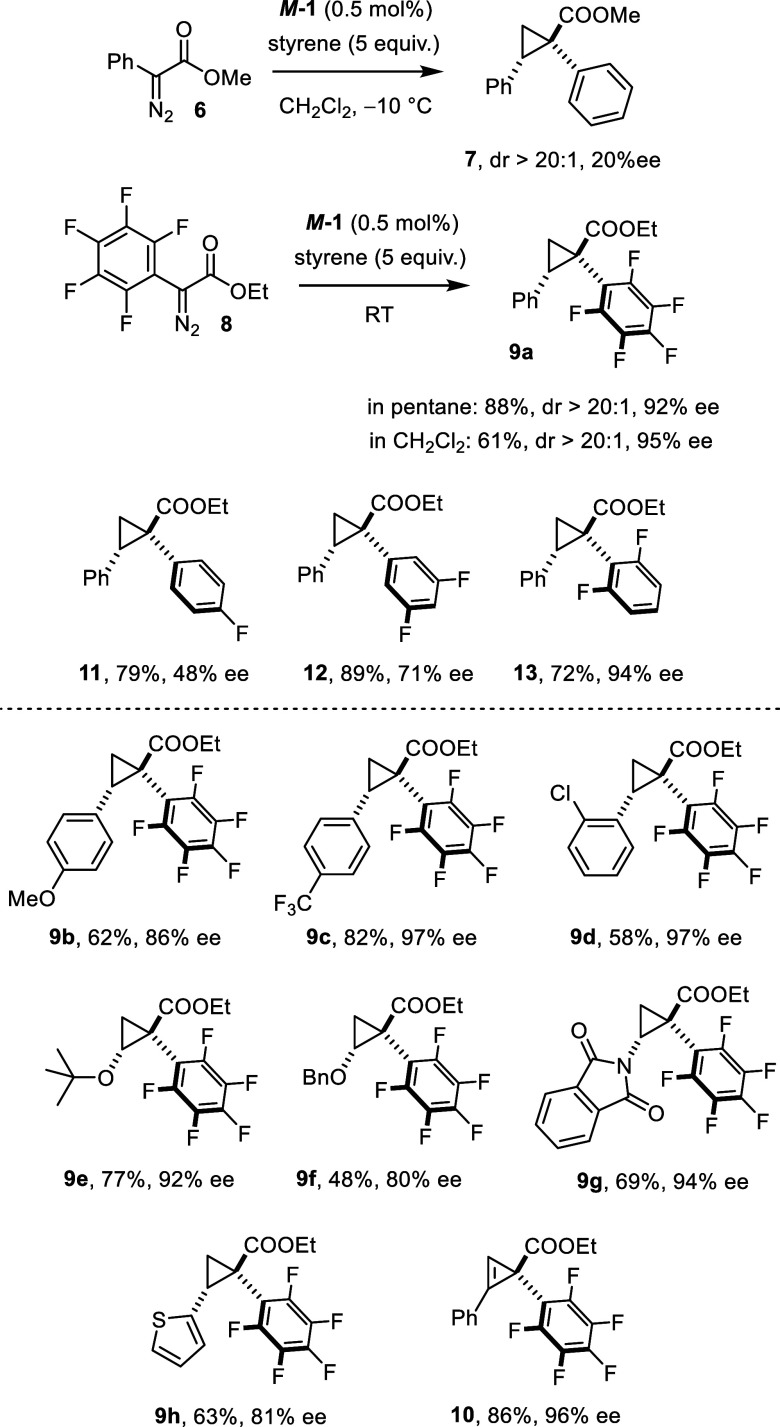
Harnessing the Fluoride Effect in Other Asymmetric Cyclopropanations Catalyzed by Complex *M* **-1**; all Reactions Were Performed in CH_2_Cl_2_ at Ambient Temperature; the dr (NMR) was ≥20:1 in All Cases Investigated

This is indeed the case, even though this chemical modification makes the aryl ring a much less good donor substituent to the adjacent metal carbene center;^53–57^ mitigating the favorable stabilizing effect by rendering the substituent less electron-rich potentially impacts on the ee’s obtained.^58^ Anyway, the reaction of **8** with styrene catalyzed by *M* **-1** in pentane at ambient temperature afforded the corresponding product **9a** essentially as a single *trans*-isomer with high optical purity (dr > 20:1, 92% ee). Gratifyingly, CH_2_Cl_2_ proved equally suitable in this case, resulting in an even higher ee (95%). Since cyclopropanes of this type comprising an all-carbon quaternary stereocenter with a pentafluorophenyl group and an ester substituent have so far not been made in catalytic asymmetric fashion, a small set of other substrates was surveyed. Once again, various styrene derivatives as well as vinyl ethers were found to be well behaved. Furthermore, the reaction extends to substrates such as 2-vinylthiophene and *N*-vinylphthalimide, which are insoluble in pentane (see above) but dissolve in CH_2_Cl_2_ and can hence be processed in the present setting. In all cases investigated, the resulting products were attained with good to excellent optical purity (Scheme 5). It is also important to note that the diastereoselectivity in favor of the *trans*-isomer was excellent (≥20:1). Gratifyingly, the prototype cyclopropenation reaction of phenylacetylene leading to **10** was equally successful.

A brief scan allowed us to narrow down which of the fluorine substituents in **8** is accountable for the auspicious outcome. As one might expect, cyclopropane **11** was formed with rather poor enantioselectivity, because, for geometric reasons, the *para*-fluoro substituent on the phenyl ring is hardly able to engage in dispersive interactions with the bromine atoms adorning the calix[4]­arene ligand of *M* **-1** in the stereodetermining transition state. The analogous cyclopropane **12** endowed with a *meta*-difluorinated phenyl substituent showed a better but still modest optical purity. In striking contrast, compound **13** with the *ortho*-difluorinated phenyl ring was highly enantioenriched (94% ee). This clear correlation between the position of the fluorine substituents and the optical purity of the resulting product can easily be reconciled with a dispersive model akin to the rationale outlined above for the trifluoromethyl derivative **3** (see Scheme 2); moreover, the observed trend strongly encourages the search for yet other fluorinated carbene sources that might also qualify for highly enantioselective transformations catalyzed by the novel backbone-chiral complex.

## Conclusions

Rhodium catalyzed asymmetric cyclopropanations with 3,3,3-trifluoro-2-diazopropionate have been a largely unsolved problem in the past, except for a special class of electron-deficient allylsulfones that have been found compliant.^26^ This challenging transformation is now shown to be accomplished with excellent diastereo- and enantioselectivity with the aid of complex ***P***-**1** (or its enantiomer *M* **-1**) as the catalyst. This unorthodox dirhodium paddlewheel complex comprises of a core of two inequivalent chiral metal centers surrounded by a set of three different achiral equatorial ligands. One of these μ_2_-bridging ligands is a carboxamidate, the protic –NH group of which plays a key role: it determines the site of carbene formation and is quintessential for controlling the trajectory by which an olefinic reaction partner will approach the reactive carbene center. The ensuing cyclopropanation benefits from an additional stabilizing force, namely the dispersive interaction between the −CF_3_ substituent flanking the superelectrophilic metal carbene center and a strategically placed bromide substituent on the catalyst’s calix[4]­arene dicarboxylate ligand backbone. This interhalogen contact determines the conformation of the reactive intermediate and entails a well-defined stereodetermining transition state, which in turn translates into excellent optical purity of the resulting trifluoromethylated products. Importantly, analogous dispersive forces can also benefit catalytic asymmetric cyclopropanations leading to building blocks with fluorinated substituents other than −CF_3_. Further extensions of the reaction scope are actively pursued in our laboratory, as are explorations of the highly enabling reactivity of our prototype catalyst *P-* **1** and complementary investigations into other types of chiral-at-the-metal complexes.^59^

## Supplementary Material



## Notes

Experimental Section including characterization data and NMR spectra of new compounds, and Computational Study (PDF)
