# Supplementary material for: Dispersive Fluorine/Bromine Interactions as Key Selectivity Determinant: Asymmetric Cyclopropanations with 3,3,3-Trifluoro-2-diazopropionate Catalyzed by a Heterochiral-at-The-Metal Centers Dirhodium Paddlewheel Complex
Source: J Am Chem Soc. 2025 Dec 18;148(1):1337–46. doi: 10.1021/jacs.5c17842 (PMC12814317; doi:10.1021/jacs.5c17842)
Supplement: Supplementary file 1 [file ja5c17842_si_001.pdf]

SUPPORTING INFORMATION

Dispersive Fluorine/Bromine Interactions as Key Selectivity Determinant: Asymmetric Cyclopropanations with 3,3,3-Trifluoro-2-diazo-propionate Catalyzed by a Heterochiral-at-the-Metal Centers Dirhodium Paddlewheel Complex

Matthias Peeters,[a] Lucas Marchal,[a] Sofia Lerda,[b] Giovanni Bistoni,[b] and Alois Fürstner*,[a]

Email: fuerstner@kofo.mpg.de

[a] *Max-Planck-Institut für Kohlenforschung, Mülheim/Ruhr, Germany*

[b] *Department of Chemistry, Biology, and Biotechnology, University of Perugia, I-06123 Perugia, Italy*

Table of Contents

Experimental Section	S2
General	S2
New Ligands and Catalysts	S3
Cyclopropanations Using Ethyl 3,3,3-Trifluoro-2-diazopropionate	S8
Gram-Scale Cyclopropanation with Reduced Catalyst Loading	S24
Ester Cleavage	S25
Cyclopropanations with Donor/Acceptor Carbenes Carrying Fluorinated Phenyl Substituents	S26
Substrates	S26
General Procedure	S28
Computational Section	S40
Electronic Circular Dichroism	S40
Structural Identification of the Carbene Intermediate	S41
Analysis of Selectivity	S44
Dispersive Effects Impacting on Diastereo- and Enantioselectivity	S47
Optimized Cartesian Coordinates	S49
NMR Spectra of New Compounds	S60
References	S143

Experimental Section

General

Unless stated otherwise, all reactions were carried out under argon atmosphere in flame-dried Schlenk glassware, ensuring inert conditions. The solvents were purified by distillation over the indicated drying agents and were transferred under argon: THF, Et_2O (Mg/anthracene); pentane, toluene (Na/K); CH_2Cl_2, DCE (CaH_2). Anhydrous α,α,α-trifluorotoluene (TFT) was bought from Sigma Aldrich and used as received.

Flash chromatography: Merck Geduran silica gel 60 (40 – 63 µm). Thin layer chromatography (TLC): Macherey-Nagel precoated plates (POLYGRAM®SIL/UV254); visualization by UV light (254 nm) and by staining with solutions of phosphomolybdic acid (PMA), $KMnO_4$ or cerium ammonium nitrate (CAN).

NMR spectra were recorded on a Bruker Avance III HD nanobay 300, Avance III HD 400, Avance III 500 or Avance Neo 600 MHz NMR spectrometer. ^1H and ^{13}C NMR chemical shifts are given in ppm relative to Me_4Si (δ = 0 ppm), coupling constants (*J*) in Hz. ^1H and ^{13}C NMR chemical shifts were referenced using the solvent signals as internal reference.[1] For ^1H NMR the following residual proton peaks of the deuterated solvents were used: $CDCl_3$: $\delta_H(CHCl_3)$ = 7.26 ppm; for ^{13}C NMR: $CDCl_3$: δ = 77.16 ppm. ^{19}F NMR chemical shifts are reported relative to CCl_3F. Unless stated otherwise, all ^{13}C, ^{19}F and spectra were recorded in {^1H}-decoupled manner.

IR: Alpha Platinum ATR (Bruker), wavenumbers ($\tilde{\nu}$) in cm^{-1}.

MS (EI): Finnigan MAT 8200 (70 eV), ESI-MS: ESQ 3000 (Bruker) or Thermo Scientific LTQ-FT or Thermo Scientific Exactive Spectrometer. HRMS: Bruker APEX III FT-MS (7 T magnet), MAT 95 (Finnigan), Thermo Scientific LTQ-FT or Thermo Scientific Exactive Spectrometer.

Optical rotations were measured with an A-Krüss Otronic Model P8000-t polarimeter at a wavelength of 589 nm. The values are given as specific optical rotation with exact temperature, concentration (c in g/100 mL) and solvent.

HPLC: Analytical LC analyses were performed on a Shimadzu LC 2020 instrument equipped with a Shimadzu SPD-M20A UV/VIS detector or Agilent system equipped with a G4212A 1290 DAD.

The circular dichroism spectra were measured on a J-1100 CD UV-Visible/NIR spectrometer; high precision Suprasil Hellma Analytics cuvettes made of quartz were used for the measurements.

New Ligands and Catalysts

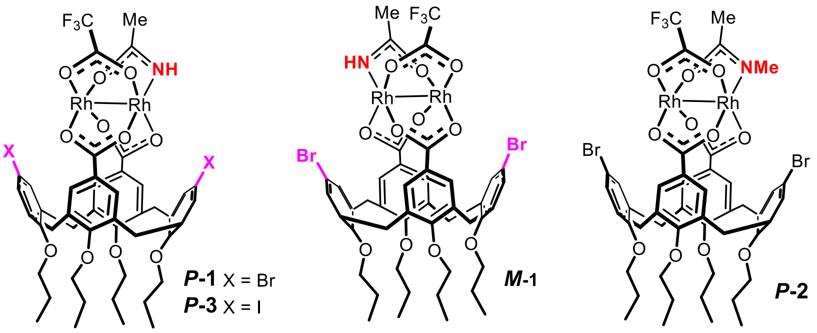

Complexes ***M*-1**, ***P*-1**, ***P*-2**, ***P*-3**,[2] and ethyl 3,3,3-trifluoro-2-diazopropionate (**3**) were prepared according to literature procedures. Unless stated otherwise, all commercially available compounds (Alfa Aesar, Sigma Aldrich, TCI, BLD Pharm and ABCR) were used as received.

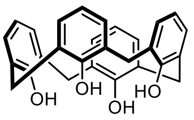

Calix[4]arene S1. Commercial 4-*tert*-butylcalix[4]arene (5.0 g, 7.7 mmol) was suspended in dry toluene (50 mL) and $AlCl_3$ (5.87 g, 46.0 mmol) and phenol (3.76 g, 46.0 mmol) were added. The mixture was stirred at room temperature for 4 h before it was poured into aq. HCl (0.2 M). The aqueous layer was extracted with ethyl acetate (3 x 50 mL), and the combined organic phases were dried with $MgSO_4$, filtered and concentrated under reduced pressure. Precipitation from methanol and filtration yielded the title compound as a white powder (2.74 g, 74%). ^{1}H NMR (400 MHz, $CDCl_3$) δ 10.22 (s, 4H), 7.07 (d, *J* = 7.5 Hz, 8H), 6.75 (t, *J* = 7.6 Hz, 4H), 4.28 (br s, 4H), 3.56 (br s, 4H). ^{13}C NMR (101 MHz, $CDCl_3$) δ 148.9, 129.1, 128.4, 122.4, 31.9.

The spectral data match the literature.[3]

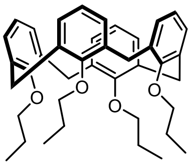

Calix[4]arene S2. NaH (565 mg, 23.6 mmol) was added to a solution of calix[4]arene **S1** (1.0 g, 2.36 mmol) in DMF (20 mL) and the mixture was stirred at room temperature for 30 min. 1-Iodopropane (2.29 mL, 23.6 mmol) was added and the mixture was stirred at room temperature for 15 h. Aqueous HCl (2 M, 20 mL) was slowly added and the precipitate was filtered off, washed with water and dried. The crude material was purified by recrystallization from methanol to give the title compound as a white solid (0.94 g, 67%). ^{1}H NMR (400 MHz, $CDCl_3$) δ 6.65 – 6.51 (m, 12H), 4.46 (d, *J* = 13.3 Hz, 4H), 3.85 (dd, *J* = 8.0, 7.0 Hz, 8H), 3.15 (d, *J* = 13.4 Hz, 4H), 1.99 – 1.86 (m, 8H), 1.00 (t, *J* = 7.5 Hz, 12H). ^{13}C NMR (101 MHz, $CDCl_3$) δ 156.7, 135.3, 128.3, 122.0, 76.9, 31.1, 23.4, 10.5.

The spectral data match the literature.[4]

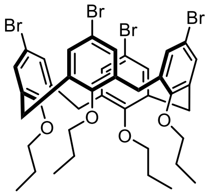

Calix[4]arene S3. *N*-Bromosuccinimide (2.35 g, 13.2 mmol) was added to a solution of calix[4]arene **S2** (0.90 g, 1.52 mmol) in DMF (30 mL). The mixture was stirred at room temperature for 24 h before aqueous HCl (1 M, 60 mL) was slowly added. The yellow precipitate was filtered off, washed with water and dried. Recrystallization from CH_2Cl_2/MeOH afforded the title compound as a white powder (1.06 g, 77%). ^{1}H NMR (400 MHz, $CDCl_3$) δ 6.80 (s, 8H), 4.35 (d, *J* = 13.4 Hz, 4H), 3.80 (dd, *J* = 8.1, 7.0 Hz, 8H), 3.08 (d, *J* = 13.5 Hz, 4H), 1.87 (h, *J* = 7.5 Hz, 8H), 0.97 (t, *J* = 7.4 Hz, 12H). ^{13}C NMR (101 MHz, $CDCl_3$) δ 155.7, 136.6, 131.2, 115.3, 77.2, 30.9, 23.2, 10.4.

The spectral data match the literature.[5]

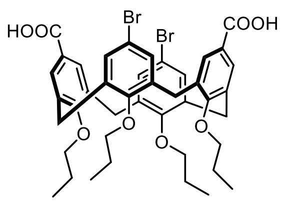

Calix[4]arene S4. *n*-BuLi (1.6 M in hexane, 2.0 mL, 3.17 mmol) was added at −78 °C to a solution of calix[4]arene **S3** (1.1 g, 2.32 mmol) in THF (45 mL). The resulting yellow solution was stirred at −78 °C for 30 min before CO_2 gas was bubbled through the mixture for 30 min. The reaction mixture was poured into ice-cold HCl (2 M, 20 mL) and the aqueous layer was extracted with $CHCl_3$ (3 x 20 mL). The combined organic phase was washed with water (50 mL) and dried over $MgSO_4$, filtered, and concentrated under reduced pressure. The residue was triturated in ethanol and the solid material filtered off to afford the title compound as a white solid (782 mg, 77 %). ^{1}H NMR (400 MHz, $CDCl_3$) δ 12.83 (s, 2H), 7.32 (s, 4H), 6.83 (s, 4H), 4.38 (d, *J* = 13.8 Hz, 4H), 4.00 – 3.92 (m, 4H), 3.64 (t, *J* = 6.7 Hz, 4H), 3.12 (d, *J* = 13.9 Hz, 4H), 1.85 (qd, *J* = 8.3, 4.8 Hz, 8H), 1.09 (t, *J* = 7.4 Hz, 6H), 0.84 (t, *J* = 7.4 Hz, 6H). ^{13}C NMR (101 MHz, $CDCl_3$) δ 171.8, 159.9, 157.0, 138.6, 133.3, 132.0, 129.9, 123.8, 115.1, 77.2, 76.7, 31.0, 23.6, 22.9, 10.9, 9.8.

The spectral data match the literature.[5]

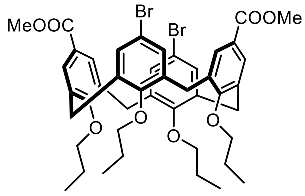

Calix[4]arene S5. DMAP (17.5 mg, 0.14 mmol) and MeOH (87 µL, 2.15 mmol) were added to a solution of calix[4]arene **S4** (600 mg, 0.72 mmol) in DMF/ CH_2Cl_2 (1:2 *v/v*, 9 mL). The mixture was cooled to 0 °C and stirred for 15 min prior to the portion-wise addition of EDC (412 mg, 2.15 mmol). After stirring for 4 h, the reaction mixture was diluted with water and the aqueous layer was extracted with CH_2Cl_2 (3 x 40 mL). The combined organic phase was washed with brine (2 x 40 mL), dried over $MgSO_4$, filtered and concentrated under reduced pressure. The residue was purified by flash chromatography (silica; hexane/EtOAc, 10:1 to 7:1) to give the title compound as a white solid (542 mg, 87 %). ^{1}H NMR (400 MHz, $CDCl_3$) δ 7.70 (s, 4H), 6.45 (s, 4H), 4.40 (d, *J* = 13.5 Hz, 4H), 4.08 – 4.00 (m, 4H), 3.69 (t, *J* = 7.0 Hz, 4H), 3.20 (d, *J* = 13.5 Hz, 4H), 1.98 – 1.80 (m, 8H), 1.06 (t, *J* = 7.4 Hz, 6H), 0.90 (t, *J* = 7.5 Hz, 6H). ^{13}C NMR (101 MHz, $CDCl_3$) δ 167.1, 161.7, 154.8, 136.0, 135.4, 130.8, 130.7, 124.4, 115.5, 77.5 (overlap $CDCl_3$), 77.0, 52.1, 31.0, 23.5, 23.2, 10.7, 10.0.

The spectral data match the literature.[2]

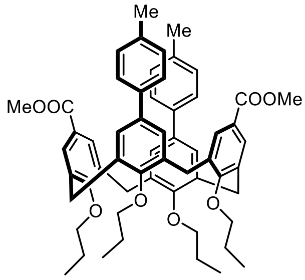

Calix[4]arene S6. A two-neck round bottom flask was charged with calix[4]arene **S5** (100 mg, 0.12 mmol), K_2CO_3 (96 mg, 0.7 mmol), *p*-tolueneboronic acid (63 mg, 0.46 mmol), MeOH (2 mL) and toluene (3 mL). After stirring for 15 min at room temperature, $PdCl_2$(dppf) (12 mg, 0.017 mmol, 15 mol%) was added and the mixture was stirred at 80 °C (bath temperature) for 24 h. After reaching room temperature, the mixture was diluted with water and the aqueous layer extracted with CH_2Cl_2 (3 × 15 mL). The combined organic phases were washed with water (20 mL) and brine (20 mL), dried over $MgSO_4$ and concentrated under reduced pressure. The residue was purified by flash chromatography (silica; hexane/EtOAc, 12:1 to 7:1) to give the title compound as a white solid (65 mg, 63 %). ^{1}H NMR (400 MHz, $CDCl_3$) δ 7.57 (s, 4H), 6.83 (s, 8H), 6.67 (s, 4H), 4.50 (d, *J* = 13.3 Hz, 4H), 4.10 – 4.01 (m, 4H), 3.83 (s, 6H), 3.81 (t, *J* = 7.3 Hz, 4H), 3.28 (d, *J* = 13.4 Hz, 4H), 2.27 (s, 6H), 2.04 – 1.86 (m, 8H), 1.05 (t, *J* = 7.4 Hz, 6H), 0.99 (t, *J* = 7.4 Hz, 6H). ^{13}C NMR (101 MHz, $CDCl_3$) δ 167.2, 161.5, 155.4, 138.0, 135.9, 135.6 (d, *J* = 1.8 Hz), 133.8, 130.2, 129.0, 126.9, 126.6, 123.9, 77.3, 77.0, 51.9, 31.3, 23.5, 23.3, 21.2, 10.6, 10.3. IR (ATR): ṽ = 2960, 2933, 2875, 1719, 1464, 1434, 1311, 1230, 1197, 1183, 1005, 964, 814, 769 cm^{-1}. HRMS (ESI+): *m/z* calcd. for $C_{58}H_{64}O_8Na$ [M+Na]$^+$: 911.44934; found: 911.44875.

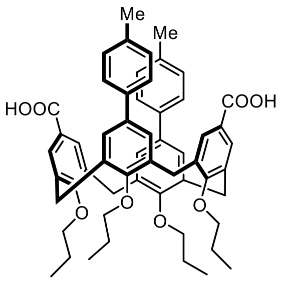

Calix[4]arene S7. KOTMS (267 mg, 2.1 mmol) was added to a solution of calix[4]arene **S6** (200 mg, 0.21 mmol) in THF (7 mL) and the resulting mixture was stirred at room temperature for 3 d. An aq. solution of citric acid (3 M, 1 mL) was carefully added and the mixture left stirring for 1 h. The mixture was diluted with water and the aqueous layer extracted with CH_2Cl_2 (3 x 20 mL). The combined organic phases were washed with brine (50 mL), dried over $MgSO_4$, filtered and concentrated under reduced pressure. The residue was washed with cold pentane (2 x 3 mL) to afford the title compound as a white solid (28 mg, 98 %). ^{1}H NMR (400 MHz, $CDCl_3$) δ 12.49 (s, 2H), 7.58 (d, *J* = 7.7 Hz, 4H), 7.38 (s, 4H), 7.25 (d, *J* = 6.4 Hz, 4H), 6.82 (s, 4H), 4.46 (d, *J* = 13.6 Hz, 4H), 4.07 – 3.97 (m, 4H), 3.67 (q, *J* = 6.9 Hz, 4H), 3.21 (d, *J* = 13.7 Hz, 4H), 2.44 (s, 6H), 1.90 (dq, *J* = 20.9, 7.5 Hz, 8H), 1.11 (t, *J* = 7.4 Hz, 6H), 0.86 (t, *J* = 7.4 Hz, 6H). ^{13}C NMR (101 MHz, $CDCl_3$) δ 171.3, 159.9, 157.3, 138.3, 136.8, 136.2, 135.3, 133.7, 129.9, 129.4, 127.8, 127.2, 123.7, 77.0 (overlap $CDCl_3$), 76.7, 31.4, 23.6, 23.0, 21.3, 11.0, 9.9. IR (ATR): ṽ = 2959, 2917, 2871, 2851, 1696, 1679, 1470, 1421, 1307, 1278, 1237, 1204, 1194, 1104, 1034, 997, 962, 905, 817 cm^{-1}. HRMS (ESI +): *m/z* calcd. for $C_{56}H_{60}O_8Na$ $[M+Na]^+$: 883.4180; found: 883.41759.

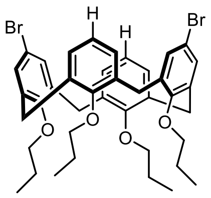

Calix[4]arene S8. *n*-BuLi (1.6 M in hexane, 1.8 mL, 2.9 mmol) was added at –78 °C to a solution of calix[4]arene **S3** (1.1 g, 1.21 mmol) in THF (45 mL). The resulting orange solution was stirred at –78 °C for 15 min before the reaction was quenched with MeOH (0.5 mL). After stirring for an additional 10 min, the mixture was poured into ice cold aq. HCl (2 M) and the aqueous layer was extracted with $CHCl_3$ (3 x 20 mL). The combined organic phase was washed with water (50 mL) and dried over $MgSO_4$, filtered and concentrated under reduced pressure to afford the title compound as a white solid (680 mg, 75 %). ^{1}H NMR (400 MHz, $CDCl_3$) δ 6.79 (s, 4H), 6.65 (s, 6H), 4.42 (d, *J* = 13.4 Hz, 4H), 3.84 (q, *J* = 7.6 Hz, 8H), 3.13 (d, *J* = 13.4 Hz, 4H), 2.01 – 1.84 (m, 8H), 1.07 – 0.95 (m, 12H). ^{13}C NMR (101 MHz, $CDCl_3$) δ 156.5, 155.8, 137.4, 134.5, 130.9, 128.5, 122.6, 114.8, 77.0, 76.9, 31.0, 23.3, 10.4, 10.4.

The spectral data match the literature.[5]

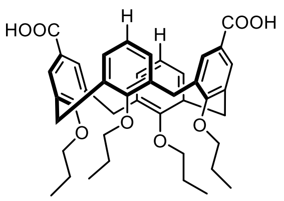

Calix[4]arene S9. *n*-BuLi (1.6 M in hexane, 1.3 mL, 2.1 mmol) was added at –78 °C to a solution of calix[4]arene **S8** (600 mg, 0.80 mmol) in THF (20 mL). The resulting yellow solution was stirred at –78 °C for 30 min before CO_2 gas was bubbled through the mixture for 30 min. The reaction mixture was then poured into ice cold HCl (2 M) and the aqueous layer was extracted with $CHCl_3$ (3 x 20 mL). The combined organic phase was washed with water (50 mL) and dried with $MgSO_4$, filtered and concentrated under reduced pressure. The residue was triturated in ethanol and filtered off to afford the title compound as a white solid (122 mg, 22 %). ^{1}H NMR (400 MHz, $CDCl_3$) δ 12.91 (s, 2H), 7.18 (d, *J* = 7.4 Hz, 4H), 7.08 – 6.99 (m, 2H), 6.76 (s, 4H), 4.42 (d, *J* = 13.5 Hz, 4H), 4.04 – 3.95 (m, 4H), 3.66 (t, *J* = 6.6 Hz, 4H), 3.15 (d, *J* = 13.6 Hz, 4H), 1.99 – 1.79 (m, 8H), 1.10 (t, *J* = 7.4 Hz, 6H), 0.86 (t, *J* = 7.5 Hz, 6H). ^{13}C NMR (101 MHz, $CDCl_3$) δ 172.2, 159.9, 157.7, 136.7, 133.8, 129.8, 129.5, 123.3, 123.0, 77.0, 76.6, 31.0, 23.6, 23.1, 10.9, 9.9.

The spectral data match the literature.[5]

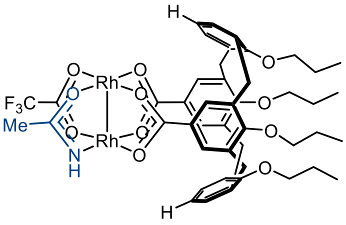

Complex *P*-4. A two-neck flask was equipped with a Soxhlet extractor (returning-arm frit) topped by a reflux condenser. The Soxhlet extractor was filled with an oven-dried mixture of K_2CO_3 and sand (1:1, ca. 5 g). The flask was charged with calix[4]arene **S9** (40 mg, 0.060 mmol), [Rh_2(acam)(Otfa)] (30 mg, 0.050 mmol), *tert*-butyl acetate (8 mL), and toluene (2 mL). Argon was bubbled through the mixture for 15 min before the flask was immersed into a pre-heated oil bath (130 °C bath temperature). The mixture was stirred at reflux temperature such that a gentle flow of condensing solvent passed through the Soxhlet extractor. (***Note***: *Initially the reaction mixture is a suspension, but slowly becomes a transparent green solution as the reaction progresses*). After 5 h, the mixture was cooled to room temperature and concentrated under reduced pressure. The residue was purified by flash chromatography (silica; toluene/acetonitrile, 12:1 to 7:1) to afford the title compound as a green solid (15.4 mg, 29%). ^{1}H NMR (600 MHz, [D_8]-THF) δ 7.17 (dt, *J* = 6.4, 1.7 Hz, 2H), 7.14 (dd, *J* = 14.5, 1.9 Hz, 1H), 7.13 (dd, *J* = 14.1, 1.7 Hz, 1H), 6.99 – 6.93 (m, 1H), 6.96 – 6.91 (m, 1H), 6.67 (dd, *J* = 1.6, 0.8 Hz, 2H), 6.59 (d, *J* = 2.2 Hz, 1H), 6.55 (d, *J* = 2.2 Hz, 1H), 5.26 (s, 1H), 4.41 (d, *J* = 13.5 Hz, 2H), 4.40 (d, *J* = 13.5 Hz, 2H), 4.01 – 3.98 (m, 4H), 3.68 (t, 2H), 3.66 (t, *J* = 6.6 Hz, 2H), 3.15 – 3.07 (m, 4H), 1.92 – 1.87 (m, 4H), 1.87 – 1.82 (m, 4H), 1.78 (s, 3H), 1.10 (t, *J* = 7.4 Hz, 3H), 1.10 (t, *J* = 7.4 Hz, 3H), 0.86 – 0.81 (m, 6H). ^{13}C NMR (151 MHz, [D_8]-THF) δ 186.3, 185.8, 184.4, 172.0, 171.9 (q, *J* = 37.6 Hz), 171.7, 159.3, 159.1, 158.8, 158.7, 138.1, 138.0, 137.9, 137.9, 133.4, 133.3, 133.3, 133.1, 130.3, 130.2, 130.1, 130.1, 129.9, 129.9, 129.9, 129.5, 127.6, 126.7, 123.0, 122.9, 112.8, 111.9 (q, *J* = 285.5 Hz), 110.9, 77.6, 77.2, 77.3, 67.7, 67.5, 67.4, 67.3, 67.1, 31.8, 31.7, 25.6, 25.4, 25.3, 25.2, 25.0, 24.4, 24.4, 23.9, 23.4, 11.2, 11.2, 10.1. ^{19}F NMR (565 MHz, [D_8]-THF) δ –75.8. HRMS (ESI +): *m/z* calcd. for $C_{46}H_{50}F_3NO_{11}Rh_2Na$ [M+Na]$^+$: 1078.13367; found: 1078.13382.

Note: *The complex turned out to be unstable in solution. NMR spectra of samples recorded after the HPLC separation did show significant amounts (≥ 10%) of decomposition products*.

HPLC analysis: 150 mm Chiralcel IB-N3, Ø 4.6 mm i.D., *n*-heptane/ethanol = 98:2 1.0 mL / min, λ = 220 nm, t(enantiomer 1) = 4.40 min, t(enantiomer 2) = 6.74 min. The enantiomers were separated by preparative HPLC using following conditions: 150 mm Chiralpak IB-N5, 5µm, Ø 20.0 mm, *n*-hexane/ ethanol = 98:2, v= 15.0 mL / min, λ = 220 nm.

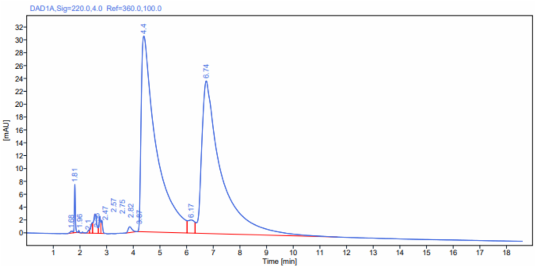

Signal Description DAD1A,Sig=220.0,4.0 Ref=360.0,100.0

Signal Type	Peak	tR [min]	Peak Area [%]	Name
DAD1	1	1.68	0.08	
DAD1	2	1.81	0.73	
DAD1	3	1.96	0.03	
DAD1	4	2.10	0.02	
DAD1	5	2.36	0.09	
DAD1	6	2.47	0.30	
DAD1	7	2.57	1.00	
DAD1	8	2.75	0.43	
DAD1	9	2.82	0.31	
DAD1	10	3.87	0.36	
DAD1	11	4.40	47.24	1. enantiomer
DAD1	12	6.17	1.38	
DAD1	13	6.74	48.02	2. enantiomer

HPLC trace of the enantiomers of complex ***P*-4**.

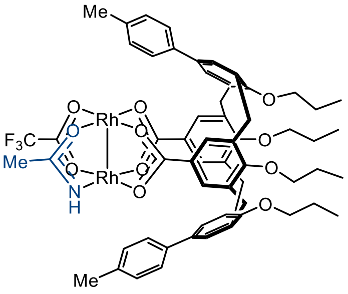

Complex *P*-5. A two-neck flask was equipped with a Soxhlet extractor (returning-arm frit) topped by a reflux condenser. The Soxhlet extractor was filled with an oven-dried mixture of K_2CO_3 and sand (1:1, ca. 5 g). The flask was charged with calix[4]arene **S7** (44 mg, 0.051 mmol), [Rh_2(acam)(Otfa)$_3$] (26 mg, 0.043 mmol), *tert*-butyl acetate (8 mL), and toluene (2 mL). Argon was bubbled through the mixture for 15 min before the flask was immersed into a pre-heated oil bath (130 °C bath temperature). The mixture was stirred at reflux temperature such that a gentle flow of condensing solvent passed through the Soxhlet extractor. (***Note***: *Initially the reaction mixture is a suspension, but slowly becomes a transparent green solution as the reaction progresses*). After 5 h, the mixture was cooled to room temperature and concentrated under reduced pressure. The residue was purified by flash chromatography (silica; toluene/acetonitrile, 12:1 to 7:1) to afford the title compound as a green solid (23.4 mg, 44%). ^{1}H NMR (600 MHz, [D_8]-THF) δ 7.78 (d, *J* = 8.0 Hz, 2H), 7.76 (d, *J* = 8.0 Hz, 2H), 7.59 – 7.54 (m, 4H), 7.33 (d, *J* = 10.2 Hz, 2H), 7.32 (d, *J* = 10.3 Hz, 2H), 6.79 (d, *J* = 2.3 Hz, 1H), 6.78 (d, *J* = 2.3 Hz, 1H), 6.72 (d, *J* = 2.2 Hz, 1H), 6.65 (d, *J* = 2.2 Hz, 1H), 5.15 (s, 1H), 4.50 – 4.43 (m, 4H), 4.07 – 4.01 (m, 4H), 3.75 – 3.68 (m, 4H), 3.31 – 3.17 (m, 4H), 2.44 (s, 3H), 2.43 (s, 3H), 1.93 – 1.86 (m, 8H), 1.74 (s, 3H), 1.12 (td, J = 7.4, 0.8 Hz, 3H), 1.12 (td, *J* = 7.4, 0.8 Hz, 3H), 0.85 (t, *J* = 7.3 Hz, 3H), 0.84 (t, *J* = 7.5 Hz, 3H). ^{13}C NMR (151 MHz, [D_8]-THF) δ 186.2, 186.0, 184.6, 171.8 (d, J = 37.8 Hz), 159.5, 159.3, 158.5, 158.4, 138.7, 138.5, 138.4, 138.2, 138.1, 137.1 (d, *J* = 4.3 Hz), 134.8, 134.5, 133.4, 133.4, 133.3, 133.2, 130.4, 130.2, 130.1, 129.9, 129.4, 127.9, 127.8, 127.8 (d, *J* = 3.7 Hz), 127.5, 127.2, 127.1, 127.0, 111.8 (q, *J* = 287.0 Hz), 77.7 (d, *J* = 2.1 Hz), 77.4, 77.3, 32.1 (dd, *J* = 7.3, 4.4 Hz), 24.4 (d, J = 1.6 Hz), 23.8, 23.7, 23.3, 21.2 (d, *J* = 2.3 Hz), 11.2 (d, *J* = 3.5 Hz), 10.1 (d, *J* = 3.3 Hz). ^{19}F NMR (565 MHz, [D_8]-THF) δ –75.9. IR (ATR): ṽ = 2960, 2925, 2874, 2854, 1641, 1468, 1402, 1384, 1201, 1158, 1109, 1005, 817 cm^{-1}. HRMS (ESI +): *m/z* calcd. for $C_{60}H_{62}F_3NO_{11}Rh_2Na$ [M+Na]$^+$: 1258.22773; found: 1258.22737.

HPLC analysis: 150 mm Chiralcel IB-N3, Ø 4.6 mm i.D., acetonitrile / water = 80:20, v = 1.0 mL / min, λ = 220 nm, t(enantiomer 1) = 7.75 min, t(enantiomer 2) = 8.16 min. The enantiomers were separated by preparative HPLC using following conditions: 250 mm YMC Chiral Art Cellulose-SB, 5µm, Ø 20.0 mm, acetonitrile / water= 80:20, v= 15.0 mL / min, λ = 220 nm with recycling loop (5 cycles).

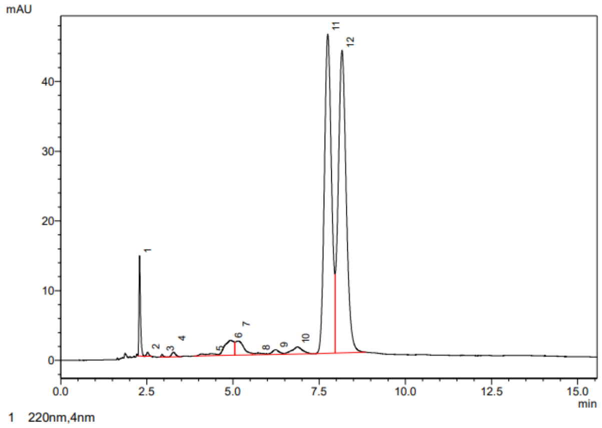

1 220nm,4nm

PDA Ch1 220nm

Peak #	Ret. Time	Area %	Name
1	2.29	2.83	
2	2.53	0.17	
3	2.94	0.09	
4	3.28	0.35	
5	4.38	0.55	
6	4.92	2.67	
7	5.15	2.32	
8	5.72	0.30	
9	6.24	0.67	
10	6.87	1.43	
11	7.75	42.94	
12	8.16	45.68	
Total		100.00	

HPLC trace of the enantiomers of complex ***P*-5**.

Cyclopropanations Using Ethyl 3,3,3-Trifluoro-2-diazopropionate

General Procedure. An oven dried cooling-Schlenk flask equipped with a magnetic stir bar was charged with complex ***P*-1** (0.5 mol%). The respective olefin (5 equiv.) and *n*-pentane (1 mL) were added and the mixture was cooled to –40°C. At this temperature, a solution of ethyl 3,3,3-trifluoro-2-diazopropionate (**3**) (36.4 mg, 0.2 mmol, 1 equiv.) in *n*-pentane (2 mL) was added dropwise over 5 min and the resulting mixture was kept stirring at –40°C for 20-22 h. The mixture was concentrated under reduced pressure and the diastereomeric ratio (dr) of the crude material was determined by ^{19}F NMR. The residue was purified by flash chromatography (silica, *n*-pentane/*tert*-butyl methyl ether). If the diastereomers were separable under these conditions, only the major diastereomer was isolated.

The corresponding racemic compounds needed as references for the ee determinations were prepared accordingly using commercial $Rh_2(esp)_2$ (1 mol%) as the catalyst.

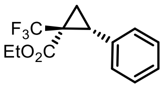

Compound 4a. Prepared according to the general procedure. The crude product was purified by flash chromatography (silica, *n*-pentane/*tert*-butyl methyl ether 40:1), to afford the title compound as a colorless oil (33 mg, 63%, dr = 12:1 (*trans*), 95% *ee*). $[\alpha]_D^{20}$ = –2.6 (c = 1.0, $CHCl_3$). ^{1}H NMR (600 MHz, $CDCl_3$) δ 7.33 – 7.21 (m, 5H), 3.92 – 3.82 (m, 2H), 2.95 (t, *J* = 9.0 Hz, 1H), 2.19 – 2.13 (m, 1H), 1.78 (dd, *J* = 9.7, 5.7 Hz, 1H), 0.88 (t, *J* = 7.1 Hz, 3H). ^{13}C NMR (151 MHz, $CDCl_3$) δ 165.2 (q, *J* = 1.1 Hz), 133.9, 129.3, 128.4, 127.7, 125.5 (q, *J* = 272.6 Hz), 61.6, 34.5 (q, *J* = 33.4 Hz), 29.3 (q, *J* = 1.9 Hz), 15.1 (q, *J* = 2.0 Hz), 13.7. ^{19}F NMR (565 MHz, $CDCl_3$) δ –66.9. IR (ATR): ṽ = 1737, 1395, 1372, 1334, 1315, 1224, 1198, 1146, 1121, 1079, 1026, 732, 697 cm^{-1}. HRMS (EI) *m/z* calcd. for $C_{13}H_{13}O_2F_3$ $[M]^+$: calcd.: 258.08622; found: 258.08632.

The optical purity was determined by HPLC (Chiralcel OJ-3R, 4.6 mm ∅, methanol/water = 80:20, v = 1.0 mL/min, λ = 220 nm): 5.32 min (minor) and 7.45 min (major).

The relative stereochemistry was determined with the aid of ^{1}H-^{1}H NOESY and ^{1}H-^{19}F HOESY spectra (see copies of spectra on pages S65-S66)

The absolute configuration was assigned by comparison of the experimental and the computed CD spectra of the two possible enantiomers of **4a**, see below (page S30); all other compounds with a -CF_3 substituent were assigned by analogy.

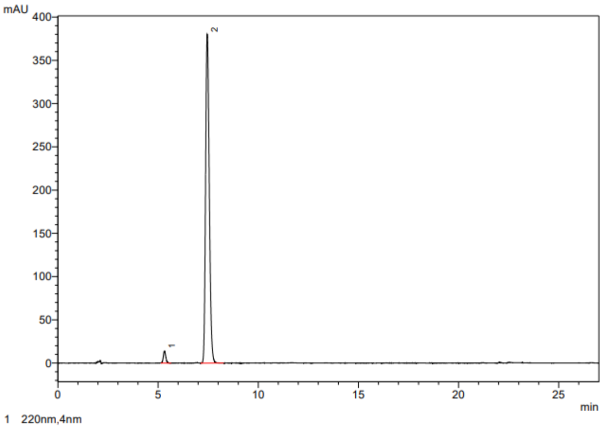

PDA Ch1 220nm

Peak #	Ret. Time	Area %	Name
1	5,32	2,38	1. Enantiomer Diastereomer 1
2	7,45	97,62	2. Enantiomer Diastereomer 1 95.2 % ee
Total		100,00	

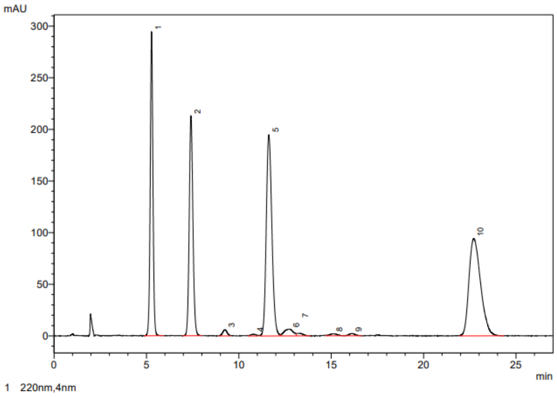

PDA Ch1 220nm

Peak #	Ret. Time	Area %	Name
1	5,28	20,23	1. Enantiomer Diastereomer 1
2	7,41	20,08	2. Enantiomer Diastereomer 1
3	9,24	0,69	
4	10,80	0,24	
5	11,62	28,03	1. Enantiomer Diastereomer 2
6	12,74	1,53	
7	13,23	0,40	
8	15,09	0,41	
9	16,12	0,36	
10	22,71	28,04	2. Enantiomer Diastereomer 2
Total		100,00	

HPLC traces of **4a** (left) and the corresponding racemate (right).

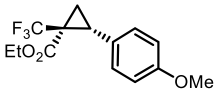

Compound 4b. Prepared according to the general procedure. The crude product was purified by flash chromatography (silica, *n*-pentane/*tert*-butyl methyl ether 25:1 to 12:1), to afford the title compound as a colorless oil (40.5 mg, 71% (contains the *cis*-isomer, dr = 6:1, 92% *ee* (*trans*)). $[\alpha]_D^{20}$ = +8.7 (c = 1.1, $CHCl_3$). ^1H NMR (400 MHz, $CDCl_3$) δ 7.20 – 7.13 (m, 2H), 6.85 – 6.78 (m, 2H), 3.91 (qd, *J* = 7.1, 1.7 Hz, 2H), 3.78 (s, 3H), 2.89 (t, *J* = 8.9 Hz, 1H), 2.12 (ddq, *J* = 9.5, 5.7, 1.9 Hz, 1H), 1.75 (dd, *J* = 9.7, 5.7 Hz, 1H), 0.94 (t, *J* = 7.1 Hz, 3H). ^{13}C NMR (101 MHz, $CDCl_3$) δ 165.3, 159.2, 130.3, 125.8, 124.5 (q, *J* = 274.0 Hz), 113.7, 61.6, 55.4, 34.5 (q, *J* = 33.3 Hz), 28.7 (q, *J* = 1.9 Hz), 15.2 (q, *J* = 2.2 Hz), 13.8. ^{19}F NMR (282 MHz, $CDCl_3$) δ –61.1 (minor diastereomer), –66.7 (major diastereomer). IR (ATR): ṽ =1733, 1614, 1517, 1391, 1372, 1332, 1316, 1295, 1248, 1225, 1197, 1142, 1113, 1077, 1032, 837, 809 cm^{-1}. HRMS (EI) *m/z* calcd. for $C_{14}H_{15}O_3F_3$ $[M]^+$: calcd.: 288.09678; found: 288.09710.

The optical purity was determined by HPLC (Chiralcel OJ-3, 4.6 mm ∅, *n*-heptane/ethanol = 98:2, v = 1.0 mL/min, λ = 230 nm): 4.49 min (minor) and 5.21 min (major).

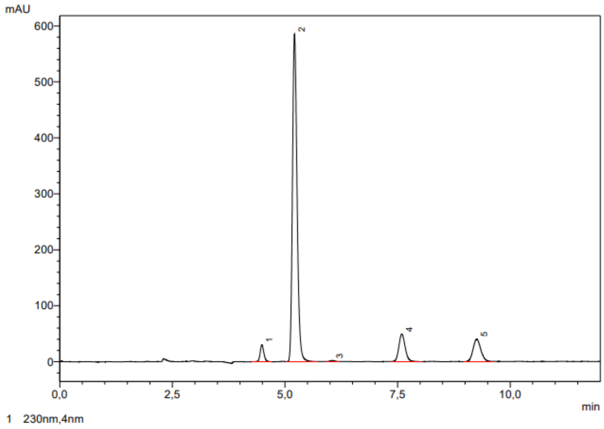

PDA Ch1 230nm

Peak #	Ret. Time	Area %	Name
1	4,49	3,26	1. Enantiomer Diastereomer 1
2	5,21	78,12	2. Enantiomer Diastereomer 1 92.0 % ee
3	6,05	0,34	
4	7,59	9,14	1. Enantiomer Diastereomer 2 racemisch
5	9,26	9,15	2. Enantiomer Diastereomer 2
Total		100,00	

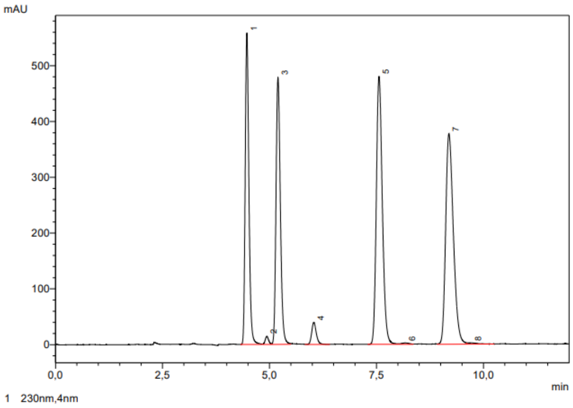

PDA Ch1 230nm

Peak #	Ret. Time	Area %	Name
1	4,47	20,20	1. Enantiomer Diastereomer 1
2	4,94	0,48	
3	5,20	20,31	2. Enantiomer Diastereomer 1
4	6,04	1,80	
5	7,56	28,31	1. Enantiomer Diastereomer 2
6	8,17	0,19	
7	9,19	28,47	2. Enantiomer Diastereomer 2
8	9,71	0,24	
Total		100,00	

HPLC traces of **4b** (left) and the corresponding racemate (right).

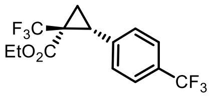

Compound 4c. Prepared according to the general procedure. The crude product was purified by flash chromatography (silica, *n*-pentane/*tert*-butyl methyl ether 40:1), to afford the title compound as a colorless oil (33 mg, 51%, dr = 10:1, 96% *ee* (*trans*)). $[\alpha]_D^{20}$ = +1.7 (c = 1.0, $CHCl_3$). ^1H NMR (600 MHz, $CDCl_3$) δ 7.58 – 7.51 (m, 2H), 7.41 – 7.34 (m, 2H), 3.93 (dd, *J* = 7.1, 0.4 Hz, 1H), 3.90 (d, *J* = 7.1 Hz, 1H), 2.97 (t, *J* = 9.0 Hz, 1H), 2.18 (ddq, *J* = 7.7, 5.7, 1.9 Hz, 1H), 1.84 (dd, *J* = 9.7, 5.8 Hz, 1H), 0.91 (t, *J* = 7.1 Hz, 3H). ^{13}C NMR (151 MHz, $CDCl_3$) δ 164.9, 138.1 (q, *J* = 1.3 Hz), 130.1 (q, *J* = 32.6 Hz), 125.3 (q, *J* = 3.8 Hz), 124.3 (q, *J* = 273.5 Hz), 124.1 (q, *J* = 272.0 Hz), 61.9, 34.6 (q, *J* = 33.9 Hz), 28.8 (q, *J* = 1.9 Hz), 15.3 (q, *J* = 2.1 Hz), 13.7. ^{19}F NMR (565 MHz, $CDCl_3$) δ –62.7, –67.0. IR (ATR): ṽ = 1736, 1393, 1373, 1323, 1225, 1152, 1109, 1063, 1019, 848, 603 cm^{-1}. HRMS (EI) *m/z* calcd. for $C_{14}H_{12}O_2F_6$ $[M]^+$: calcd.: 326.07360; found: 326.07352.

The optical purity was determined by HPLC (Chiralcel OJ-3R, 4.6 mm ∅, acetonitrile/water = 40:60, v = 1.0 mL/min, λ = 220 nm): 34.32 min (minor) and 36.34 min (major).

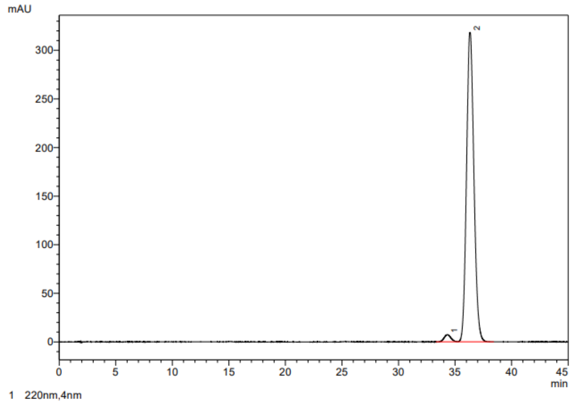

PDA Ch1 220nm

Peak #	Ret. Time	Area %	Name
1	34,32	2,11	1. Enantiomer
2	36,34	97,89	2. Enantiomer 95.8 % ee
Total		100,00	

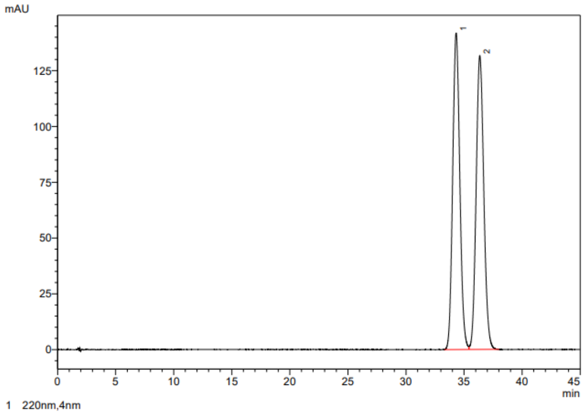

PDA Ch1 220nm

Peak #	Ret. Time	Area %	Name
1	34,32	49,99	1. Enantiomer
2	36,36	50,01	2. Enantiomer
Total		100,00	

HPLC traces of **4c** (left) and the corresponding racemate (right).

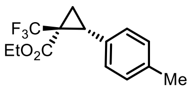

Compound 4d. Prepared according to the general procedure. The crude product was purified by flash chromatography (silica, *n*-pentane/*tert*-butyl methyl ether 99:1) which afforded the desired product as a colorless oil (41 mg, 73%, d.r. = 6:1 (*trans*), 90% *ee*). $[\alpha]_D^{20}$ = +13.6 (c = 0.5, $CHCl_3$). ^{1}H NMR (400 MHz, $CDCl_3$) δ 7.14 – 7.07 (m, 4H), 3.90 (qd, *J* = 7.1, 0.7 Hz, 2H), 2.91 (t, *J* = 9.0 Hz, 1H), 2.31 (s, 3H), 2.13 (ddq, *J* = 7.7, 5.7, 2.0 Hz, 1H), 1.75 (dd, *J* = 9.7, 5.6 Hz, 1H), 0.92 (t, *J* = 7.1 Hz, 3H). ^{13}C NMR (101 MHz, $CDCl_3$) δ 165.3, 137.4, 130.8, 129.1, 129.0, 124.6 (q, J = 273.6 Hz), 61.6, 34.5 (q, J = 33.4 Hz), 29.1 (q, J = 2.0 Hz), 21.3, 15.1 (q, J = 2.0 Hz), 13.8. ^{19}F NMR (376 MHz, $CDCl_3$) δ –61.1 (minor diastereomer), –66.7 (major diastereomer). IR (ATR): ṽ = 1736, 1389, 1372, 1331, 1314, 1224, 1195, 1143, 1114, 1076, 1027, 828 cm^{-1}. HRMS (EI) *m/z* calcd. for $C_{14}H_{15}O_2F_3$ $[M]^+$: calcd: 272.10194; found: 272.10187.

The optical purity was determined by HPLC (Chiralcel OJ-3R, 4.6 mm ∅, acetonitrile/water= 60:40, v = 1.0 mL/min, λ = 220 nm): 5.81 min (minor) and 6.53 min (major).

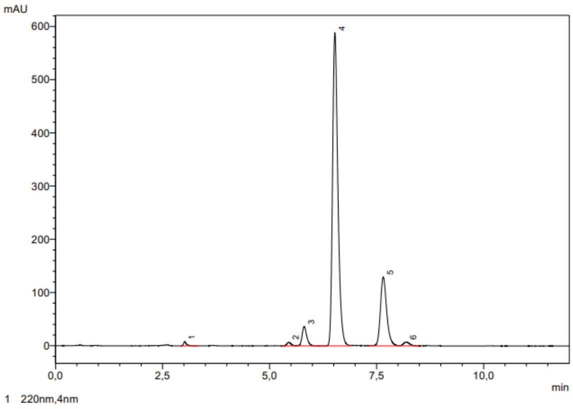

PDA Ch1 220nm

Peak #	Ret. Time	Area %	Name
1	3,02	0,57	
2	5,45	0,73	
3	5,81	4,03	1. Enantiomer Diastereomer 1
4	6,53	74,23	2. Enantiomer Diastereomer 1 89.7 % ee
5	7,66	19,36	1. u. 2. Enantiomer Diastereomer 2
6	8,20	1,09	
Total		100,00	

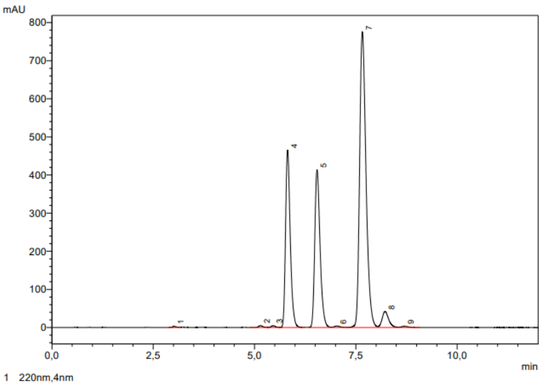

PDA Ch1 220nm

Peak #	Ret. Time	Area %	Name
1	3,02	0,11	
2	5,15	0,22	
3	5,46	0,21	
4	5,82	22,52	1. Enantiomer Diastereomer 1
5	6,54	22,58	2. Enantiomer Diastereomer 1
6	7,03	0,25	
7	7,66	51,02	1. u. 2. Enantiomer Diastereomer 2
8	8,22	2,86	
9	8,70	0,23	
Total		100,00	

HPLC traces of **4d** (left) and the corresponding racemate (right).

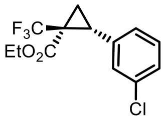

Compound 4e. Prepared according to the general procedure. The crude product was purified by flash chromatography (silica, *n*-pentane/*tert*-butyl methyl ether 40:1), to afford the title compound as a colorless oil (40.4 mg, 67%, dr = 10:1, 96% *ee* (*trans*)). $[\alpha]_D^{20}$ = +7.5 (c = 1.2, $CHCl_3$). ^{1}H NMR (400 MHz, $CDCl_3$) δ 7.28 – 7.19 (m, 3H), 7.17 – 7.11 (m, 1H), 3.93 (qd, *J* = 7.1, 2.2 Hz, 2H), 2.91 (t, *J* = 8.9 Hz, 1H), 2.13 (ddq, *J* = 7.7, 5.7, 1.9 Hz, 1H), 1.79 (dd, *J* = 9.7, 5.8 Hz, 1H), 0.95 (t, *J* = 7.1 Hz, 3H). ^{13}C NMR (101 MHz, $CDCl_3$) δ 164.9, 136.0, 134.3, 129.6, 128.0, 127.5, 124.4 (q, *J* = 274.9 Hz), 61.8, 34.5 (q, *J* = 33.7 Hz), 28.7 (q, *J* = 2.2 Hz), 15.1 (q, *J* = 2.1 Hz), 13.8. ^{19}F NMR (282 MHz, $CDCl_3$) δ –67.0. IR (ATR): ṽ = 1735, 1390, 1372, 1334, 1314, 1223, 1196, 1145, 1120, 1076, 1027, 788, 692, 678 cm^{-1}. HRMS (EI) *m/z* calcd. for $C_{13}H_{12}O_2F_3Cl$ $[M]^+$: calcd.: 292.04724; found: 292.04721.

The optical purity was determined by HPLC (Chiralcel OJ-3R, 4.6 mm ∅, acetonitrile/water = 50:50, v = 1.0 mL/min, λ = 220 nm): 9.83 min (minor) and 11.13 min (major).

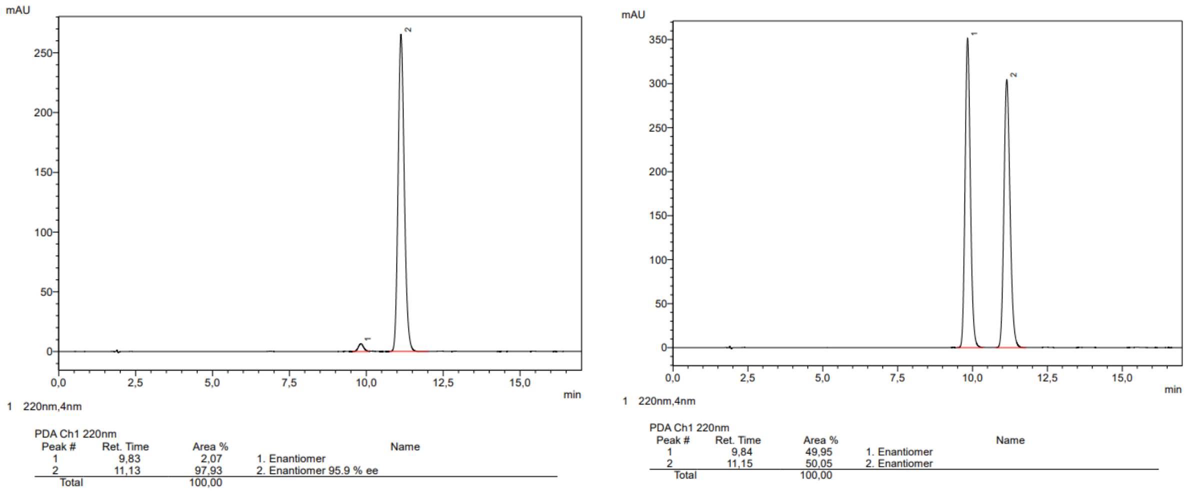

HPLC traces of **4e** (left) and the corresponding racemate (right).

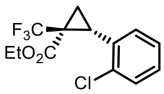

Compound 4f. Prepared according to the general procedure. The crude product was purified by flash chromatography (silica, *n*-pentane/*tert*-butyl methyl ether 40:1 to 30:1), to afford the title compound as a colorless oil (37 mg, 63%, dr = 7:1, 93% *ee* (*trans*)). $[\alpha]_D^{20}$ = –61.5 (c = 1.1, $CHCl_3$). ^{1}H NMR (400 MHz, $CDCl_3$) δ 7.38 – 7.32 (m, 1H), 7.28 – 7.20 (m, 3H), 3.95 (q, *J* = 7.1 Hz, 2H), 2.99 (t, *J* = 9.0 Hz, 1H), 2.13 (ddq, *J* = 8.6, 5.6, 1.5 Hz, 1H), 1.84 (dd, *J* = 9.6, 5.7 Hz, 1H), 0.94 (t, *J* = 7.1 Hz, 3H). ^{13}C NMR (101 MHz, $CDCl_3$) δ 165.4, 135.9, 132.3, 130.9, 129.3, 129.1, 126.5, 124.4 (q, *J* = 273.7 Hz), 61.7, 34.1 (q, *J* = 33.5 Hz), 28.4 (q, *J* = 2.3 Hz), 15.8 (q, *J* = 2.1 Hz), 13.7. ^{19}F NMR (282 MHz, $CDCl_3$) δ –66.8. IR (ATR): ṽ = 1734, 1390, 1372, 1335, 1314, 1221, 1142, 1116, 1053, 1025, 769, 747, 686 cm^{-1}. HRMS (EI) *m/z* calcd. for $C_{13}H_{12}O_2F_3Cl$ $[M]^+$: calcd.: 292.04724; found: 292.04721.

The optical purity was determined by 2D-HPLC: 1st dimension: (50 mm Eclipse Plus C18, 4.6 mm ∅, acetonitrile/water gradient 60:40 to 90:10 in 5 min, 1.0 mL/min, 308 K, λ = 220 nm): 2.71-2.73 min; 2nd dimension: (Chiralpak OJ-3R, 4.6 mm ∅, methanol/water = 80:20, v = 1.0 mL/min, λ = 220 nm): 4.65 min (minor) and 6.83 min (major).

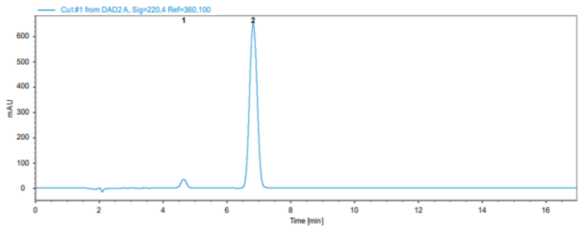

Signal: DAD2 A, Sig=220,4 Ref=360,100

Compound	Cut	Ret.Time	Area	Width	Height	Symmetry
1	1	4.651	422.162 **422.162**	0.192	33.937	1.050
2	1	6.832	10863.525 **10863.525**	0.267	650.363	0.938

= 92.5 % ee

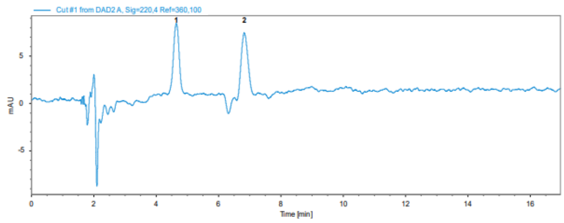

Signal: DAD2 A, Sig=220,4 Ref=360,100

Compound	Cut	Ret.Time	Area	Width	Height	Symmetry
1	1	4.652	93.950 **93.950**	0.210	7.459	1.016
2	1	6.833	97.357 **97.357**	0.243	6.678	0.794

HPLC traces of **4f** (left) and the corresponding racemate (right).

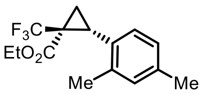

Compound 4g. Prepared according to the general procedure. The crude product was purified by flash chromatography (silica, *n*-pentane/*tert*-butyl methyl ether 40:1), to afford the title compound as a colorless oil (34 mg, 59%, dr = 9:1, 96% *ee* (*trans*)). $[\alpha]_D^{20}$ = –28.1 (c = 0.9, $CHCl_3$). ^{1}H NMR (400 MHz, $CDCl_3$) δ 7.07 – 7.00 (m, 1H), 6.97– 6.93 (m, 2H), 3.90 (q, *J* = 7.1 Hz, 2H), 2.82 (t, *J* = 8.9 Hz, 1H), 2.32 (s, 3H), 2.29 (s, 3H), 2.18 (ddt, *J* = 7.3, 3.7, 1.7 Hz, 1H), 1.78 (dd, *J* = 9.6, 5.6 Hz, 1H), 0.90 (t, *J* = 7.1 Hz, 3H). ^{13}C NMR (101 MHz, $CDCl_3$) δ 165.4, 138.3, 137.5, 130.9, 129.0, 126.3, 124.7 (q, *J* = 273.4 Hz), 61.5, 34.3 (q, *J* = 33.2 Hz), 28.2 (q, *J* = 2.2 Hz), 21.1, 19.2, 15.1 (q, *J* = 2.3 Hz), 13.7. ^{19}F NMR (282 MHz, $CDCl_3$) δ –66.4.IR (ATR): ṽ = 1736, 1389, 1372, 1334, 13114, 1252, 1218, 1198, 1149, 1111, 1076, 1028, 829 cm^{-1}. HRMS (EI) *m/z* calcd. for $C_{15}H_{17}O_2F_3$ $[M]^+$: calcd.: 286.11752; found: 286.11773.

The optical purity was determined by HPLC (Chiralcel OJ-3R, 4.6 mm ∅, acetonitrile/water = 80:20, v = 1.0 mL/min, λ = 220 nm): 8.75 min (minor) and 13.04 min (major).

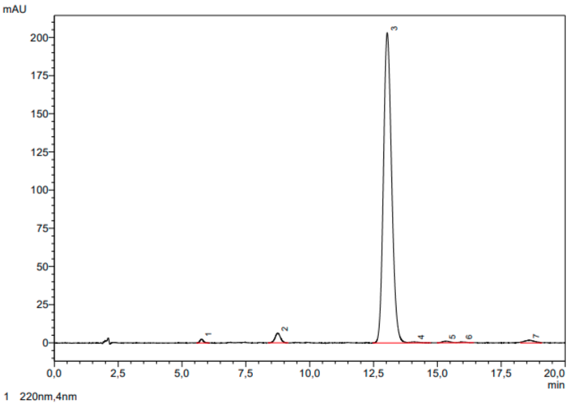

PDA Ch1 220nm

Peak #	Ret. Time	Area %	Name
1	5,77	0,50	
2	8,75	2,01	1. Enantiomer
3	13,04	95,33	2. Enantiomer 95.9 % ee
4	14,09	0,34	
5	15,32	0,49	
6	15,96	0,23	
7	18,59	1,10	
Total		100,00	

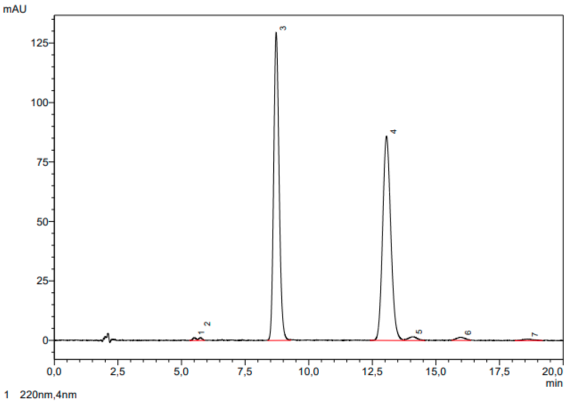

PDA Ch1 220nm

Peak #	Ret. Time	Area %	Name
1	5,50	0,28	
2	5,74	0,29	
3	8,72	48,54	1. Enantiomer
4	13,06	48,56	2. Enantiomer
5	14,06	1,00	
6	15,99	0,87	
7	18,62	0,47	
Total		100,00	

HPLC traces of **4g** (left) and the corresponding racemate (right).

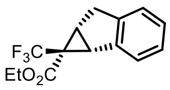

Compound 4h. Prepared according to the general procedure. The crude product was purified by flash chromatography (silica, *n*-pentane/*tert*-butyl methyl ether 40:1) to afford the title compound as a colorless oil (43 mg, 80% (contains an unknown impurity), dr > 50:1, 97% *ee* (*trans*)). An analytically pure sample was obtained after preparatory HPLC separation. $[\alpha]_D^{20}$ = +36.8 (c = 0.4, $CHCl_3$). ^1H NMR (600 MHz, $CDCl_3$) δ 7.41 – 7.36 (m, 1H), 7.20 – 7.12 (m, 3H), 3.84 – 3.72 (m, 2H), 3.39 (d, *J* = 17.5 Hz, 1H), 3.29 (dd, *J* = 17.5, 6.7 Hz, 1H), 3.15 (dd, *J* = 6.8, 1.3 Hz, 1H), 2.54 (td, *J* = 6.8, 0.9 Hz, 1H), 0.77 (t, *J* = 7.1 Hz, 3H). ^{13}C NMR (151 MHz, $CDCl_3$) δ 163.6, 142.2, 139.4, 127.5, 126.9, 125.7, 125.0, 124.0 (q, *J* = 275.6 Hz), 61.6, 38.0 (q, *J* = 32.7 Hz), 33.6 (q, *J* = 2.3 Hz), 33.1, 25.4 (q, *J* = 2.6 Hz), 13.6. ^{19}F NMR (565 MHz, $CDCl_3$) δ –66.4. IR (ATR): ṽ = 1735, 1372, 1327, 1314, 1287, 1229, 1215, 1155, 1077, 1042, 1024, 750, 725 cm^{-1}. HRMS (EI) *m/z* calcd. for $C_{14}H_{13}O_2F_3$ $[M]^+$: calcd.: 270.08622; found: 270.08621.

The optical purity was determined by HPLC (Chiralcel OJ-3R, 4.6 mm ∅, methanol/water = 80:20, v = 1.0 mL/min, λ = 220 nm): 6.16 min (minor) and 7.38 min (major).

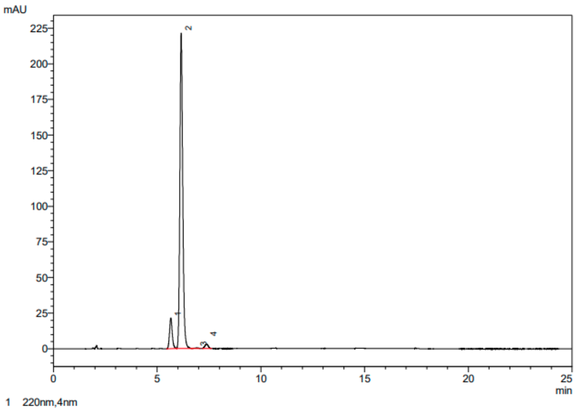

PDA Ch1 220nm

Peak #	Ret. Time	Area %	Name
1	5.66	8.22	
2	6.16	90.19	1st enantiomer
3	6.91	0.14	
4	7.38	1.46	2nd enantiomer
Total		100.00	

= 96.8 % ee

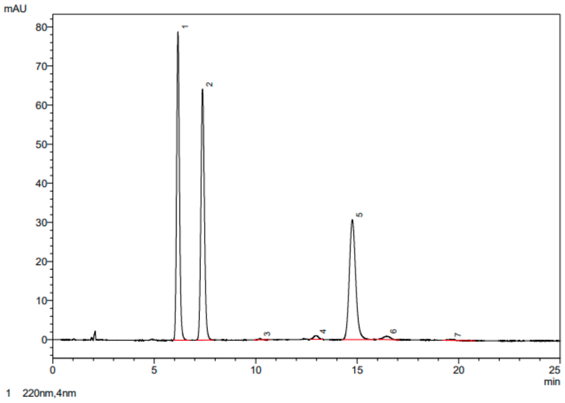

PDA Ch1 220nm

Peak #	Ret. Time	Area %	Name
1	6.17	34.16	1st enantiomer
2	7.38	34.10	2nd enantiomer
3	10.22	0.15	
4	12.97	0.75	
5	14.76	29.73	
6	16.43	0.96	
7	19.63	0.15	
Total		100.00	

HPLC traces of **4h** (left) and the corresponding racemate (right).

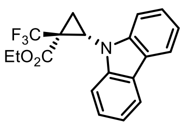

Compound 4i. Prepared according to the general procedure. The crude product was purified by flash chromatography (silica, toluene) to afford the title compound as a colorless oil (25.1 mg, 48%, d.r. = 6.5:1 (*trans*), 94% *ee*). $[\alpha]_D^{20}$ = +5.9 (c = 1.0, $CHCl_3$). ^1H NMR (600 MHz, [D_6]-DMSO, 383 K) δ 8.11 (dt, *J* = 7.8, 0.9 Hz, 2H), 7.59 (d, *J* = 8.2 Hz, 2H), 7.47 (ddd, *J* = 8.3, 7.2, 1.2 Hz, 2H), 7.25 (ddd, *J* = 7.9, 7.2, 0.9 Hz, 2H), 4.45 (dd, *J* = 8.5, 6.6 Hz, 1H), 3.60 – 3.46 (m, 2H), 2.71 (tq, *J* = 6.7, 1.6 Hz, 1H), 2.53 (dd, *J* = 8.5, 6.8 Hz, 1H), 0.50 (t, *J* = 7.1 Hz, 3H). ^{13}C NMR (151 MHz, [D_6]-DMSO, 383 K) δ 162.7, 140.0, 125.3, 123.4 (q, *J* = 273.9 Hz), 122.4, 119.6, 119.3, 109.1, 60.9, 35.4 (q, *J* = 2.8 Hz), 33.1 (q, *J* = 32.5 Hz), 17.7 (q, *J* = 1.8 Hz), 11.9. ^{19}F NMR (565 MHz, [D_6]-DMSO, 383 K) δ –64.6. IR (ATR): ṽ = 1736, 1455, 1402, 1321, 1233, 1207, 1155, 1140 ,1120, 1093, 749, 725 cm^{-1}. HRMS (EI) *m/z* calcd. for $C_{19}H_{16}F_3NO_2Na$ $[M+Na]^+$: calcd: 370.10262; found: 370.10253.

The optical purity was determined by HPLC (Chiralcel OJ-3R, 4.6 mm ∅, acetonitrile/water= 60:40, v= 1.0 mL/min, λ =235 nm): 7.60 min (minor) and 9.34 min (major).

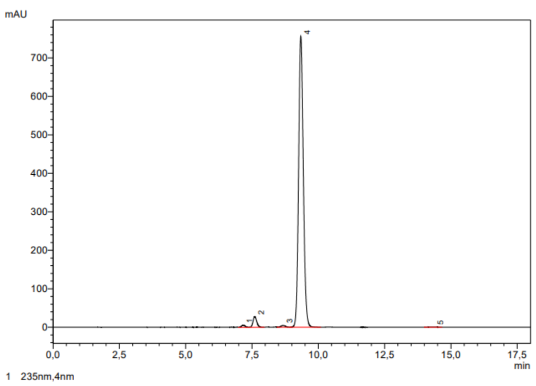

PDA Ch1 235nm

Peak #	Ret. Time	Area %	Name
1	7,17	0,53	
2	7,60	2,81	1. Enantiomer
3	8,68	0,57	
4	9,34	95,98	2. Enantiomer 94.3 % ee
5	14,36	0,10	
Total		100,00	

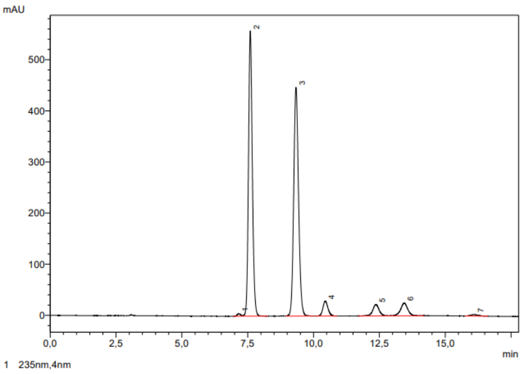

PDA Ch1 235nm

Peak #	Ret. Time	Area %	Name
1	7,16	0,36	
2	7,60	44,60	1. Enantiomer
3	9,33	44,74	2. Enantiomer
4	10,44	3,11	
5	12,37	3,02	
6	13,44	3,72	
7	16,11	0,45	
Total		100,00	

HPLC traces of **4i** (left) and the corresponding racemate (right).

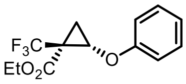

Compound 4j. Prepared according to the general procedure. The crude product was purified by flash chromatography (silica, *n*-pentane/*tert*-butyl methyl ether 40:1), to afford the title compound as a colorless oil (32 mg, 59%, dr = 5:1, 92% *ee* (*trans*)). $[\alpha]_D^{20}$ = –150.3 (c = 1.1, $CHCl_3$). ^1H NMR (400 MHz, $CDCl_3$) δ 7.33 – 7.27 (m, 2H), 7.07 – 6.97 (m, 3H), 4.25 (dd, *J* = 7.4, 5.3 Hz, 1H), 4.09 (q, *J* = 7.1 Hz, 2H), 2.27 (tt, *J* = 5.4, 1.7 Hz, 1H), 1.80 (t, *J* = 7.3 Hz, 1H), 1.04 (t, *J* = 7.1 Hz, 3H). ^{13}C NMR (101 MHz, $CDCl_3$) δ 163.6, 157.3, 129.7, 123.8 (q, *J* = 272.3 Hz), 122.5, 115.0, 62.2, 57.7 (q, *J* = 2.5 Hz), 34.0 (q, *J* = 33.2 Hz), 17.2 (q, *J* = 2.2 Hz), 13.9. ^{19}F NMR (282 MHz, $CDCl_3$) δ –65.3. IR (ATR): ṽ = 1739, 1591, 1494, 1383, 1372, 1319, 1249, 1202, 1140, 1100, 1080, 1026, 753, 691 cm^{-1}. HRMS (EI) *m/z* calcd. for $C_{13}H_{13}O_3F_3$ $[M]^+$: calcd.: 274.08113; found: 274.08121.

The optical purity was determined by HPLC (Chiralcel OJ-3R, 4.6 mm ∅, acetonitrile/water = 50:50, v = 1.0 mL/min, λ = 220 nm): 7.41 min (minor) and 8.64 min (major).

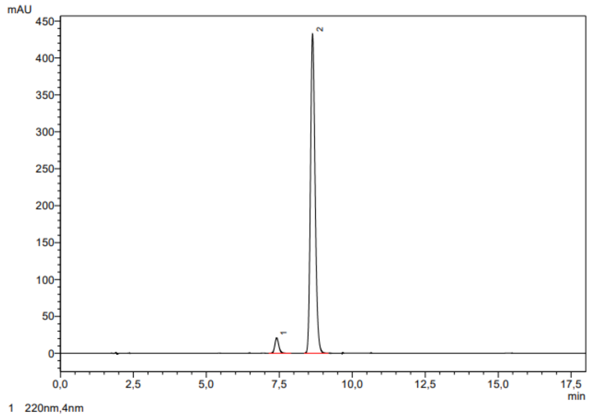

1 220nm,4nm

PDA Ch1 220nm

Peak #	Ret. Time	Area %	Name
1	7,41	3,87	1. Enantiomer
2	8,64	96,13	2. Enantiomer 92.3 % ee
Total		100,00	

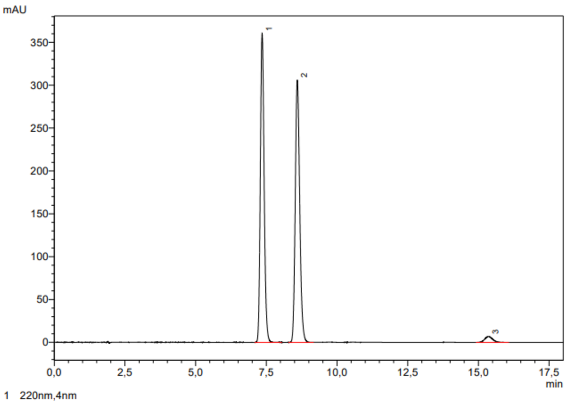

1 220nm,4nm

PDA Ch1 220nm

Peak #	Ret. Time	Area %	Name
1	7,35	48,98	1. Enantiomer
2	8,60	49,07	2. Enantiomer
3	15,36	1,95	
Total		100,00	

HPLC traces of **4j** (left) and the corresponding racemate (right).

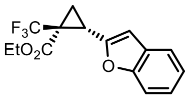

Compound 4k. Prepared according to the general procedure. The crude product was purified by flash chromatography (silica, *iso*-hexane/EtOAc 99:01) to afford the title compound as a colorless oil (26.7 mg, 60%, d.r. = 4.5:1 (*trans*), 79% *ee*). $[\alpha]_D^{20}$ = –1.6 (c = 0.4, $CHCl_3$). ^{1}H NMR (400 MHz, $CDCl_3$) δ 7.50 (ddd, *J* = 7.5, 1.5, 0.7 Hz, 1H), 7.41 (dd, *J* = 8.1, 1.0 Hz, 1H), 7.28 – 7.19 (m, 2H), 6.59 (t, *J* = 1.0 Hz, 1H), 4.00 (qd, *J* = 7.1, 1.7 Hz, 2H), 2.99 – 2.90 (m, 1H), 2.22 (ddq, *J* = 7.7, 5.6, 1.8 Hz, 1H), 1.90 (dd, *J* = 9.8, 5.7 Hz, 1H), 0.96 (t, *J* = 7.1 Hz, 3H). ^{13}C NMR (101 MHz, $CDCl_3$) δ 164.6, 154.9, 151.4, 128.2, 124.4, 124.0 (q, *J* = 273.9 Hz), 123.0, 121.0, 111.1, 105.9, 62.1, 34.5 (q, *J* = 33.9 Hz), 22.1 (q, *J* = 2.4 Hz), 15.3 (q, *J* = 2.3 Hz), 13.8. ^{19}F NMR (376 MHz, $CDCl_3$) δ –62.4 (minor diastereomer), –67.2 (major diastereomer). IR (ATR): ṽ = 1738, 1454, 1400, 1373, 1319, 1209, 1163, 1116, 1106, 1078, 751 cm^{-1}. HRMS (EI) *m/z* calcd. for $C_{15}H_{13}O_3F_3$ $[M]^+$: calcd: 298.08119; found: 298.08113.

The optical purity was determined by HPLC (Chiralcel OJ-3R, 4.6 mm ∅, acetonitrile/water= 55:45, v= 1.0 mL/min, λ =250 nm): 9.40 min (major) and 10.36 min (minor).

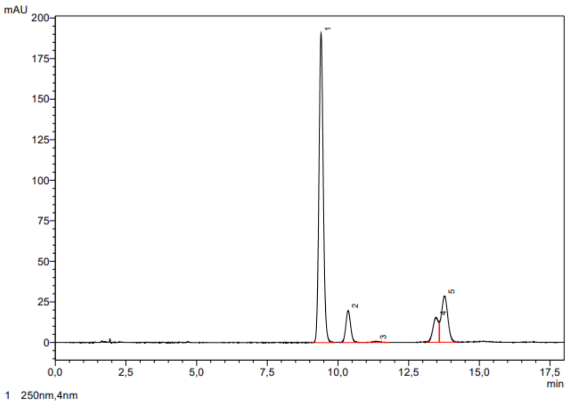

PDA Ch1 250nm

Peak #	Ret. Time	Area %	Name
1	9,40	68,79	1. Enantiomer 79.4 % ee
2	10,36	7,89	2. Enantiomer
3	11,35	0,44	
4	13,47	7,29	
5	13,77	15,59	
Total		100,00	

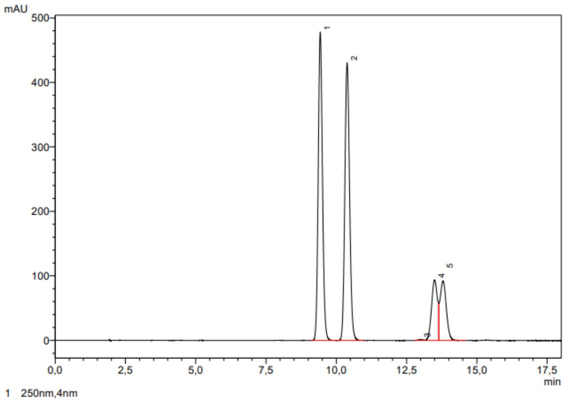

PDA Ch1 250nm

Peak #	Ret. Time	Area %	Name
1	9,43	38,95	1. Enantiomer
2	10,38	38,99	2. Enantiomer
3	13,00	0,17	
4	13,50	10,73	
5	13,80	11,16	
Total		100,00	

HPLC traces of **4k** (left) and the corresponding racemate (right).

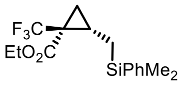

Compound 4l. Prepared according to the general procedure. The crude product was purified by flash chromatography (silica, *n*-pentane/*tert*-butyl methyl ether 40:1), to afford the title compound as a colorless oil (34 mg, 51%, dr = 2:1, 68% *ee* (*trans*)). $[\alpha]_D^{20}$ = +7.0 (c = 0.3, $CHCl_3$). ^1H NMR (400 MHz, $CDCl_3$) δ 7.53 – 7.48 (m, 2H), 7.41 – 7.34 (m, 3H), 4.33 – 4.12 (m, 2H), 1.62 (tdd, *J* = 9.2, 8.1, 5.7 Hz, 1H), 1.42 (dd, *J* = 9.5, 5.2 Hz, 1H), 1.30 (t, *J* = 7.2 Hz, 4H), 1.09 (dd, *J* = 14.8, 5.7 Hz, 1H), 1.00 – 0.90 (m, 1H), 0.33 (d, *J* = 2.0 Hz, 6H). ^{13}C NMR (101 MHz, $CDCl_3$) δ 167.0, 138.1, 133.7, 129.4, 128.0, 124.8 (q, *J* = 272.4 Hz), 61.8, 32.5 (q, *J* = 33.4 Hz), 22.5 (d, *J* = 1.8 Hz), 18.7 (q, *J* = 2.1 Hz), 14.3, 13.0, –3.1 (d, *J* = 6.5 Hz). ^{19}F NMR (282 MHz, $CDCl_3$) δ –66.2. IR (ATR): ṽ = 1731, 1371, 1321, 1213, 1146, 1126, 1114, 1094, 832, 806, 732, 698 cm^{-1}. HRMS (ESI +) *m/z* calcd. for $C_{16}H_{21}O_2F_3SiNa$ $[M+Na]^+$: calcd.: 353.11551; found: 353.11553.

The optical purity was determined by HPLC (Chiralcel OJ-3R, 4.6 mm ∅, methanol/water = 80:20, v = 1.0 mL/min, λ = 220 nm): 16.23 min (major) and 18.60 min (minor).

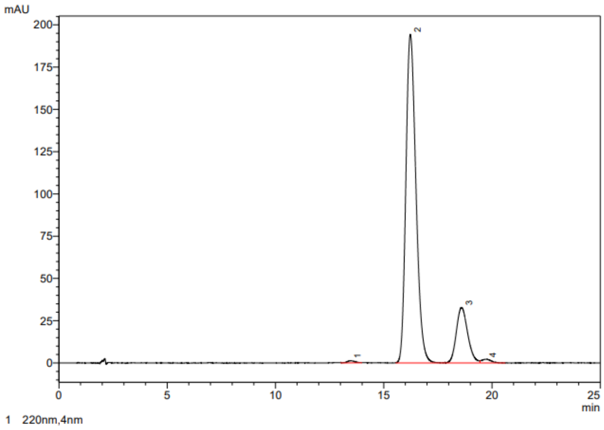

PDA Ch1 220nm

Peak #	Ret. Time	Area %	Name
1	13,45	0,46	
2	16,23	82,80	1. Enantiomer 67.9 % ee
3	18,60	15,85	2. Enantiomer
4	19,70	0,90	
Total		100,00	

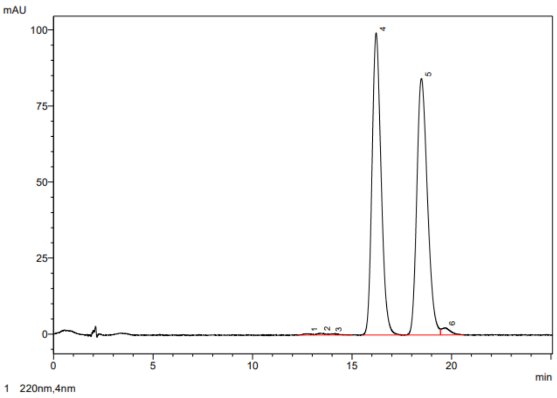

PDA Ch1 220nm

Peak #	Ret. Time	Area %	Name
1	12,78	0,21	
2	13,41	0,25	
3	13,99	0,20	
4	16,21	49,17	1. Enantiomer
5	18,49	49,01	2. Enantiomer
6	19,69	1,16	
Total		100,00	

HPLC traces of **4l** (left) and the corresponding racemate (right).

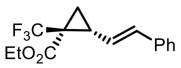

Compound 4m. Prepared according to the general procedure. The crude product was purified by flash chromatography (silica, *n*-pentane/*tert*-butyl methyl ether 40:1), to afford the title compound as a colorless oil (34 mg, 59%, dr = 5:1, 88% *ee* (*trans*)). $[\alpha]_D^{20}$ = +29.5 (c = 2.2, $CHCl_3$). ^{1}H NMR (400 MHz, $CDCl_3$) δ 7.36 – 7.28 (m, 5H), 7.26 – 7.20 (m, 1H), 6.68 (d, *J* = 15.9 Hz, 1H), 6.07 (dd, *J* = 15.9, 8.8 Hz, 1H), 4.54 – 4.09 (m, 2H), 2.47 (q, *J* = 8.7 Hz, 1H), 1.84 (tt, *J* = 5.7, 1.9 Hz, 1H), 1.72 (dd, *J* = 9.5, 5.6 Hz, 1H), 1.29 (t, *J* = 7.1 Hz, 3H). ^{13}C NMR (101 MHz, $CDCl_3$) δ 166.3, 136.7, 134.6, 128.8, 127.9, 126.3, 125.7, 123.5, 62.1, 34.1 (q, *J* = 33.7 Hz), 28.4 (q, *J* = 2.1 Hz), 18.0 (q, *J* = 2.3 Hz), 14.3. ^{19}F NMR (282 MHz, $CDCl_3$) δ –66.7. IR (ATR): ṽ = 1731, 1394, 1372, 1323, 1313, 1218, 1203, 1152, 1137, 1111, 1048, 1022, 964, 761, 740, 692 cm^{-1}. HRMS (EI) *m/z* calcd. for $C_{15}H_{15}O_2F_3$ $[M]^+$: calcd.: 284.10187; found: 284.10205.

The optical purity was determined by HPLC (Chiralcel OJ-3R, 4.6 mm ∅, acetonitrile/water = 55:45, v = 1.0 mL/min, λ = 220 nm): 11.52 min (minor) and 13.69 min (major).

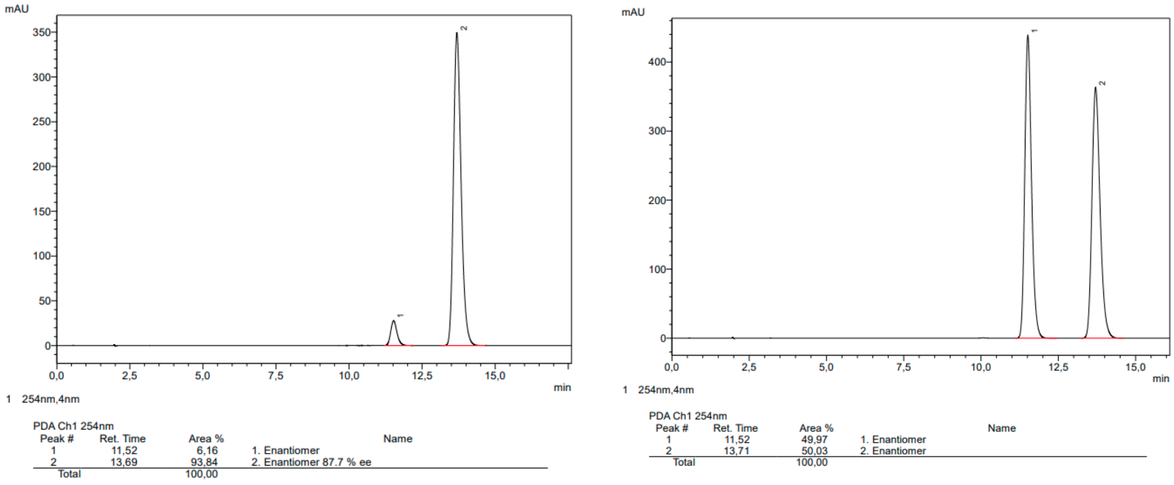

HPLC traces of **4m** (left) and the corresponding racemate (right).

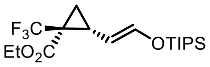

Compound 4n. Prepared according to the general procedure. The crude product was purified by flash chromatography (silica, *n*-pentane/*tert*-butyl methyl ether 100:1 to 40:1), to afford the title compound as a colorless oil (52 mg, 68%, dr = 4:1 (contains the *cis*-diastereomer), 80% *ee* (*trans*)). $[\alpha]_D^{20}$ = –38.1 (c = 1.3, $CHCl_3$). ^1H NMR (600 MHz, $CDCl_3$) δ 6.55 (dd, *J* = 11.9, 0.6 Hz, 1H), 4.89 (dd, *J* = 12.0, 8.8 Hz, 1H), 4.24 – 4.16 (m, 2H), 2.18 (qd, *J* = 9.3, 0.6 Hz, 1H), 1.55 (dd, *J* = 9.7, 5.5 Hz, 1H), 1.28 (t, *J* = 7.1 Hz, 3H), 1.20 – 1.08 (m, 3H), 1.06 (d, *J* = 6.9 Hz, 18H). ^{13}C NMR (151 MHz, $CDCl_3$) δ 166.3, 145.2, 124.6 (q, *J* = 272.2 Hz), 104.8, 61.8, 33.3 (q, *J* = 33.4 Hz), 24.7 (q, *J* = 2.0 Hz), 17.8, 17.3 (q, *J* = 2.1 Hz), 14.2, 12.1 (q, *J* = 1.5 Hz). ^{19}F NMR (565 MHz, $CDCl_3$) δ –61.0 (minor *cis*-diastereomer), –66.6 (*trans*-isomer). IR (ATR): ṽ = 2946, 2869, 1735, 1659, 1371, 1328, 1309, 1206, 1181, 1153, 1112, 1098, 925, 881, 787, 686, 664 cm^{-1}. HRMS (ESI+) *m/z* calcd. for $C_{18}H_{31}O_3F_3SiNa$ $[M+Na]^+$: calcd.: 403.18868; found: 403.18887.

The optical purity was determined by HPLC (Chiralcel OJ-3R, 4.6 mm ∅, acetonitrile/water = 50:50, v = 1.0 mL/min, λ = 220 nm): 32.28 min (minor) and 34.71 min (major).

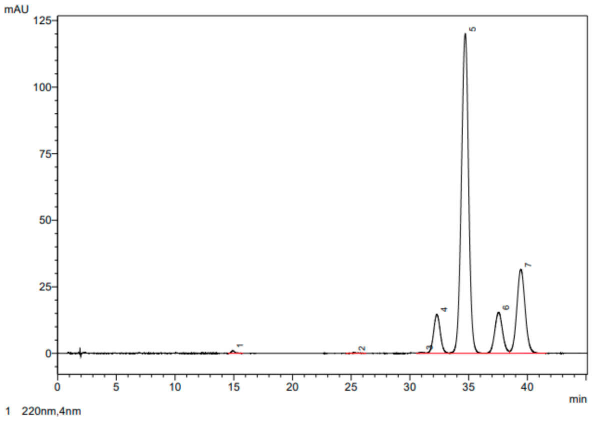

PDA Ch1 220nm

Peak #	Ret. Time	Area %	Name
1	14,93	0,25	
2	25,28	0,11	
3	31,04	0,18	
4	32,28	7,23	1. Enantiomer Diastereomer 1
5	34,71	64,10	2. Enantiomer Diastereomer 1 79.7 % ee
6	37,55	8,86	1. Enantiomer Diastereomer 2
7	39,45	19,25	2. Enantiomer Diastereomer 2 37.0 % ee
Total		100,00	

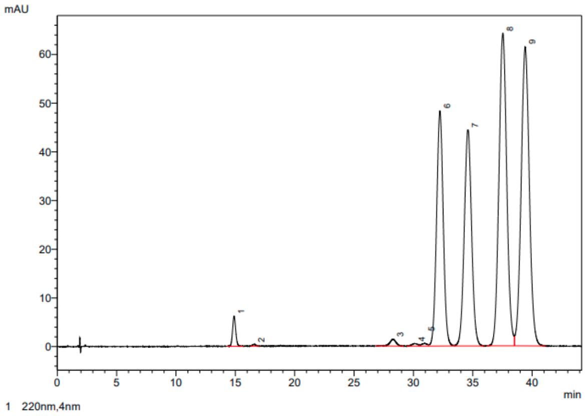

PDA Ch1 220nm

Peak #	Ret. Time	Area %	Name
1	14,90	1,15	
2	16,59	0,07	
3	28,30	0,53	
4	30,09	0,18	
5	30,92	0,18	
6	32,23	19,11	1. Enantiomer Diastereomer 1
7	34,59	19,34	2. Enantiomer Diastereomer 1
8	37,54	29,67	1. Enantiomer Diastereomer 2
9	39,42	29,75	2. Enantiomer Diastereomer 2
Total		100,00	

HPLC traces of **4n** (left) and the corresponding racemate (right).

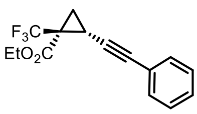

Compound 4o. Prepared according to the general procedure. The crude product was purified by flash chromatography (silica, *n*-pentane/*tert*-butyl methyl ether 99.5:0.5) to afford the title compound as a yellow oil (28.5 mg, 67%, d.r. = 6:1 (*trans*), 85% *ee*). $[\alpha]_D^{20}$ = +7.2 (c = 1.0, $CHCl_3$). ^1H NMR (400 MHz, $CDCl_3$) δ 7.40 – 7.36 (m, 2H), 7.32 – 7.27 (m, 3H), 4.30 (q, *J* = 7.1 Hz, 2H), 2.45 – 2.38 (m, 1H), 1.99 (ddq, *J* = 7.3, 5.5, 1.9 Hz, 1H), 1.73 (dd, *J* = 9.6, 5.4 Hz, 1H), 1.29 (t, *J* = 7.1 Hz, 3H). ^{13}C NMR (101 MHz, $CDCl_3$) δ 164.8, 131.9, 128.5, 128.4, 123.5 (d, *J* = 274.1 Hz), 122.7, 83.9, 81.2, 62.3, 34.5 (q, *J* = 33.9 Hz), 18.3 (t, *J* = 2.2 Hz), 14.6 (q, *J* = 2.7 Hz), 14.3. ^{19}F NMR (376 MHz, $CDCl_3$) δ –62.7 (minor diastereomer), –67.1 (major diastereomer). IR (ATR): ṽ = 1739, 1390, 1372, 1330, 1315, 1202, 1151, 1119, 1088, 755, 690 cm^{-1}. HRMS (EI) *m/z* calcd. for $C_{15}H_{13}O_2F_3$ $[M]^+$: calcd: 282.08586; found: 282.08622.

The optical purity was determined by HPLC (Chiralcel IG-3, 4.6 mm ∅, acetonitrile/water= 60:40, v= 1.0 mL/min, λ =220 nm): 4.39 min (major) and 5.63 min (minor).

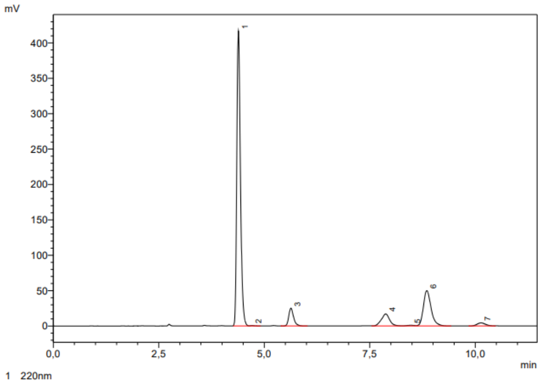

Detector A 220nm

Peak #	Ret. Time	Area %	Name
1	4,39	68,38	1. Enantiomer 85.1 % ee
2	4,71	0,13	
3	5,63	5,49	2. Enantiomer
4	7,88	6,48	
5	8,47	0,37	
6	8,85	17,42	
7	10,14	1,73	
Total		100,00	

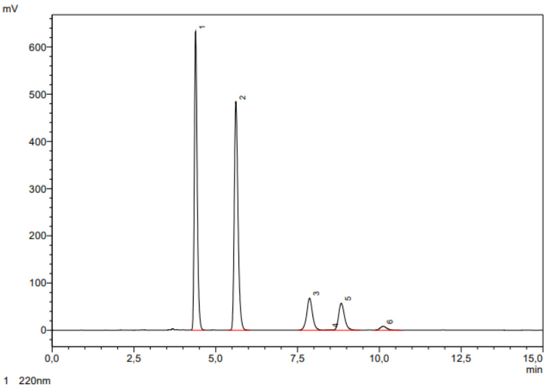

Detector A 220nm

Peak #	Ret. Time	Area %	Name
1	4,39	40,81	1. Enantiomer
2	5,62	41,13	2. Enantiomer
3	7,87	8,95	
4	8,46	0,12	
5	8,84	7,71	
6	10,12	1,27	
Total		100,00	

HPLC traces of **4o** (left) and the corresponding racemate (right).

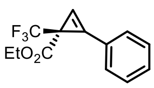

Compound 4p. Prepared according to the general procedure, but the reaction was performed at room temperature. The crude product was purified by flash chromatography (silica, *n*-pentane/*tert*-butyl methyl ether 99:01) to afford the title compound as a yellow oil (20 mg, 52%, 72% *ee*). $[\alpha]_D^{20}$ = –16.6 (c = 0.5, $CHCl_3$). ^1H NMR (400 MHz, $CDCl_3$) δ 7.65 – 7.55 (m, 2H), 7.52 – 7.42 (m, 3H), 6.82 (q, *J* = 1.4 Hz, 1H), 4.22 (q, *J* = 7.1 Hz, 2H), 1.25 (t, *J* = 7.1 Hz, 3H). ^{13}C NMR (101 MHz, $CDCl_3$) δ 169.3, 131.1, 130.4, 129.2, 124.8 (q, *J* = 276.4 Hz), 123.5, 111.2 (d, *J* = 1.6 Hz), 93.2 (d, *J* = 2.1 Hz), 61.7, 31.8 (q, *J* = 36.5 Hz), 14.2. ^{19}F NMR (282 MHz, $CDCl_3$) δ –64.6. IR (ATR): ṽ = 1728, 1298, 1269, 1190, 1142, 1127, 1038, 1025, 766, 171, 695 cm^{-1}. HRMS (EI) *m/z* calcd. for $C_{13}H_{11}O_2F_3$ $[M]^+$: calcd: 256.07083; found: 256.07057.

The optical purity was determined by HPLC (Chiralcel OJ-3R, 4.6 mm ∅, acetonitrile/water= 50:50, v= 1.0 mL/min, λ =220 nm): 12.05 min (minor) and 14.11 min (major).

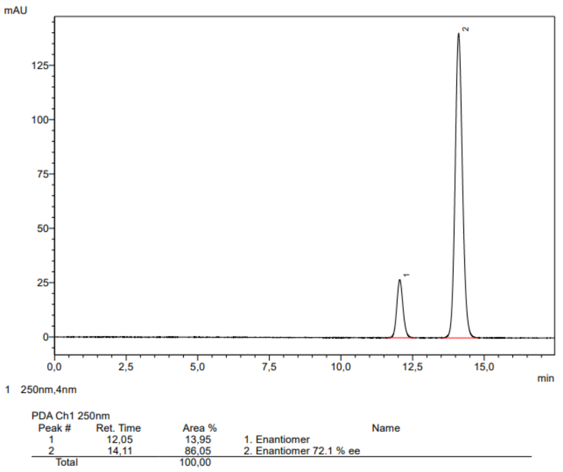

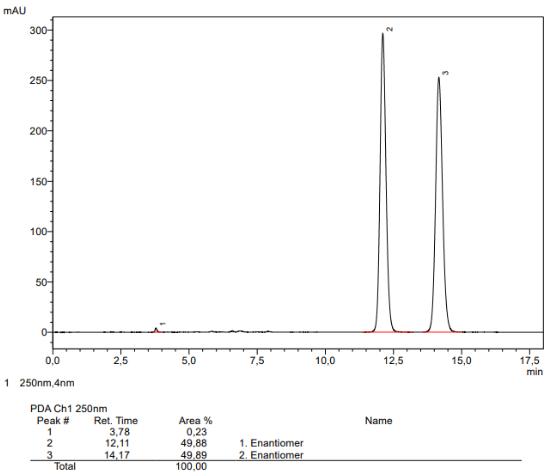

HPLC traces of **4p** (left) and the corresponding racemate (right).

Gram-Scale Cyclopropanation with Reduced Catalyst Loading

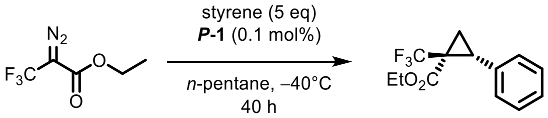

trans-**4a** (1.13 g, 72%, 96% *ee*)

An oven dried cooling-Schlenk flask equipped with a magnetic stir bar was charged with catalyst ***P*-1** (8 mg, 0.1 mol%). Styrene (3.5 mL, 30.2 mmol) and *n*-pentane (10 mL) were added and the mixture was cooled to –40°C. At this temperature, a solution of ethyl 3,3,3-trifluoro-2-diazopropionate (**3**) (1.11 g, 6.1 mmol) in *n*-pentane (20 mL) was added dropwise over 20 min and the resulting mixture was kept stirring at –40°C for 40 h. The mixture was concentrated under reduced pressure and the residue was purified by flash chromatography (silica, *n*-pentane/*tert*-butyl methyl ether 40:1) to afford product **4a** as a colorless oil (1.13 g, 72%, 96% *ee*). For the spectral data, see above.

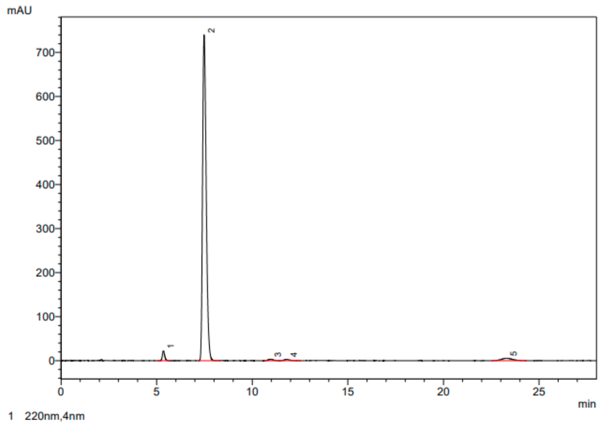

PDA Ch1 220nm

Peak #	Ret. Time	Area %	Name
1	5,35	1,84	1. Enantiomer
2	7,48	94,91	2. Enantiomer 96.2 % ee
3	10,96	0,57	
4	11,80	0,57	
5	23,29	2,12	
Total		100,00	

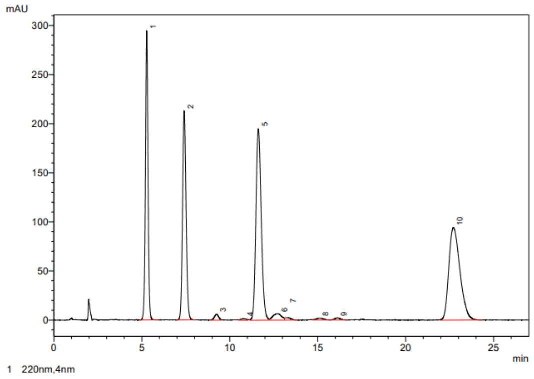

PDA Ch1 220nm

Peak #	Ret. Time	Area %	Name
1	5,28	20,23	1. Enantiomer Diastereomer 1
2	7,41	20,08	2. Enantiomer Diastereomer 1
3	9,24	0,69	
4	10,80	0,24	
5	11,62	28,03	1. Enantiomer Diastereomer 2
6	12,74	1,53	
7	13,23	0,40	
8	15,09	0,41	
9	16,12	0,36	
10	22,71	28,04	2. Enantiomer Diastereomer 2
Total		100,00	

HPLC traces of **4a** (gram scale experiment) (left) and the corresponding racemate (right).

Ester Cleavage

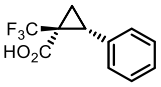

Acid 5a. A microwave vial (10 mL) equipped with a magnetic stirring bar was charged with ester **4a** (100 mg, 0.35 mmol). Ethanol/THF/water (1:1:1, 6 mL) was added, the vial was sealed, and the mixture stirred at 100°C for 12 h in a microwave reactor. The solvent was evaporated under reduced pressure and the remaining aqueous residue was acidified with aq. HCl (1 M). The residue was extracted with CH_2Cl_2 (3 x 10 mL), the combined organic phases were washed with brine (20 mL), dried over anhydrous $MgSO_4$ and concentrated under reduced pressure. The residue was purified by flash chromatography (silica, *n*-pentane/EtOAc 10:1 + 1% formic acid) to afford the title compound as a colorless oil that solidified upon standing at 4°C (78.2 mg, 97%). $[\alpha]_D^{20}$ = –11.6 (c = 1.0, $CHCl_3$). ^1H NMR (400 MHz, $CDCl_3$) δ 7.33 – 7.22 (m, 3H), 7.23 – 7.16 (m, 2H), 3.02 (t, *J* = 9.1 Hz, 1H), 2.09 (dtd, *J* = 9.3, 3.8, 1.8 Hz, 1H), 1.82 (dd, *J* = 9.7, 5.7 Hz, 1H). ^{13}C NMR (101 MHz, $CDCl_3$) δ 170.7, 133.0, 129.2, 128.5, 127.9, 124.1 (q, *J* = 274.3 Hz), 34.1 (q, *J* = 34.1 Hz), 30.6 (q, *J* = 1.8 Hz), 16.1 (q, *J* = 2.0 Hz).

The spectral data match the literature.[6]

The optical purity was determined by HPLC (Chiralcel OJ-3R, 4.6 mm ∅, methanol/water = 70:30 + 0.1% TFA, v = 0.5 mL/min, λ = 220 nm): 12.24 min (minor) and 13.20 min (major).

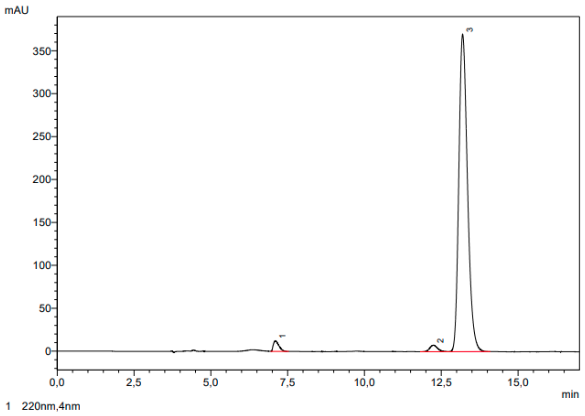

PDA Ch1 220nm

Peak #	Ret. Time	Area %	Name
1	7,10	2,09	
2	12,24	1,81	1. Enantiomer
3	13,20	96,10	2. Enantiomer 96.3 % ee
Total		100,00	

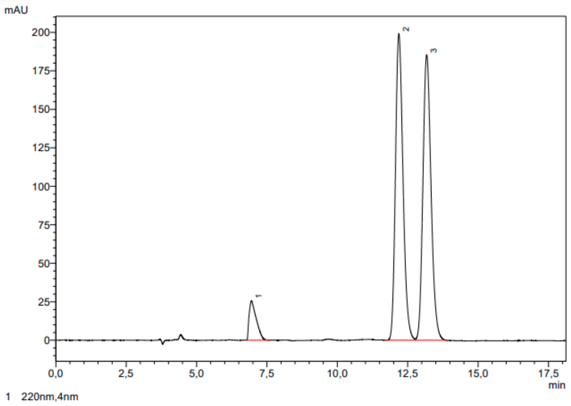

PDA Ch1 220nm

Peak #	Ret. Time	Area %	Name
1	6,95	5,74	
2	12,19	47,06	1. Enantiomer
3	13,17	47,20	2. Enantiomer
Total		100,00	

HPLC traces of **5a** (left) and the corresponding racemate (right).

Cyclopropanations With Donor/Acceptor Carbenes Carrying Fluorinated Phenyl Substituents

Substrates

General Procedure. Esterification. An oven-dried cooling Schlenk flask equipped with a magnetic stir bar was charged with the corresponding phenylacetic acid derivative (10 mmol, 1 equiv.), ethanol (1.2 equiv.), DMAP (10 mol%) and acetonitrile (20 mL). The reaction mixture was cooled to 0°C and DCC (1.1 equiv.) was added in portions. After 2 h, the reaction mixture was filtered and the precipitate was washed with *tert*-butyl methyl ether. The combined filtrates were concentrated and the residue purified by flash chromatography (silica, *i*-hexane/*tert*-butyl methyl ether).

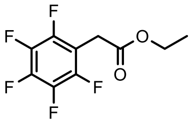

Compound S10. Prepared according to the general procedure. The crude material was purified by flash chromatography (silica, *i*-hexane/*tert*-butyl methyl ether 50:1) to give the title compound as a colorless oil (95%). ^{1}H NMR (600 MHz, $CDCl_3$) δ 4.20 (q, *J* = 7.1 Hz, 2H), 3.74 – 3.70 (m, 2H), 1.28 (t, *J* = 7.1 Hz, 3H). ^{13}C NMR (151 MHz, $CDCl_3$) δ 168.4, 145.5 (dm, *J* = 251.2 Hz), 140.8 (dm, *J* = 253.5 Hz), 137.6 (dm, *J* = 251.9 Hz), 108.3 (td, *J* = 18.5, 4.1 Hz), 62.0, 28.0, 14.2. ^{19}F NMR (565 MHz, $CDCl_3$) δ –142.27 – –142.35 (m), –155.29 (t, *J* = 20.8 Hz), –162.29 – –162.40 (m). IR (ATR): ṽ = 1742, 1522, 1505, 1304, 1184, 1127, 1010, 976, 916 cm^{-1}. HRMS (EI) *m/z* calcd. for $C_{10}H_7O_2F_5$ $[M]^+$: calcd.: 254.03607; found: 254.03624.

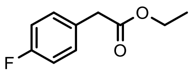

Compound S11. Prepared according to the general procedure. The crude material was purified by flash chromatography (silica, *i*-hexane/*tert*-butyl methyl ether 20:1) to give the title compound as a colorless oil (91%). ^{1}H NMR (400 MHz, $CDCl_3$) δ 7.30 – 7.20 (m, 2H), 7.06 – 6.96 (m, 2H), 4.15 (q, *J* = 7.1 Hz, 2H), 3.58 (s, 2H), 1.25 (t, *J* = 7.1 Hz, 3H). ^{13}C NMR (101 MHz, $CDCl_3$) δ 171.6, 162.1 (d, *J* = 245.4 Hz), 130.9 (d, *J* = 8.0 Hz), 130.0 (d, *J* = 3.4 Hz), 115.4 (d, *J* = 21.7 Hz), 61.1, 40.7, 14.3. ^{19}F NMR (282 MHz, $CDCl_3$) δ –115.9. IR (ATR): ṽ = 1731, 1509, 1253, 1220, 1152, 1095, 1030, 825, 798 cm^{-1}. HRMS (EI) *m/z* calcd. for $C_{10}H_{11}O_2F$ $[M]^+$: calcd.: 182.07376; found: 182.07364.

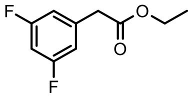

Compound S12. Prepared according to the general procedure. The crude product was purified by flash chromatography (silica, *i*-hexane/*tert*-butyl methyl ether 50:1) to give the title compound as a colorless oil (86%). ^{1}H NMR (400 MHz, $CDCl_3$) δ 6.87 – 6.78 (m, 2H), 6.71 (tt, *J* = 9.0, 2.4 Hz, 1H), 4.17 (q, *J* = 7.1 Hz, 2H), 3.58 (s, 2H), 1.26 (t, *J* = 7.1 Hz, 3H). ^{13}C NMR (101 MHz, $CDCl_3$) δ 170.5, 163.1 (dd, *J* = 248.3, 12.9 Hz), 137.7 (t, *J* = 9.8 Hz), 112.4 (dd, *J* = 18.5, 7.0 Hz), 102.8 (t, *J* = 25.2 Hz), 61.4, 41.1 (t, *J* = 2.1 Hz), 14.3. ^{19}F NMR (282 MHz, $CDCl_3$) δ –110.1. IR (ATR): ṽ = 1733, 1626, 1596, 1461, 1309, 1161, 1139, 1117, 1031, 995, 848, 671 cm^{-1}. HRMS (EI) *m/z* calcd. for $C_{10}HO_2F_2$ $[M]^+$: calcd.: 200.06434; found: 200.06433.

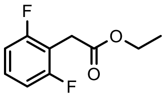

Compound S13. Prepared according to the general procedure. The crude material was purified by flash chromatography (silica, *i*-hexane/*tert*-butyl methyl ether 50:1) to give the title compound as a colorless oil (95%). ^{1}H NMR (400 MHz, $CDCl_3$) δ 7.28 – 7.18 (m, 1H), 6.94 – 6.85 (m, 2H), 4.18 (q, *J* = 7.1 Hz, 2H), 3.70 (s, 2H), 1.26 (t, *J* = 7.1 Hz, 3H). ^{13}C NMR (101 MHz, $CDCl_3$) δ 169.8, 161.7 (dd, *J* = 248.1, 8.0 Hz), 129.1 (t, *J* = 10.2 Hz), 111.2 (dd, *J* = 18.8, 6.4 Hz), 110.8 (t, *J* = 20.1 Hz), 61.4, 28.2 (t, *J* = 3.2 Hz), 14.2. ^{19}F NMR (282 MHz, $CDCl_3$) δ –114.7. IR (ATR): ṽ = 1737, 1470, 1336, 1271, 1238, 1213, 1163, 1015, 782 cm^{-1}. HRMS (EI) *m/z* calcd. for $C_{10}H_{10}O_2F_2$ $[M]^+$: calcd.: 200.06434; found: 200.06431.

Diazo Transfer. Procedure A. An oven dried cooling Schlenk flask equipped with a magnetic stir bar was charged with the ester derivative (1 mmol, 1 equiv.), *ortho*-nitrobenzenesulfonyl azide (*o*-NBSA, 1.5 equiv.) and acetonitrile (7 mL). The mixture was cooled to 0°C before DBU (2 equiv.) was added dropwise. After stirring for 20-22 h, sat. aq. NH_4Cl was added and the mixture was extracted three times with *tert*-butyl methyl ether. The combined organic layers were dried over $MgSO_4$ and concentrated, and the residue was purified by flash chromatography (silica, *i*-hexane/*tert*-butyl methyl ether).

Diazo Transfer. Procedure B. An oven dried cooling Schlenk flask equipped with a magnetic stir bar was charged with the ester derivative (1 mmol, 1 equiv.), *para*-acetamidobenzenesulfonyl azide (*p*-ABSA, 1.2 equiv.) and acetonitrile (4 mL). The mixture was cooled to 0°C before DBU (1.2 equiv.) was added dropwise. After stirring for 20-22 h, water was added and the mixture was extracted three times with *tert*-butyl methyl ether. The combined organic layers were dried over $MgSO_4$ and concentrated, and the residue was purified by flash chromatography (silica, *i*-hexane/*tert*-butyl methyl ether).

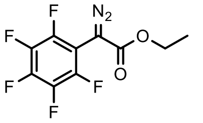

Diazoester 8. Prepared according to procedure A. The crude product was purified by flash chromatography (silica, *i*-hexane/*tert*-butyl methyl ether 20:1) to afford the title compound as a yellow oil (62%). ^{1}H NMR (600 MHz, $CDCl_3$) δ 4.31 (q, *J* = 7.1 Hz, 2H), 1.31 (t, *J* = 7.1 Hz, 3H). ^{13}C NMR (151 MHz, $CDCl_3$) δ 163.6, 144.8 (ddd, *J* = 250.9, 11.3, 5.4 Hz), 141.9 (dm, *J* = 256.9 Hz), 138.0 (dm, *J* = 253.7 Hz), 101.9 (td, *J* = 17.0, 4.1 Hz), 62.2, 52.3, 14.5. ^{19}F NMR (565 MHz, $CDCl_3$) δ –136.59 – –136.64 (m), –136.64 – –136.68 (m), –152.24 (t, *J* = 20.9 Hz), –161.08 – –161.21 (m). IR (ATR): ṽ = 2105, 1705, 1520, 1494, 1277, 1247, 1137, 1066, 1007, 982, 882 cm^{-1}. HRMS (EI) *m/z* calcd. for $C_{10}H_5F_5N_2O_2Na$ $[M+Na]^+$: calcd.: 303.01634; found: 303.01644.

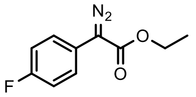

Diazoester S14. Prepared according to procedure B. The crude product was purified by flash chromatography (silica, *i*-hexane/*tert*-butyl methyl ether 20:1) to afford the title compound as a yellow oil (26%). ^{1}H NMR (400 MHz, $CDCl_3$) δ 7.48 – 7.40 (m, 2H), 7.12 – 7.04 (m, 2H), 4.33 (q, *J* = 7.1 Hz, 2H), 1.34 (t, *J* = 7.1 Hz, 3H). ^{13}C NMR (101 MHz, $CDCl_3$) δ 165.4, 161.1 (d, *J* = 246.3 Hz), 126.0 (d, *J* = 7.9 Hz), 121.5 (d, *J* = 3.4 Hz), 116.1 (d, *J* = 22.0 Hz), 62.7, 61.2, 14.6. ^{19}F NMR (282 MHz, $CDCl_3$) δ –116.4. IR (ATR): ṽ = 2080, 1696, 1509, 1341, 1287, 1233, 1154, 1044, 831, 608 cm^{-1}. HRMS (EI) *m/z* calcd. for $C_{10}H_9FN_2O_2Na$ $[M+Na]^+$: calcd.: 231.05403; found: 231.05429.

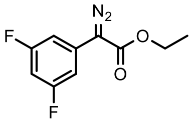

Diazoester S15. Prepared according to procedure B. The crude product was purified by flash chromatography (silica, *i*-hexane/*tert*-butyl methyl ether 20:1) to afford the title compound as a yellow oil (62%). ^{1}H NMR (400 MHz, $CDCl_3$) δ 7.12 – 7.00 (m, 2H), 6.60 (tt, *J* = 8.8, 2.3 Hz, 1H), 4.34 (q, *J* = 7.1 Hz, 2H), 1.35 (t, *J* = 7.1 Hz, 3H). ^{13}C NMR (101 MHz, $CDCl_3$) δ 164.2, 163.5 (dd, *J* = 247.4, 13.7 Hz), 129.9 (t, *J* = 11.5 Hz), 106.4 (dd, *J* = 20.2, 8.6 Hz), 100.9 (t, *J* = 25.6 Hz), 64.1, 61.5, 14.5. ^{19}F NMR (282 MHz, $CDCl_3$) δ –108.8. IR (ATR): ṽ = 2085, 1700, 1622, 1588, 1269, 1243, 1219, 1139, 1118, 1071, 987, 837, 670 cm^{-1}. HRMS (EI) *m/z* calcd. for $C_{10}H_8F_2N_2O_2Na$ $[M+Na]^+$: calcd.: 249.04460; found: 249.04472.

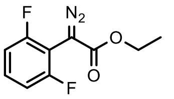

Diazoester S16. Prepared according to procedure B. The crude product was purified by flash chromatography (silica, *i*-hexane/*tert*-butyl methyl ether 20:1) to afford the title compound as a yellow oil (70%). ^{1}H NMR (300 MHz, $CDCl_3$) δ 7.38 – 7.27 (m, 1H), 7.01 – 6.90 (m, 2H), 4.29 (q, *J* = 7.1 Hz, 2H), 1.29 (t, *J* = 7.1 Hz, 3H). ^{13}C NMR (101 MHz, $CDCl_3$) δ 164.6, 160.6 (dd, *J* = 251.7, 5.8 Hz), 130.9 (t, *J* = 10.3 Hz), 112.0 – 111.6 (m), 103.7 (t, *J* = 18.5 Hz), 61.5, 53.1, 14.2. ^{19}F NMR (282 MHz, $CDCl_3$) δ –109.0. IR (ATR): ṽ = 2097, 1702, 1467, 1295, 1263, 1235, 1173, 1156,

1037, 1001, 784, 744 cm^{-1}. HRMS (EI) *m/z* calcd. for $C_{10}H_8F_2N_2O_2Na$ $[M+Na]^+$: calcd.: 249.04460; found: 249.04474.

General Procedure for the Cyclopropanations. An oven dried cooling Schlenk flask equipped with a magnetic stir bar was charged with catalyst ***M*-1** (0.5 mol%). The respective olefin (5 equiv.) and CH_2Cl_2 (1 mL) were added. A solution of the respective diazo ester (0.1 mmol, 1 equiv.) in CH_2Cl_2 (2 mL) was added dropwise over 5 min and the mixture was stirred at room temperature for 20-22 h. The mixture was concentrated under reduced pressure and the *trans:cis* of the crude material was determined by ^{1}H NMR. The crude product was then purified by flash chromatography (silica, *n*-pentane/*tert*-butyl methyl ether). If the diastereomers were separable under these conditions, only the major diastereomer was collected.

The corresponding racemic compounds needed as references for the ee determinations were prepared analogously using commercial $Rh_2(esp)_2$ (1 mol%) as the catalyst.

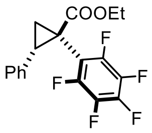

Compound 9a. Prepared according to the general procedure. The crude product was purified by flash chromatography (silica, *n*-pentane/*tert*-butyl methyl ether 50:1) to afford the title compound as a colorless oil (24 mg, 61%, *trans:cis* > 20:1, 95% *ee* (*trans*)). $[\alpha]_D^{20}$ = –39.9 (c = 1.0, $CHCl_3$). ^{1}H NMR (500 MHz, $CDCl_3$) δ 7.17 – 7.11 (m, 3H), 6.90 (dd, *J* = 6.6, 2.9 Hz, 2H), 4.18 (ddq, *J* = 45.7, 10.8, 7.1 Hz, 2H), 3.25 (ddt, *J* = 9.4, 7.7, 1.7 Hz, 1H), 2.25 (dd, *J* = 9.5, 6.0 Hz, 1H), 2.07 (ddt, *J* = 7.1, 6.1, 1.2 Hz, 1H), 1.21 (t, *J* = 7.1 Hz, 3H). ^{13}C NMR (126 MHz, $CDCl_3$) δ 171.1, 146.6 (dd, *J* = 246.6, 65.8 Hz), 140.6 (dm, *J* = 253.6 Hz), 137.0 (dm, *J* = 249.7 Hz), 134.7, 128.1, 127.4 (2C), 109.7 (t, *J* = 15.9 Hz), 62.2, 33.3, 27.9, 20.1 (d, *J* = 5.4 Hz), 14.2. ^{19}F NMR (470 MHz, $CDCl_3$) δ –135.6, –141.7, –154.4 (t, *J* = 21.1 Hz), –162.9, –163.3. IR (ATR): ṽ = 1726, 1523, 1498, 1265, 1156, 1095, 1080, 990, 695 cm^{-1}. HRMS (EI) *m/z* calcd. for $C_{18}H_{13}O_2F_5$ $[M]^+$: calcd.: 356.08302; found: 356.08289.

The optical purity was determined by HPLC (Chiralpak IB-N-3, 4.6 mm ∅, acetonitrile/water= 50:50, v = 1.0 mL/min, λ = 220 nm): 20.49 min (major) and 22.30 min (minor).

The relative stereochemistry of **9a** was determined with the aid of ^{1}H-^{1}H NOESY and ^{1}H-^{19}F HOESY spectra (see copies of spectra on pages S91-S92)

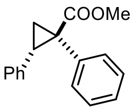

7a, R = Me
b, R = CH_2Cl_3

The absolute configuration of **9a** and all compounds shown below was assigned in analogy to the known configuration of cyclopropanes **7a**,**b** formed on reaction of the corresponding 2-phenyl-2-diazoacetate derivative with styrene in te presence of catalyst ***M*-1**.[7]

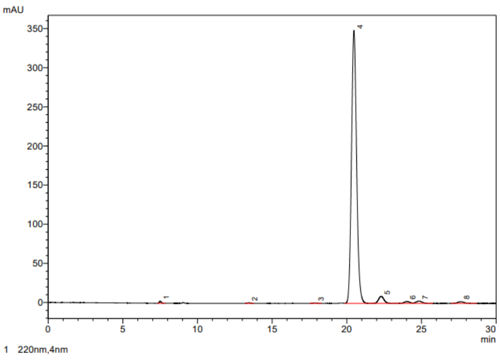

PDA Ch1 220nm

Peak #	Ret. Time	Area %	Name
1	7,50	0,26	
2	13,44	0,12	
3	17,87	0,13	
4	20,48	94,35	1. Enantiomer 94.6 % ee
5	22,30	2,64	2. Enantiomer
6	24,03	0,75	
7	24,85	0,99	
8	27,64	0,77	
Total		100,00	

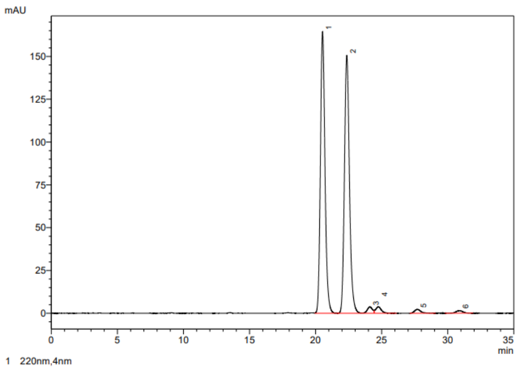

PDA Ch1 220nm

Peak #	Ret. Time	Area %	Name
1	20,53	47,86	1. Enantiomer
2	22,37	48,00	2. Enantiomer
3	24,11	1,22	
4	24,75	1,35	
5	27,71	0,90	
6	30,89	0,67	
Total		100,00	

HPLC traces of **9a** (left) and the corresponding racemate (right).

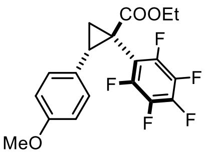

Compound 9b. Prepared according to the general procedure. The crude product was purified by flash chromatography (silica, *n*-pentane/*tert*-butyl methyl ether 50:1) to afford the title compound as a colorless oil (24 mg, 62%, *trans:cis* > 20:1, 86% *ee* (*trans*)). ^{1}H NMR (400 MHz, $CDCl_3$) δ 6.89 – 6.79 (m, 2H), 6.72 – 6.64 (m, 2H), 4.17 (ddq, *J* = 38.2, 10.8, 7.1 Hz, 2H), 3.74 (s, 3H), 3.21 (ddt, *J* = 9.5, 7.7, 1.7 Hz, 1H), 2.23 (dd, *J* = 9.6, 5.9 Hz, 1H), 2.01 (ddt, *J* = 8.0, 5.9, 1.2 Hz, 1H), 1.20 (t, *J* = 7.1 Hz, 3H). ^{13}C NMR (101 MHz, $CDCl_3$) δ 171.1, 158.9, 146.8 (dm, *J* = 248.9 Hz), 140.7 (dm, *J* = 254.5 Hz), 137.3 (dm, *J* = 251.9 Hz), 128.7, 126.8, 113.6, 110.3 (m), 62.0, 55.3, 32.9, 27.9, 20.1 (t, *J* = 3.1 Hz), 14.2. ^{19}F NMR (282 MHz, $CDCl_3$) δ –135.6, –141.7, –154.6 (t, *J* = 21.3 Hz), –163.1. IR (ATR): ṽ = 1725, 1522, 1498, 1254, 1177, 1156, 1089, 1034, 989, 840 cm^{-1}. HRMS (EI) *m/z* calcd. for $C_{19}H_{15}O_3F_5$ $[M]^+$: calcd.: 386.09359; found: 386.09413.

$[\alpha]_D^{20}$ = –30.3 (c = 1.0, $CHCl_3$). The optical purity was determined by HPLC (Chiralpak IB-N-3, 4.6 mm ∅, acetonitrile/water= 50:50, v = 1.0 mL/min, λ = 240 nm): 20.35 min (major) and 21.49 min (minor).

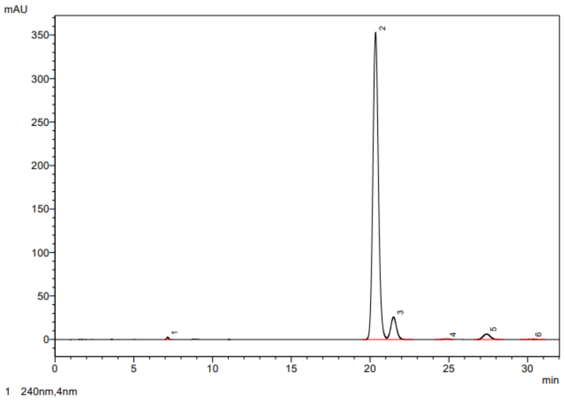

PDA Ch1 240nm

Peak #	Ret. Time	Area %	Name
1	7,15	0,23	
2	20,35	90,17	1. Enantiomer 85.6 % ee
3	21,49	7,01	2. Enantiomer
4	24,83	0,28	
5	27,40	2,19	
6	30,24	0,12	
Total		100,00	

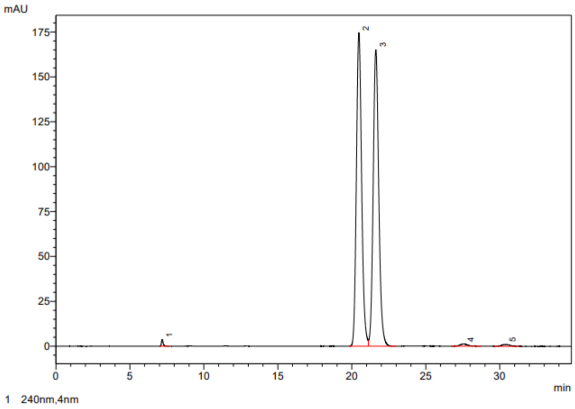

PDA Ch1 240nm

Peak #	Ret. Time	Area %	Name
1	7,19	0,34	
2	20,48	49,23	1. Enantiomer
3	21,62	49,48	2. Enantiomer
4	27,56	0,50	
5	30,39	0,44	
Total		100,00	

HPLC traces of **9b** (left) and the corresponding racemate (right).

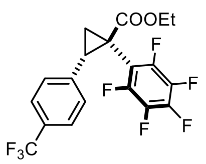

Compound 9c. Prepared according to the general procedure. The crude product was purified by flash chromatography (silica, *n*-pentane/*tert*-butyl methyl ether 50:1) to afford the title compound as a colorless oil (35 mg, 82%, *trans:cis* > 20:1, 97% *ee* (*trans*)). $[\alpha]_D^{20}$ = –43.0 (c = 1.0, $CHCl_3$). ^1H NMR (400 MHz, $CDCl_3$) δ 7.46 – 7.38 (m, 2H), 7.02 (d, *J* = 8.1 Hz, 2H), 4.30 – 4.08 (m, 2H), 3.30 (tt, *J* = 9.2, 1.6 Hz, 1H), 2.31 (dd, *J* = 9.5, 6.1 Hz, 1H), 2.08 (ddt, *J* = 7.3, 6.0, 1.1 Hz, 1H), 1.21 (t, *J* = 7.1 Hz, 3H). ^{13}C NMR (101 MHz, $CDCl_3$) δ 170.69, 146.8 (dm, *J* = 250.2 Hz), 141.1 (dm, *J* = 254.4 Hz), 139.4, 137.4 (dm, *J* = 250.6 Hz), 129.7 (q, *J* = 32.5 Hz), 127.9, 125.2 (q, *J* = 3.8 Hz), 124.1 (q, *J* = 271.7 Hz), 109.5 (m),62.4, 32.5, 28.4, 20.4 (t, *J* = 3.2 Hz), 14.2. ^{19}F NMR (565 MHz, $CDCl_3$) δ –62.7, –136.1, –141.5, –153.4 (t, *J* = 20.9 Hz), –162.2, –162.6. IR (ATR): ṽ = 1728, 1523, 1499, 1326, 1266, 1166, 1121, 1089, 1068, 991, 850 cm^{-1}. HRMS (EI) *m/z* calcd. for $C_{19}H_{12}F_8O_2Na$ $[M+Na]^+$: calcd.: 447.06018; found: 447.06032.

The optical purity was determined by HPLC (Chiralpak IB-N-3, 4.6 mm ∅, acetonitrile/water = 50:50, v = 1.0 mL/min, λ = 225 nm): 25.02 min (major) and 26.88 min (minor).

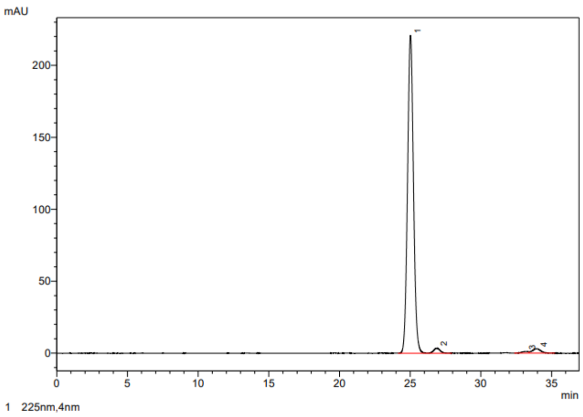

PDA Ch1 225nm

Peak #	Ret. Time	Area %	Name
1	25,02	95,90	1. Enantiomer 96.6 % ee
2	26,88	1,63	2. Enantiomer
3	33,13	0,57	
4	33,95	1,91	
Total		100,00	

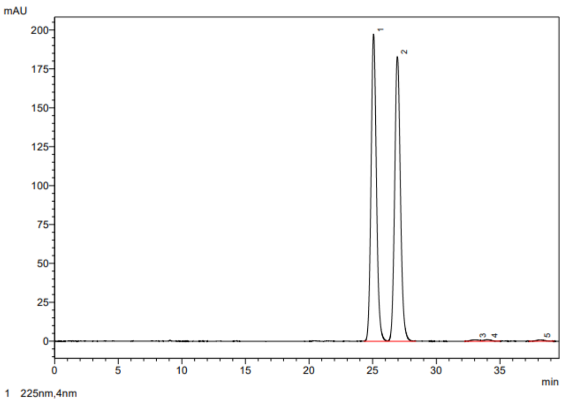

PDA Ch1 225nm

Peak #	Ret. Time	Area %	Name
1	25,07	49,50	1. Enantiomer
2	26,94	49,52	2. Enantiomer
3	33,11	0,36	
4	34,03	0,32	
5	38,19	0,30	
Total		100,00	

HPLC traces of **9c** (left) and the corresponding racemate (right).

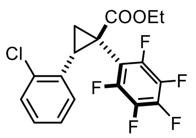

Compound 9d. Prepared according to the general procedure. The crude product was purified by flash chromatography (silica, *n*-pentane/*tert*-butyl methyl ether 50:1) to afford the title compound as a colorless oil (23 mg, 58%, *trans:cis* > 20:1, 97% *ee* (*trans*)). $[\alpha]_D^{20}$ = +40.8 (c = 1.0, $CHCl_3$). ^1H NMR (600 MHz, $CDCl_3$) δ 7.38 (dd, J = 8.0, 1.2 Hz, 1H), 7.10 (ddd, J = 8.0, 7.4, 1.6 Hz, 1H), 6.93 (tdd, J = 7.3, 1.3, 0.5 Hz, 1H), 6.37 (dd, J = 7.9, 1.6 Hz, 1H), 4.20 (ddq, J = 40.0, 10.8, 7.1 Hz, 2H), 3.70 (ddt, J = 9.5, 8.0, 1.5 Hz, 1H), 2.29 (dd, J = 9.6, 6.1 Hz, 1H), 2.12 – 2.06 (m, 1H), 1.22 (t, J = 7.1 Hz, 3H). ^{13}C NMR (151 MHz, $CDCl_3$) δ 170.6, 146.9 (dm, *J* = 251.6 Hz), 140.9 (dm, *J* = 254.1 Hz), 137.2 (dm, *J* = 250.4 Hz), 136.9, 132.7, 129.9, 128.5, 126.1, 125.2, 109.71 (td, *J* = 16.0, 4.1 Hz), 62.2, 30.2, 28.1, 19.5 (t, *J* = 3.1 Hz), 14.2. ^{19}F NMR (565 MHz, $CDCl_3$) δ –135.8, –141.1,–-154.0 (t, *J* = 21.1 Hz), –162.9. IR (ATR): ṽ = 1726, 1523, 1497, 1444, 1262, 1157, 1088, 1053, 1024, 989, 759, 744 cm^{-1}. HRMS (EI) *m/z* calcd. for $C_{18}H_{12}ClF_5O_2Na$ $[M+Na]^+$: calcd.: 413.03382; found: 413.03399.

The optical purity was determined by HPLC (Chiralpak IB-N-3, 4.6 mm ∅, acetonitrile/water = 50:50, v = 1.0 mL/min, λ = 220 nm): 26.17 min (major) and 28.87 min (minor).

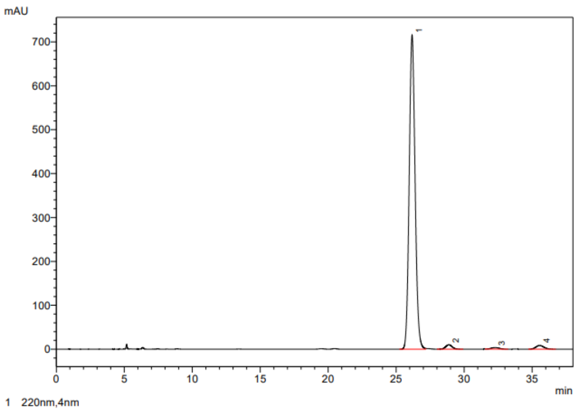

PDA Ch1 220nm

Peak #	Ret. Time	Area %	Name
1	26,17	96,23	1. Enantiomer 96.9 % ee
2	28,87	1,53	2. Enantiomer
3	32,26	0,66	
4	35,55	1,58	
Total		100,00	

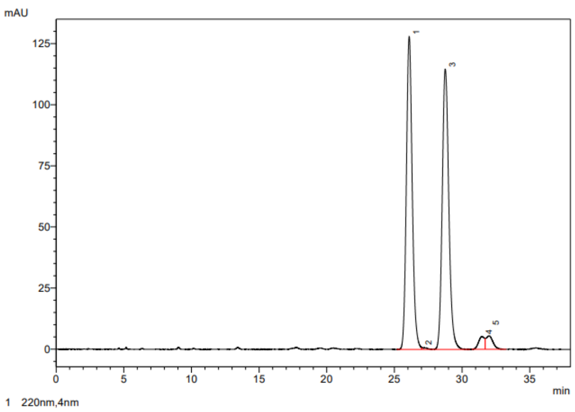

PDA Ch1 220nm

Peak #	Ret. Time	Area %	Name
1	26.10	47.54	1. Enantiomer
2	27.02	0.23	
3	28.76	47.79	2. Enantiomer
4	31.47	1.97	
5	31.98	2.48	
Total		100.00	

HPLC traces of **9d** (left) and the corresponding racemate (right).

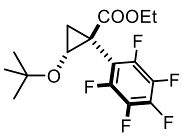

Compound 9e. Prepared according to the general procedure. The crude product was purified by flash chromatography (silica, *n*-pentane/*tert*-butyl methyl ether 50:1) to afford the title compound as a colorless oil (27 mg, 77%, *trans:cis* > 20:1, 92% *ee* (*trans*)). $[\alpha]_D^{20}$ = –37.8 (c = 1.0, $CHCl_3$). ^{1}H NMR (400 MHz, $CDCl_3$) δ 4.22 – 4.01 (m, 2H), 3.95 (ddt, *J* = 6.3, 4.9, 1.5 Hz, 1H), 1.95 (t, *J* = 6.0 Hz, 1H), 1.40 (ddt, *J* = 6.1, 4.9, 1.2 Hz, 1H), 1.19 – 1.16 (m, 12H). ^{13}C NMR (101 MHz, $CDCl_3$) δ 171.1, 147.2 (dm, *J* = 245.6 Hz), 140.6 (dm, *J* = 252.2 Hz), 137.4 (dm, *J* = 248.5 Hz), 110.0 (m), 76.0, 61.8, 57.1, 28.1, 24.5 (t, *J* = 2.4 Hz), 23.3, 14.3. ^{19}F NMR (376 MHz, $CDCl_3$) δ –139.8, –155.9 (t, *J* = 21.0 Hz), –164.0 – –164.2 (m). IR (ATR): ṽ = 1727, 1523, 1499, 1261, 1170, 1144, 1093, 1080, 1011, 990, 942, 799 cm^{-1}. HRMS (EI) *m/z* calcd. for $C_{16}H_{17}F_5O_3Na$ $[M+Na]^+$: calcd.: 375.09901; found: 375.09906.

The optical purity was determined by HPLC (Chiralcel OJ-3R, 4.6 mm ∅, acetonitrile/water = 50:50, v = 1.0 mL/min, λ = 220 nm): 11.90 min (minor) and 12.34 min (major).

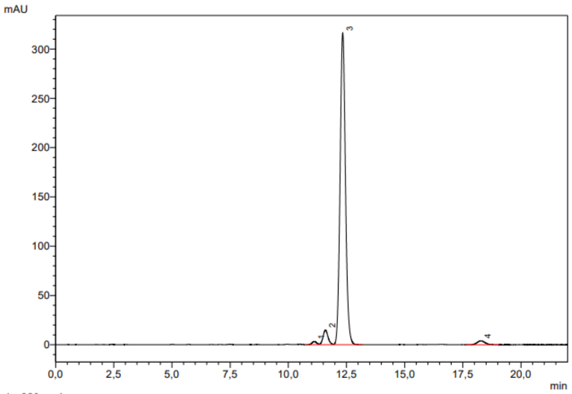

1 220nm,4nm

PDA Ch1 220nm

Peak #	Ret. Time	Area %	Name
1	11,12	0,85	
2	11,60	4,07	1. Enantiomer
3	12,34	93,30	2. Enantiomer 91.6 % ee
4	18,28	1,78	
Total		100,00	

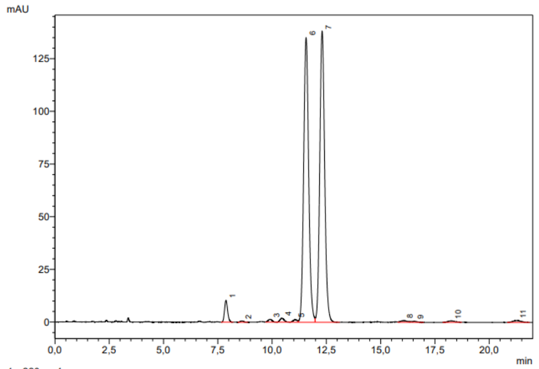

1 220nm,4nm

PDA Ch1 220nm

Peak #	Ret. Time	Area %	Name
1	7,88	2,24	
2	8,62	0,15	
3	9,92	0,38	
4	10,45	0,57	
5	11,06	0,36	
6	11,57	47,36	1. Enantiomer
7	12,31	47,52	2. Enantiomer
8	16,05	0,37	
9	16,54	0,21	
10	18,27	0,35	
11	21,26	0,50	
Total		100,00	

HPLC traces of **9e** (left) and the corresponding racemate (right).

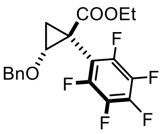

Compound 9f. Prepared according to the general procedure. The crude product was purified by flash chromatography (silica, *n*-pentane/*tert*-butyl methyl ether 50:1) to afford the title compound as a colorless oil (19 mg, 48% (contains minor unknown impurity), *trans:cis* > 20:1, 80% *ee* (*trans*)). $[\alpha]_D^{20}$ = +12.0 (c = 1.0, $CHCl_3$). ^{1}H NMR (400 MHz, $CDCl_3$) δ 7.37 – 7.27 (m, 3H), 7.22 – 7.18 (m, 2H), 4.68 – 4.58 (m, 2H), 4.21 – 4.04 (m, 3H), 1.98 (t, *J* = 6.7 Hz, 1H), 1.59 (ddt, *J* = 6.2, 4.9, 1.1 Hz, 1H), 1.19 (t, *J* = 7.1 Hz, 3H). ^{13}C NMR (101 MHz, $CDCl_3$) δ 170.3, 147.1 (dm, *J* = 248.5 Hz), 141.0 (dm, *J* = 253.6 Hz), 137.6 (dm, *J* = 246.2 Hz), 137.2, 128.7, 128.1, 127.7, 109.7 (m), 73.4, 63.9, 61.9, 24.8, 22.2 (t, *J* = 2.8 Hz), 14.2. ^{19}F NMR (565 MHz, $CDCl_3$) δ –137.8, –140.9, –155.0 (t, *J* = 21.0 Hz), –163.2. IR (ATR): ṽ = 1726, 1523, 1497, 1271, 1147, 1088, 1064, 1011, 982, 797, 740, 697 cm^{-1}. HRMS (EI) *m/z* calcd. for $C_{19}H_{15}O_3F_5Na$ $[M+Na]^+$: calcd.: 409.08336; found: 409.08356.

The optical purity was determined by HPLC (Chiralcel OJ-3R, 4.6 mm ∅, methanol/water = 80:20, v = 1.0 mL/min, λ = 220 nm): 18.36 min (minor) and 22.73 min (major).

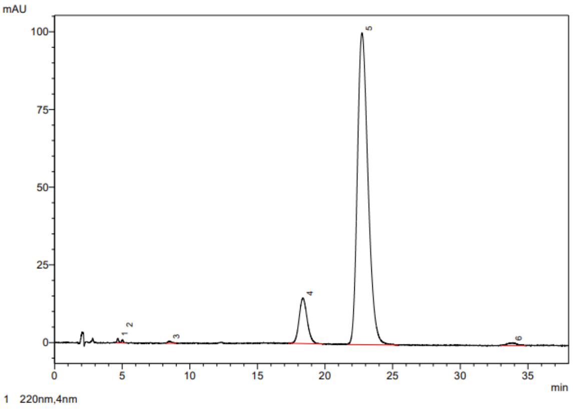

PDA Ch1 220nm

Peak #	Ret. Time	Area %	Name
1	4,68	0,21	
2	5,02	0,14	
3	8,48	0,22	
4	18,36	9,86	1. Enantiomer
5	22,73	88,88	2. Enantiomer 80.0 % ee
6	33,79	0,68	
Total		100,00	

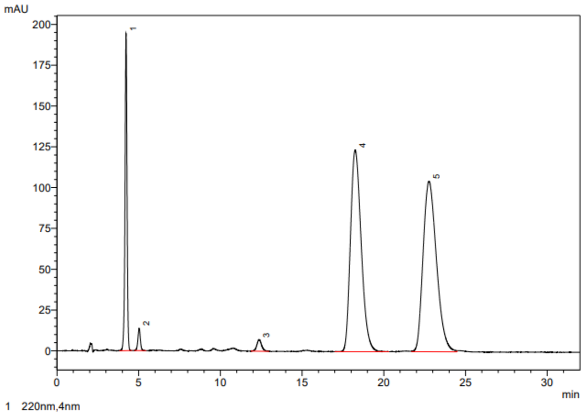

PDA Ch1 220nm

Peak #	Ret. Time	Area %	Name
1	4.23	12.40	
2	5.03	1.05	
3	12.37	1.07	
4	18.26	41.61	1. Enantiomer
5	22.78	43.88	2. Enantiomer
Total		100.00	

HPLC traces of **9f** (left) and the corresponding racemate (right).

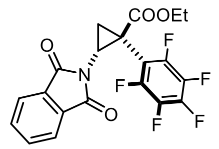

Compound 9g. Prepared according to the general procedure. The crude product was purified by flash chromatography (silica, *n*-pentane/*tert*-butyl methyl ether 50:1) to afford the title compound as a colorless oil (29 mg, 69% (contains minor unknown impurity), *trans:cis* > 20:1, 94% *ee* (*trans*)). $[\alpha]_D^{20}$ = +0.8 (c = 1.0, $CHCl_3$). ^1H NMR (400 MHz, $CDCl_3$) δ 7.80 – 7.66 (m, 4H), 4.41 (ddt, *J* = 9.6, 7.0, 1.2 Hz, 1H), 4.27 – 4.10 (m, 2H), 3.51 (tt, *J* = 6.7, 0.9 Hz, 1H), 2.22 (dd, *J* = 9.8, 6.5 Hz, 1H), 1.21 (t, *J* = 7.1 Hz, 3H). ^{13}C NMR (101 MHz, $CDCl_3$) δ 169.9, 167.9, 146.8 (dm, *J* = 250.4 Hz), 141.3 (dm, *J* = 254.9 Hz), 137.5 (dm, *J* = 252.0 Hz), 134.6, 131.1, 123.6, 108.9 (m), 62.4, 38.3, 24.2, 15.9 (t, *J* = 2.8 Hz), 14.2. ^{19}F NMR (376 MHz, $CDCl_3$) δ –139.8, –153.3 (t, *J* = 21.0 Hz), –162.0. IR (ATR): ṽ = 1716, 1523, 1498, 1383, 1271, 1215, 1158, 989, 721, 710 cm^{-1}. HRMS (EI) *m/z* calcd. for $C_{20}H_{12}F_5N_1O_4Na$ $[M+Na]^+$: calcd.: 448.05787; found: 448.05762.

The optical purity was determined by HPLC (Chiralcel OJ-3R, 4.6 mm ∅, methanol/water = 40:60, v = 1.0 mL/min, λ = 225 nm): 29.89 min (major) and 32.10 min (minor).

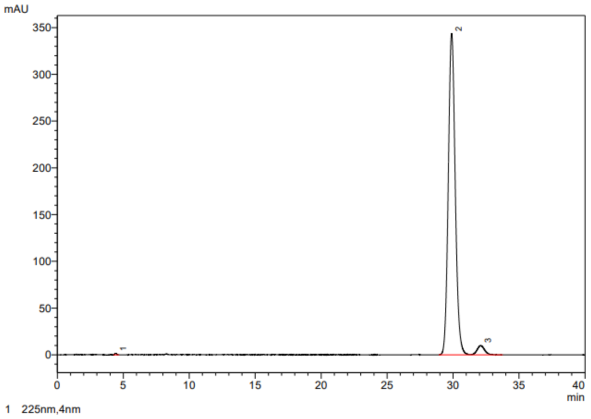

PDA Ch1 225nm

Peak #	Ret. Time	Area %	Name
1	4,45	0,11	
2	29,89	96,91	1. Enantiomer 94.0 % ee
3	32,10	2,98	2. Enantiomer
Total		100,00	

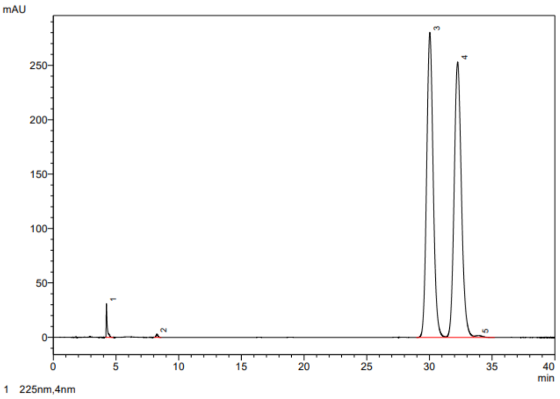

PDA Ch1 225nm

Peak #	Ret. Time	Area %	Name
1	4,24	0,89	
2	8,26	0,14	
3	30,03	49,15	1. Enantiomer
4	32,25	49,45	2. Enantiomer
5	33,92	0,36	
Total		100,00	

HPLC traces of **9g** (left) and the corresponding racemate (right).

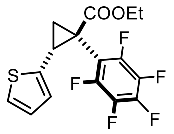

Compound 9h. Prepared according to the general procedure. The crude product was purified by flash chromatography (silica, *n*-pentane/*tert*-butyl methyl ether 50:1) to afford the title compound as a yellow oil (23 mg, 63% (contains unknown minor impurity), *trans:cis* > 20:1 (*trans*), 81% *ee*). $[\alpha]_D^{20}$ = –49.0 (c = 1.0, $CHCl_3$). ^1H NMR (400 MHz, $CDCl_3$) δ 7.02 (dd, *J* = 5.1, 1.2 Hz, 1H), 6.81 (dd, *J* = 5.1, 3.5 Hz, 1H), 6.71 (d, *J* = 3.6 Hz, 1H), 4.18 (ddq, *J* = 39.9, 10.8, 7.1 Hz, 2H), 3.43 (ddt, *J* = 9.4, 7.6, 1.8 Hz, 1H), 2.36 (dd, *J* = 9.6, 5.9 Hz, 1H), 1.95 (ddt, *J* = 7.2, 5.9, 1.2 Hz, 1H), 1.21 (t, *J* = 7.1 Hz, 3H). ^{13}C NMR (101 MHz, $CDCl_3$) δ 170.5, 147.1 (dm, *J* = 250.0 Hz), 141.0 (dm, *J* = 254.4 Hz), 139.2, 137.4 (dm, *J* = 256.9 Hz), 127.1, 126.3, 124.4, 110.0 (m), 62.2, 28.7, 28.6, 22.9 (t, *J* = 3.3 Hz), 14.2. ^{19}F NMR (376 MHz, $CDCl_3$) δ –135.7, –141.6, –154.2 (t, *J* = 21.0 Hz), –162.8, –163.2.

. IR (ATR): ṽ = 1726, 1523, 1498, 1266 ,1155, 1093, 988, 699 cm^{-1}. HRMS (EI) *m/z* calcd. for $C_{16}H_{11}F_5O_2SNa$ $[M+Na]^+$: calcd.: 385.02921; found: 385.02941.

The optical purity was determined by HPLC (Chiralpak IG-3, 4.6 mm ∅, acetonitrile/water= 45:55, v= 1.0 mL/min, λ =245 nm): 21.43 min (minor) and 23.22 min (major).

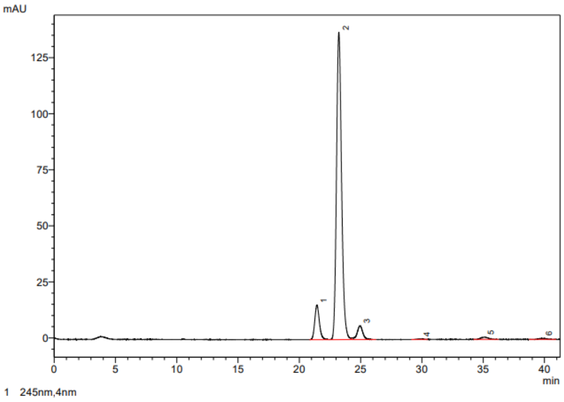

PDA Ch1 245nm

Peak #	Ret. Time	Area %	Name
1	21,43	8,78	1. Enantiomer
2	23,22	85,23	2. Enantiomer 81.3 % ee
3	24,95	4,34	
4	29,85	0,18	
5	35,07	0,95	
6	39,81	0,52	
Total		100,00	

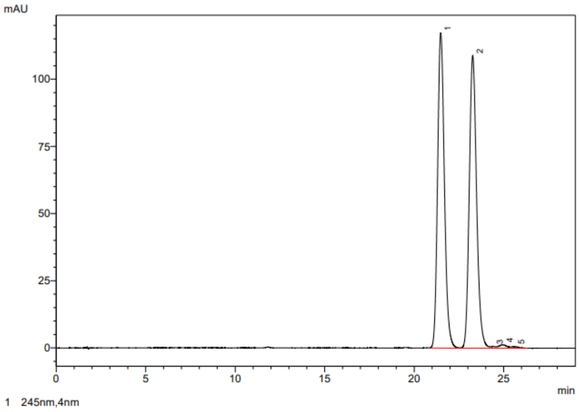

PDA Ch1 245nm

Peak #	Ret. Time	Area %	Name
1	21.48	49.47	1. Enantiomer
2	23.27	49.45	2. Enantiomer
3	24.39	0.19	
4	24.95	0.64	
5	25.59	0.26	
Total		100.00	

HPLC traces of **9h** (left) and the corresponding racemate (right).

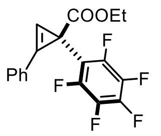

Compound 10. Prepared according to the general procedure using phenylacetylene as the substrate. The crude product was purified by flash chromatography (silica, *n*-pentane/*tert*-butyl methyl ether 50:1) to afford the title compound as a yellow oil (30 mg (contains unknown minor impurities), 86%, 96% *ee*). $[\alpha]_D^{20}$ = –25.1 (c = 1.0, $CHCl_3$). ^{1}H NMR (400 MHz, $CDCl_3$) δ 7.69 – 7.60 (m, 2H), 7.51 – 7.39 (m, 3H), 7.21 (t, *J* = 1.5 Hz, 1H), 4.19 (qd, *J* = 7.1, 3.2 Hz, 2H), 1.21 (t, *J* = 7.1 Hz, 3H). ^{13}C NMR (101 MHz, $CDCl_3$) δ 172.7, 146.2 (dm, *J* = 246.5 Hz), 140.6 (dm, *J* = 252.8 Hz), 137.6 (dm, *J* = 250.7 Hz), 130.7, 130.1 (t, *J* = 2.0 Hz), 129.0, 124.5, 117.1, 99.3 (t, *J* = 2.8 Hz), 61.9, 25.0, 14.3. ^{19}F NMR (376 MHz, $CDCl_3$) δ –141.5 (dd, *J* = 24.0, 8.1 Hz), –156.3 (t, *J* = 20.8 Hz), –162.7 (td, *J* = 22.4, 8.1 Hz). IR (ATR): ṽ = 1775, 1724, 1521, 1498, 1448, 1258, 1168, 1107, 1032, 992, 699 cm^{-1}. HRMS (EI) *m/z* calcd. for $C_{18}H_{11}O_2F_5$ $[M]^+$: calcd.: 354.06737; found: 354.06753.

The optical purity was determined by HPLC (Chiralcel OJ-3R, 4.6 mm ∅, acetonitrile/water = 50:50, v = 1.0 mL/min, λ = 254 nm): 15.06 min (minor) and 22.03 min (major).

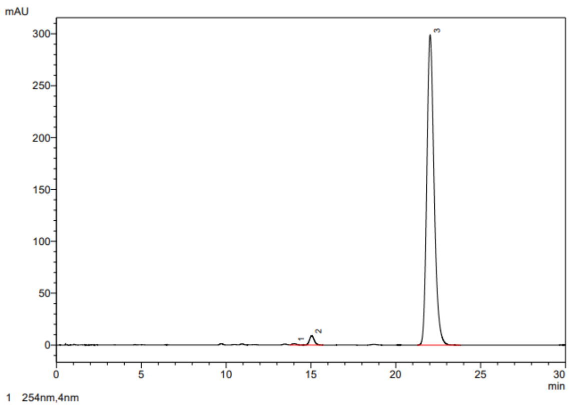

PDA Ch1 254nm

Peak #	Ret. Time	Area %	Name
1	14,02	0,26	
2	15,05	1,89	1. Enantiomer
3	22,03	97,85	2. Enantiomer 96.2 % ee
Total		100,00	

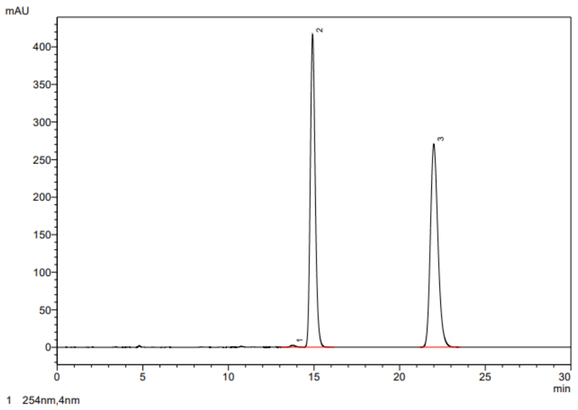

PDA Ch1 254nm

Peak #	Ret. Time	Area %	Name
1	13,76	0,35	
2	14,92	49,76	1. Enantiomer
3	22,00	49,89	2. Enantiomer
Total		100,00	

HPLC traces of **10** (left) and the corresponding racemate (right).

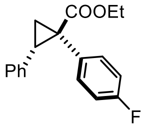

Compound 11. Prepared according to the general procedure. The crude product was purified by flash chromatography (silica, *n*-pentane/*tert*-butyl methyl ether 50:1) to afford the title compound as a colorless oil (23 mg, 79%, *trans:cis* > 20:1, 48% *ee* (*trans*)). $[\alpha]_D^{20}$ = –5.7 (c = 1.0, $CHCl_3$). ^{1}H NMR (400 MHz, $CDCl_3$) δ 7.13 – 7.03 (m, 3H), 7.01 – 6.95 (m, 2H), 6.86 – 6.73 (m, 4H), 4.23 – 4.04 (m, 2H), 3.09 (dd, *J* = 9.3, 7.3 Hz, 1H), 2.14 (dd, *J* = 9.3, 4.9 Hz, 1H), 1.84 (dd, *J* = 7.3, 5.0 Hz, 1H), 1.18 (t, *J* = 7.1 Hz, 3H). ^{13}C NMR (101 MHz, $CDCl_3$) δ 173.7, 161.8 (d, *J* = 245.6 Hz), 136.3, 133.6 (d, *J* = 8.3 Hz), 130.9 (d, *J* = 3.3 Hz), 128.2, 127.9, 126.5, 114.7 (d, *J* = 21.5 Hz), 61.5, 36.9, 33.1, 20.4, 14.3. ^{19}F NMR (282 MHz, $CDCl_3$) δ –115.4. IR (ATR): ṽ = 1712, 1512, 1250, 1219, 1158, 1088, 1023, 838, 741, 695, 550 cm^{-1}. HRMS (EI) *m/z* calcd. for $C_{18}H_{17}FO_2Na$ $[M+Na]^+$: calcd.: 307.11048; found: 307.11043.

The optical purity was determined by HPLC (Chiralcel OJ-3R, 4.6 mm ∅, acetonitrile/water= 50:50, v= 1.0 mL/min, λ =225 nm): 13.05 min (major) and 14.93 min (minor).

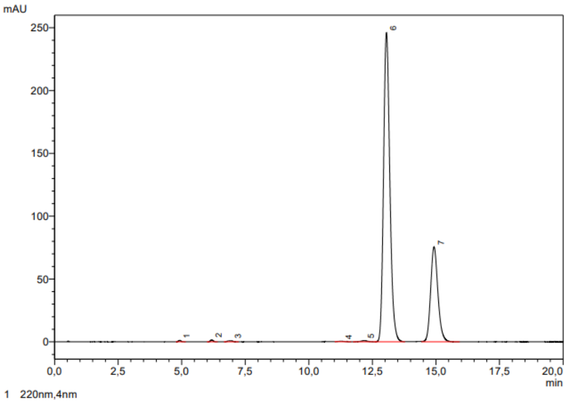

PDA Ch1 220nm

Peak #	Ret. Time	Area %	Name
1	4,92	0,13	
2	6,18	0,20	
3	6,94	0,21	
4	11,30	0,12	
5	12,17	0,30	
6	13,05	73,05	1. Enantiomer 47.5 % ee
7	14,93	26,01	2. Enantiomer
Total		100,00	

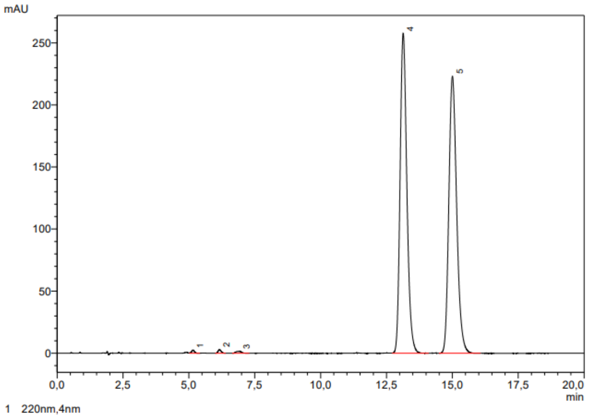

PDA Ch1 220nm

Peak #	Ret. Time	Area %	Name
1	5,15	0,20	
2	6,16	0,25	
3	6,92	0,24	
4	13,13	49,70	1. Enantiomer
5	15,00	49,62	2. Enantiomer
Total		100,00	

HPLC traces of **11** (left) and the corresponding racemate (right).

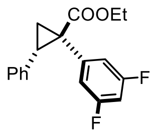

Compound 12. Prepared according to the general procedure. The crude product was purified by flash chromatography (silica, *n*-pentane/*tert*-butyl methyl ether 50:1) to afford the title compound as a colorless oil (27 mg, 89%, *trans:cis* > 20:1, 71% *ee* (*trans*)). $[\alpha]_D^{20}$ = +22.2 (c = 1.0, $CHCl_3$). ^{1}H NMR (400 MHz, $CDCl_3$) δ 7.16 – 7.05 (m, 3H), 6.87 – 6.77 (m, 2H), 6.62 – 6.52 (m, 3H), 4.15 (qq, *J* = 10.8, 7.1 Hz, 2H), 3.13 (dd, *J* = 9.4, 7.3 Hz, 1H), 2.12 (dd, *J* = 9.3, 5.2 Hz, 1H), 1.86 (dd, *J* = 7.3, 5.2 Hz, 1H), 1.20 (t, *J* = 7.1 Hz, 3H). ^{13}C NMR (101 MHz, $CDCl_3$) δ 172.7, 162.4 (dd, *J* = 247.3, 13.2 Hz), 139.1 (t, *J* = 9.7 Hz), 135.6, 128.2, 128.1, 126.9, 114.9 (dd, *J* = 18.6, 6.5 Hz), 102.8 (t, *J* = 25.4 Hz), 61.7, 37.1 (t, *J* = 2.4 Hz), 33.4, 20.0, 14.3. ^{19}F NMR (282 MHz, $CDCl_3$) δ –111.2. IR (ATR): ṽ = 1714, 1623, 1595, 1435, 1261, 1154, 1117, 1095, 985, 858, 714, 695, 684 cm^{-1}. HRMS (EI) *m/z* calcd. for $C_{18}H_{16}F_2O_2Na$ $[M+Na]^+$: calcd.: 325.10106; found: 325.10098.

The optical purity was determined by HPLC (Chiralpak IG-3, 4.6 mm ∅, acetonitrile/water = 45:55, v = 1.0 mL/min, λ = 225 nm): 23.52 min (major) and 25.55 min (minor).

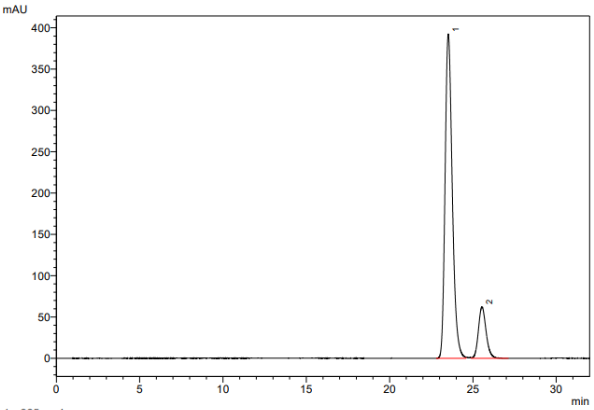

1 225nm,4nm

PDA Ch1 225nm

Peak #	Ret. Time	Area %	Name
1	23,52	85,54	1. Enantiomer 71.1 % ee
2	25,55	14,46	2. Enantiomer
Total		100,00	

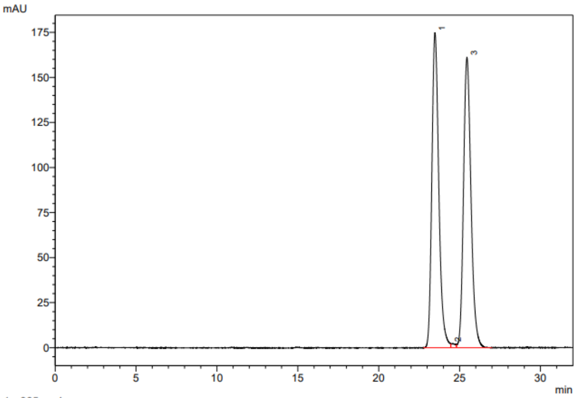

1 225nm,4nm

PDA Ch1 225nm

Peak #	Ret. Time	Area %	Name
1	23,48	49,95	1. Enantiomer
2	24,47	0,41	
3	25,45	49,64	2. Enantiomer
Total		100,00	

HPLC traces of **12** (left) and the corresponding racemate (right).

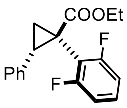

Compound 13. Prepared according to the general procedure. The crude product was purified by flash chromatography (silica, *n*-pentane/*tert*-butyl methyl ether 50:1) to afford the title compound as a colorless oil (22 mg, 72% (contains minor unknown impurity), *trans:cis* > 20:1, 94% *ee* (*trans*)). $[\alpha]_D^{20}$ = –55.3 (c = 1.0, $CHCl_3$). ^{1}H NMR (400 MHz, $CDCl_3$) δ 7.15 – 7.01 (m, 4H), 6.92 – 6.84 (m, 2H), 6.66 (s, 2H), 4.16 (ddq, *J* = 41.6, 10.8, 7.1 Hz, 2H), 3.21 (ddt, *J* = 9.3, 7.7, 1.5 Hz, 1H), 2.22 (dd, *J* = 9.5, 5.7 Hz, 1H), 2.05 (ddt, *J* = 8.0, 5.7, 1.3 Hz, 1H), 1.18 (t, *J* = 7.1 Hz, 3H). ^{13}C NMR (101 MHz, $CDCl_3$) δ 172.3, 162.9 (dd, *J* = 249.8, 7.5 Hz), 136.2, 129.4 (t, *J* = 10.6 Hz), 127.7, 127.6, 126.7, 112.1 (t, *J* = 17.5 Hz), 110.5 (t, *J* = 22.5 Hz), 61.6, 33.2, 20.8 (t, *J* = 3.2 Hz), 28.4, 14.2. ^{19}F NMR (565 MHz, $CDCl_3$) δ –107.9, –113.4. IR (ATR): ṽ = 1719, 1466, 1257, 1234, 1174, 1162, 1004, 771, 696 cm^{-1}. HRMS (EI) *m/z* calcd. for $C_{18}H_{16}O_2F_2$ $[M]^+$: calcd.: 302.11129; found: 302.11133.

The optical purity was determined by HPLC (Chiralcel OJ-3R, 4.6 mm ∅, acetonitrile/water = 50:50, v = 1.0 mL/min, λ = 220 nm): 11.67 min (major) and 15.72 min (minor).

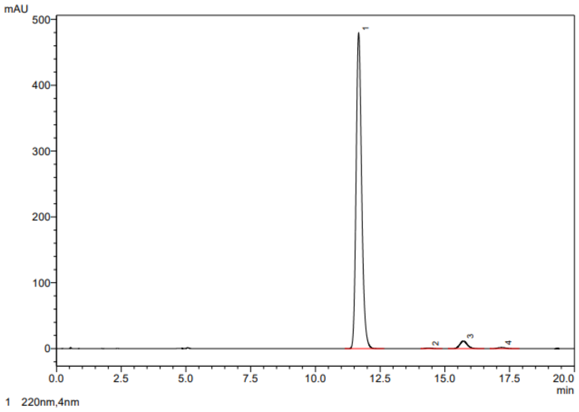

PDA Ch1 220nm

Peak #	Ret. Time	Area %	Name
1	11.67	96.12	1. Enantiomer 93.6 % ee
2	14.37	0.24	
3	15.72	3.15	2. Enantiomer
4	17.18	0.48	
Total		100.00	

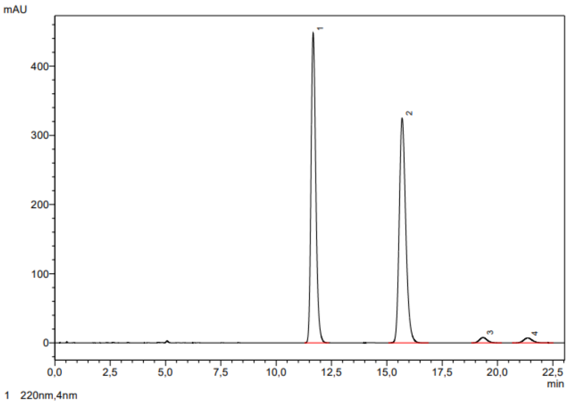

PDA Ch1 220nm

Peak #	Ret. Time	Area %	Name
1	11,67	48,60	1. Enantiomer
2	15,69	48,58	2. Enantiomer
3	19,35	1,38	
4	21,37	1,43	
Total		100,00	

HPLC traces of **13** (left) and the corresponding racemate (right).

Computational Section

Electronic Circular Dichroism

The optical properties of the two *trans*-isomers of cyclopropane **4a**, that is 1*R*,2*S*-**4a** and 1*S*,2*R*-**4a**, were investigated by simulating the electronic circular dichroism spectra at the wB97X/def2-TZVP(-f) + RIJCOSX + CPCM(heptane) (wB97X,[8] Ahlrichs BS,[9] RIJCOSX,[10],[11],[12] CPCM[13],[14]) level of theory. To account for the sensitivity of circular dichroism (CD) to geometric changes, the computed spectra were obtained as a convolution of the individual spectra of each conformer, where the contributions are weighted according to the Boltzmann distribution.

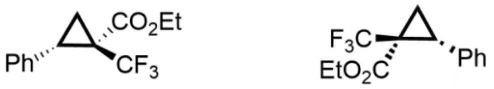

The conformers were obtained applying the following protocol. First, conformational sampling was performed via metadynamics simulations using the CREST program.[15] The simulations employed the GFN2-xTB semiempirical method[16] and were carried out without any constraint. An in-house code was then used to compare RMSDs across the conformational space obtained from the CREST calculation and select a smaller diverse subset. The geometries of the selected conformers were refined using the wB97X/def2-TZVP(-f) + RIJCOSX + CPCM(heptane) protocol.

The simulated CD spectra were initially found to be shifted to the left with respect to experimental results. This is a common situation with these predictions and is due to the expected error in the calculated excited state energies. These systematic errors can be eliminated by doing a counter-shift on the prediction. This is obtained by comparing the maximum of the simulated absorption spectrum with the experimental maximum (Figure S1). The shift between the two (in energy units) is then applied to the CD spectra. Finally, the shifted CD spectra are obtained, as shown in Figure S2.

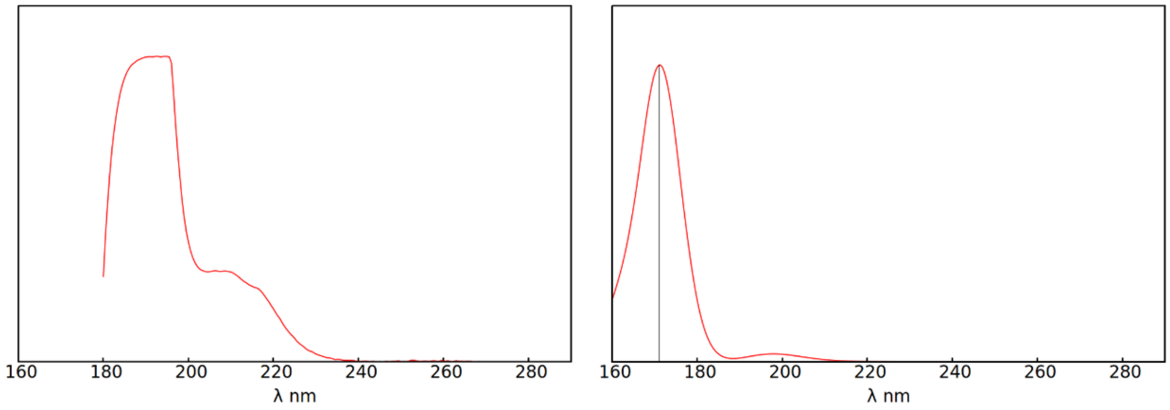

Figure S1. Left: Experimental UV-Vis spectrum of *trans*-**4a**; right: simulated UV-Vis spectrum of *trans*-**4a**, computed at the wB97X/def2-TZVP(-f) + RIJCOSX + CPCM(heptane) level of theory.

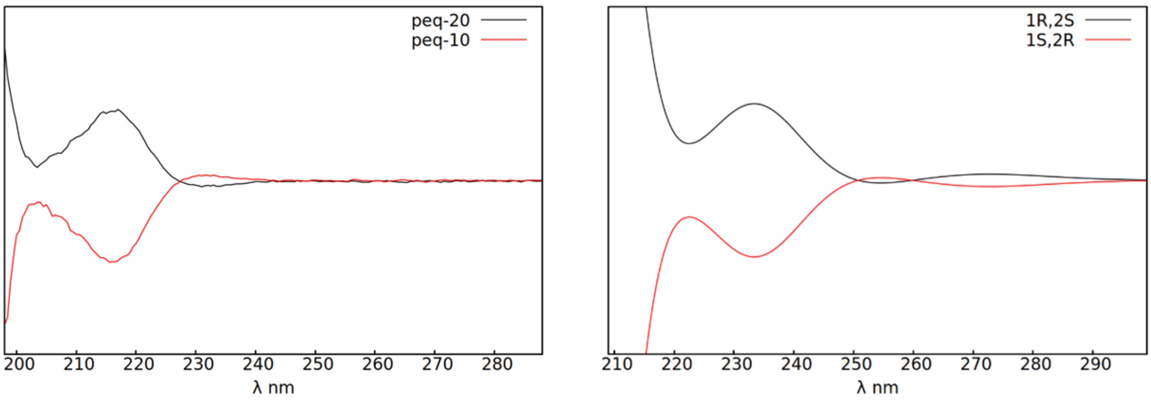

Figure S2. Left: Experimental electronic circular dichroism spectra of the two possible enantiomers of *trans*-**4a**, obtained with PEQ-10 and PEQ-20 catalysts; right: simulated CD spectra of the enantiomers, computed at the wB97X/def2-TZVP(-f) + RIJCOSX + CPCM(heptane) level of theory; the spectra are shifted according to the procedure described.

Structural Identification of the Carbene Intermediate

Conformational Sampling. Conformational sampling was performed via metadynamics simulations using the CREST program. The simulations employed the GFN2-xTB semiempirical method and were carried out without any constraint. In analogy to the conformational sampling of the product, an in-house code was used to compare RMSDs across the conformational space obtained from the CREST calculation. This approach allowed us to select a small subset of diverse conformers of carbene $\mathbf{B_{CF3}}$.

The geometries of the selected conformers were refined using the B3LYP-D3(BJ) (B3LYP,[17],[18],[19],[20] D3BJ[21],[22]) functional and Ahlrichs def2-SVP basis set with the RIJCOSX approximation. Implicit solvation effects were included by using the parameters for pentane, to reproduce experimental reaction conditions as closely as possible. Free energies were obtained by adding thermal corrections computed at B3LYP-D3BJ/def2-SVP+RIJCOSX+CPCM(pentane) level of theory to electronic energies calculated at B3LYP-D3BJ/def2-TZVP+RIJCOSX+CPCM(pentane). The same protocol was applied to the dimer model used to study the Br/F interaction.

The geometry of the most stable conformer was further double-checked by performing a constrained Global Optimizer Algorithm (GOAT) calculation with xTB.[23] This tool allowed us to explore the potential energy surface and find local minima, then it converges to the global minimum. As shown in Figure S3, the most stable conformer of carbene $\mathbf{B_{CF3}}$ found with GOAT is fundamentally identical to the one found using the CREST program.

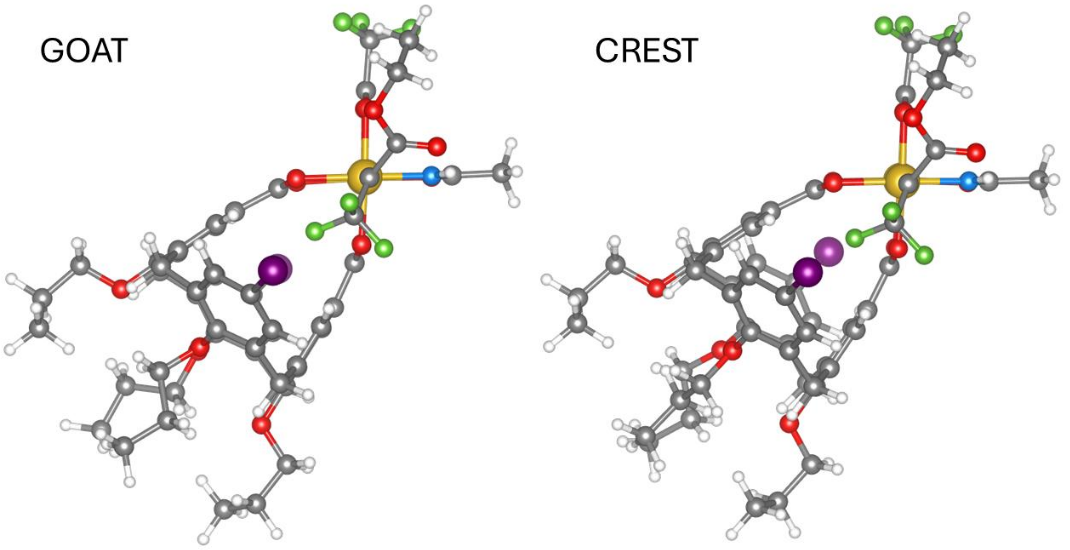

Figure S3. Structure of the most stable conformer of the carbene intermediate $\mathbf{B_{CF3}}$ as computed with GOAT and with CREST calculations; both geometries were further refined at B3LYP-D3BJ/def2-SVP+RIJCOSX+CPCM(pentane).

Results. The subset of selected carbene conformers is depicted in Figure S4. The geometry of the three most stable conformers was visually inspected in order to get first insights into the factors that might determine the stability of $\mathbf{B_{CF3}}$. As shown in Figure S5, the most stable conformer **a** displays both a hydrogen bond and a fluorine/bromine interaction, while conformers **b** and **c** exhibit either one or the other.

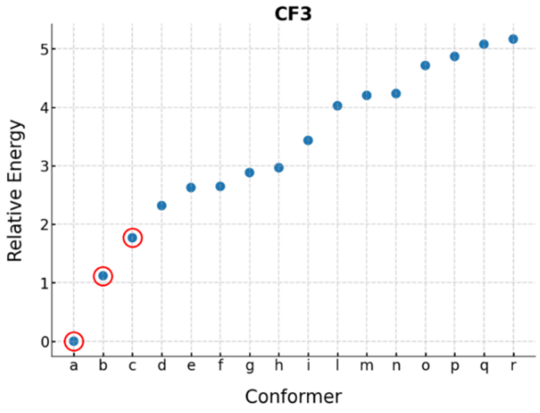

Figure S4. Subset of conformers of the carbene intermediate $\mathbf{B_{CF3}}$ selected from the CREST conformational sampling calculation; the three most stable structures are highlighted with a red circle.

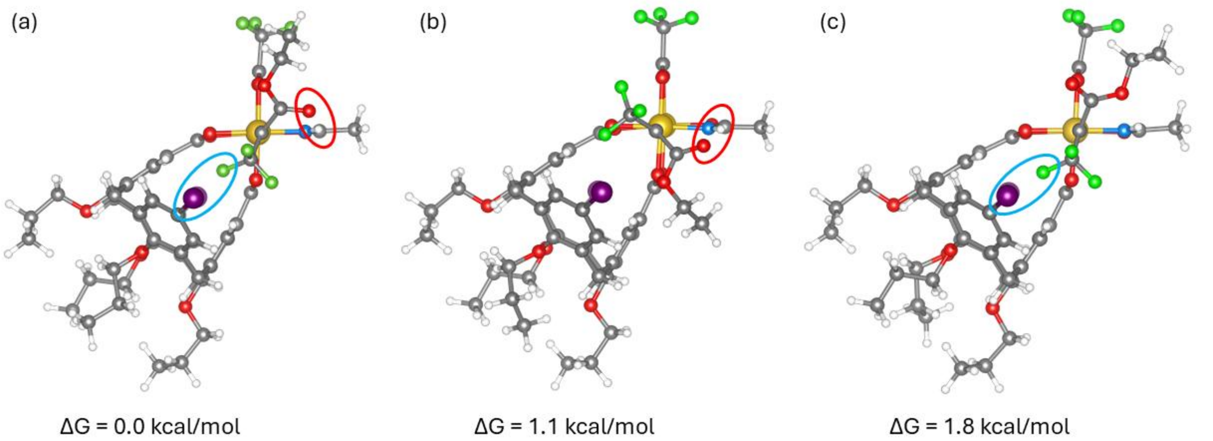

Figure S5. Three most stable conformers of the carbene intermediate $\mathbf{B_{CF3}}$; each conformer is reported with the corresponding free energy relative to the most stable conformer (a).

Fluorine/Bromine Interaction. In order to obtain insights into the nature of the fluorine/bromine interaction, calculations were run on a simpler dimer model consisting of phenyl bromide and trifluoroethane, as to isolate this interaction from all the other factors contributing to the stability of the carbene intermediate $\mathbf{B_{CF3}}$.

The energy of the system was computed by fixing the geometry of the two units as they are arranged in the most stable conformer of carbene $\mathbf{B_{CF3}}$ and only optimizing the added hydrogens. Single point electronic energies at B3LYP-D3BJ/def2-TZVP+RIJCOSX+CPCM(pentane) level of theory were performed for the dimer model, as well as for the two isolated monomers. The dimeric system was found 0.81 kcal/mol lower in energy than the two isolated units. If dispersive contributions are neglected, however, the dimer is found 1.53 kcal/mol higher in energy with respect to the two separated units, therefore indicating that the system is stabilized by dispersive rather than electrostatic interactions. This observation was further investigated by performing an Atomic Decomposition of London Dispersion energy (ADLD)[24] on the dimer model (Figure S6). ADLD calculations were performed using ORCA 6.1., the most recent version of the ORCA program package. It shows that the main dispersive contributions can be attributed to the bromine atom in the phenyl bromide and to the fluorine atoms in the trifluoroethane, as expected.

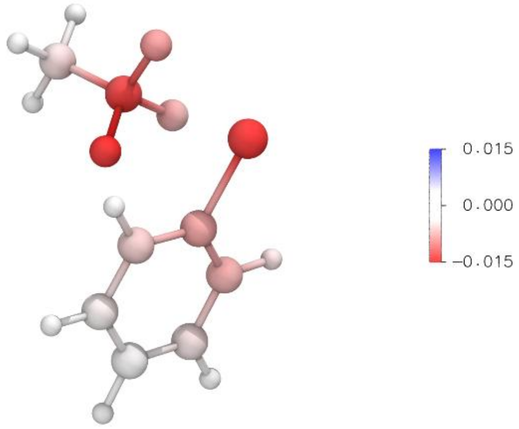

Figure S6. ADLD dispersion density difference function $\Delta\rho_{disp}$ (α = 0.5) for [$C_6H_5Br \cdots CH_3CF_3$].

On the other hand, halogen/halogen interactions via a σ-hole on the bromine atom were ruled out as a plausible explanation, since the relative orientation of the fluorine and bromine atoms do not align with the directional geometry typically required for such interactions.[25],[26] Figure S7 shows the map of electrostatic potential of the two isolated units. It is clearly seen that the most electron-dense regions on the fluorine atoms are not oriented towards the sigma hole on the bromine.

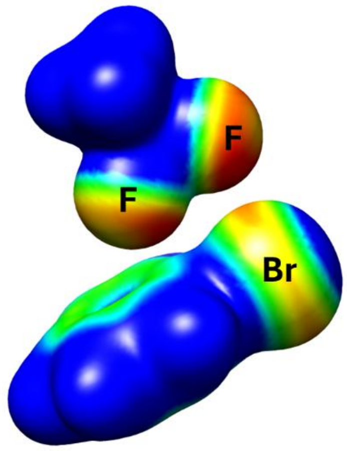

Figure S7. Map of electrostatic potential of the dimer model, displayed at an isosurface value of 0.008. The color scale ranges from −0.02 (red, electron-rich regions) to +0.02 (blue, electron-deficient regions).

Analysis of Selectivity

Reactant Complex Optimization. As a preliminary step to evaluate the interaction of the styrene with the carbene intermediate, we performed reactant complex (RC) optimizations for the four quadrants (A through D), each of them leading to one of the four possible stereoisomers (Figure S8). Among these, quadrant A is the most accessible, leading directly to the experimentally observed 1*S*,2*R* isomer without requiring any rearrangement of the carbene intermediate.

In contrast, attack from the other quadrants would require distortion of the most stable carbene geometry. Specifically, approaches via quadrants B and D (leading to the 1*R*,2*S* and 1*S*,2*S* products, respectively) are hindered by the stabilizing hydrogen bond between the catalyst's NH group and the ester carbonyl of the carbene. An approach via quadrant C, which leads to the 1*R*,2*R* product, encounters both steric hindrance and a stabilizing interaction between fluorine and bromine atoms.

These insights are supported by RC optimizations: while RCs in quadrants B, C, and D converge to local minima, optimization from quadrant A proceeds directly to the 1*S*,2*R* isomer. To further verify the barrierless nature of this pathway, a relaxed surface scan was conducted at the B3LYP-D3BJ/def2-SVP-RIJCOSX-CPCM(pentane) level, followed by single-point energy refinement using the def2-TZVP level. As shown in Figure S9, the energy continuously decreases from an initial separation of 5.53 Å to the final C–C bond length of 1.51 Å, confirming that the approach of styrene via quadrant A proceeds without an energy barrier.

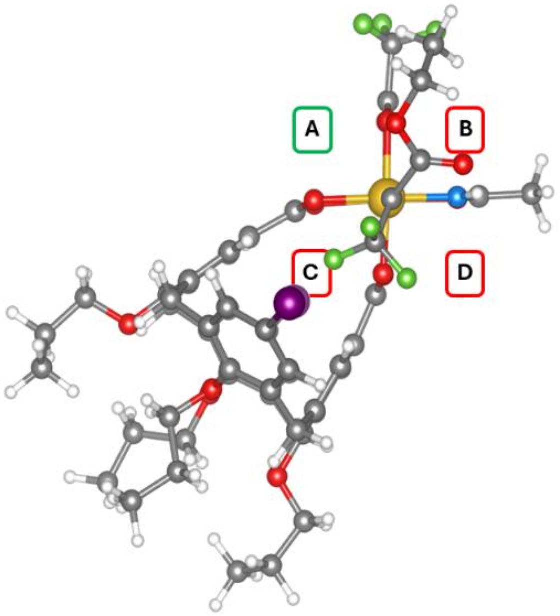

Figure S8. The most stable carbene intermediate **B$_{CF3}$**; labels for all four quadrants are specified for clarity.

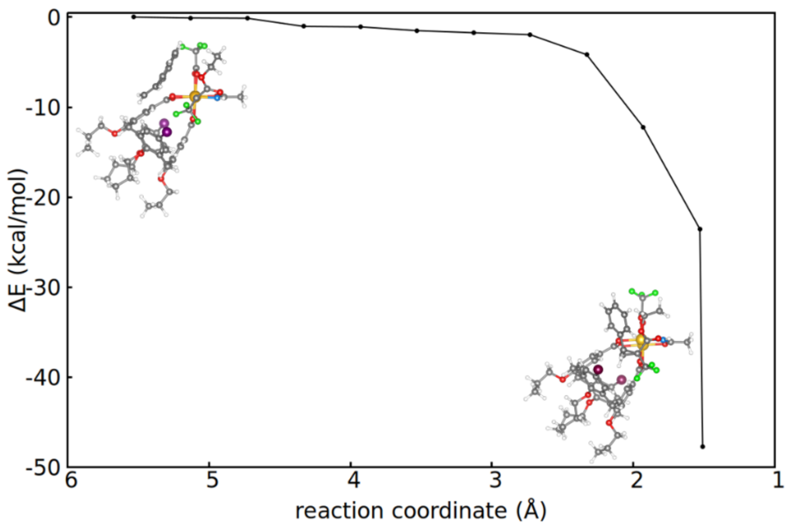

Figure S9. Potential energy surface leading to the 1*S*,2*R* isomer obtained through relaxed surface scans (attack via quadrant A); electronic energies are reported relative to the first point of the PES.

To model the formation of the other stereoisomeric products, relaxed surface scans were also conducted for carbenes **b** and **c** (see Figure S5), which exhibit different most-accessible quadrants compared to carbene **a**. The resulting potential energy surfaces were also largely flat and either barrierless or associated with only negligible energy barriers. As the 1*S*,2*R* isomer *trans*-**4a** is obtained with excellent dr and ee, these findings suggest that the high stereoselectivity of the cyclopropanation reaction is primarily governed by the structural features of the most stable carbene intermediate. Specifically, selectivity appears to arise from the energy difference between this favored conformer

and the alternative carbene conformers, which would better accommodate styrene in pathways leading to the other stereoisomers.

Stannylated Carbene $\mathbf{B_{SnMe3}}$ and Derived Reactive Complex. As for the conformational analysis of the stannylated carbene intermediate $\mathbf{B_{SnMe3}}$, we identified a different conformer from the one previously reported as the most stable intermediate structure, cf. main text of this publication.[2] This result was obtained by extending the original conformational sampling, performing multiple CREST runs from a variety of initial structures, and subsequently optimizing a broader set of conformers at the DFT level. These findings were further corroborated by GOAT calculations.[23] The new conformer of $\mathbf{B_{SnMe3}}$ identified as the most stable one, however, does not change the interpretaion of the stereochemical course of the ensuing cyclopropanation, because the derived $\mathbf{RC_{SnMe3}}$ is exactly as reported in ref.[2]

Partial Charges at the Carbene C-Atom. To gain a deeper understanding of the higher reactivity of carbene $\mathbf{B_{CF3}}$ flanked by a trifluoromethyl group with respect to the previously published carbene $\mathbf{B_{SnMe3}}$ with an adjacent trimethylstannyl substituent, we performed both Mulliken and Löwdin population analysis on the carbene C-atom of both carbenes. In line with conventional chemical logic, the results indicate that the trifluoromethyl substituent renders the carbene $\mathbf{B_{CF3}}$ more electron deficient, consistent with a barrierless process towards the preferred product (Table S1).

	Mulliken	Löwdin
$\mathbf{B_{CF3}}$	-0.235	0.030
$\mathbf{B_{SnMe3}}$	-0.368	-0.087
Δ	0.133	0.117

Table S1. Partial charge at the carbene C-atoms of the carbene intermediate $\mathbf{B_{CF3}}$ and the previously published stannylated carbene $\mathbf{B_{SnMe3}}$; both Mulliken and Löwdin population analysis data are reported.

Dispersive Effects Impacting on Diastereo- and Enantioselectivity. The reaction of allyl(dimethylphenyl)silane with 3,3,3-trifluoro-2-diazopropionate **3** catalyzed by the heterochiral-at-metal complex ***P*-1**, was notably less diastereo- and enantioselective than reactions with various styrene derivatives. The reason for this difference was investigated by ADLD analysis[24] on a representative structure along the minimum-energy path toward product formation (Figure S10). Specifically, we selected the geometry where the forming C–C bond length (3.13 Å) closely matches that observed in the transition state of the stannylated carbene (see below). Atomic dispersion energies were computed for the full structure, for the styrene fragment in its fixed geometry, and for the carbene intermediate in the same geometry. Subtracting the latter two from the complete system isolates the styrene–carbene dispersive interaction. Summing the atomic values for the benzene ring yields a stabilizing interaction of 5.1 kcal mol^{-1}. This substantial dispersive effect rationalizes the reduced stereoselectivity observed with olefins bearing aliphatic substituents, which are expected to lack analogous stabilization.

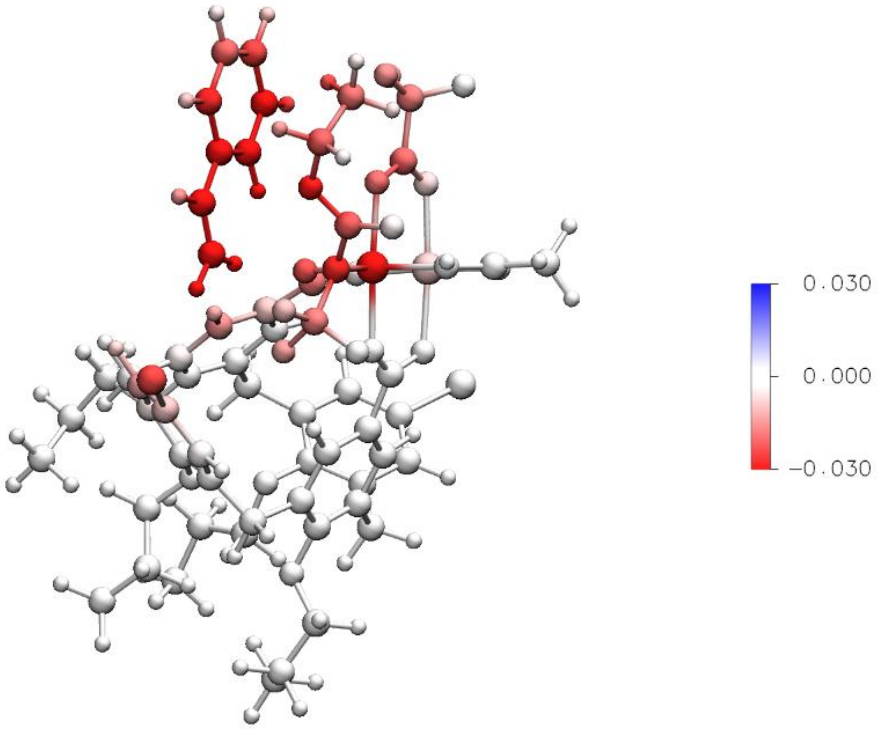

Figure S10. ADLD dispersion density difference function Δρdisp (α = 0.5) is reported in kcal·mol^{-1}·Bohr^{-3} and shows the interaction between the benzene ring (upper left corner) and the rest of the structure. Color bar range values were adjusted for visualization purposes.

The same analysis was performed for the transition state (TS) leading to the major stereoisomer in the stannylated carbene case (Figure S11). Once again, atomic dispersion energies were computed for the full TS, for the styrene fragment in the fixed TS geometry, and for the carbene intermediate in the same geometry. Subtracting the latter two from the complete system isolates the styrene–carbene

dispersive interaction. Summing the atomic values for the benzene ring yields a stabilizing interaction of 4.3 kcal·mol^{-1} in this case.

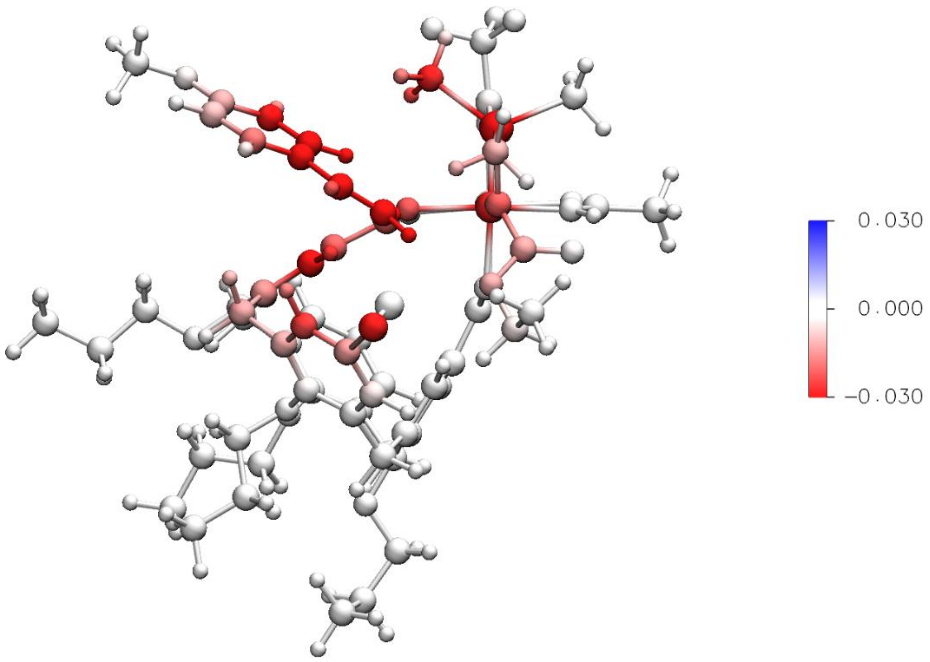

Figure S11. ADLD dispersion density difference function Δρdisp (α = 0.5) is reported in kcal·mol^{-1}·Bohr^{-3} and shows the interaction between the benzene ring (upper left corner) and the rest of the structure. Color bar range values were adjusted for visualization purposes.

Optimized Cartesian Coordinates (in Å)

1R,2S cyclopropane

C	-1.01566763683199	-0.63163622715534	-0.40652571867611
C	0.09687295940543	-1.54935790573443	0.08117910910780
C	-0.55237768814340	-1.79750374624030	-1.23325153656362
H	-0.01400587154483	-1.51840513275902	-2.12869932054857
H	-1.21745466804862	-2.64419292402121	-1.32112451806218
C	1.48969746629562	-1.02921109953889	0.23509417605436
H	-0.22231600219097	-2.20279754763409	0.88646840870703
C	1.81177980612015	-0.28697826014103	1.36890397594872
C	3.09371034057177	0.20644929390356	1.54838351455240
C	4.07268365557318	-0.04011033767889	0.59511618701008
C	3.76087937091704	-0.78369998969553	-0.53239407476392
C	2.47531519395083	-1.27568022897674	-0.71076979455975
H	2.23801805163365	-1.85426047908020	-1.59599079290362
H	1.04568437670035	-0.09056930609605	2.11241983430875
H	3.32991551821227	0.78608838669276	2.43297679820968
H	5.07546822749786	0.34670699193268	0.73195596228247
H	4.51992577342988	-0.98136629269573	-1.27994055929798
C	-0.59524196529875	0.73036241797126	-0.88326756278726
O	0.08547381374337	0.90836217500485	-1.86045273462083
O	-1.02816616137596	1.69532529845241	-0.07849787379898
C	-0.61895688201308	3.04322870373589	-0.40834355346733
C	0.77169583471909	3.32717136355545	0.11140562023846
H	-1.36485762380012	3.67223742490731	0.07388327858648
H	-0.68028860546748	3.17095645314212	-1.48830891588524
H	1.50535304154770	2.67321149545286	-0.36147874784695
H	0.81575260596452	3.18481776712141	1.19221282009534
H	1.03645916610357	4.36233320777904	-0.11138432497158
C	-2.33124375349309	-0.72655866052405	0.31740837402551
F	-2.70514275618350	-2.01023833415820	0.48914953332016
F	-2.29781293448404	-0.17690214082357	1.54316832783049
F	-3.32028306561048	-0.12011465849830	-0.36304979142381

1S,2R cyclopropane

C	-0.66867771064443	-1.05057064030986	-0.42451718098741
C	-1.58618431027979	0.07373870050054	0.08184249572208
C	-1.86246875790315	-0.59040105728535	-1.22651509060354
H	-1.60612381950700	-0.06041301645096	-2.14543273630829
H	-2.71856796292953	-1.26383324134817	-1.28555783413064
C	-1.02852342961403	1.45253942454588	0.23854128082446
H	-2.21955235862744	-0.24949479689684	0.91501390248838
C	-0.31225231998450	1.75400658112479	1.40866065157846
C	0.25469205487964	3.01618678858758	1.59001260726711
C	0.11254811722265	3.99613596509795	0.60078233742228
C	-0.60037245713982	3.70410045674537	-0.56534124383961
C	-1.16711909171490	2.43831278900324	-0.74570780812092
H	-1.71852861971857	2.21518864739663	-1.66131643729722
H	-0.18679273271017	0.98014412250625	2.17106629159027
H	0.81434103540948	3.23524597903670	2.50295749175947
H	0.55777006279888	4.98463147673033	0.73921457712152
H	-0.71481895752553	4.46425447838560	-1.34228640890937
C	0.67857459219606	-0.59983771713847	-0.92187575446665
O	0.83660715925614	0.06608337772770	-1.91815322267193
O	1.65950191170639	-0.96765838846164	-0.09454134978666
C	2.99469401461069	-0.50868565609394	-0.40361947768939
C	3.21460202936102	0.90629437364370	0.09996758570704

H	3.65504740342789	-1.22771669512738	0.10018539354245
H	3.14850332626040	-0.57572527549831	-1.49028285842972
H	2.54165379311305	1.61209939732278	-0.40821500513627
H	3.03272029271042	0.96948153877771	1.18373757393920
H	4.25444534197229	1.21224739661271	-0.09765529126596
C	-0.72292472638681	-2.35621175594662	0.32824309732795
F	-1.99864231171013	-2.75124714836942	0.52547182628713
F	-0.15522315845009	-2.27535029635078	1.54354262365368
F	-0.10126070187909	-3.34267622056774	-0.34197593648790

Most stable carbene a (CREST)

Br	0.97648318633345	-6.33423975099502	0.11389116003969
C	-0.81888904299006	-4.50410897574412	1.39279489839150
H	-0.31203580460231	-4.80774981414781	2.31061176997566
C	-0.41994806568838	-5.02988460779746	0.16371463213486
C	-0.98502028573688	-4.58527430119892	-1.03165883175948
H	-0.61342355941959	-4.95729785934505	-1.98813652767711
C	-3.48698137663425	4.02695407165836	-1.26167037049478
H	-3.34560024585150	4.49845342557493	-2.23590537105766
C	-3.18743906056773	4.72694749300731	-0.09201362463763
C	-3.27730071756700	4.11559027640864	1.15889203677823
H	-2.98390737144374	4.65893711908319	2.05893482628076
Br	-2.57972379707626	6.53530653476347	-0.21083000917956
Rh	3.22662699918657	0.09695911730631	-0.39254444361869
O	1.20733223859911	1.99992352423132	1.09272755139623
O	1.85531410674944	-0.15251609848712	1.15849788183429
O	0.98913786589023	1.90625571218733	-1.63603021172824
O	1.64959525024229	-0.24725208297772	-1.63460610391619
C	1.02229319224211	0.78182245246064	1.39524425752539
C	-0.28578259796116	0.39428102431346	1.98485145154064
C	-0.58154911749213	-0.95647956264102	2.20750715136919
H	0.18550265092724	-1.70581633375166	2.00866831002452
C	-1.28863835931110	1.35829459310750	2.14286432492054
H	-1.06364420846889	2.39608097836510	1.89427148016656
C	-0.91682180570746	-1.13147268054682	-2.19216325752748
H	-0.12627833519526	-1.86269619831660	-2.02367133520999
C	-0.59781613403130	0.23297299557430	-2.16442409927227
C	0.77612382193434	0.67066741655927	-1.81130272613669
C	-1.61669315155125	1.18626984529239	-2.28220127308021
H	-1.36357836994391	2.24288574149971	-2.18755871068155
Rh	2.52943353826940	2.45129328529076	-0.41710023022360
N	4.43190977078464	0.60494213975139	-1.92558721096744
O	3.79538446456109	2.79216629219626	-1.97780361419300
O	4.73617007144844	0.67151925995694	0.90318631807028
O	4.09242336639209	2.84112674579988	0.88230058018768
C	5.44917874930271	2.13220914913394	-3.54431264545653
C	6.05420892301011	2.23155858198285	2.09092570008336
C	4.82708545216637	1.89014172208590	1.21464051783746
H	4.86950589847642	2.51671827370656	-4.39740239415630
H	6.13635268269053	2.93041018782299	-3.22464964413583
H	6.02685981310014	1.25308078688618	-3.85927052911547
C	4.50192983802293	1.82254305134082	-2.41342276670779
H	5.04589832000181	-0.10848612490288	-2.31354617347537
F	5.93664256285912	3.42692884420316	2.65938338612064
F	6.22422066771607	1.31770012611951	3.05424587748039
F	7.15616613024171	2.23401145986957	1.32151451083134
O	-4.10305697506023	-0.73830829281764	3.19055238535729
O	-3.51180809688243	-2.28530319133985	0.26831220808773
O	-4.54459662018078	-0.97610588930775	-2.67453795798081
O	-4.52331239065221	0.81846280399569	0.18033693515563
C	-1.85612424203726	-1.35252124369805	2.61532944038373

C	-2.84145866543643	-0.36266925316333	2.80319613128838
C	-2.57514789556663	0.99708604871464	2.54408506270449
C	-2.17804187728396	-2.83377275409543	2.73853404828847
H	-3.23433703003500	-2.94966054124654	3.01106357136931
H	-1.58346267387229	-3.27982969332316	3.55085422392996
C	-1.84914967212845	-3.55706276036546	1.44508107299861
C	-2.02074273015075	-3.64320032330637	-1.00329718940371
C	-2.48557070425548	-3.18516456656723	0.24365674648534
C	-2.57268832379701	-3.03598301780592	-2.27746849784250
H	-2.14633644850090	-3.57043966965242	-3.14110561438565
H	-3.66167370261032	-3.15354916490507	-2.32034390410263
C	-2.23533968554905	-1.55509131171024	-2.36398697269430
C	-2.94585562835509	0.79744529714560	-2.45093783945822
C	-3.24155067591270	-0.57870605615508	-2.51489058048694
C	-4.04381352601332	1.85076232677703	-2.43958291946560
H	-5.01806626945362	1.35282407315325	-2.50596734938677
H	-3.94965744144980	2.50360872368890	-3.32127900594334
C	-3.92795642984956	2.69985793648029	-1.18714880017661
C	-3.72503025649500	2.79205193861970	1.25782377460856
C	-4.09762725927764	2.11279172806261	0.08290509893643
C	-3.67746099527800	2.04522895780653	2.57494464023549
H	-3.49401058823430	2.76398573647243	3.38912573163370
H	-4.63441931350996	1.55118088800191	2.77628649824551
C	-4.34719954055187	-0.68856364837656	4.59952283088827
H	-3.66464618729137	-1.39374343747144	5.11191375453587
H	-4.12066716202666	0.32504372089479	4.97735933240119
C	-5.79725608782332	-1.05006782436893	4.86851882037518
H	-6.44496399778952	-0.29747194790173	4.38647573244824
H	-5.96210862970828	-0.95274504155048	5.95552749462984
C	-6.18318327322981	-2.45272644843785	4.40065105831216
H	-6.08184622314266	-2.54843667818506	3.31028583405699
H	-5.53794564086129	-3.21785159336922	4.86458959552645
H	-7.22640498295316	-2.68662393132335	4.66523077982896
C	-4.77409594986592	-2.76016777580673	0.74694054234976
H	-5.25720233510611	-1.89533025353091	1.21835513747438
H	-4.61866632585138	-3.51710571751036	1.53415507178750
C	-5.64398123243432	-3.33823956129823	-0.35996874118555
H	-5.72511610871418	-2.59225557773807	-1.16491790845487
H	-5.14128761261312	-4.21939826250650	-0.79493174122055
C	-7.02683844272136	-3.73047876075965	0.15802514780563
H	-6.96084073051690	-4.49703948961834	0.94836352524285
H	-7.55211854770425	-2.86085975792136	0.58686058691190
H	-7.65710342931231	-4.13626162945322	-0.64878117439541
C	-4.95102655118783	-1.19314295846305	-4.02960056585593
H	-4.89064825583051	-0.23734603056392	-4.58493102515655
H	-4.25453795838420	-1.90204823279783	-4.51312703060386
C	-6.36790696809022	-1.73883020037746	-4.04609267338171
H	-6.37727478439094	-2.71365136792313	-3.52874314467208
H	-6.62698148835583	-1.94251943528652	-5.09948307511315
C	-7.40071214150408	-0.80164022681866	-3.42152111983101
H	-7.19027456667982	-0.63400166314189	-2.35566726403447
H	-7.39803604751560	0.18174999970495	-3.92139821903974
H	-8.41661437102077	-1.21874757703656	-3.50403809283292
C	-5.91215262455400	0.57096012590712	-0.05656929718381
H	-6.29483133910205	1.26298560772865	-0.82604123120578
H	-5.96558298201035	-0.44292018853499	-0.47305776381319
C	-6.75408277833479	0.68472943800458	1.20675167252038
H	-6.31774044069881	0.02928196672161	1.97670662229895
H	-6.68914609716563	1.71608637342088	1.59417525099405
C	-8.21459313401393	0.31766279637182	0.94798090840778
H	-8.66964135596886	0.97980531883421	0.19231706243256
H	-8.30657750408211	-0.71694412237507	0.57758991042604

H	-8.81611759048696	0.39710967624335	1.86693026938640
C	3.78693768678255	-1.74363256961812	-0.32780391426235
C	5.23717153160964	-2.04377841584171	-0.40394880859715
O	5.78027751123174	-2.33703920518981	0.76065107380491
C	7.23056572038058	-2.44608950046082	0.83131592551846
C	7.85426080200820	-1.08152254199700	1.04346932848737
H	7.58733122981737	-2.92512503397984	-0.09091201836144
H	7.40718263026016	-3.11801286458429	1.68131099645707
H	8.94773756107728	-1.18765196660698	1.12392530844481
H	7.47714253603659	-0.61687406692553	1.96477227894832
H	7.63313520566927	-0.41167044091879	0.19992892195863
O	5.81017548654348	-1.95762182339133	-1.47369580131556
C	2.88338680108622	-2.95008135729120	-0.17540879336748
F	3.00748259752826	-3.41379450307888	1.08050148505607
F	1.59570838005774	-2.69537137656339	-0.38856630765290
F	3.25607943442624	-3.92386145478389	-1.02687035514144

Most stable carbene (GOAT)

Br	0.91240322789441	-6.86322517868261	-0.50697306804716
C	-0.54788583850629	-4.91150047184772	0.99908701713535
H	-0.00387400193267	-5.31190804770809	1.85656970299573
C	-0.31547866424764	-5.41621559307208	-0.28166711300182
C	-0.93238466439128	-4.86357800758349	-1.40589316067991
H	-0.67911814932193	-5.22305329519459	-2.40500485005558
C	-2.04569281245810	3.85661218691012	-0.84937092900884
H	-1.86904884567995	4.28293049898774	-1.83854405313832
C	-1.44714317694445	4.42281350420425	0.27614028515904
C	-1.58423085815922	3.84114509074245	1.53814250634603
H	-1.04154023168865	4.24828289445178	2.39332463932786
Br	-0.34643521429474	5.97206253969125	0.07345760224659
Rh	3.85256547996880	-0.77027071432418	-0.89791111421509
O	2.26101329157526	1.24107142538532	0.92691667257873
O	2.61329345202884	-0.97126154101102	0.76544061583062
O	1.72984789019004	1.34731171624950	-1.77163072947804
O	2.12491071045490	-0.86313275967267	-1.97362398335490
C	1.94161268936464	0.03573283533330	1.16158465772896
C	0.65493821245216	-0.22261232824633	1.86010975863050
C	0.15724317008017	-1.52737401708474	1.97275781421066
H	0.76883863467297	-2.35803801773748	1.61808946972008
C	-0.15090813849821	0.85865660037922	2.23118908705736
H	0.22934925297642	1.86847554142959	2.07657462988493
C	-0.57436009113163	-1.39373347632793	-2.37701545862188
H	0.13105653824073	-2.22505923108574	-2.39040923711177
C	-0.08655930639500	-0.08765268463257	-2.24648578584299
C	1.35740491804559	0.16003498984091	-2.00508092770115
C	-0.98531708889223	0.98032115410333	-2.14177011910763
H	-0.58576658940371	1.97928550514305	-1.97096314954987
Rh	3.44214376623221	1.64578482680842	-0.70095170723923
N	4.96278201413833	-0.23793946142638	-2.50384646426484
O	4.54124663127123	1.99701789464403	-2.37399941675446
O	5.53596427253877	-0.49919074113580	0.26972169568040
O	5.18399153579274	1.73308169450440	0.42461942605800
C	5.98559311843490	1.28983183670742	-4.11688088448775
C	7.16201872710036	0.80438375129645	1.37902499541474
C	5.81711254318790	0.67812587109837	0.62703007172179
H	6.42726109795537	0.38103004082441	-4.54692614651628
H	5.38302977605392	1.80385269106355	-4.88118512306949
H	6.78641373188983	1.97905085331665	-3.80769775389688
C	5.10919400952546	0.99701894356949	-2.92589249756696
H	5.49287798997954	-0.96735021876391	-2.97726447339446
F	7.27426779573233	-0.14075014734345	2.32073517072138

F	8.17022904533284	0.64169362480602	0.50470648181380
F	7.29645887858544	1.99326996974111	1.95684317033900
O	-3.19743025795245	-0.87382300889642	3.29541235227916
O	-3.12833654697399	-2.43915431689195	0.23304187777286
O	-4.18485547224792	-0.78721081816658	-2.34902755517822
O	-3.90952268020099	1.12871078306895	0.73198486983283
C	-1.12678712618612	-1.76419389691088	2.46838383887814
C	-1.92060583424487	-0.65827327323471	2.84420036992026
C	-1.44400543166727	0.66288764976092	2.71759695525218
C	-1.67066945158400	-3.18651007668093	2.51090377701445
H	-2.73331120402906	-3.15025786003242	2.78012255297996
H	-1.15227171442560	-3.76138924103549	3.29492860555322
C	-1.47517511283042	-3.87724293953975	1.17707337877335
C	-1.86326384310899	-3.82982913437629	-1.24730277250121
C	-2.16690290708204	-3.39520958001241	0.05396559990431
C	-2.44595800275746	-3.07954479291080	-2.42966230449837
H	-2.14015348814223	-3.58140699701861	-3.36075631167802
H	-3.54288017154690	-3.08008690957042	-2.39788865736973
C	-1.94748382920622	-1.64166912305154	-2.42209738488075
C	-2.36412821226002	0.77110020011171	-2.17624686331359
C	-2.83606386634762	-0.54922153900128	-2.33604546359902
C	-3.31553852773927	1.93822810140152	-1.92598076179233
H	-4.32560597539481	1.54499863494409	-1.77035480238342
H	-3.33912736939396	2.60204523542522	-2.80525700831142
C	-2.85190810200586	2.71760908243988	-0.71407549218870
C	-2.38246431122429	2.70202983242839	1.69317664121876
C	-3.07185376059680	2.19840355963587	0.57306909549060
C	-2.33333268596847	1.87686741387152	2.96543828819606
H	-1.91722049327861	2.48828164517593	3.78115883434026
H	-3.32990731390393	1.54198696789554	3.27420742202412
C	-3.33990584480662	-0.97439608238809	4.71511874010063
H	-2.64309546882719	-1.74295167454667	5.10044116965615
H	-3.06269321129800	-0.01134889864737	5.18235056413212
C	-4.77758104207369	-1.33992193594229	5.03911890175673
H	-5.43905555521546	-0.56386297648474	4.61534604112193
H	-4.89501046475131	-1.28627033156249	6.13526337009783
C	-5.19053708739022	-2.72111566121855	4.53159929935778
H	-4.54964985121674	-3.50909106381782	4.96135373002528
H	-6.23333013385306	-2.94990207893775	4.80216855575117
H	-5.10616805878301	-2.78142275075452	3.43728547169694
C	-4.44307030718988	-2.98010137914024	0.39899867412323
H	-4.43670238808909	-3.71675530379930	1.22478142813336
H	-4.73933071557766	-3.52404331181763	-0.51737177454001
C	-5.38936041389716	-1.83966564909438	0.69509427491881
H	-5.00855377804112	-1.33294632155758	1.59254345386308
H	-5.31190268790961	-1.12226611424395	-0.13358534304385
C	-6.83396178174070	-2.28905314133581	0.89547645647348
H	-6.93105865306109	-2.98299815939668	1.74699469992112
H	-7.48687199822648	-1.42468667438079	1.09616731856743
H	-7.22920744495561	-2.80022167214489	0.00205511756164
C	-4.83857893890315	-0.57801313860716	-3.60417498048877
H	-4.68551759820667	0.46495439345952	-3.93545177522674
H	-4.38353714918653	-1.23993170831762	-4.36586035399504
C	-6.31699098846237	-0.87947254437376	-3.43921410488652
H	-6.81924330512185	-0.61093345044305	-4.38453723397234
H	-6.72544345870483	-0.20670847121785	-2.66492270590811
C	-6.60669258819420	-2.33627719902869	-3.08067475501092
H	-6.12138640368226	-2.61054731521828	-2.13336213774272
H	-7.68799146678825	-2.51386294422035	-2.97104316020051
H	-6.22892648652280	-3.01873252822422	-3.86046125461269
C	-5.17101436920097	1.43625240596832	1.34158497837437
H	-5.42883142810961	0.57933971331976	1.97866895464329

H	-5.06140310422834	2.31734772217043	1.99729823493017
C	-6.26191826364478	1.69152858445053	0.30975885207453
H	-5.98134808666593	2.56554026935850	-0.30255114475046
H	-6.30848825508164	0.82787551377461	-0.37297686288999
C	-7.62268934851296	1.92239178103800	0.96557619735424
H	-7.93340954822654	1.04448813911457	1.55684719822373
H	-7.59985124584405	2.78970007559559	1.64654168788452
H	-8.40320680638260	2.11077018592902	0.21210679863333
C	4.21354007527289	-2.63617712881234	-1.19253588813944
C	5.62238716250407	-3.07833191722588	-1.30117454763874
O	6.09262352008225	-3.62483106951198	-0.20088833075699
C	7.51055287895416	-3.95524643733508	-0.15149748705300
C	8.33034660060488	-2.74071872900648	0.23421898111254
H	7.80596337305472	-4.35874594412250	-1.13010443819445
H	7.57143673094777	-4.75095953326335	0.60210317006477
H	9.39127314738400	-3.02762865350059	0.31158638845179
H	8.00453584978860	-2.33668823584124	1.20280978138406
H	8.24094329200139	-1.94663511153318	-0.52040291922761
O	6.23346933701750	-2.87114881238032	-2.33372128297046
C	3.16519434276911	-3.65824270223245	-1.58787194316784
F	3.60833510243269	-4.91610493116696	-1.39728658311611
F	2.04525229033294	-3.51573032643005	-0.87813677667651
F	2.86856844715814	-3.52520168041549	-2.88983106177658

Carbene b

Br	1.74888802357405	-5.83343980586334	0.61725561931322
C	-0.32204794408754	-4.20169599525516	1.75398127291901
H	0.22455156404482	-4.33152366045769	2.68960384810734
C	0.17064409221389	-4.75652429044966	0.57238222044243
C	-0.45424482011011	-4.50877782986004	-0.64871403648034
H	-0.02641117325242	-4.89083288365048	-1.57675584071134
C	-3.58621035610643	3.76709474056719	-1.68488499950968
H	-3.45561325135260	4.18613192157092	-2.68434537035469
C	-3.43848727688284	4.57842180747970	-0.55913833799716
C	-3.52289600115331	4.04892962402698	0.72909274891664
H	-3.35358198831105	4.68739407414686	1.59807051815577
Br	-3.05731989770957	6.43734895581767	-0.78717260082267
Rh	3.37739965246756	0.24457548244214	-0.09272889195933
O	1.19771568303655	2.22840303980347	1.02959904252442
O	1.99900397060287	0.17249473366345	1.46455343706861
O	1.15980275025999	1.81965011762289	-1.67797214777487
O	1.83891250828178	-0.29819158157390	-1.32191014388934
C	1.08240452095942	1.05700073550452	1.50094149821203
C	-0.23512986516890	0.63601821205836	2.04300306003143
C	-0.44269205747439	-0.70860572193456	2.37309616269440
H	0.39466513821874	-1.40317246465932	2.30251022943902
C	-1.31715362122801	1.52432122248803	2.04176294932551
H	-1.15615746621120	2.55135273895945	1.71098663706653
C	-0.63078002889376	-1.32341390876376	-1.98310765396181
H	0.16729187837334	-1.99348889316636	-1.66434395115783
C	-0.36174468708910	0.04831119095314	-2.07783819102934
C	0.97287885810681	0.56944462921922	-1.68942260515859
C	-1.40223668157111	0.94202013330077	-2.36052376887764
H	-1.19925186517570	2.01371337622360	-2.34120910934789
Rh	2.61107586420324	2.55204730425146	-0.43671415637729
N	4.58861464169245	0.55201007395410	-1.67200768484523
O	3.96051071227825	2.72433438329480	-1.95848532215389
O	4.81175318434023	1.02545024023894	1.16184814601173
O	4.05940197461662	3.14500734643821	0.90894625198346
C	5.61917862557694	1.89942921780930	-3.43793634393540
C	5.92205546713946	2.73280480919322	2.34864079390083

C	4.81032786230577	2.26694321311920	1.38091242724249
H	6.32874871865097	2.70125643699527	-3.18201585332763
H	6.17346219773918	0.98212294367009	-3.67684073594994
H	5.05202195453559	2.22965575911213	-4.32141170526202
C	4.66397093544620	1.71137370779125	-2.28694782858505
H	5.19625295736616	-0.19997292607099	-1.98966438534020
F	5.90910188467725	1.99666610210829	3.46418208093663
F	7.11745489531522	2.58101666398344	1.75929610757582
F	5.77798528854416	4.01233865475400	2.68348751967183
O	-4.02399523849745	-0.68834686484049	3.13962514288063
O	-3.37474947656981	-2.60455980956848	0.46794823927837
O	-4.18630582080946	-1.38655555569021	-2.84370629176733
O	-4.26854853871005	0.55497277382851	-0.05008087393983
C	-1.70873058754742	-1.17186125154298	2.73300173103920
C	-2.77568288841233	-0.25339362289219	2.77072083259642
C	-2.59542398719145	1.09593853470305	2.39992218461450
C	-1.91996978199855	-2.65937002490302	2.96909625991464
H	-2.96828753427237	-2.83557729433197	3.23546932428692
H	-1.30785782492253	-2.99215887689095	3.82132290177008
C	-1.50081198816967	-3.44317291441017	1.73672248051255
C	-1.63281340400741	-3.75405395392357	-0.69201568479783
C	-2.19045758830130	-3.28976120362497	0.51493144194818
C	-2.20893080804755	-3.30207684909111	-2.01606480518157
H	-1.76438653153706	-3.90023408943871	-2.82777176115834
H	-3.29281944998012	-3.45233785262470	-2.05089340081515
C	-1.91079384148299	-1.82349178247450	-2.22966710555047
C	-2.69634768103636	0.47799673604530	-2.59489928107654
C	-2.92794415556419	-0.91224900592072	-2.57425759078233
C	-3.84028632199032	1.47060088108761	-2.72973232588915
H	-4.78355848693290	0.92005617017154	-2.82876780672855
H	-3.71804733639220	2.07190084267053	-3.64387119133090
C	-3.86400958362297	2.40311537536895	-1.53179554240081
C	-3.80751368026989	2.68927440942352	0.90674399102117
C	-4.02223118489251	1.88558039444808	-0.22946750859680
C	-3.77023395860255	2.05403738427493	2.28201791827898
H	-3.68635315002714	2.84865956568043	3.04011675331190
H	-4.69860550428143	1.50664711172384	2.47956348226667
C	-4.33059827967568	-0.51307729395355	4.52765963027949
H	-3.67190702686630	-1.16900073356018	5.12927633787308
H	-4.11637924084157	0.52987332225074	4.82283234686718
C	-5.79163145913876	-0.84614795501683	4.77250493916019
H	-6.41846051378765	-0.13028090356999	4.21319083814890
H	-5.99065787655966	-0.66382221556442	5.84278997839525
C	-6.17323645839067	-2.27850184711220	4.40304492204854
H	-6.04517654640871	-2.45435438691187	3.32589880688763
H	-5.54353190146903	-3.00905656732740	4.93860728148552
H	-7.22400094704468	-2.48784452232617	4.65790852878291
C	-4.50343120065836	-3.27311621445102	1.05499525872480
H	-5.22920781515282	-2.47800077860739	1.27057644750070
H	-4.20888712750599	-3.72220029455858	2.01512014335787
C	-5.10823697015297	-4.34737919734942	0.15745159140213
H	-4.29928314622924	-5.01956032193985	-0.17885881151840
H	-5.76940902845046	-4.96613009728787	0.78956876576645
C	-5.89269422235111	-3.80400943314900	-1.03460600750690
H	-6.23444593001213	-4.61720394700280	-1.69482291852516
H	-6.78544465656947	-3.25148133598918	-0.69831577748669
H	-5.28542280548904	-3.11079183094415	-1.63205827593687
C	-4.46463776769919	-1.57673971599812	-4.23666762333346
H	-4.32248840304334	-0.61686528888980	-4.76831074127889
H	-3.74056551454864	-2.29888636729523	-4.65669838567129
C	-5.88771373271680	-2.07738607281999	-4.40802421389263
H	-5.97612203848067	-3.07578807303604	-3.94863744187041

H	-6.05174829902227	-2.21773560875692	-5.49056162088810
C	-6.94639340185746	-1.13913616118859	-3.83029800938326
H	-7.96158844332782	-1.51379884214780	-4.03481686773342
H	-6.83510683329005	-1.04276457423951	-2.74092149934769
H	-6.86549432165309	-0.12910133169021	-4.26638371544358
C	-5.59453680808393	0.08199477359454	-0.30523944053568
H	-6.02832415443861	0.61699260377385	-1.16692363446570
H	-5.47730166052968	-0.96925990240204	-0.59508047945748
C	-6.51115469177774	0.21263110958062	0.90339291559075
H	-6.02027790155677	-0.27114751943099	1.76127286383690
H	-6.62536839885497	1.28005505159085	1.16018213651140
C	-7.88336905790019	-0.40854266013408	0.64578000639550
H	-8.38656412232381	0.06184913438193	-0.21587885135539
H	-7.79903179643942	-1.48654213709233	0.42991497341500
H	-8.54366874403416	-0.29526388690563	1.51978665859564
C	3.80679191368351	-1.61411258754756	0.15725942756299
C	3.94651184725053	-2.47214196538570	-1.03937761132661
O	2.86045833214494	-3.14354105791042	-1.36216096114256
C	2.86047829402154	-3.87124091107587	-2.62013606497986
C	2.46821029344989	-2.95696626077380	-3.76422798950750
H	2.13575923473758	-4.67909378225991	-2.46005464308445
H	3.85695961627705	-4.30960046178159	-2.76770634051933
H	1.47412383362276	-2.51798088332410	-3.59651126106997
H	2.44492839057114	-3.53303840703948	-4.70278377788893
H	3.19498016973164	-2.13874219406423	-3.87810338351688
O	5.00185137820066	-2.45703141208114	-1.64500944053488
C	3.87758952744076	-2.31283398159137	1.49915736768085
F	2.64485867372348	-2.76548658463328	1.79560938284566
F	4.70954945954807	-3.37034846848347	1.46111574632221
F	4.27983592341018	-1.51871583098565	2.48638534842988

Carbene c

Br	0.98809637052530	-6.30757828535346	-0.06273843003960
C	-0.79411620730640	-4.49793157087785	1.26443682879071
H	-0.27229024624500	-4.80725452664902	2.17183110842068
C	-0.41784274018767	-5.01727830131916	0.02554059696587
C	-1.01742739045349	-4.57671119969284	-1.15481517163876
H	-0.65839142579724	-4.93695681112970	-2.12067059160275
C	-3.45668106799023	4.02717715620692	-1.08871080759703
H	-3.28541043489646	4.50817114448675	-2.05339510552062
C	-3.18578213703953	4.71099247756307	0.09723485807067
C	-3.32459129799012	4.08570453063099	1.33646659423020
H	-3.03541035862858	4.60893501695220	2.24987213068896
Br	-2.54563636089276	6.51042896634585	0.01954035422690
Rh	3.19697088709259	0.12866854437951	-0.37250454030485
O	1.17145294818098	2.01138339707682	1.13086567804491
O	1.83944230837214	-0.13163496412388	1.16887086736405
O	0.96049089147166	1.93060258968770	-1.60016385738664
O	1.61109631418012	-0.22910381123664	-1.59587459849726
C	0.99322436970368	0.78914252964150	1.41780528274447
C	-0.30976384829131	0.37859768473455	1.99983697200300
C	-0.59895173130263	-0.98168463209912	2.17271342000444
H	0.16695790916427	-1.71985169146709	1.93095927301217
C	-1.31315949212079	1.33248017149182	2.20636382084959
H	-1.09538724545362	2.38044313401884	1.99654188587259
C	-0.94342484718940	-1.09797583034284	-2.22657843415759
H	-0.14942907062161	-1.83541841496534	-2.10563946561411
C	-0.62758534682024	0.26434848039442	-2.14090670869618
C	0.74598119420610	0.69537038190905	-1.77891847575194
C	-1.64953764996222	1.21938621991795	-2.20588140665010
H	-1.39583593096306	2.27063161584597	-2.06636485788419

Rh	2.49115614988863	2.48723077643746	-0.36881607349477
N	4.40555331484433	0.72483283013115	-1.89198192687606
O	3.73591106714005	2.90278132195856	-1.92154926957629
O	4.71521838786599	0.65648082916521	0.92348292743897
O	4.08584953335195	2.83150140736917	0.92369197518935
C	5.49416147391198	2.31413998101503	-3.39934657473109
C	6.10052485860252	2.23481864679208	2.02638855264755
C	4.82708322044615	1.87487740611861	1.22721047148290
H	4.98438783691278	2.83338466376914	-4.22436341835397
H	6.22294228425521	3.01636176543896	-2.96494786396142
H	6.02299191349271	1.43356842085902	-3.78784220229095
C	4.47888757811588	1.95746774823913	-2.34317381361741
H	5.09794855145628	0.07118301965445	-2.24253481118940
F	7.01991814962899	2.71698111814812	1.16775489597321
F	5.85459733991093	3.16913266574846	2.94158401941479
F	6.62512363301207	1.16865900850235	2.63261702969473
O	-4.09865468731421	-0.80972910494061	3.23780021718876
O	-3.51786728115684	-2.28502860463622	0.23769173706719
O	-4.57994300710819	-0.93010161276067	-2.63855386599482
O	-4.61389953449647	0.82853612707356	0.23376418708416
C	-1.86527387329644	-1.39818900906825	2.58358923439197
C	-2.84810748923047	-0.41770598960101	2.83076252672876
C	-2.59164256704858	0.95082659866816	2.61683129472903
C	-2.18820003107935	-2.88290325670552	2.64783433834804
H	-3.25398764780842	-3.00658314973848	2.87261001082839
H	-1.61951235129278	-3.35757453702975	3.46290380680692
C	-1.83659525388052	-3.56593890845271	1.34167360187353
C	-2.05508409512935	-3.63798486494555	-1.10290684799930
C	-2.49722931520686	-3.18844142479554	0.15776259776132
C	-2.59282068181709	-3.00028240320460	-2.37111635254677
H	-2.13579843847108	-3.50173848915745	-3.23841127333835
H	-3.67861610577145	-3.12739790527095	-2.46100882313843
C	-2.26409650156678	-1.51535923461564	-2.39935688191531
C	-2.98008358754484	0.83665603947018	-2.37257293378363
C	-3.27461573245602	-0.53687356830898	-2.49057686496312
C	-4.08060974282312	1.88415216973640	-2.30025567970441
H	-5.05234839344270	1.37813479721760	-2.32017436821012
H	-4.02897773441718	2.54956215926915	-3.17652772081350
C	-3.93216083958519	2.71169773484997	-1.04022037303849
C	-3.78665574413239	2.76479850008312	1.40937502514432
C	-4.15325044766346	2.11109726269498	0.21481668329693
C	-3.69447725393191	1.99550152548748	2.71474924839346
H	-3.46784037087234	2.70417884240383	3.52642073788130
H	-4.63682415487451	1.49957675876949	2.97375549744873
C	-4.26020153993123	-0.94557560898363	4.65342905845947
H	-3.46311579175798	-1.59809370106777	5.05319310073519
H	-4.14570949043676	0.04761216166660	5.12918090114815
C	-5.62955544455181	-1.53019002971508	4.95077403905818
H	-5.69939006594037	-1.65219182683833	6.04557080894635
H	-5.68473259773936	-2.54517907955755	4.52072749569472
C	-6.79273519783631	-0.68001182980507	4.44165983381369
H	-7.76042627578037	-1.10715019487057	4.74823551250517
H	-6.73690256716707	0.34833399912408	4.83692719973730
H	-6.78578773381573	-0.61545250114080	3.34413840984722
C	-4.84144967134167	-2.77520141749428	0.00147917163906
H	-4.81942874154348	-3.57489190435856	-0.75765363347272
H	-5.39379365767432	-1.93250862960576	-0.42997726179106
C	-5.51768549665798	-3.27938531237438	1.26878026407128
H	-4.97958491008516	-4.16825492919289	1.64124023953450
H	-5.42198837389146	-2.50365691937322	2.04303488397606
C	-6.98824056027235	-3.61686381406406	1.02523043321699
H	-7.54571020951759	-2.72945323404289	0.68116571328452

H	-7.10439841533092	-4.39829158781579	0.25524212107328
H	-7.47355425240958	-3.98032710333990	1.94459623874962
C	-5.05583979431871	-0.98001600008590	-3.98665938636833
H	-4.83896108216493	-0.02066549803414	-4.49050983091830
H	-4.51294046957093	-1.77390285975027	-4.53537577058881
C	-6.54897632738559	-1.25480987797763	-3.97856463592306
H	-6.88849760827507	-1.24531798445586	-5.02873501711716
H	-7.06076913502714	-0.41461874380335	-3.47770173518294
C	-6.93221321955098	-2.57611243517367	-3.31312307691854
H	-8.01530522603896	-2.76024532211310	-3.39092443460235
H	-6.41034405611579	-3.42502593363073	-3.78629348079695
H	-6.66637450181917	-2.57443136723048	-2.24647585944454
C	-5.89294985835089	0.55463580116474	0.82317047955188
H	-5.76461671462018	-0.37962404264822	1.38200743653737
H	-6.15282251575855	1.34429928251680	1.54731254447684
C	-6.99303829768211	0.42095447661992	-0.22368057608162
H	-6.61228673874757	-0.21790901524615	-1.03635206576921
H	-7.82862074034918	-0.12937852498933	0.24499408468329
C	-7.50774766550090	1.74885375025732	-0.78020726672471
H	-6.70461776054334	2.33701629463860	-1.24811689791874
H	-8.28862910399098	1.58582402876132	-1.54043032962184
H	-7.94303745736170	2.37028612991220	0.02021150738843
C	3.82511876265905	-1.68802382191641	-0.51088284148064
C	5.20586467586510	-1.96169773049452	-0.06078502876302
O	6.13889236615785	-1.39292152609977	-0.81058571213810
C	7.46467130201141	-1.20733163325507	-0.24431636700914
C	8.06355497699274	0.02153580198514	-0.89184411692405
H	8.05090843422748	-2.11790758187150	-0.44252892123915
H	7.35577768479506	-1.09075201107284	0.84289010279773
H	7.44736683522132	0.90601459592857	-0.67624750319204
H	8.14233796955351	-0.10119470872860	-1.98329396391341
H	9.07334340256621	0.19684829635011	-0.49010372474272
O	5.37310546586435	-2.56074105031186	0.97717253101972
C	3.01395565808884	-2.84274480406216	-1.06273849368333
F	1.79147073939381	-2.89291936670122	-0.52557622472684
F	2.89032486750497	-2.68203670082566	-2.39208158750127
F	3.59583645049070	-4.03397053307213	-0.85218341961571

Dimer model

Br	0.95750387725115	-6.36180219295252	0.16498593591819
C	-0.81603989429859	-4.50093644742276	1.43201745408120
H	-0.30945402165355	-4.79910559933211	2.35180829802600
C	-0.42406178309803	-5.04239185722726	0.20737399238488
C	-0.99126324505458	-4.60720205600116	-0.99056530133008
H	-0.62485054458234	-4.99083943640015	-1.94449535948843
C	-1.84040290751644	-3.54695921812079	1.47777944108040
C	-2.02397845422256	-3.66195708912418	-0.96828457554930
C	-2.48127882760075	-3.18722201272508	0.27522898309678
C	3.82118889559824	-1.72406934304475	-0.42636965348816
C	2.92514450764266	-2.90157254739402	-0.75976744866506
F	3.48209188536015	-4.06668575715318	-0.37735074085347
F	1.73846329123857	-2.80929110839000	-0.15847205128049
F	2.73005894191860	-2.95882151442113	-2.08650283360931
H	-2.48620864961507	-3.32773132000579	-1.89937912189920
H	-3.30442775565416	-2.46746042811534	0.30290948877343
H	-2.16074029592499	-3.12446522305744	2.43227956250411
H	4.78227121487107	-1.84489483751012	-0.94411991179335
H	3.99005952305282	-1.68166175301700	0.65847612747677
H	3.33731311451838	-0.79578847568757	-0.75971862041427

Stannylated carbenic unit

C	4.29395842816769	-0.87265366876564	-0.67026530234674
C	4.00084765608892	-1.90719923211460	-1.55457185666357
H	4.22661068313170	-1.45473710531915	3.69433192189565
O	2.83917596028843	-2.52617442824577	-1.70948605333856
C	2.78458214184001	-3.57609244249857	-2.70652509306455
C	3.28754604072839	-4.89212976775283	-2.14287379415857
H	1.72385268538855	-3.63240916830628	-2.98707153988909
H	3.36880697034574	-3.26520512728220	-3.58482524227069
H	2.71762252891889	-5.17699115695527	-1.24456341276341
H	3.17139964584721	-5.68958369695120	-2.89420160214398
H	4.35302113541394	-4.82015280710167	-1.87789793089860
Sn	5.28541165643969	-1.51273357372376	1.17279022148889
C	5.83230627448309	-3.60209424493044	0.95500263648468
C	7.01011245182695	-0.22890063597496	1.41243767208904
C	3.79512674296659	-1.18889498810277	2.71774011830700
H	6.49128333282784	-3.91647327039720	1.77781870448393
H	4.92476355032180	-4.22372824232719	0.95537933219740
H	6.35610346910956	-3.73078136219092	-0.00338099269662
H	6.68388548944876	0.82087425953782	1.43614620482319
H	7.69694976990417	-0.37716579230021	0.56663226935802
H	7.53044377742218	-0.47297396380754	2.35067917869026
H	2.91440899625836	-1.81867836777235	2.52635462539045
H	3.49455032710544	-0.13145393883763	2.72767820564381
O	5.04432612807674	-2.10343200786000	-2.21285722638182

Fluorinated carbenic unit

C	3.88144258870309	-1.75288099173549	-0.51836549031751
C	5.16814703375988	-2.11074360453508	-0.08894463822336
O	5.68828636610862	-2.29537898612603	1.08341933924357
C	7.14050803001981	-2.46988162381511	1.15424147803716
C	7.84411780863016	-1.12889656740455	1.17209287447673
H	7.45491753205796	-3.09126834833190	0.30369351233119
H	7.28997863107175	-3.03126196684360	2.08499951557361
H	8.92640485511233	-1.28920632078100	1.29920951675789
H	7.48498954293832	-0.50677679207663	2.00591219201687
H	7.68677335043909	-0.58727946954445	0.22730889785582
O	5.70946735928088	-2.09756285828465	-1.20818881387068
C	2.99177325649013	-2.87160130052410	-0.97069701583147
F	3.59370454750831	-4.05313671532158	-1.21526955564976
F	2.13113639121300	-3.05452780961838	0.05248288166683
F	2.29278392406661	-2.56428144335742	-2.06550986166690

NMR Spectra of New Compounds

S6: ^{1}H NMR (400 MHz, $CDCl_3$)

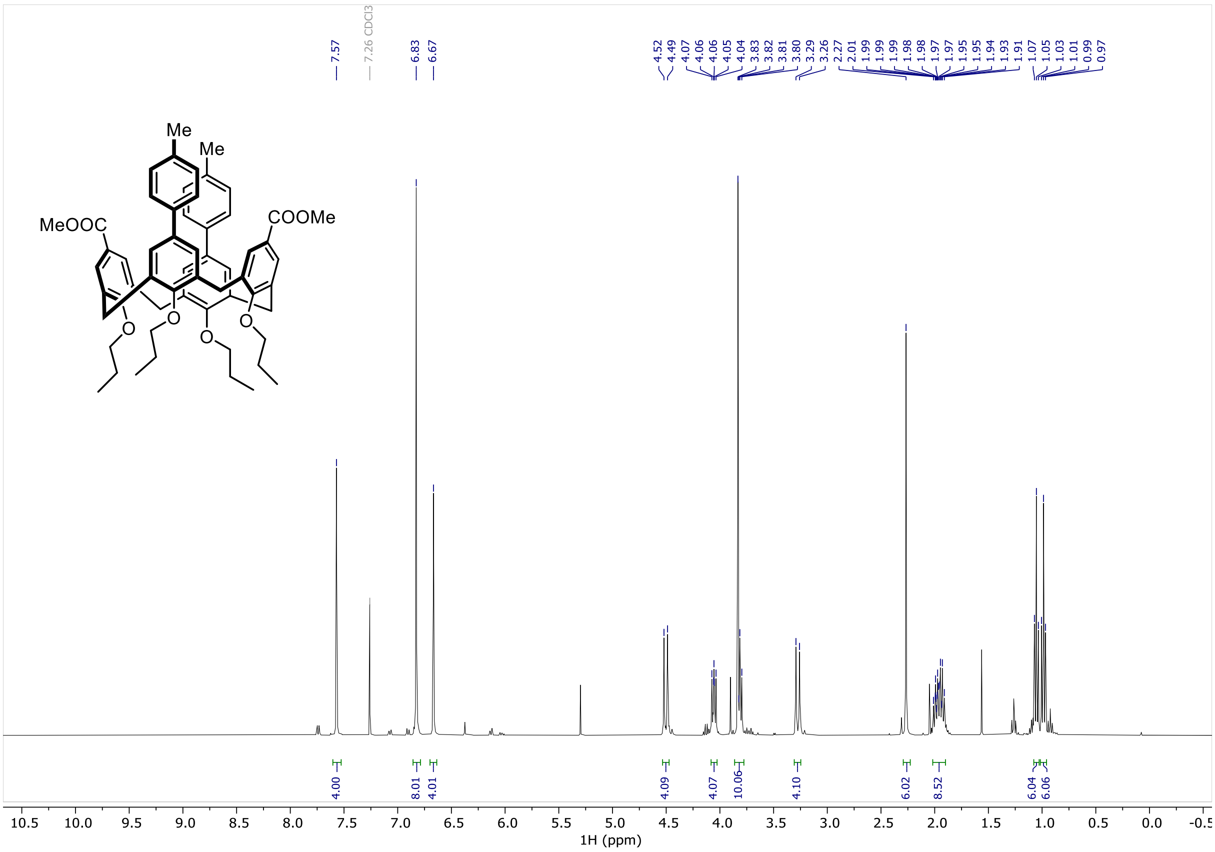

S6: ^{13}C NMR (101 MHz, $CDCl_3$)

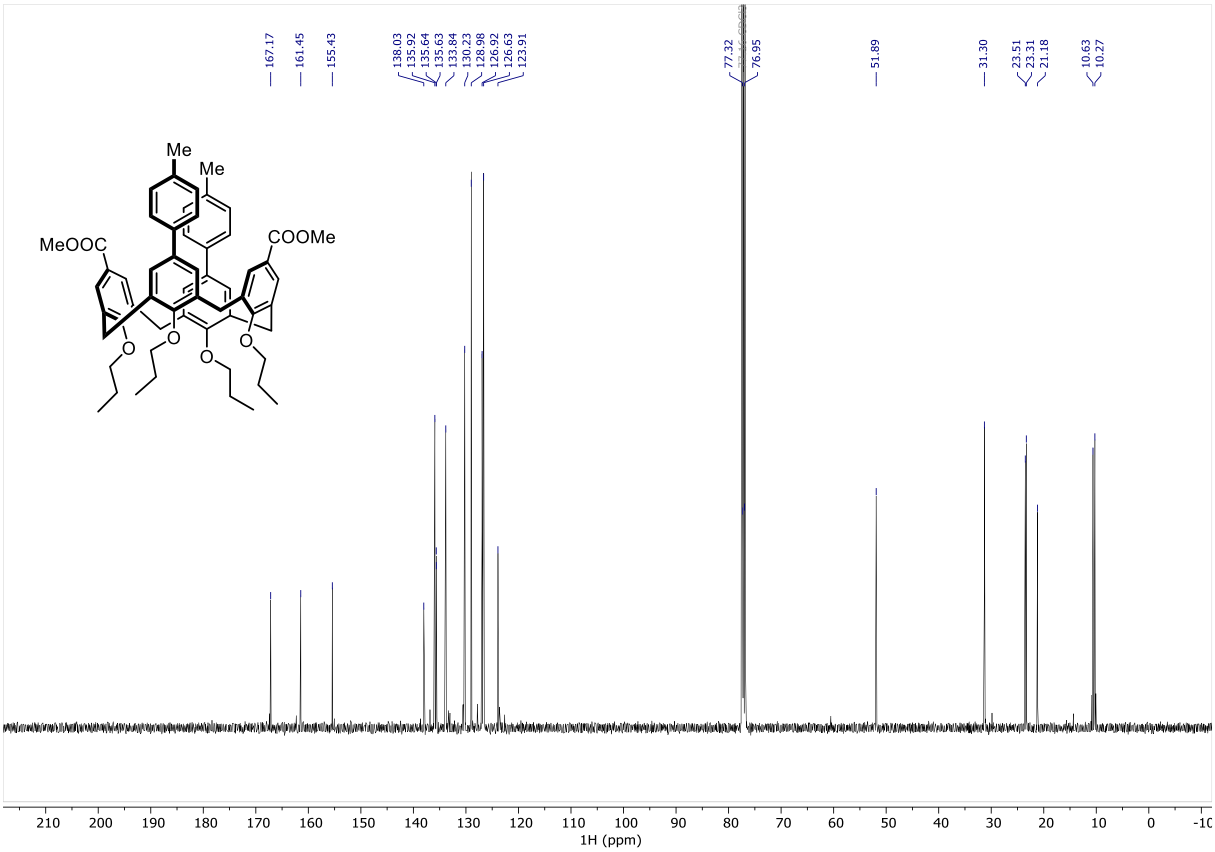

S7: ^{1}H NMR (400 MHz, $CDCl_3$)

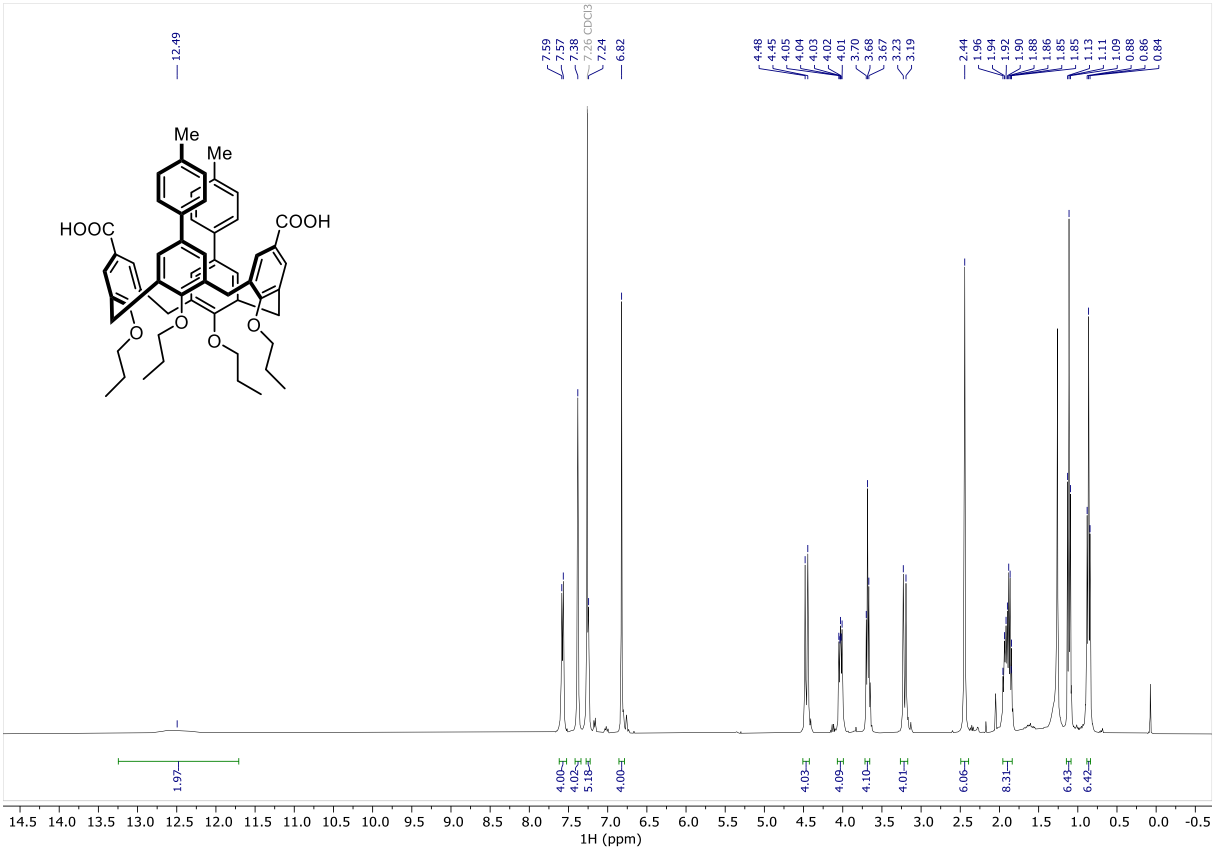

S7: ^{13}C NMR (101 MHz, $CDCl_3$)

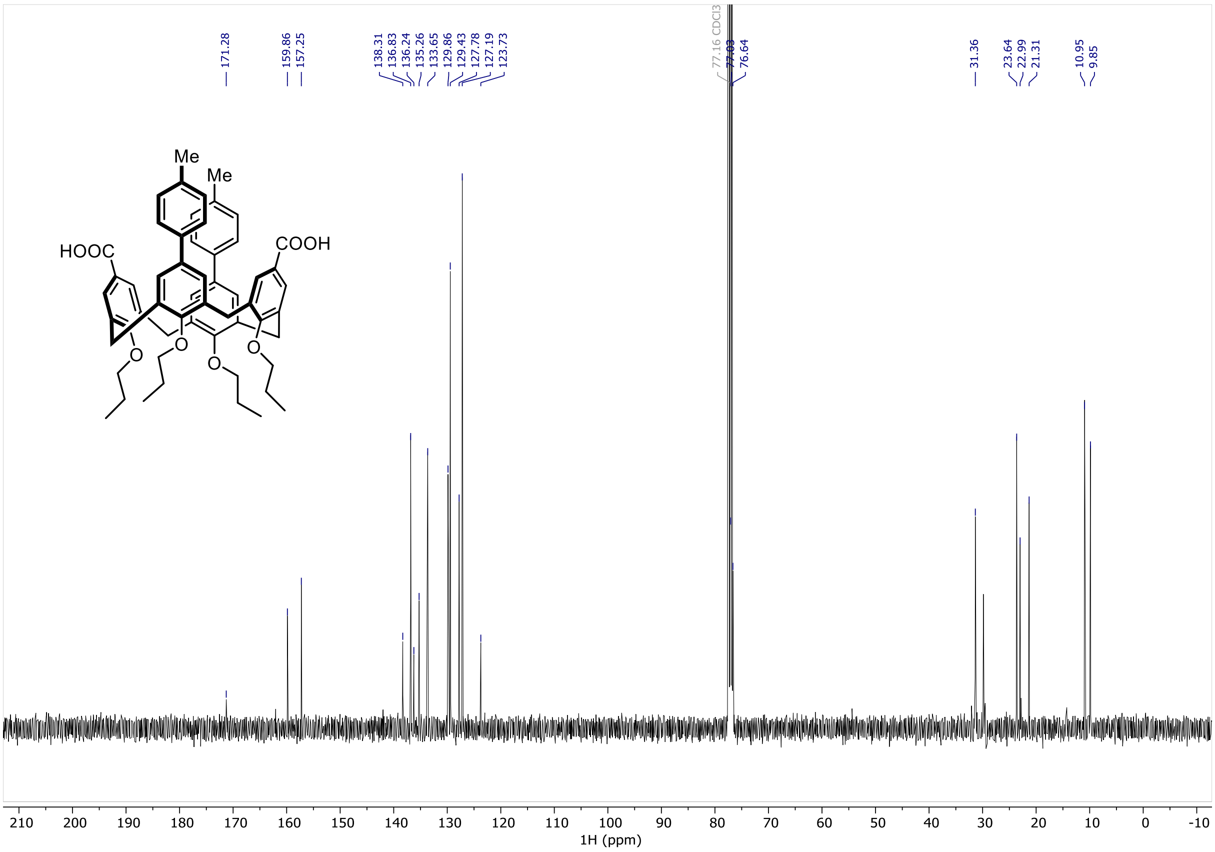

***P*-4 [the complex is unstable in solution, ≥10% impurities]**: ^{1}H NMR (600 MHz, [D$_8$]-THF)

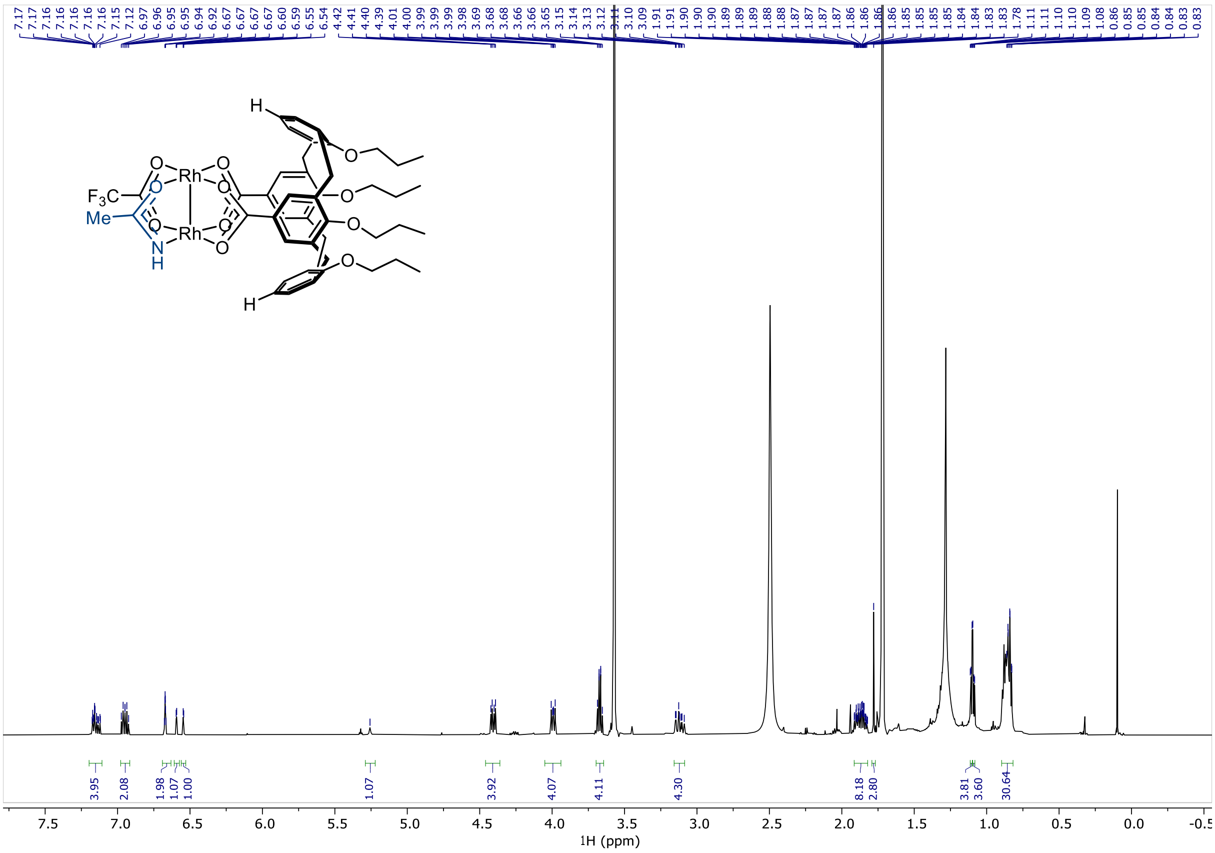

***P*-4 [the complex is unstable in solution, ≥10% impurities]**: ^{13}C NMR (151 MHz, [D$_8$]-THF)

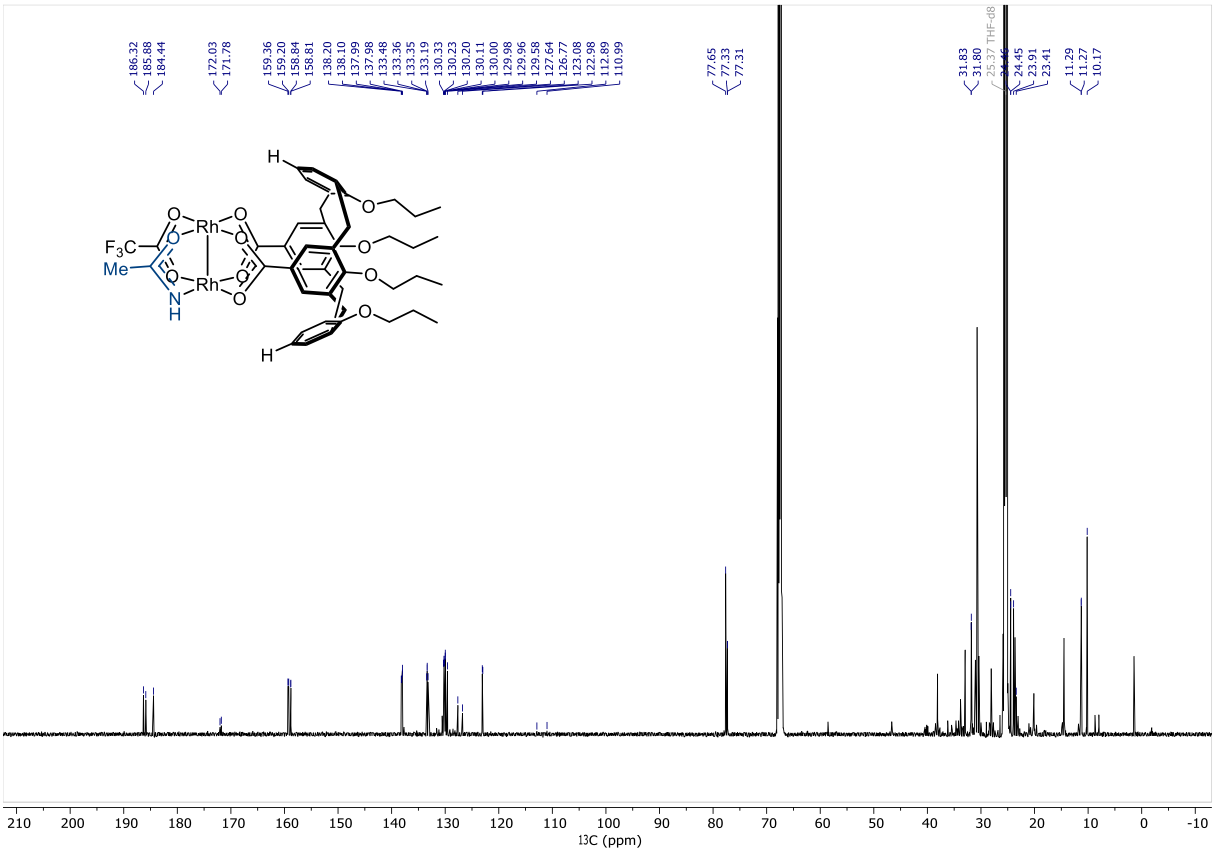

***P*-4 [*the complex is unstable in solution, ≥ 10% impurities*]:** ^{19}F NMR (565 MHz, [D$_8$]-THF)

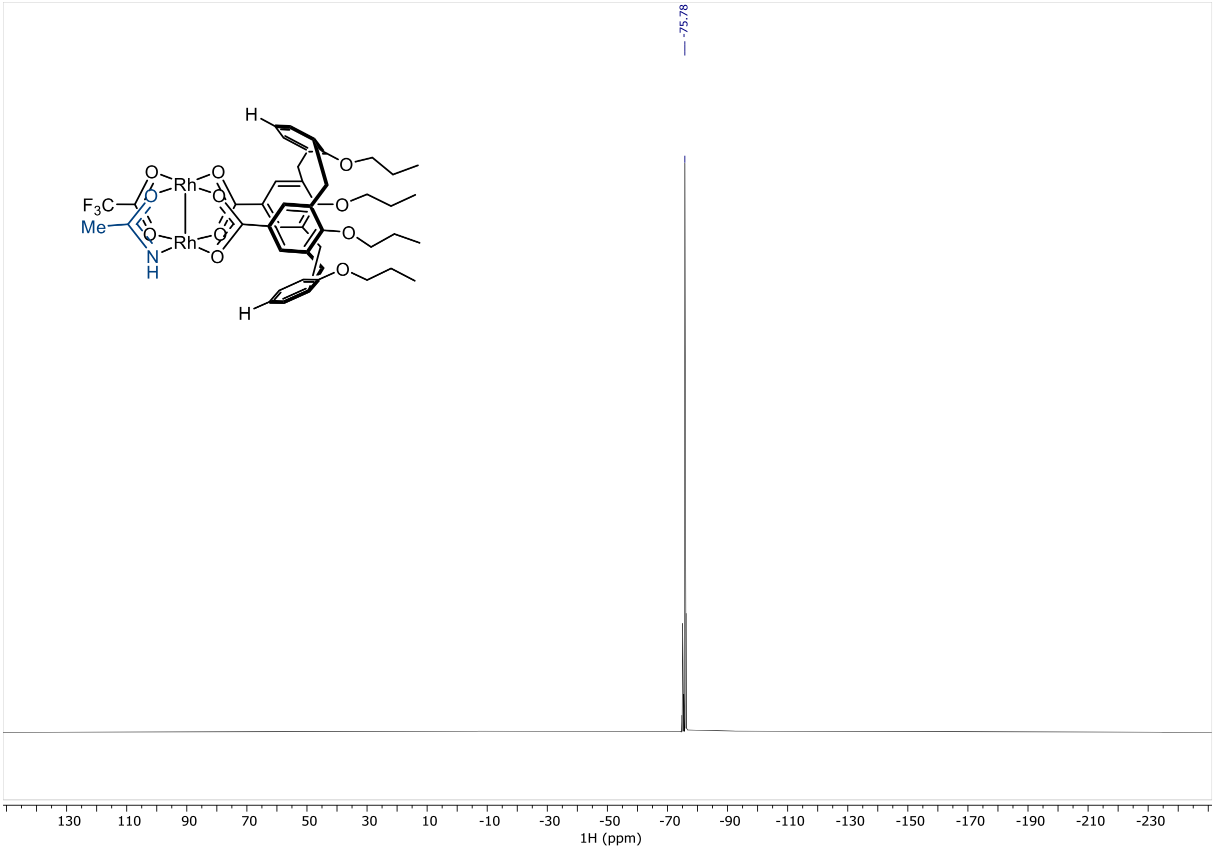

***P*-5:** ^{1}H NMR (600 MHz, [D$_8$]-THF)

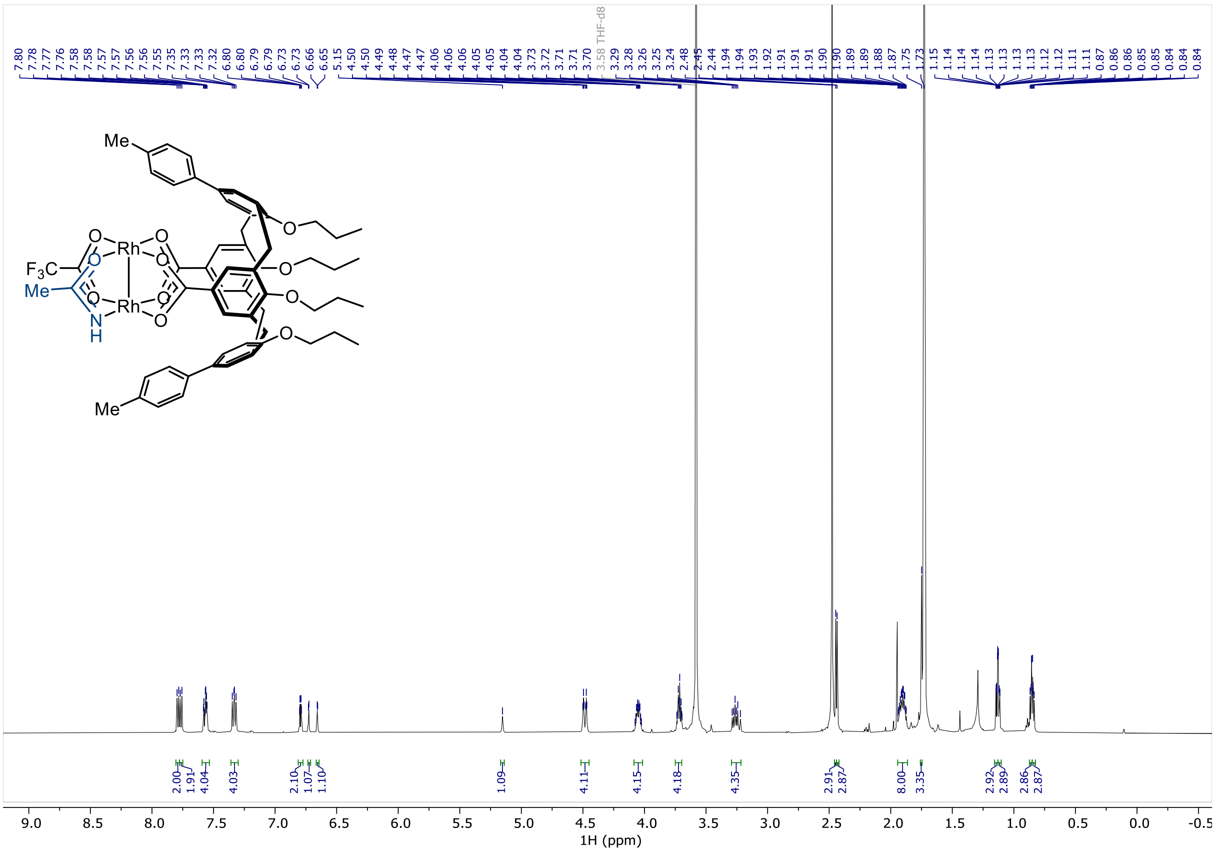

***P*-5:** ^{13}C NMR (151 MHz, [D$_8$]-THF)

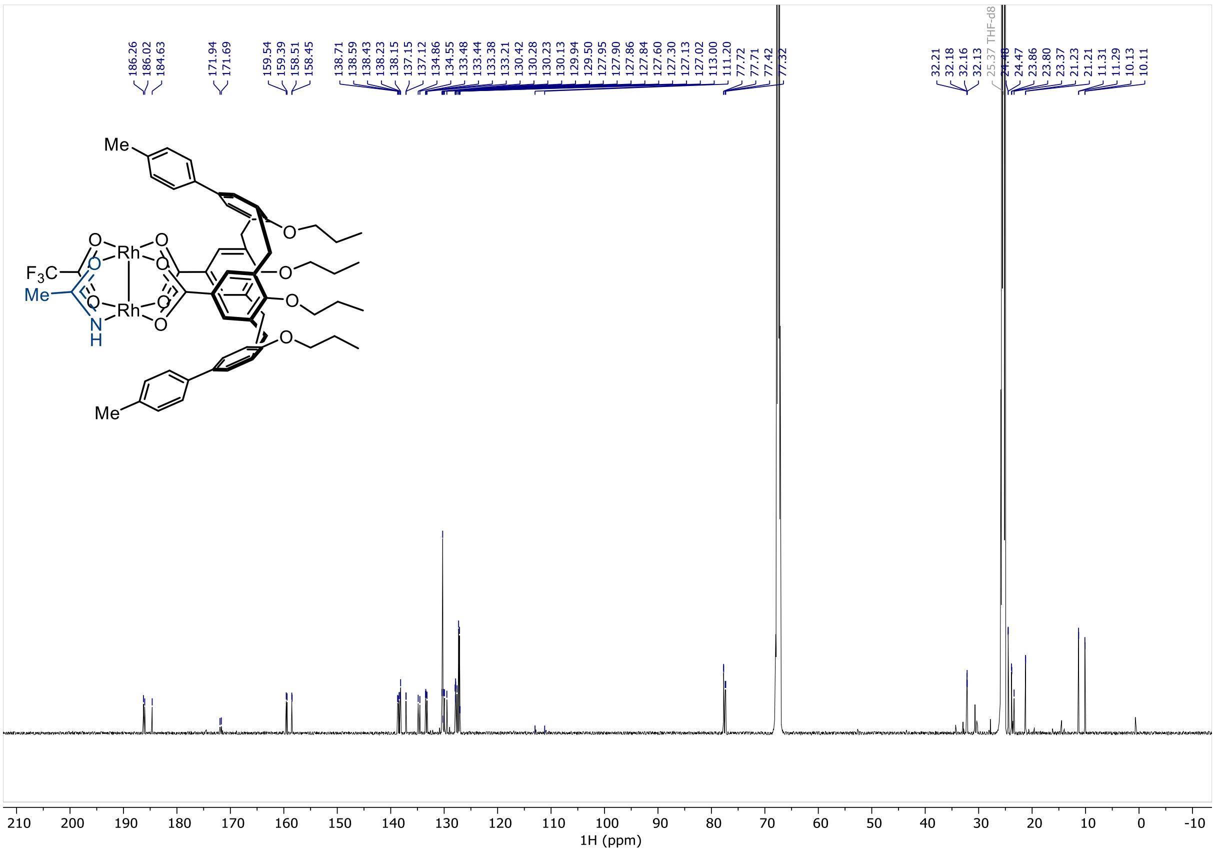

***P*-5:** ^{19}F NMR (565 MHz, [D$_8$]-THF)

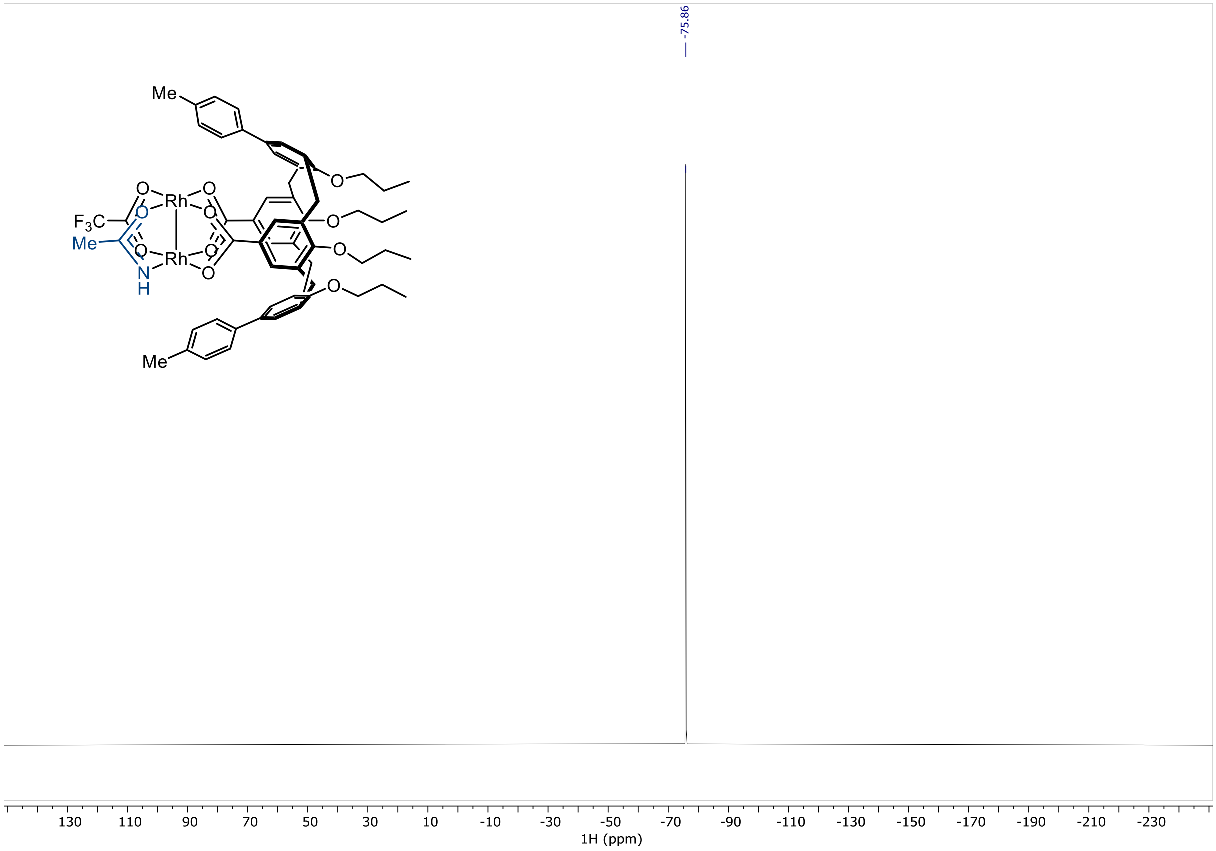

S10: ^{1}H NMR (600 MHz, $CDCl_3$)

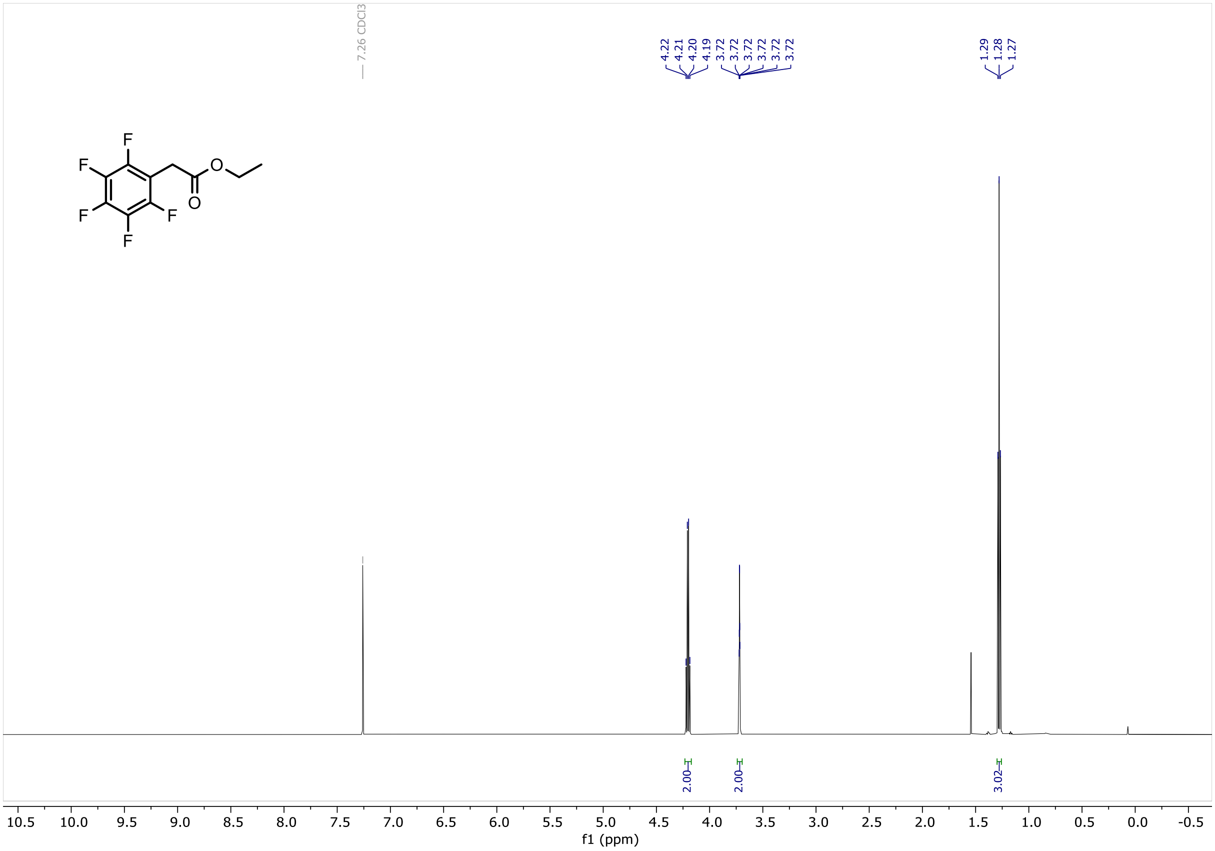

S10: ^{13}C NMR (151 MHz, $CDCl_3$)

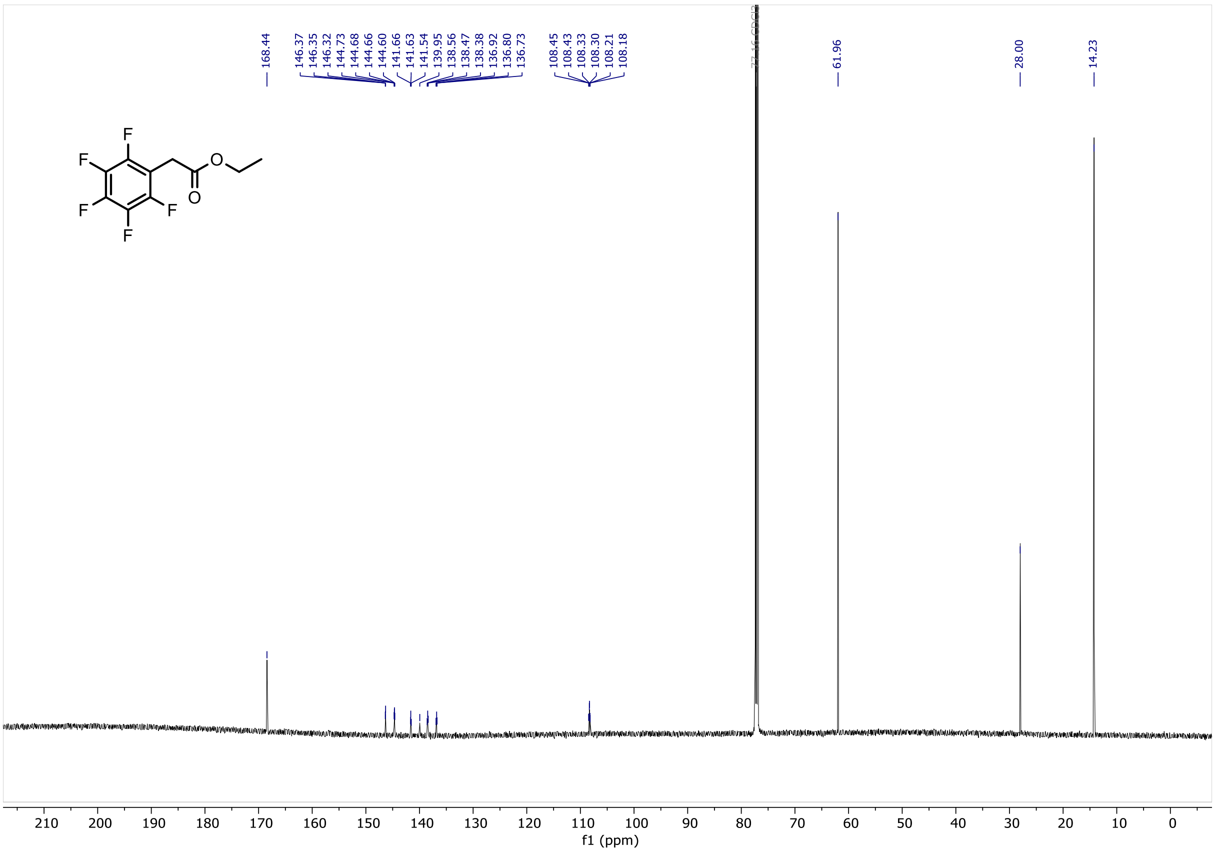

S10: ^{19}F NMR (565 MHz, $CDCl_3$)

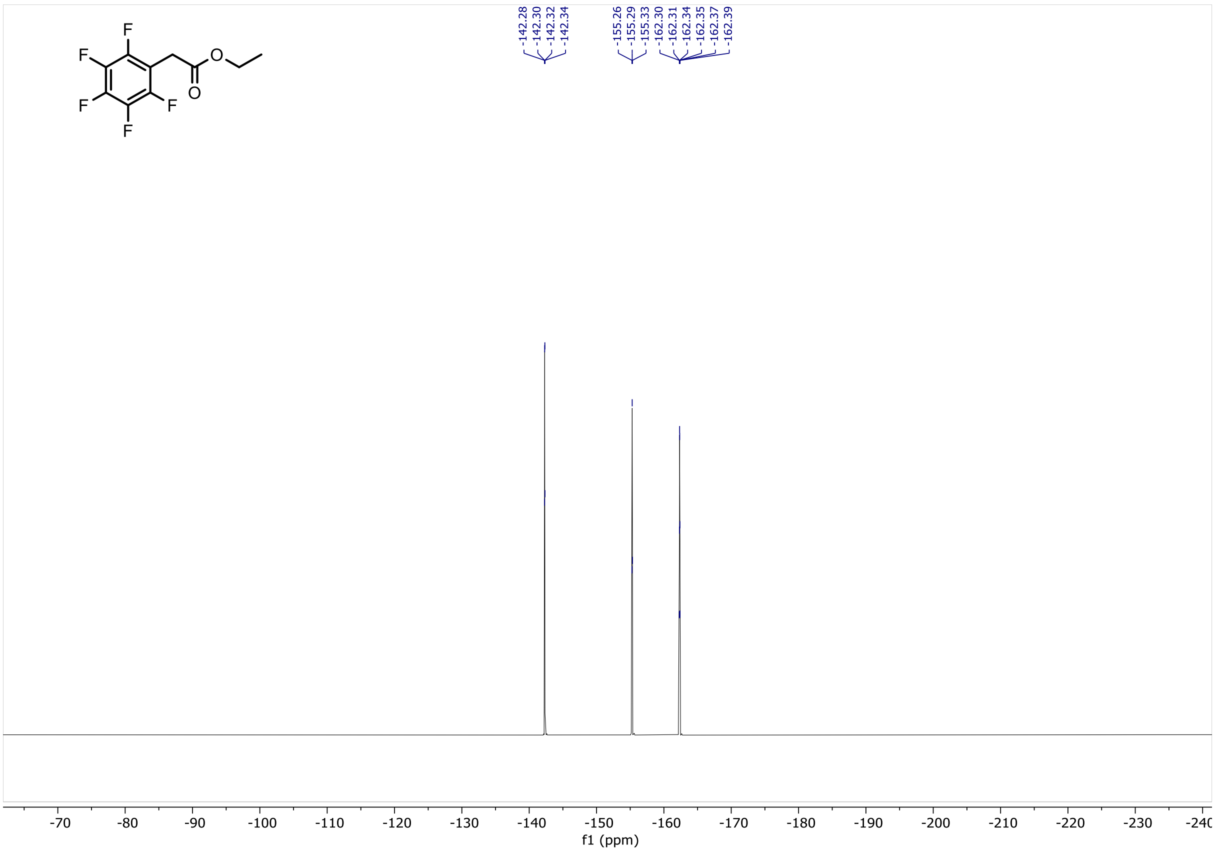

S11: ^{1}H NMR (400 MHz, $CDCl_3$)

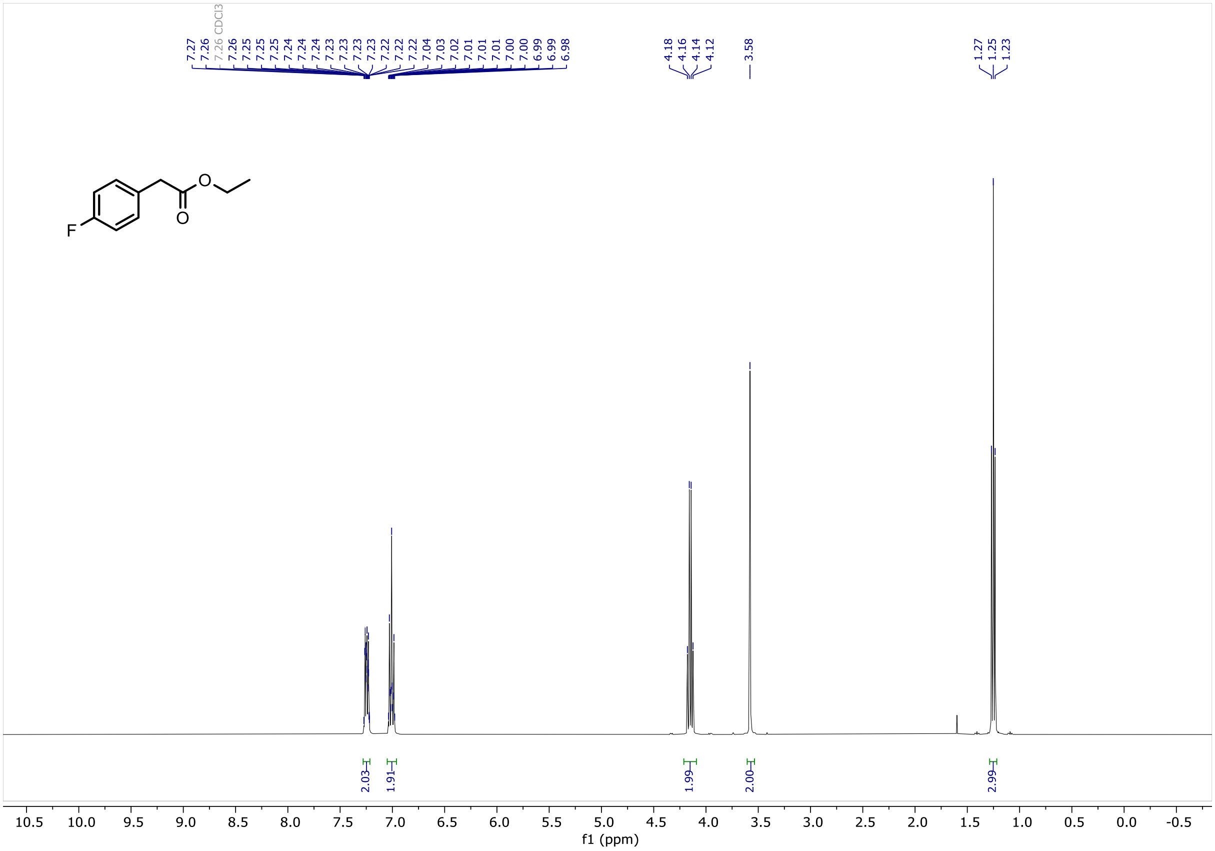

S11: ^{13}C NMR (101 MHz, $CDCl_3$)

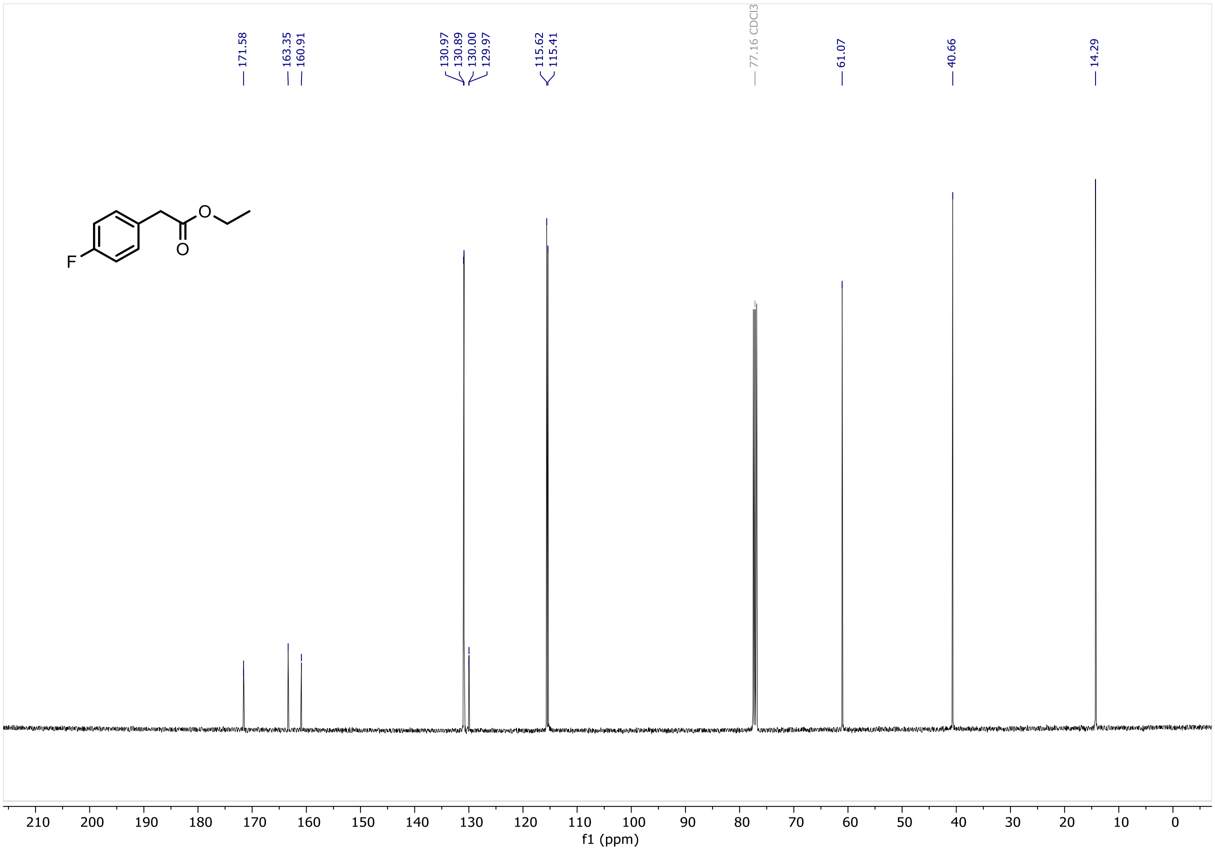

S11: ^{19}F NMR (282 MHz, $CDCl_3$)

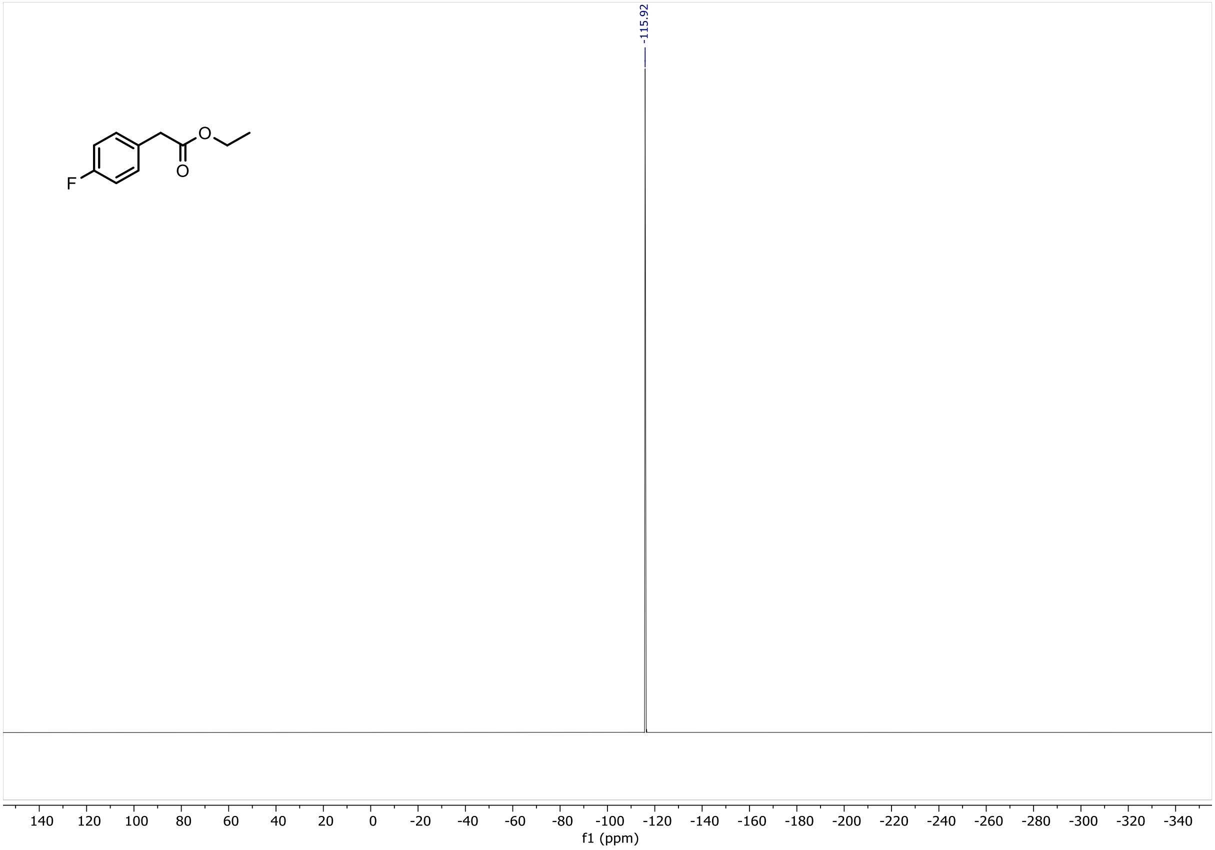

S12: ^{1}H NMR (400 MHz, $CDCl_3$)

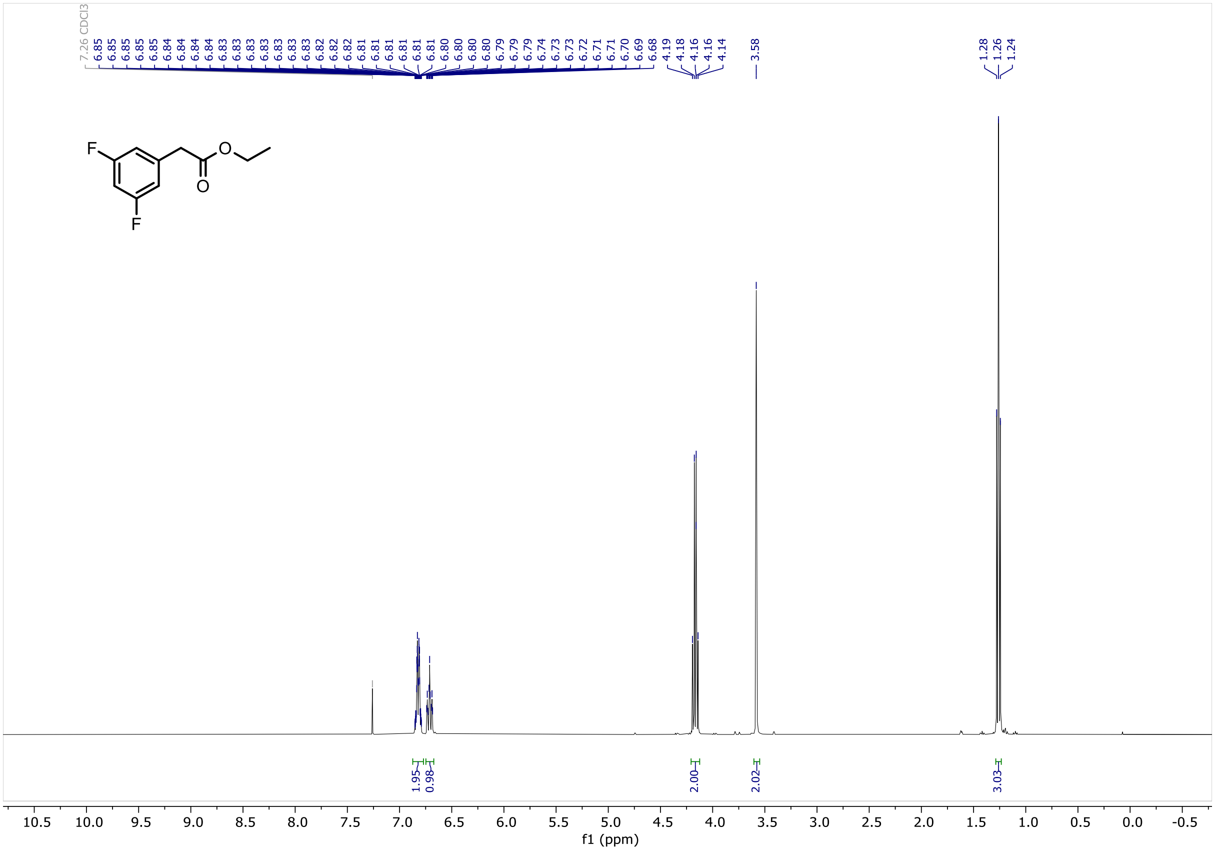

S12: ^{13}C NMR (101 MHz, $CDCl_3$)

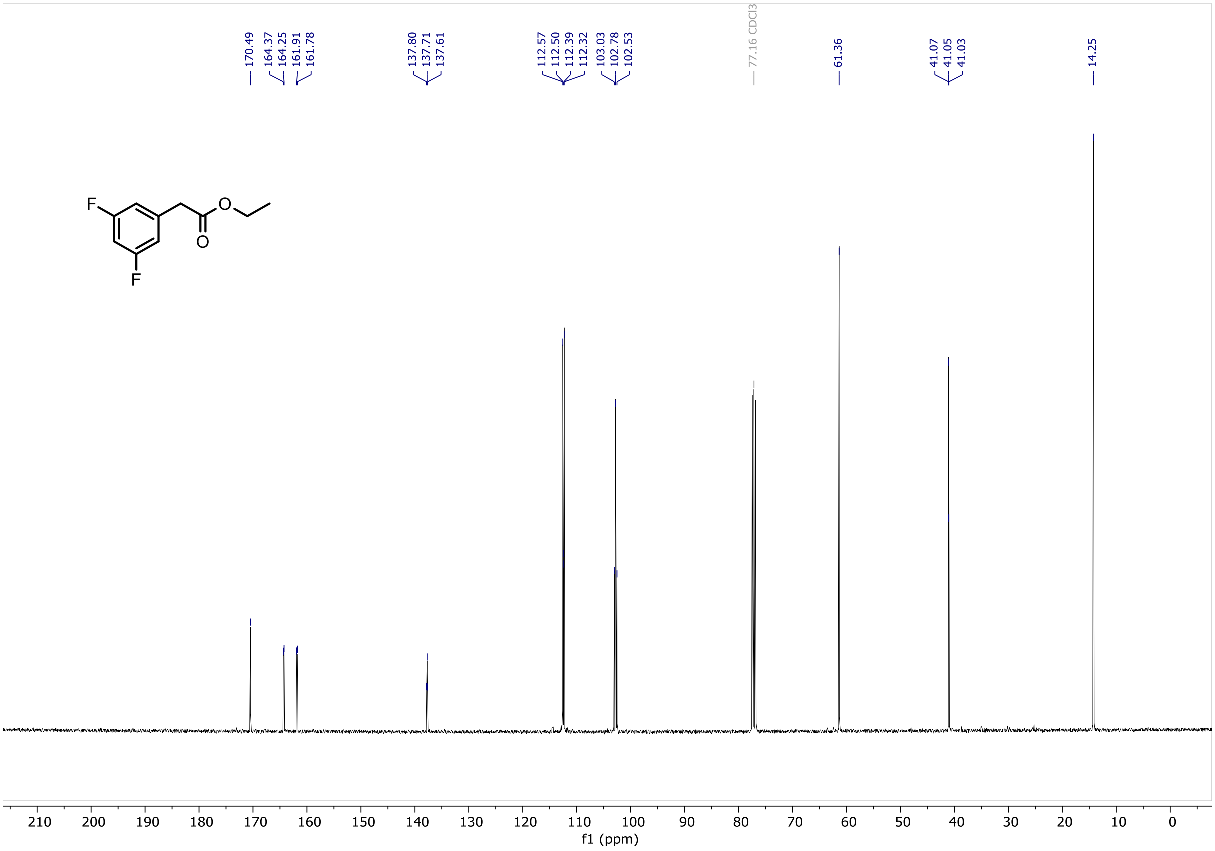

S12: ^{19}F NMR (282 MHz, $CDCl_3$)

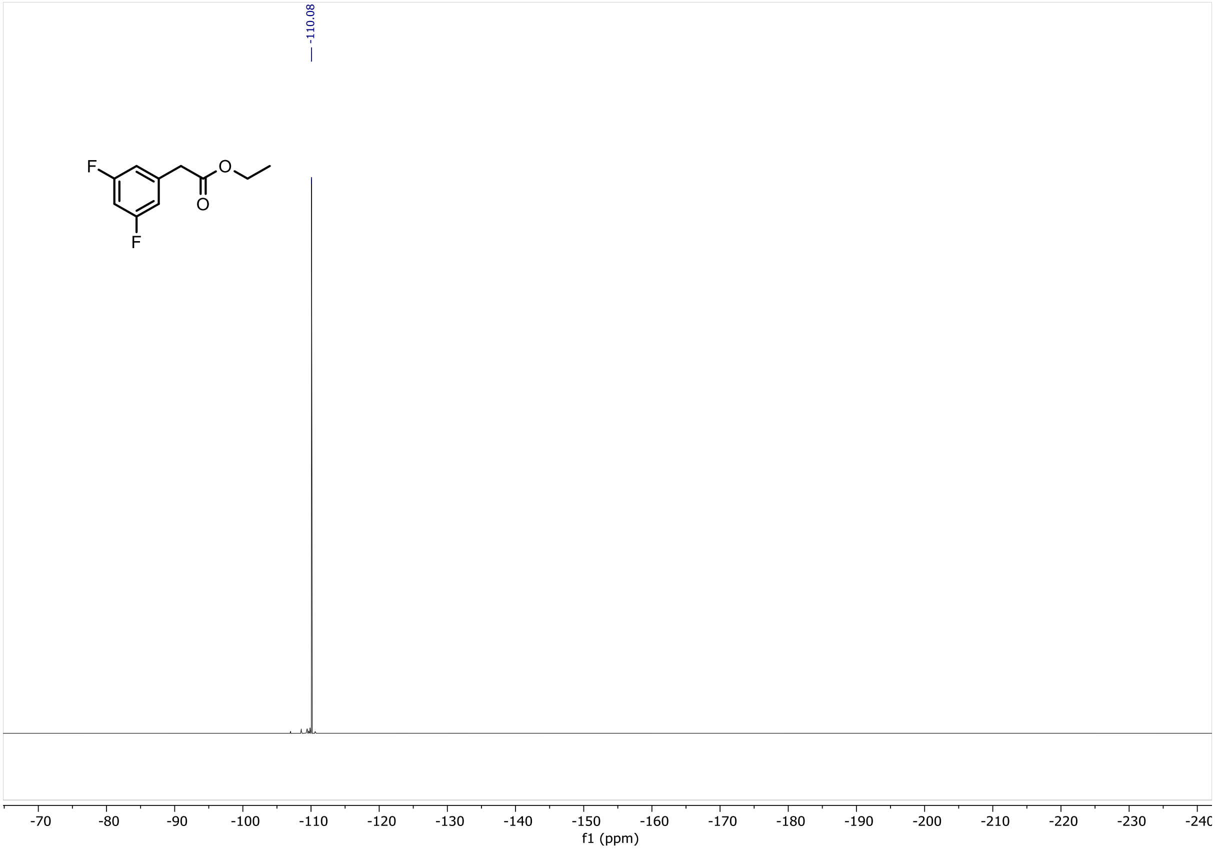

S13: ^{1}H NMR (400 MHz, $CDCl_3$)

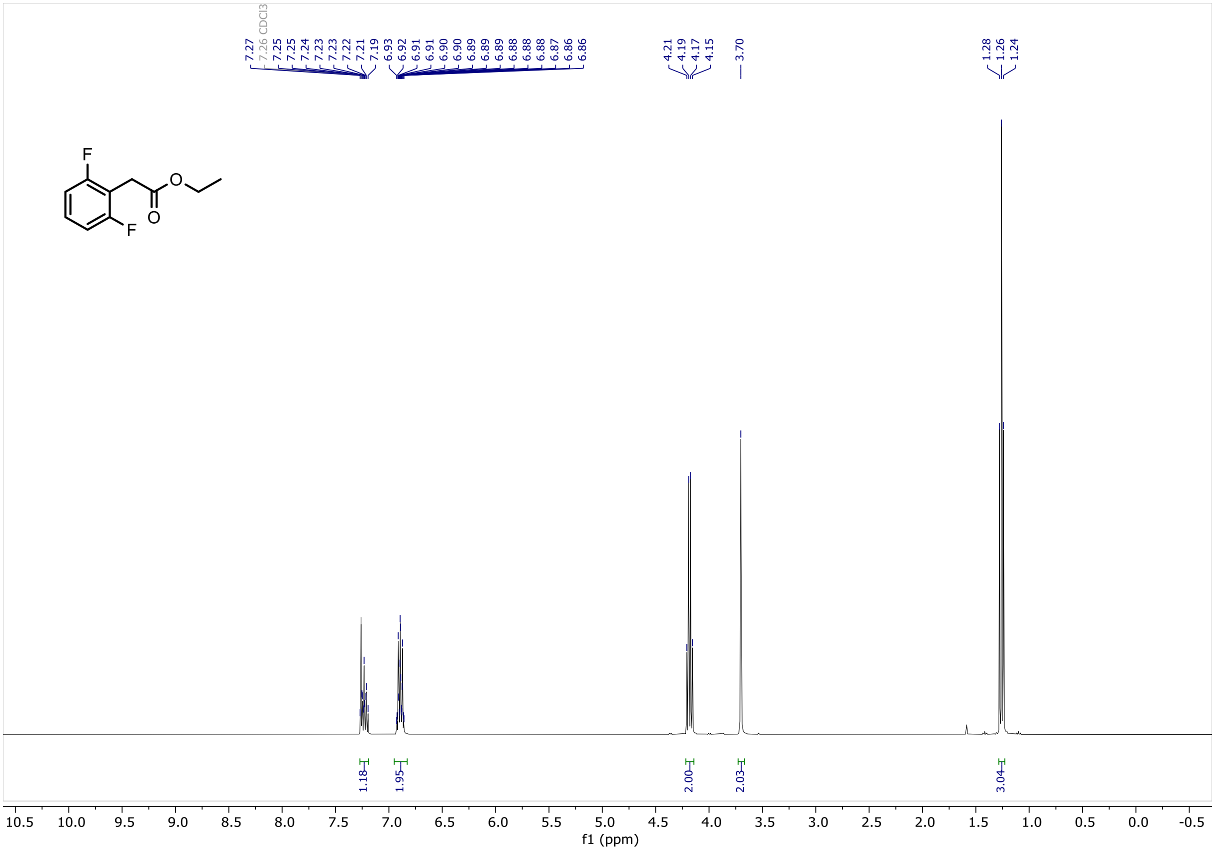

S13: ^{13}C NMR (101 MHz, $CDCl_3$)

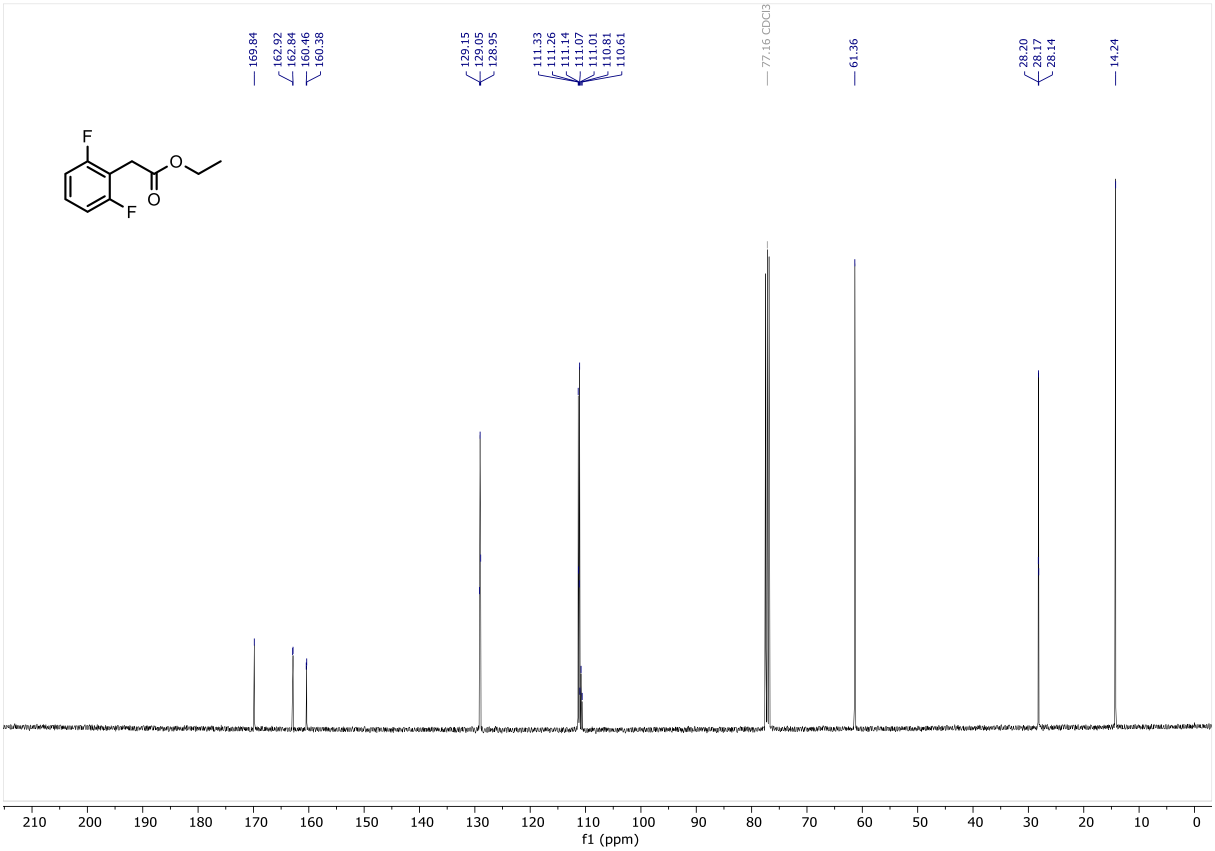

S13: ^{19}F NMR (282 MHz, $CDCl_3$)

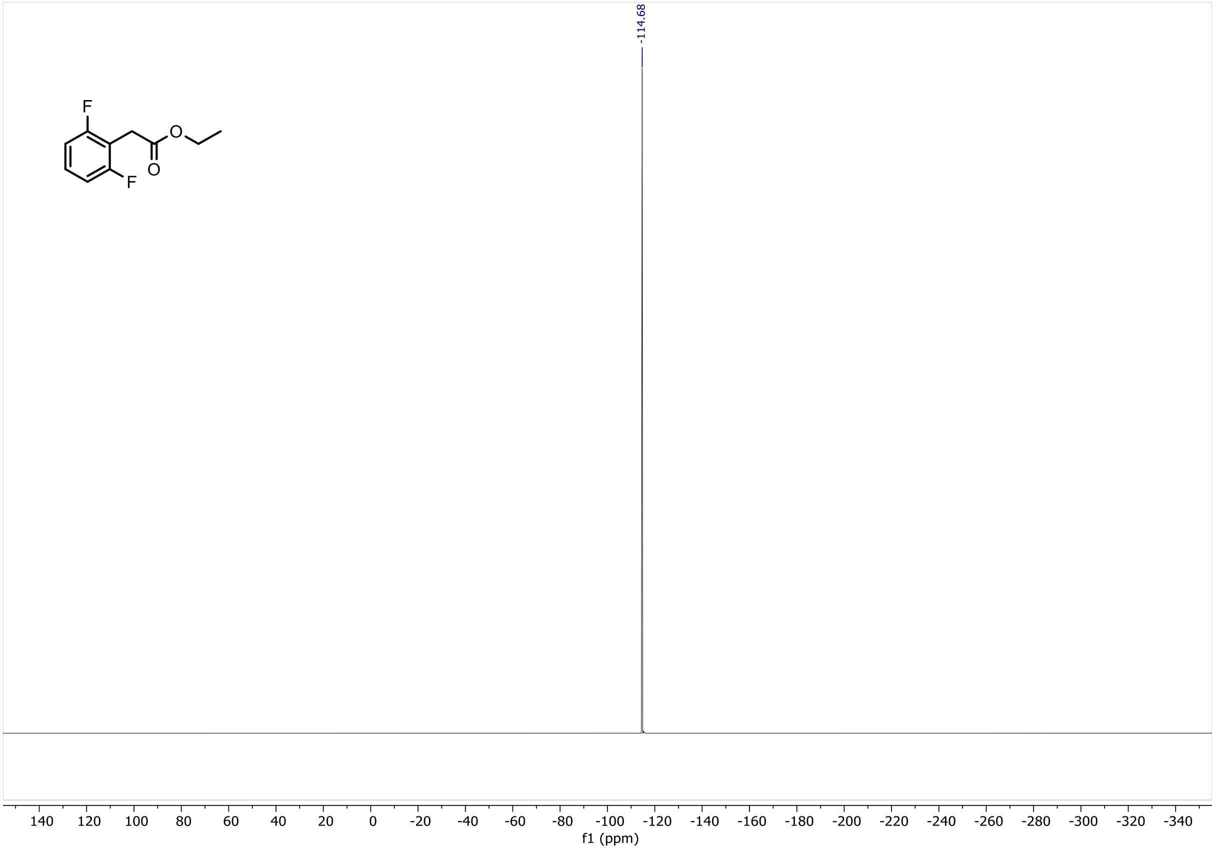

8: ^{1}H NMR (600 MHz, $CDCl_3$)

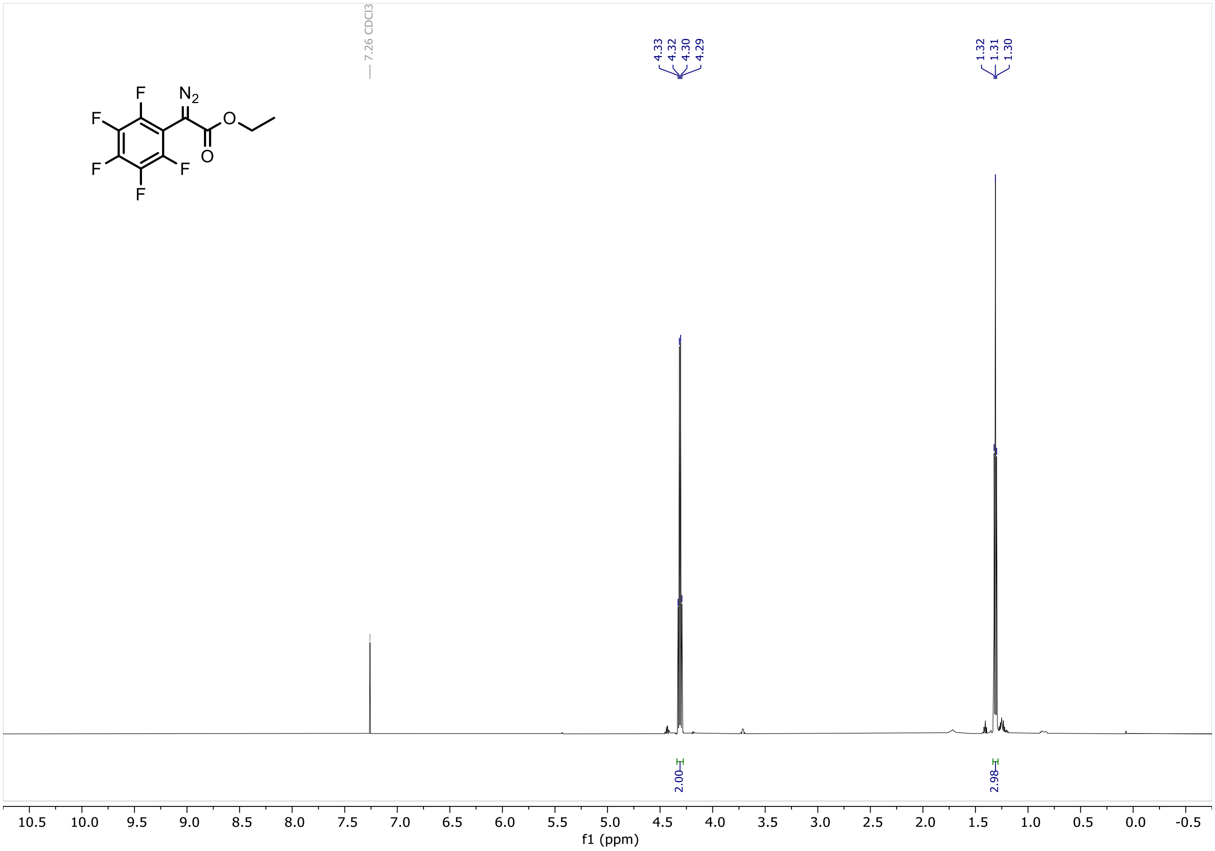

8: ^{13}C NMR (151 MHz, $CDCl_3$)

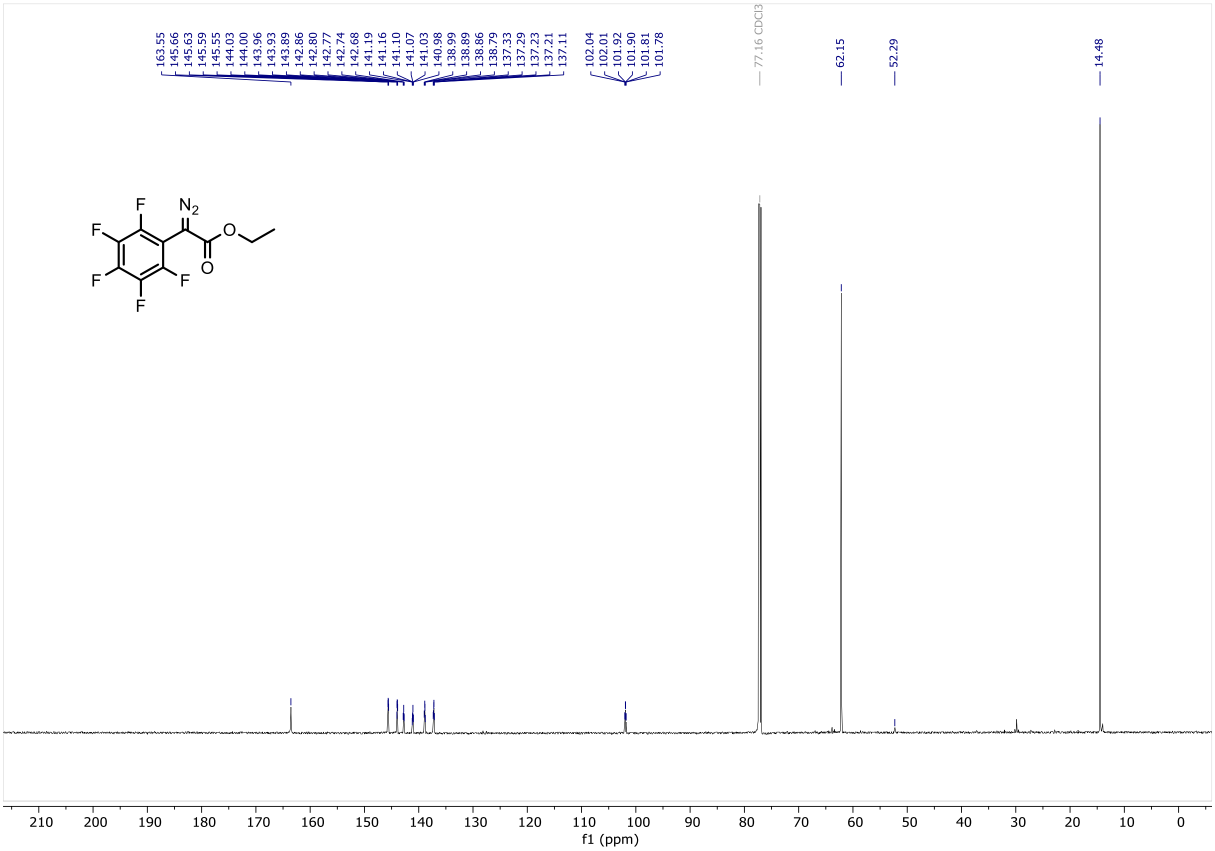

8: ^{19}F NMR (565 MHz, $CDCl_3$)

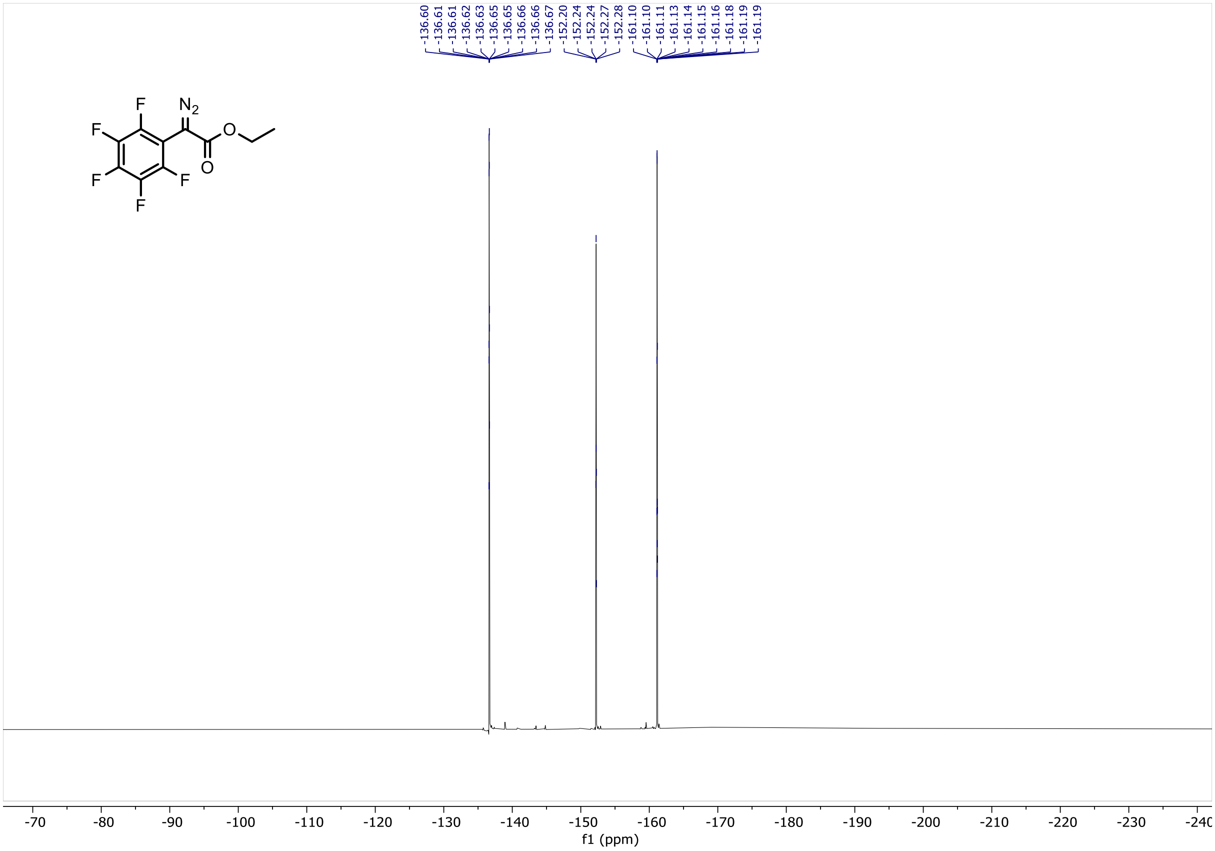

S14: ^{1}H NMR (400 MHz, $CDCl_3$)

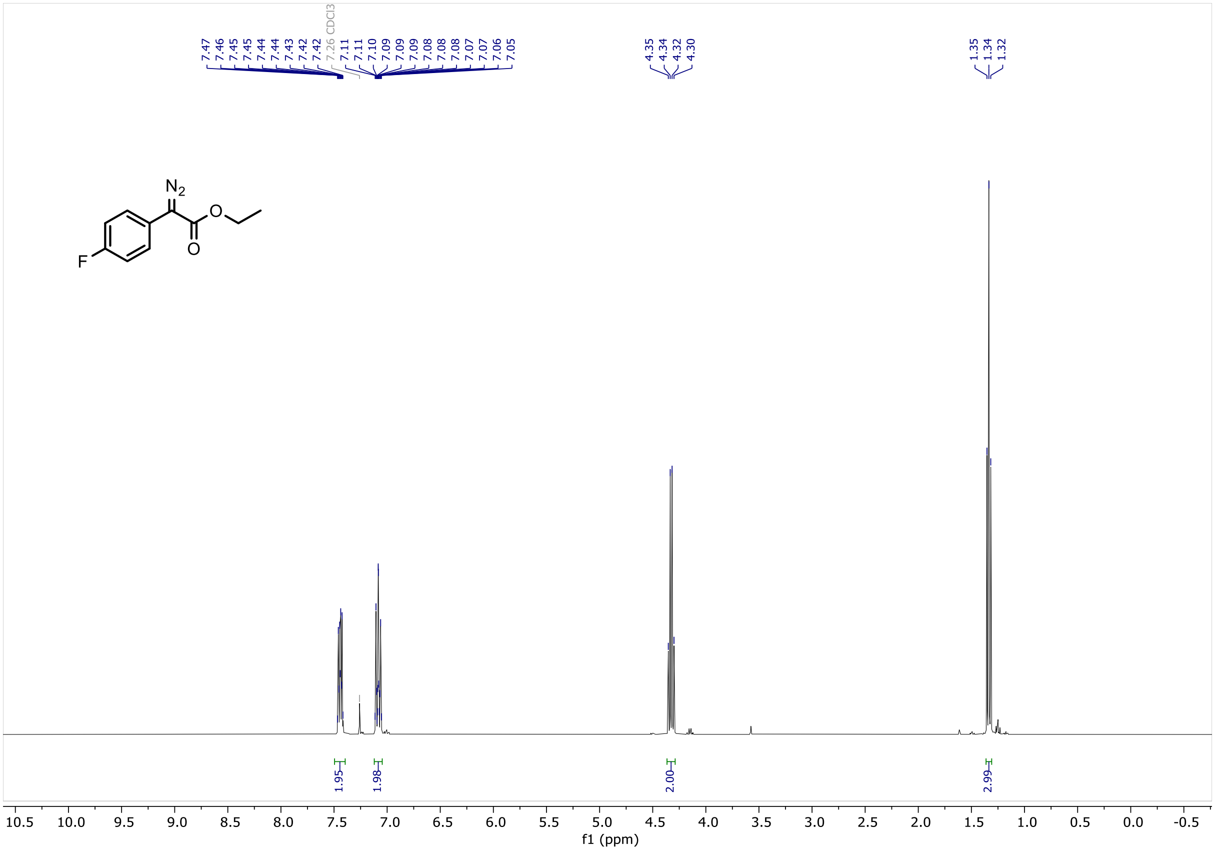

S14: ^{13}C NMR (101 MHz, $CDCl_3$)

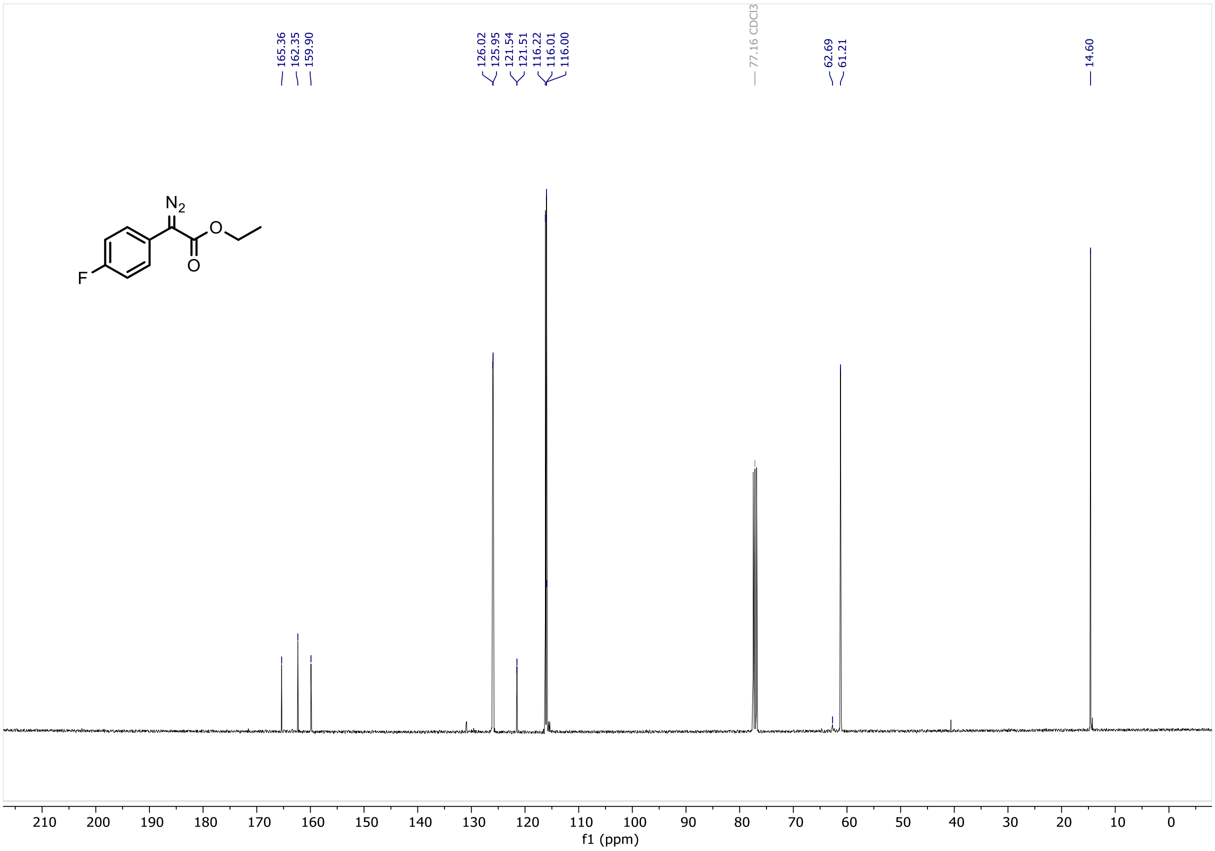

S14: ^{19}F NMR (282 MHz, $CDCl_3$)

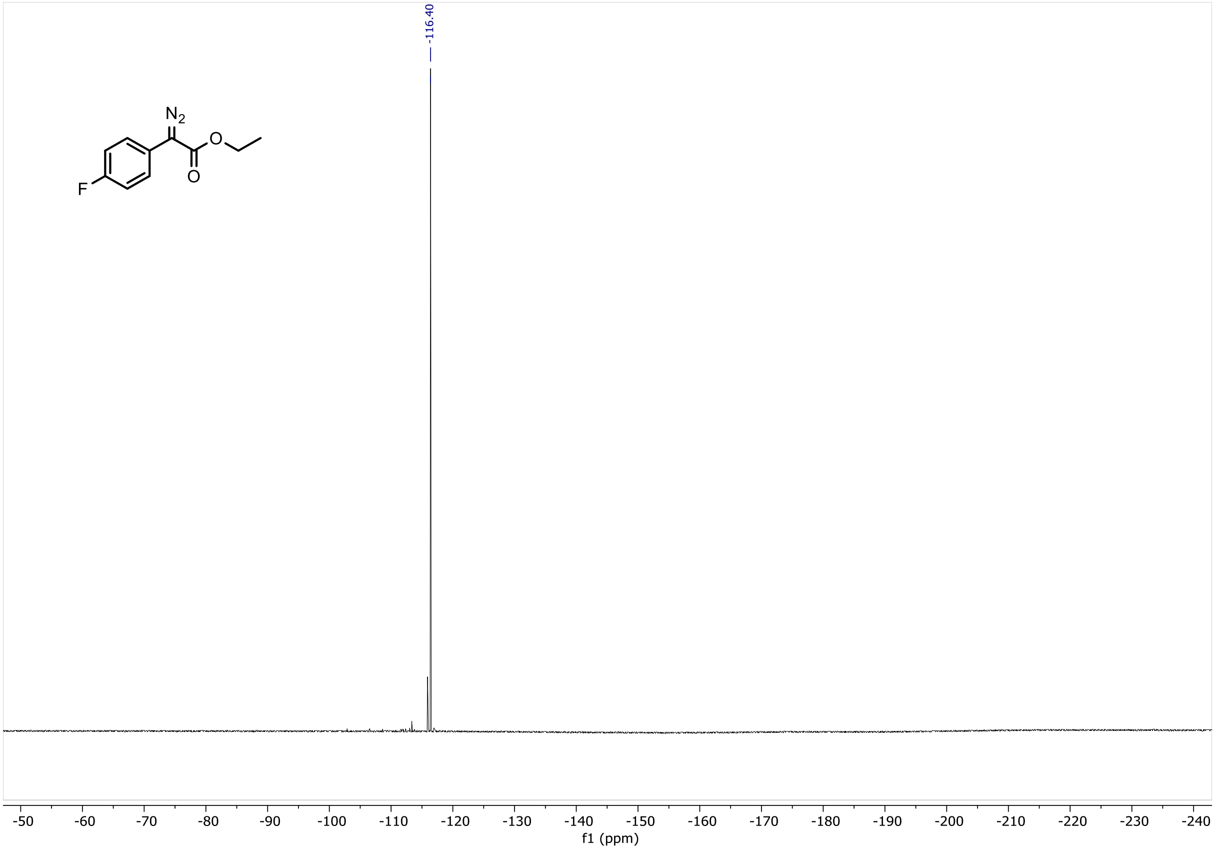

S15: ^{1}H NMR (400 MHz, $CDCl_3$)

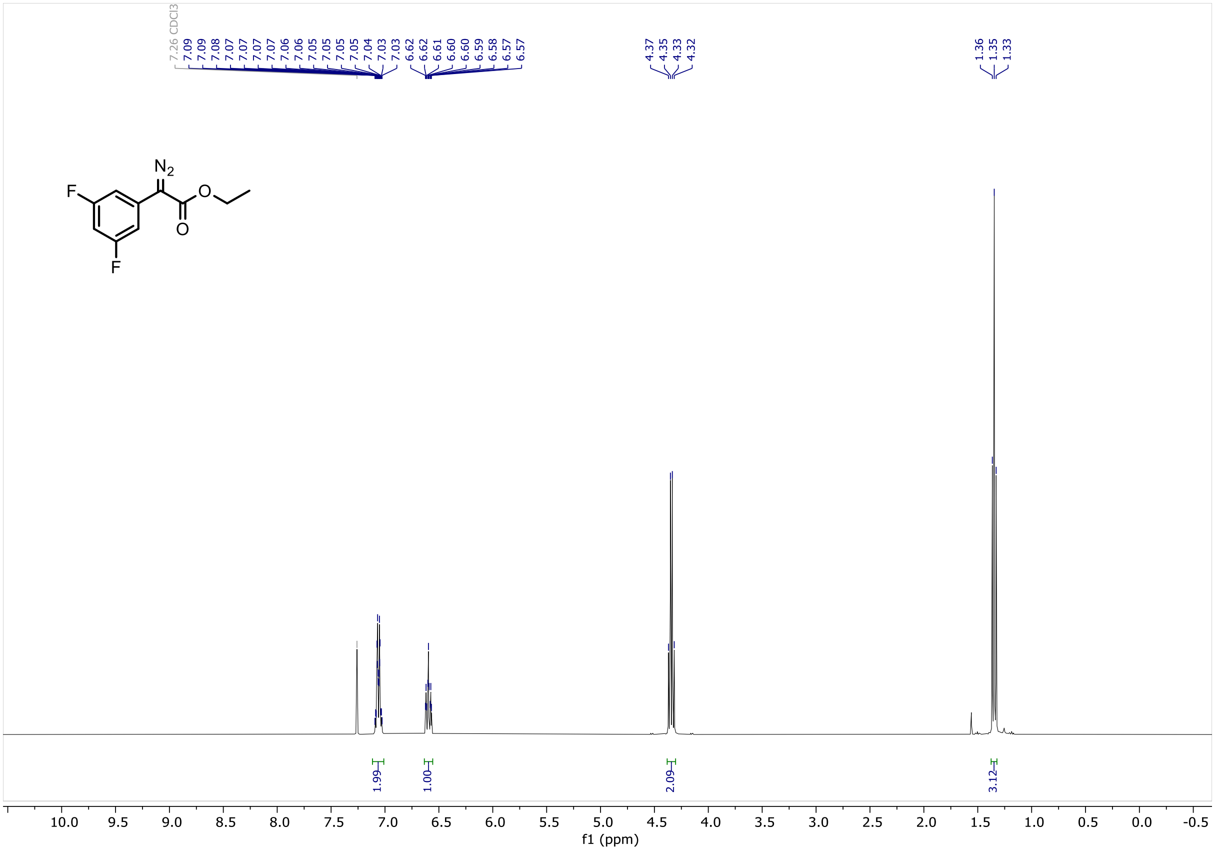

S15: ^{13}C NMR (101 MHz, $CDCl_3$)

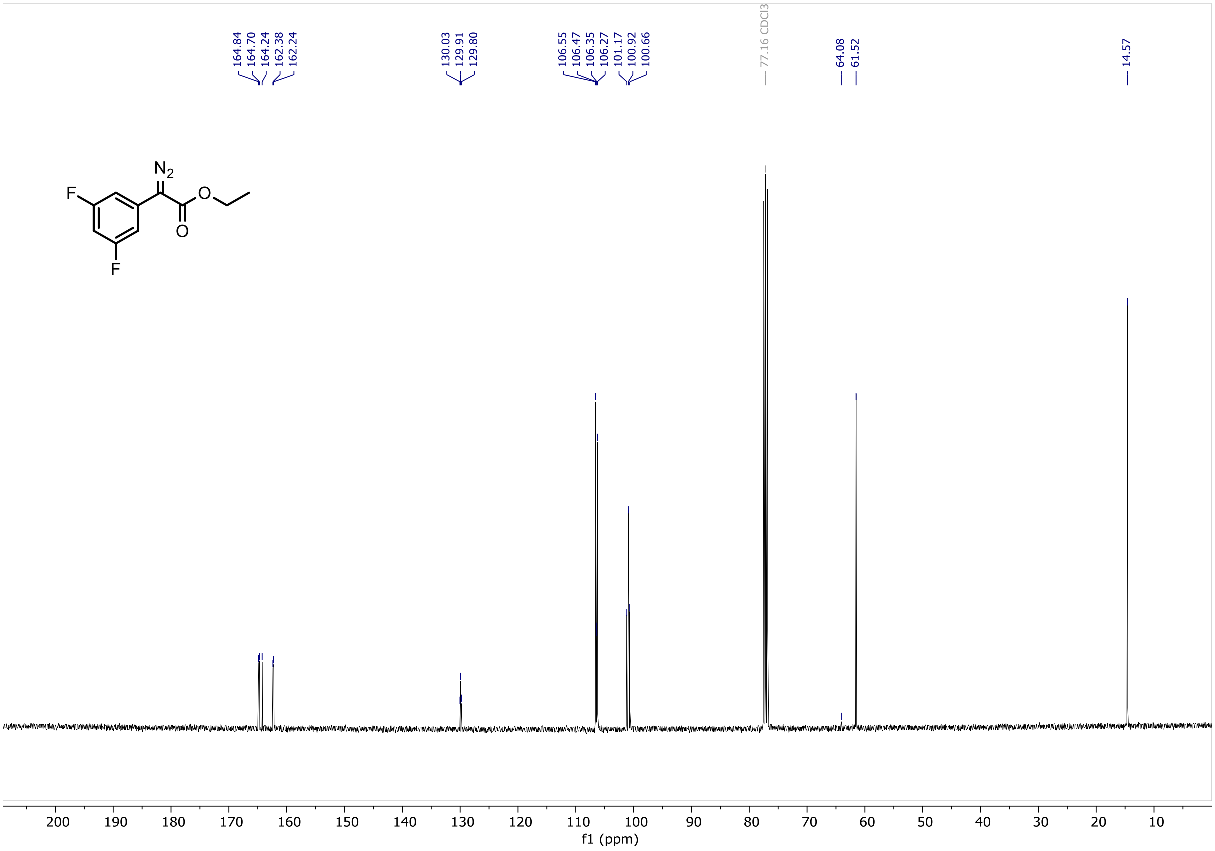

S15: ^{19}F NMR (282 MHz, $CDCl_3$)

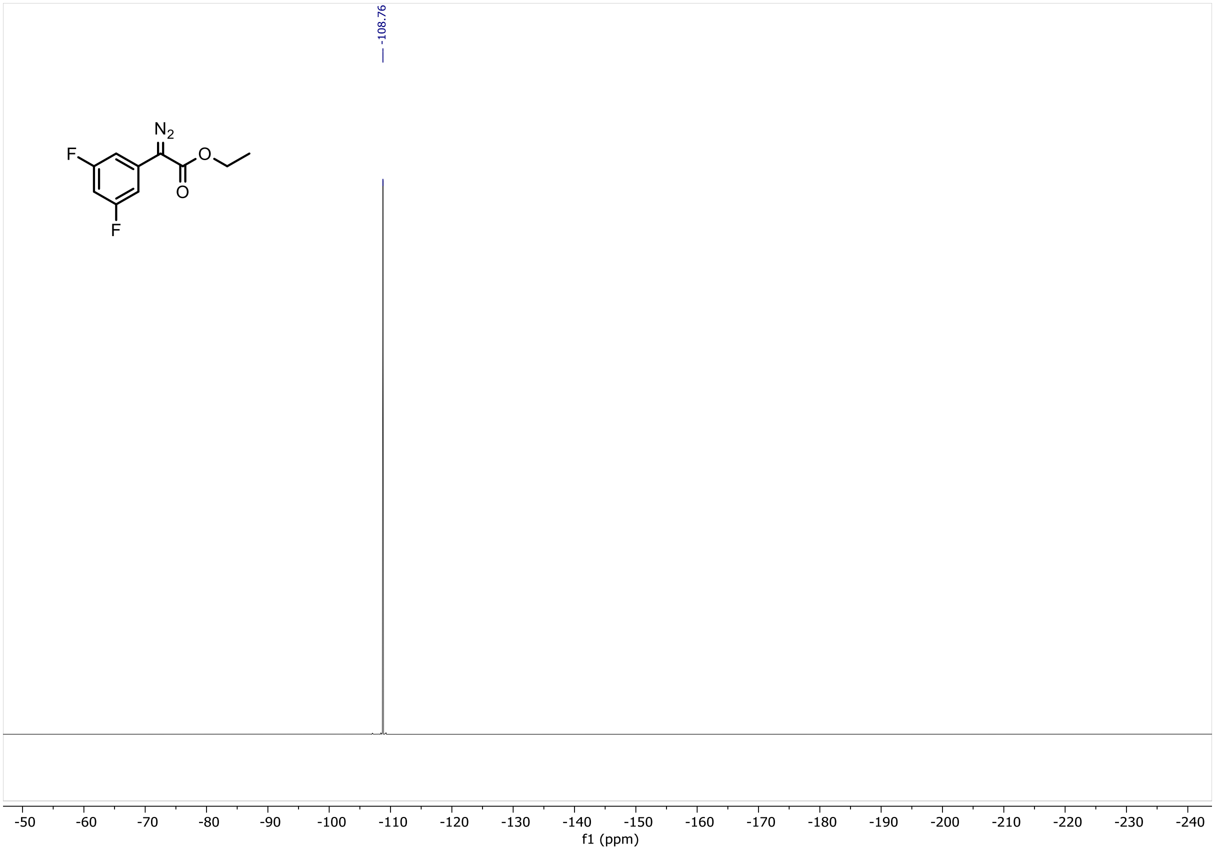

S16: ^{1}H NMR (300 MHz, $CDCl_3$)

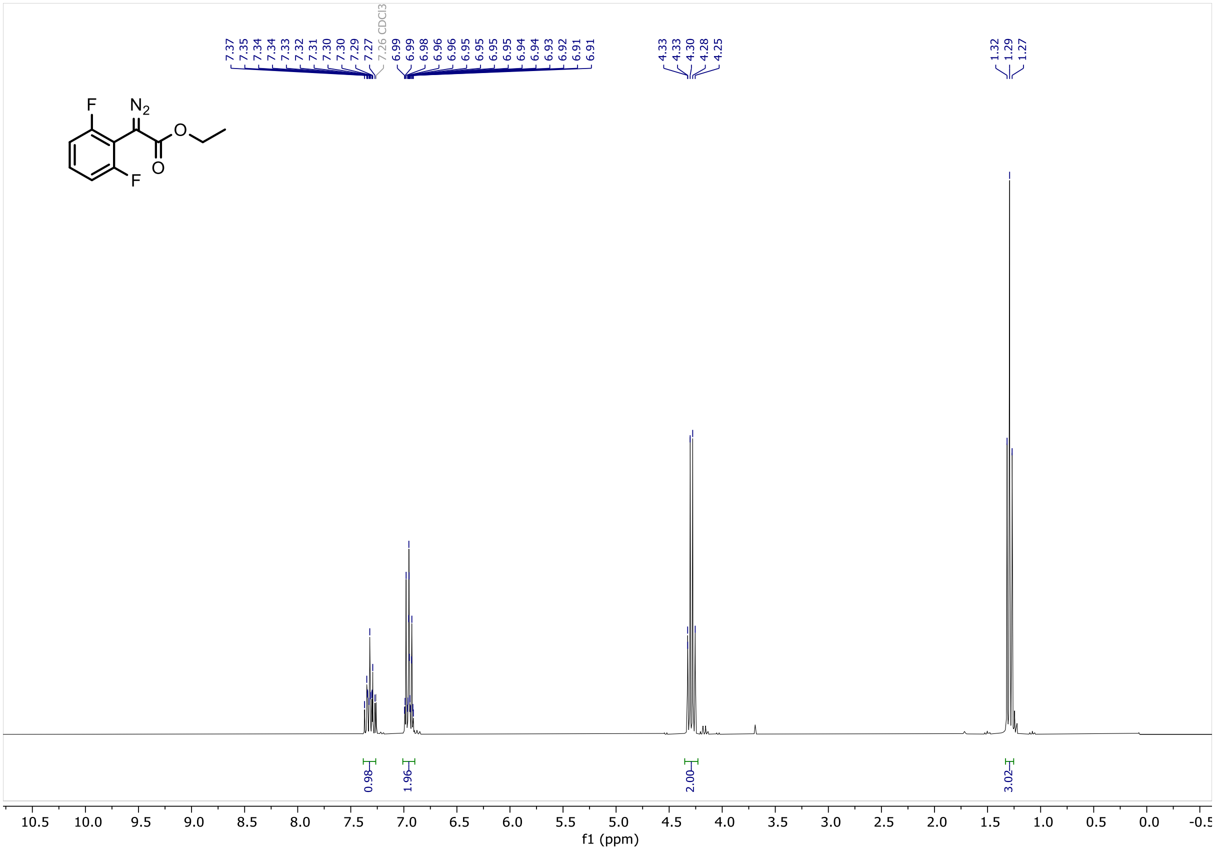

S16: ^{13}C NMR (101 MHz, $CDCl_3$)

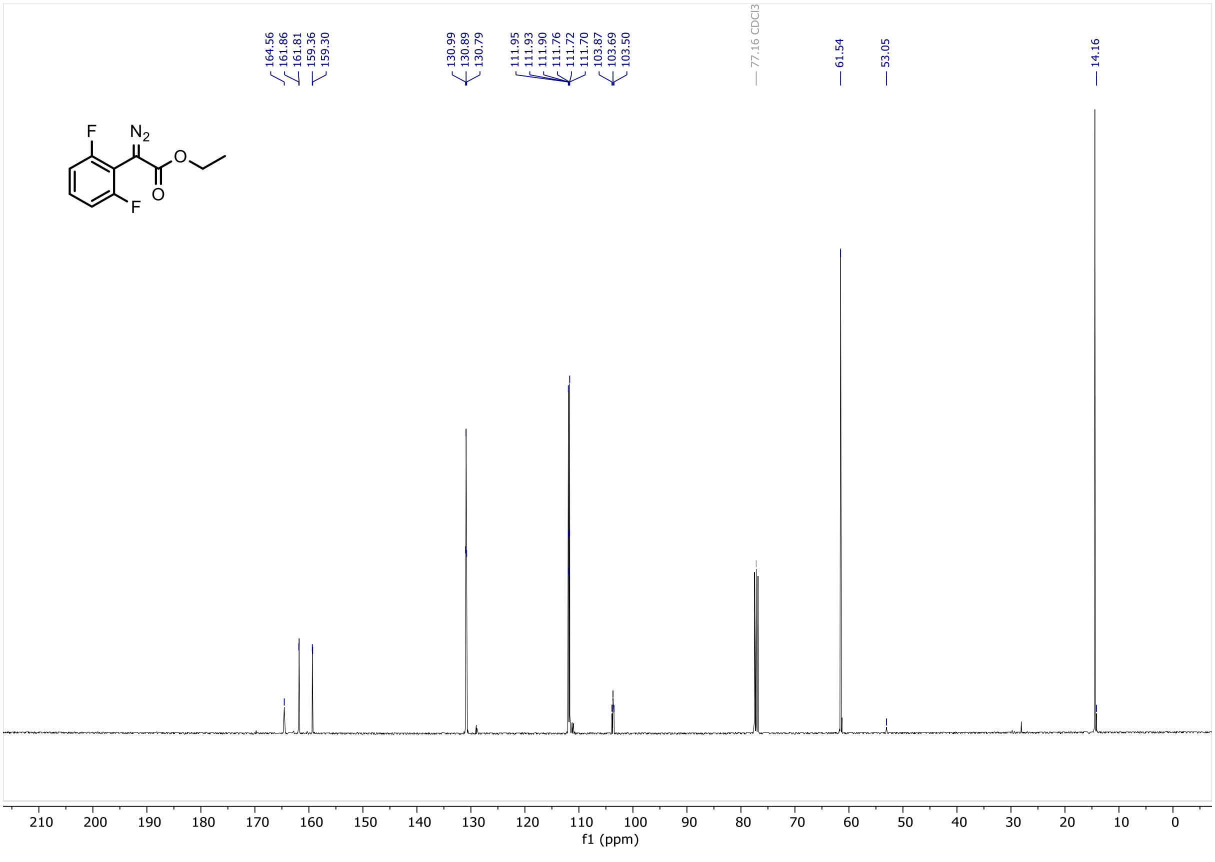

S16: ^{19}F NMR (282 MHz, $CDCl_3$)

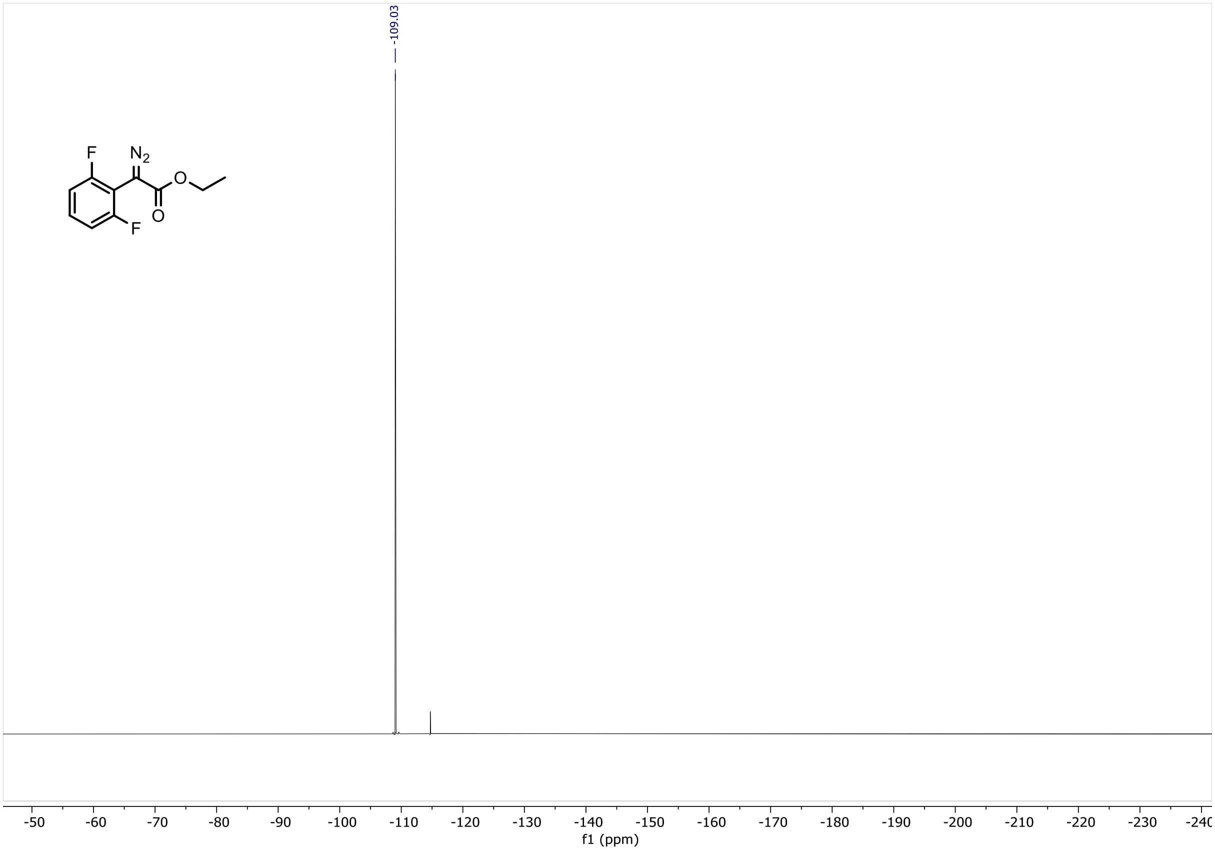

4a: ^{1}H NMR (600 MHz, $CDCl_3$)

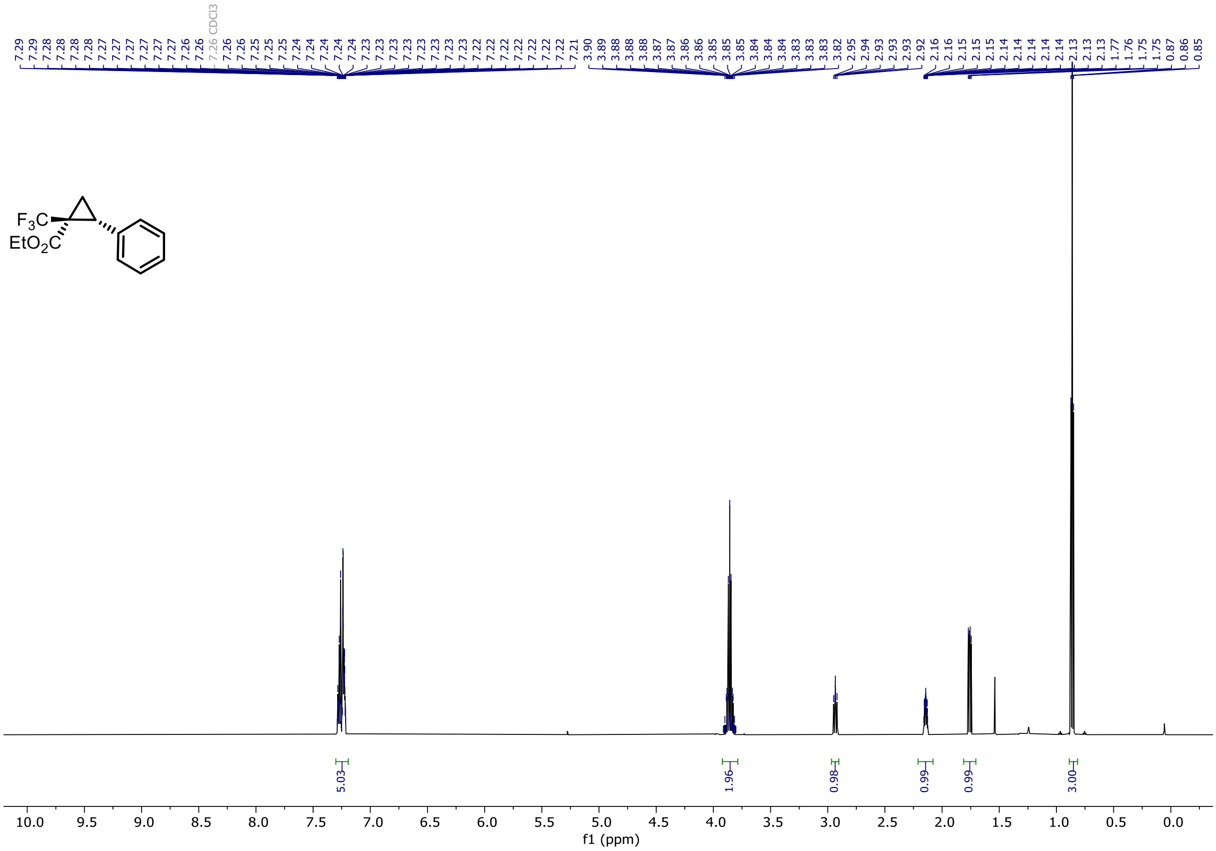

4a: ^{13}C NMR (151 MHz, $CDCl_3$)

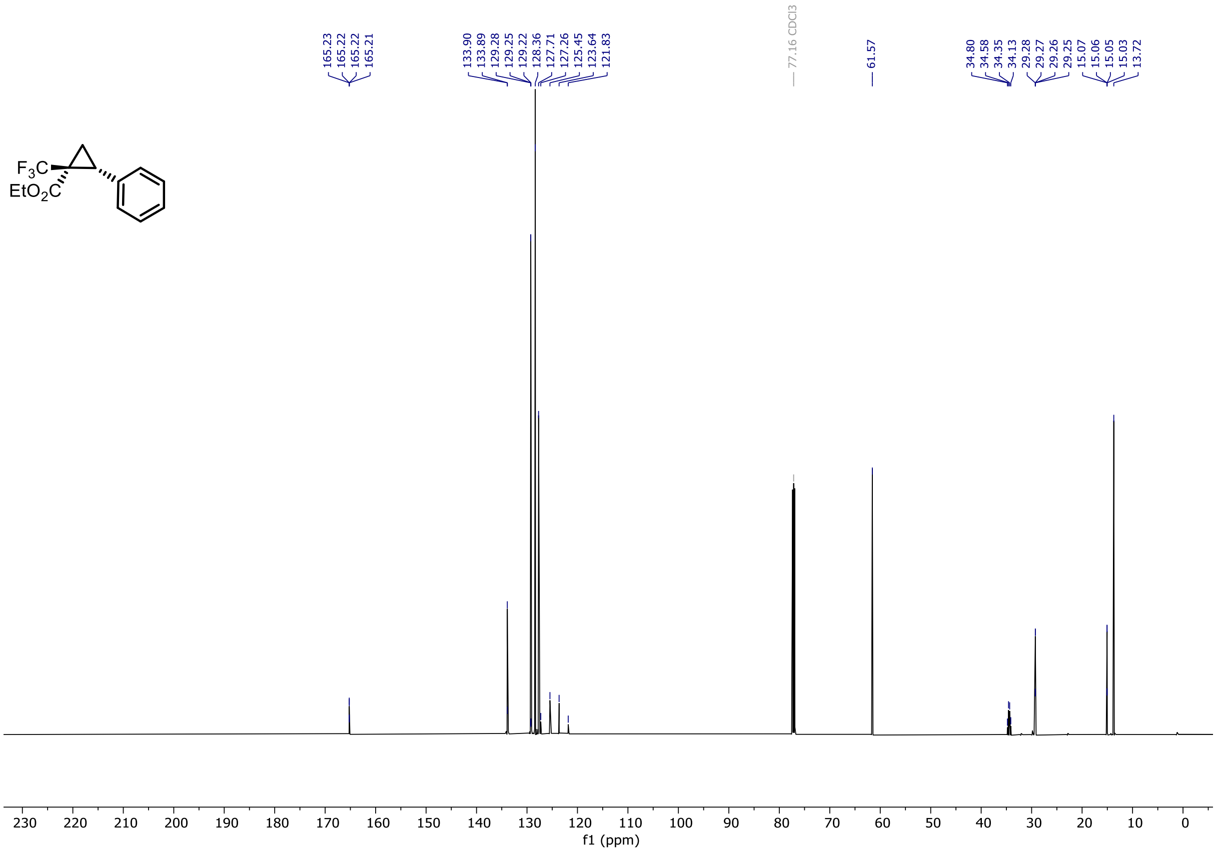

4a: ^{19}F NMR (565 MHz, $CDCl_3$)

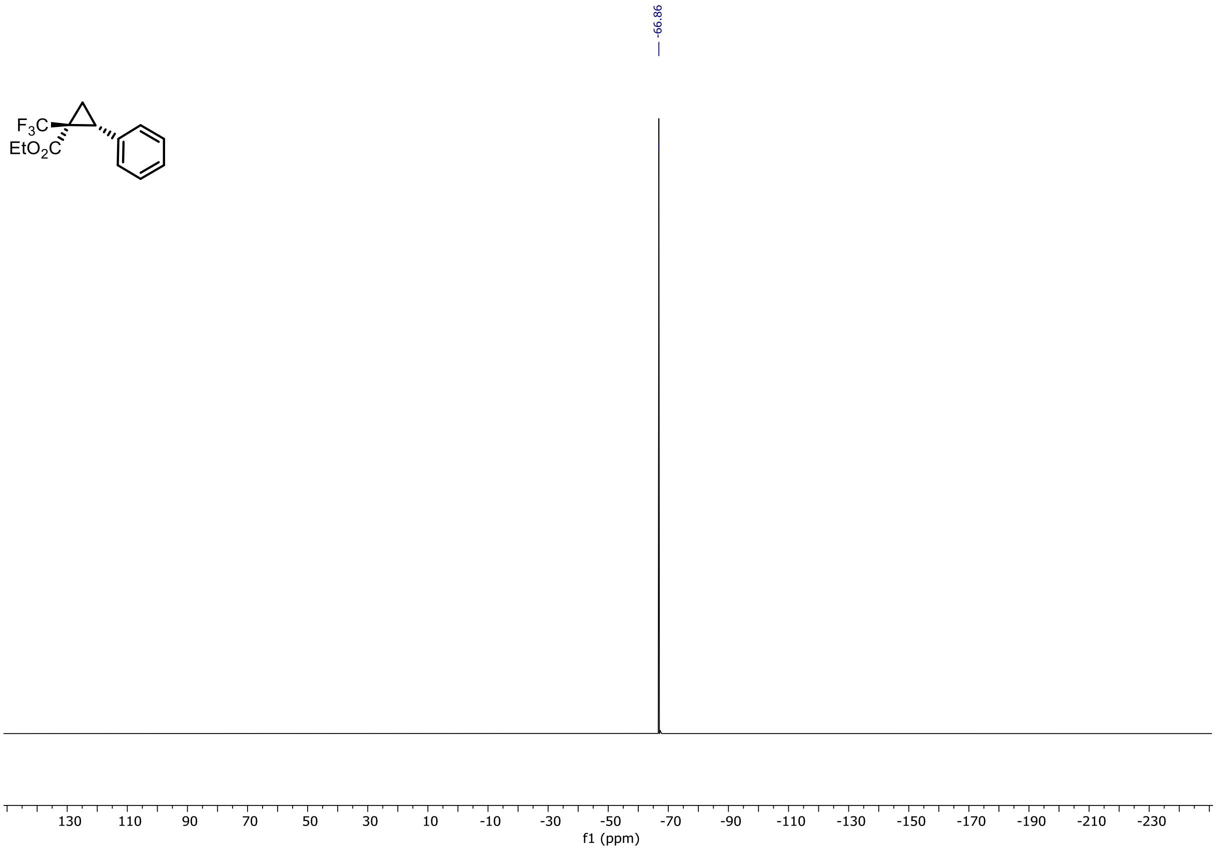

4a: ^{1}H-^{1}H NOESY ($CDCl_3$)

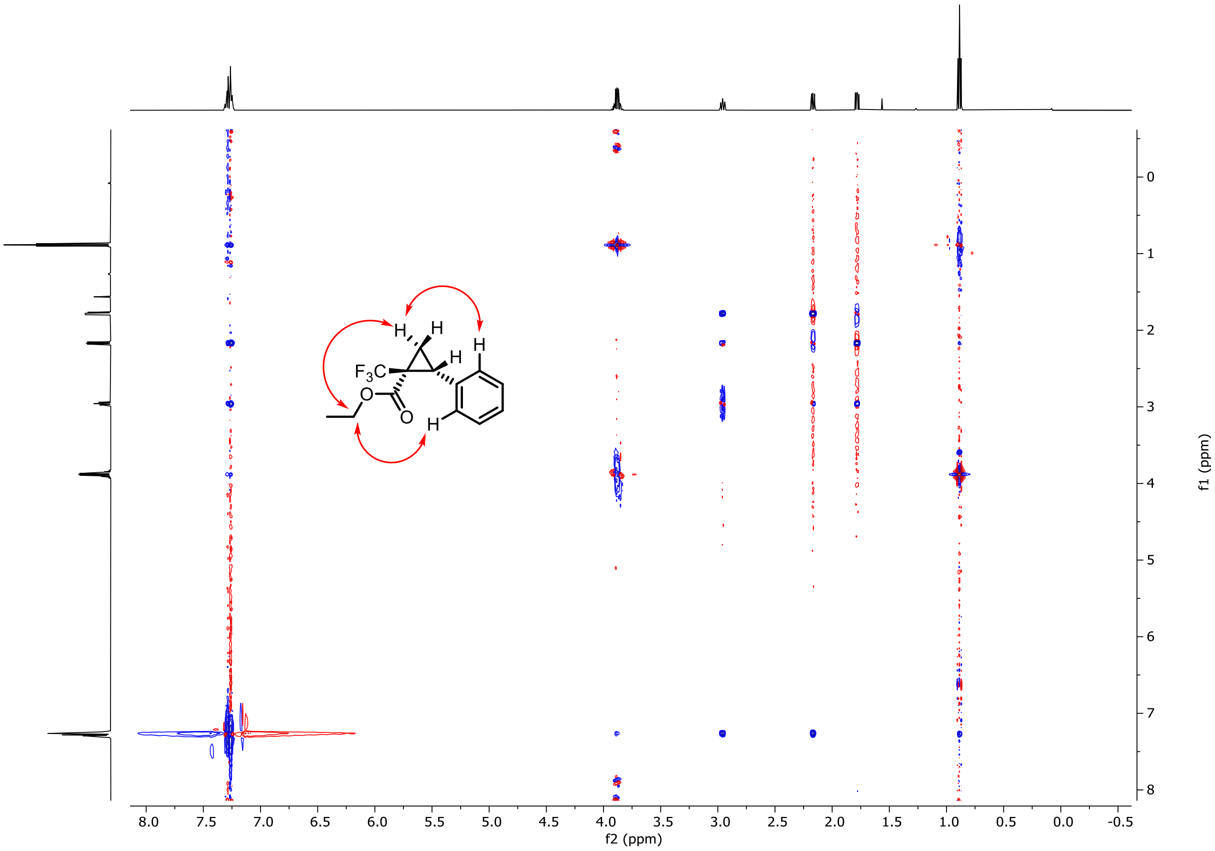

4a: ^{1}H-^{19}F HOESY ($CDCl_3$)

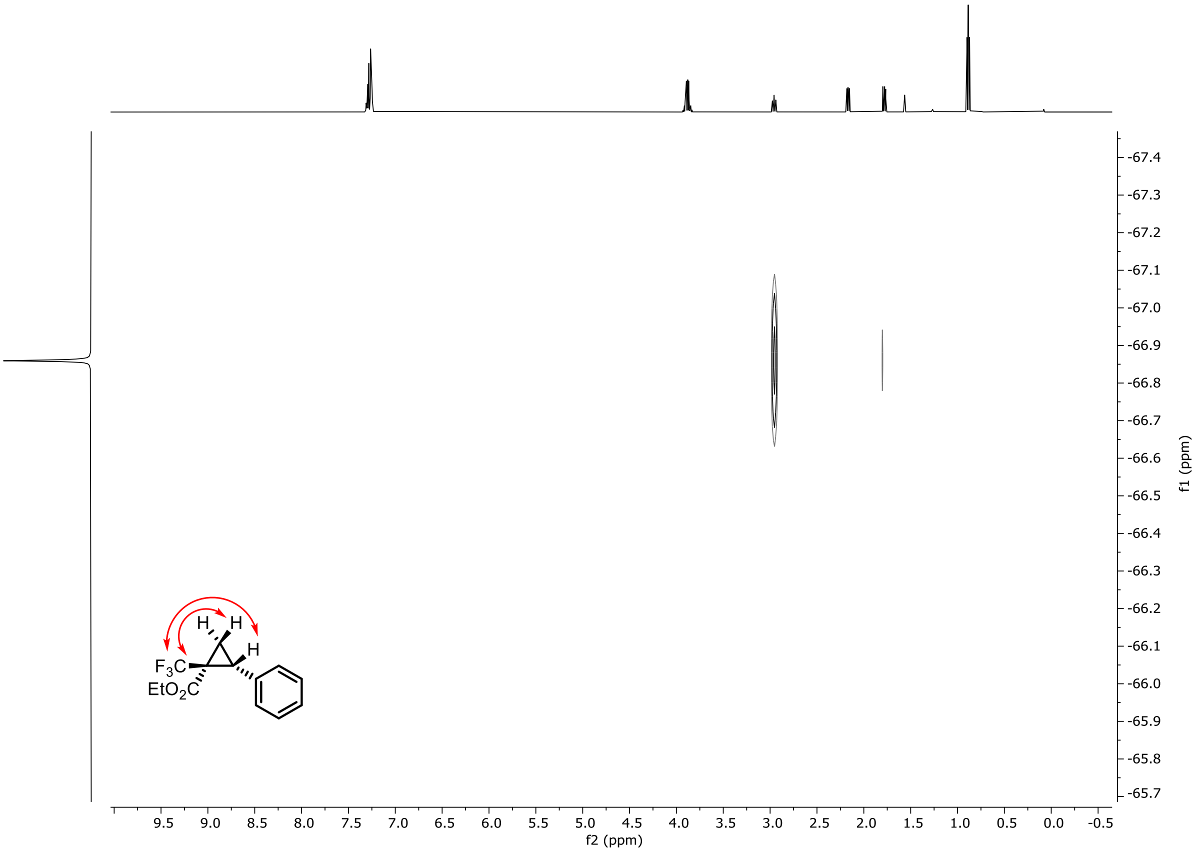

4b (mixture of diastereomers, dr ≈ 6:1): ^{1}H NMR (400 MHz, $CDCl_3$)

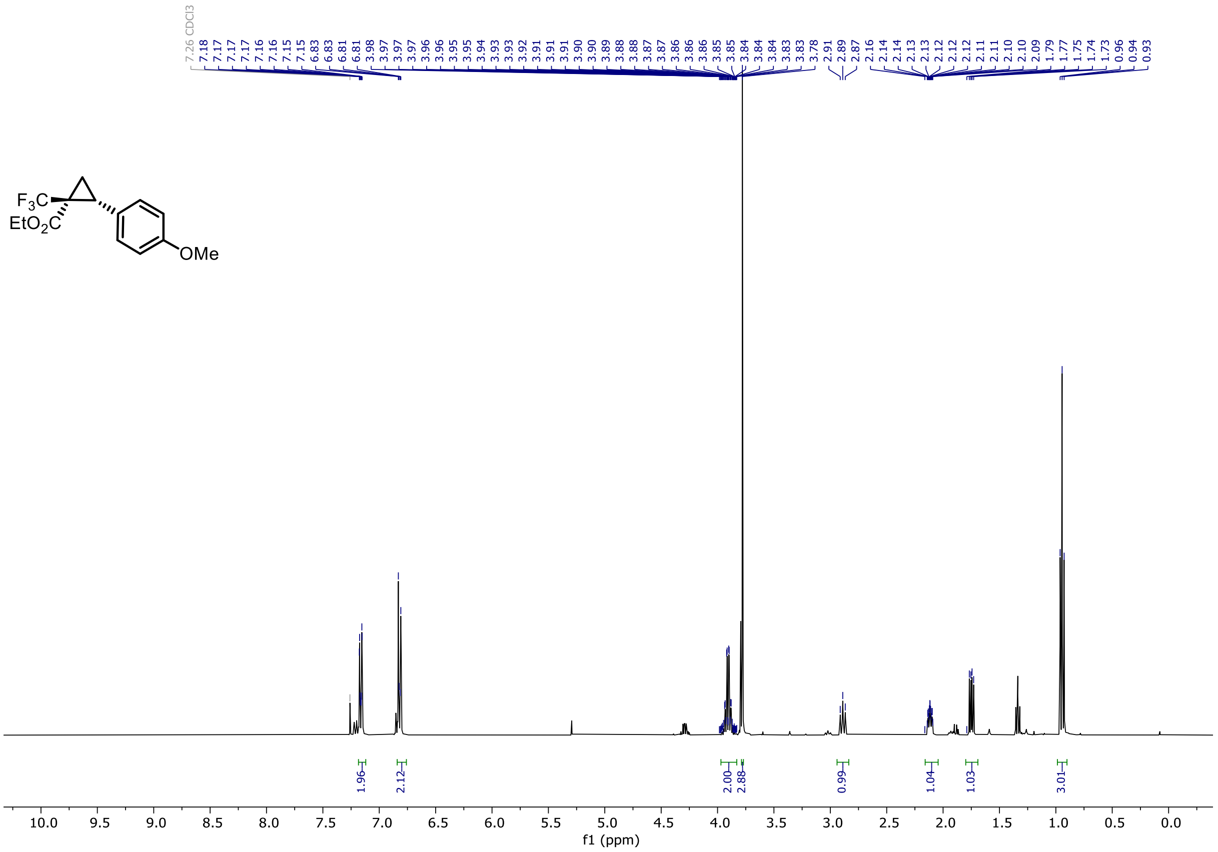

4b (mixture of diastereomers, dr ≈ 6:1): ^{13}C NMR (101 MHz, $CDCl_3$)

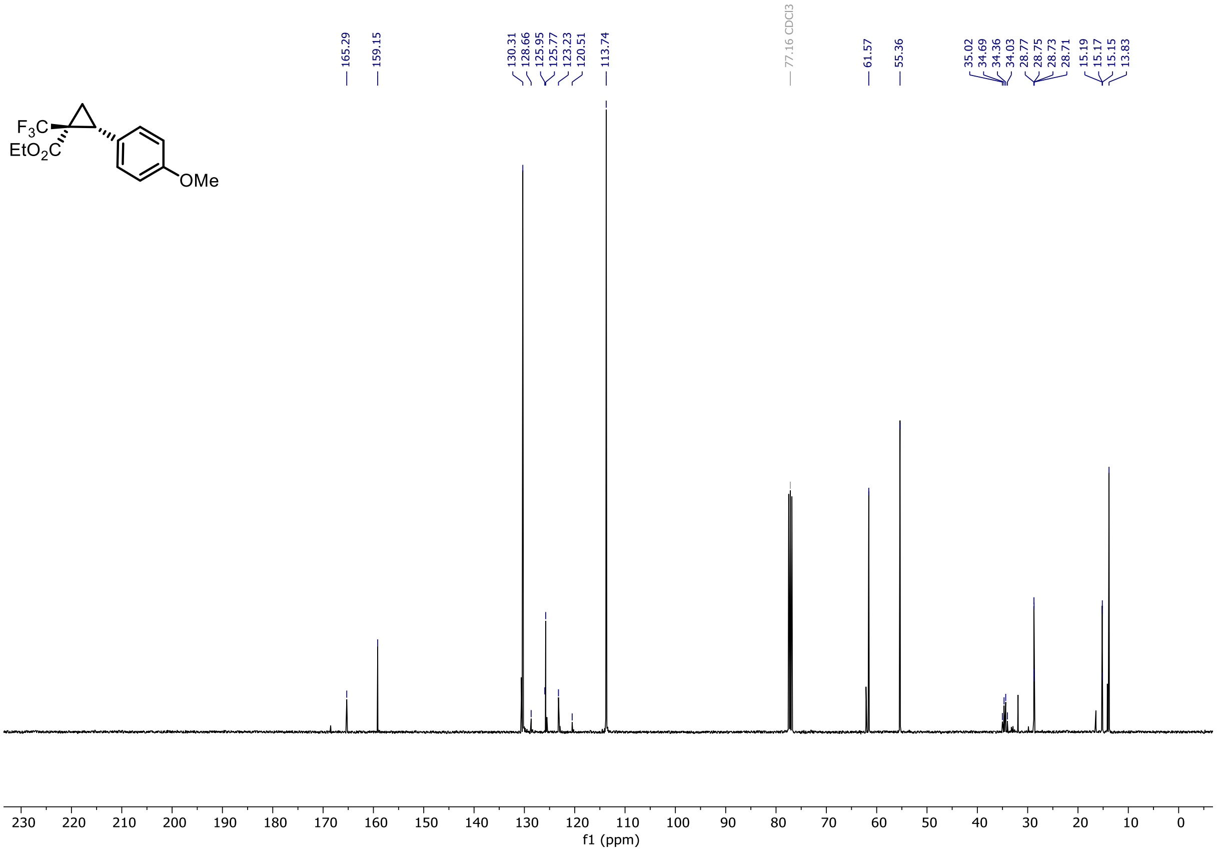

4b (mixture of diastereomers, dr ≈ 6:1): ^{19}F NMR (282 MHz, $CDCl_3$)

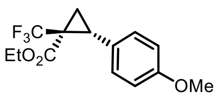

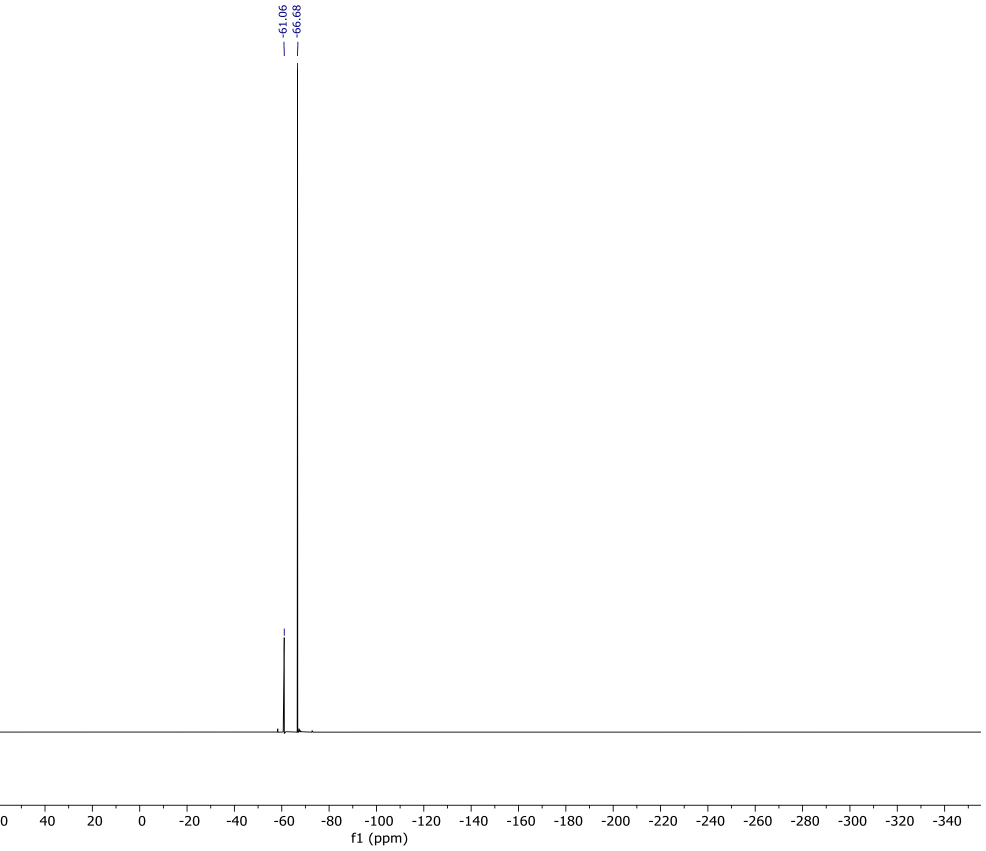

4c: ^{1}H NMR (600 MHz, $CDCl_3$)

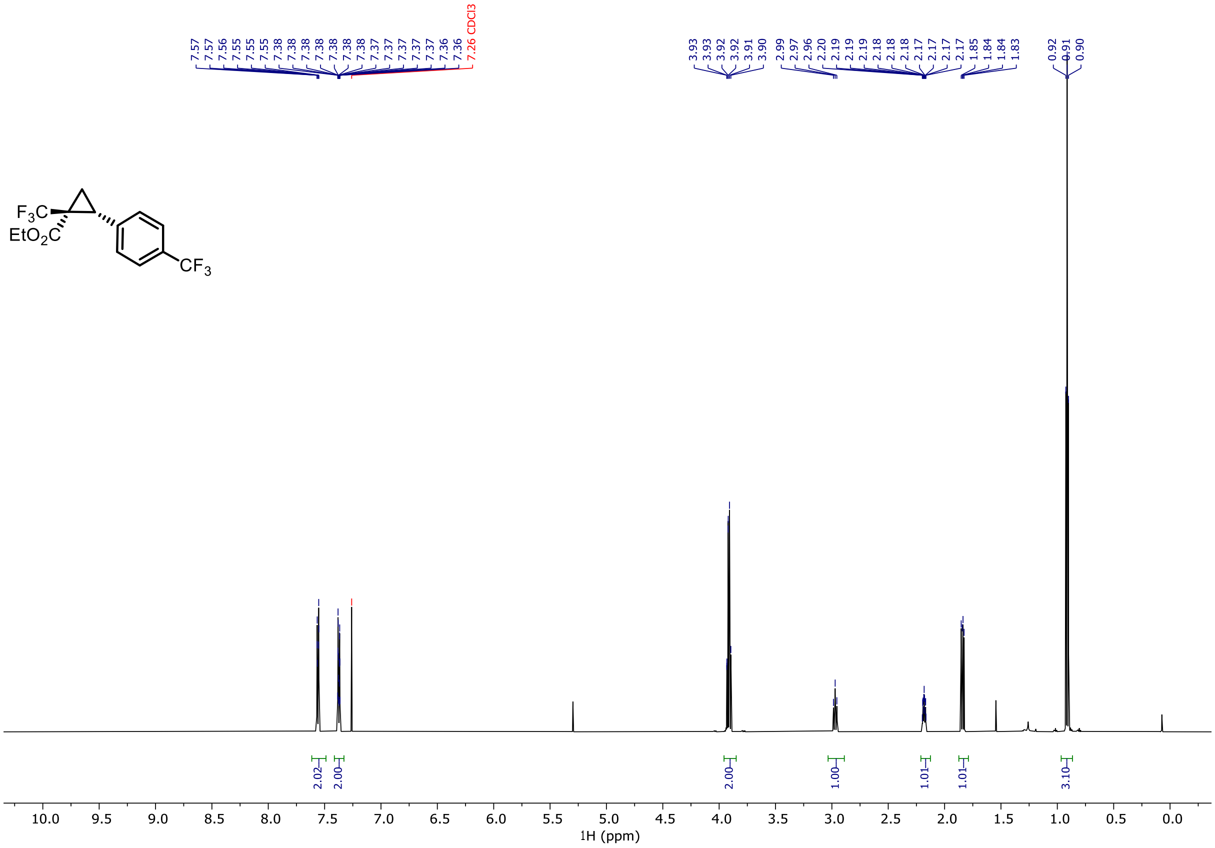

4c: ^{13}C NMR (151 MHz, $CDCl_3$)

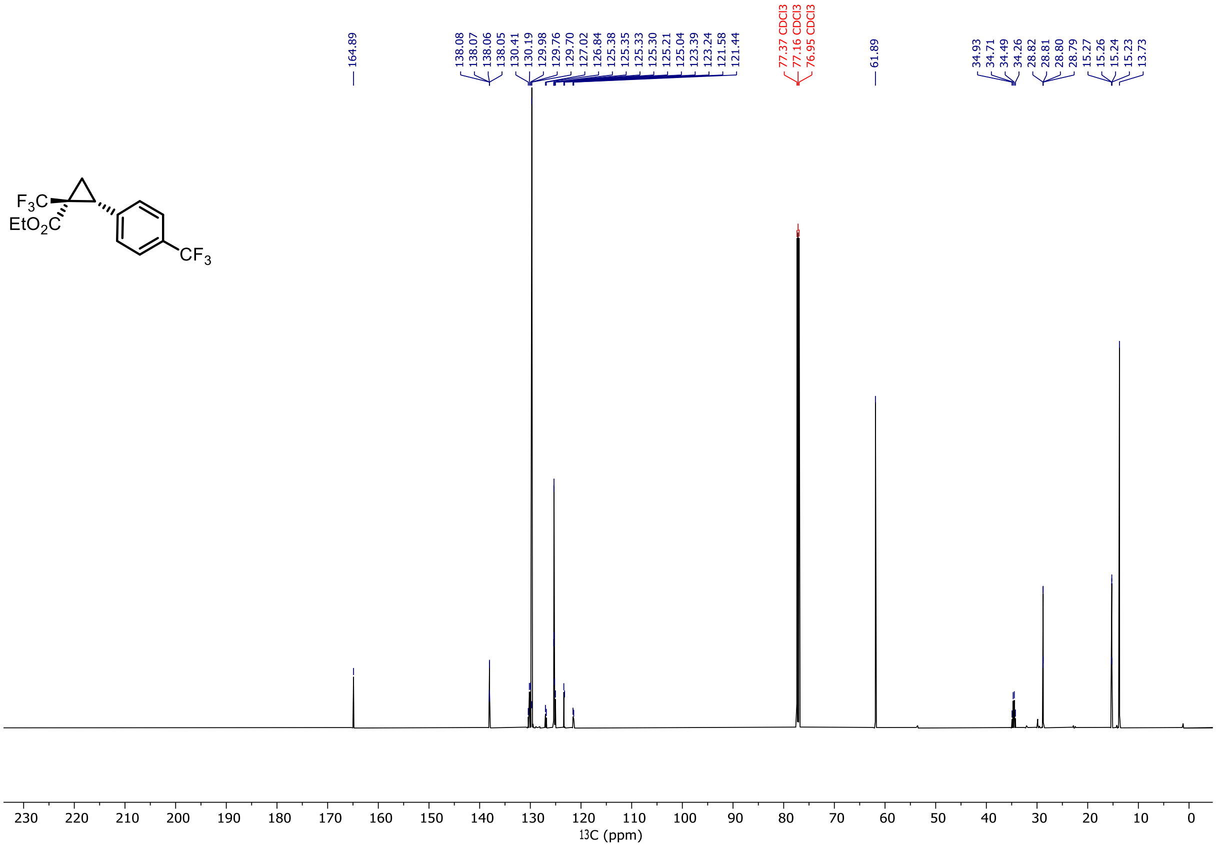

4c: ^{19}F NMR (565 MHz, $CDCl_3$)

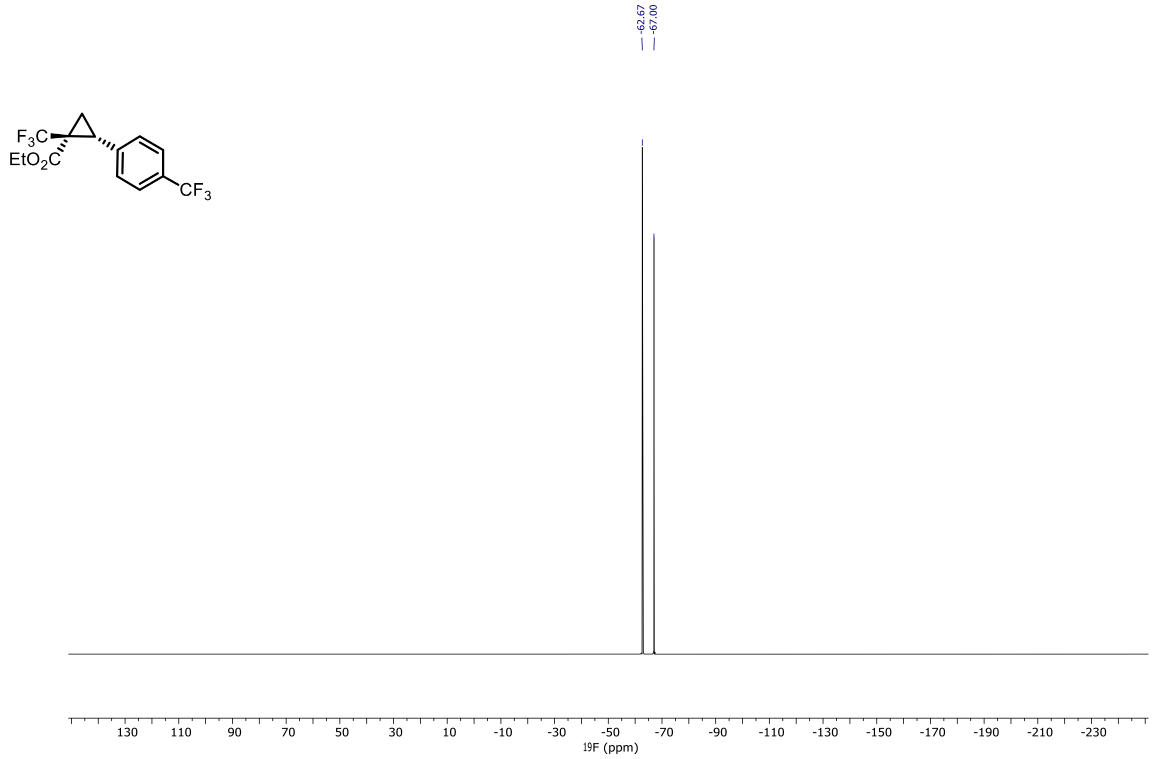

4c: ^{1}H-^{1}H NOESY ($CDCl_3$)

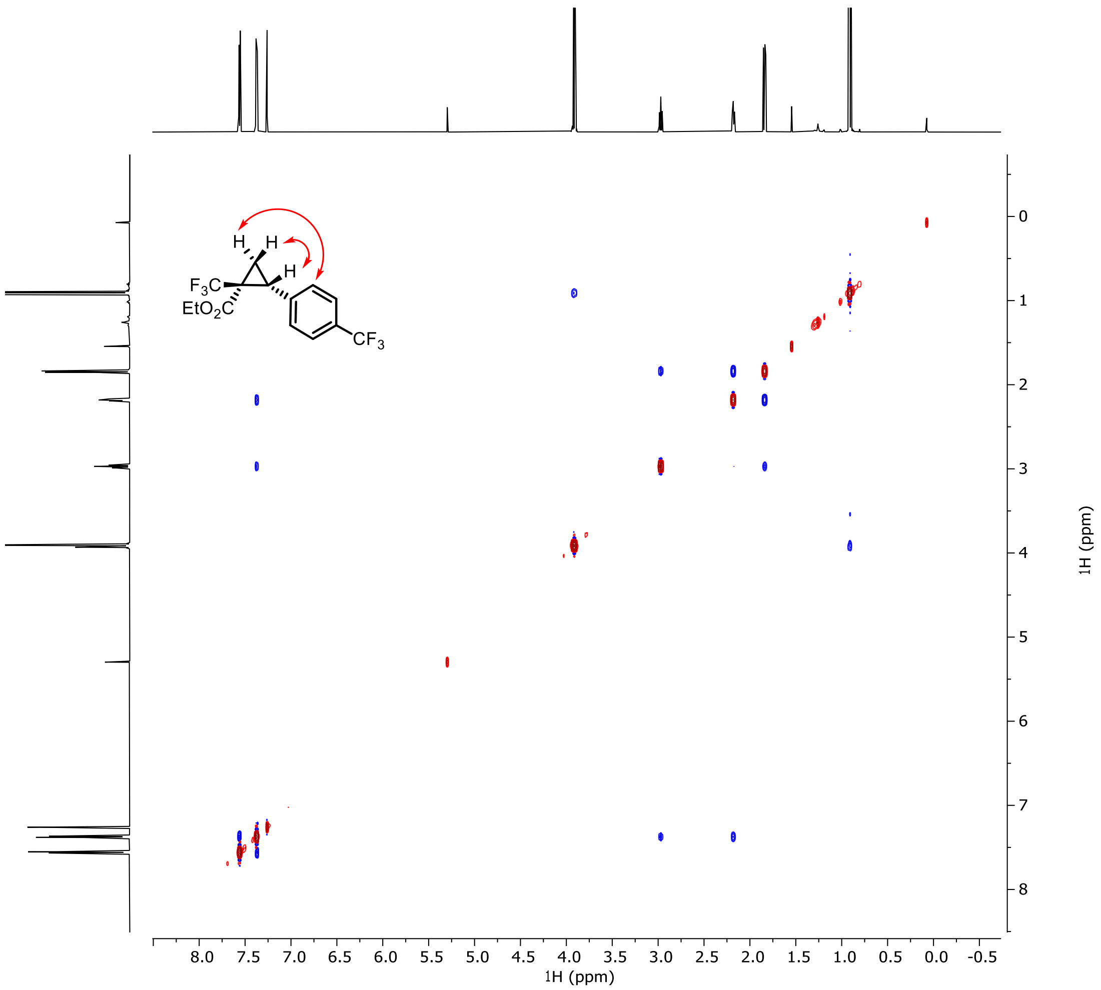

4c: ^1H-^{19}F HOESY ($CDCl_3$)

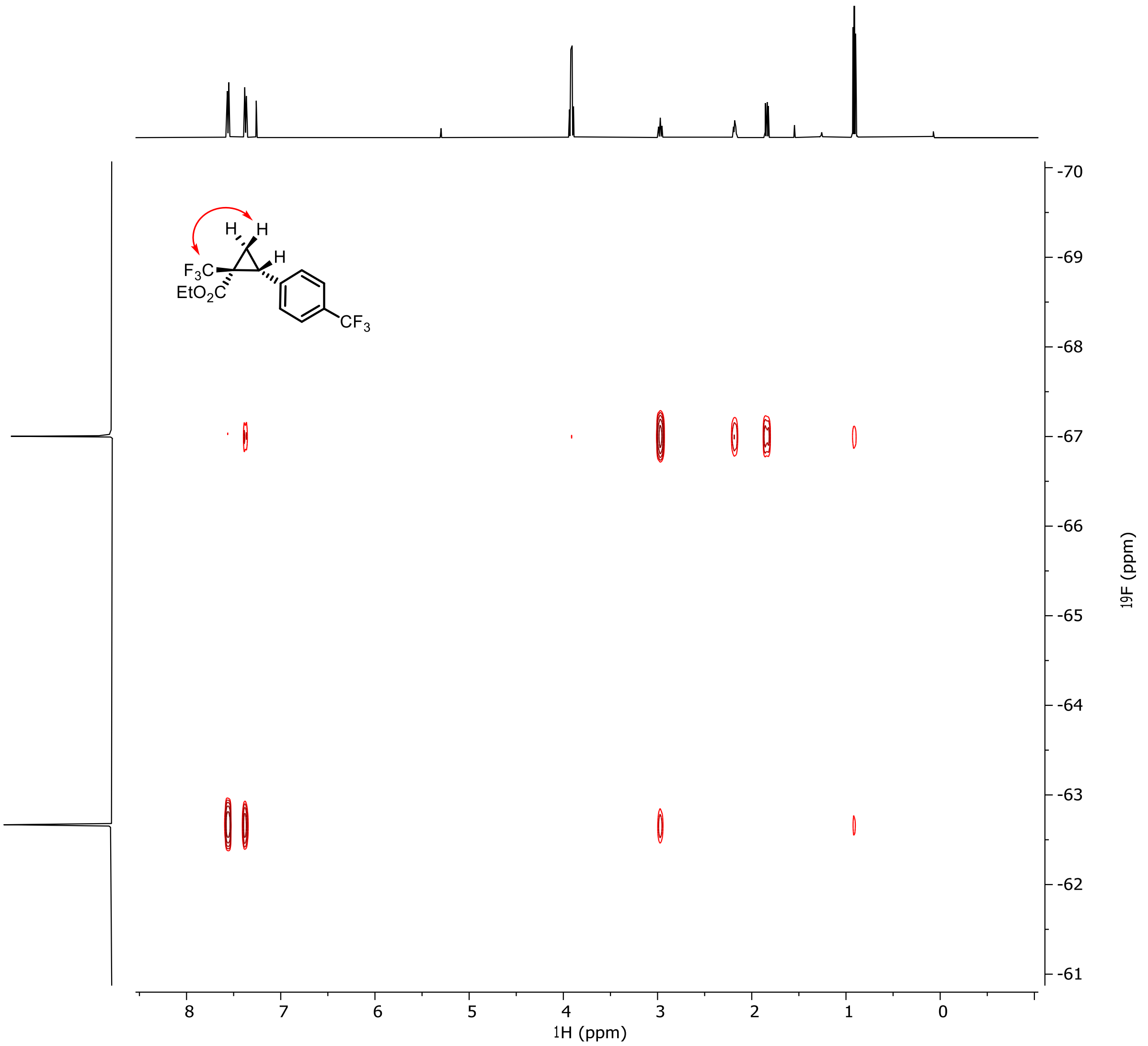

4d (mixture of diastereomers, dr ≈ 6:1): ^{1}H NMR (400 MHz, $CDCl_3$)

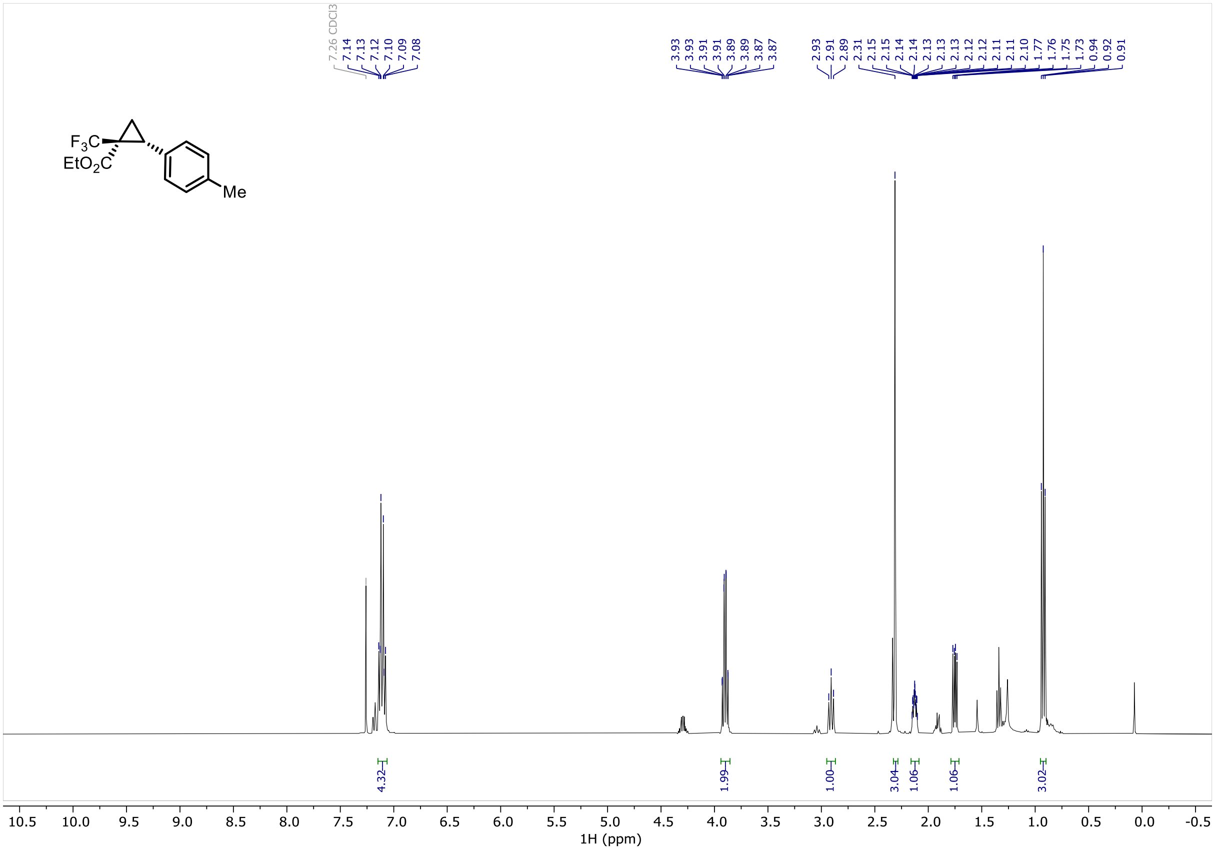

4d (mixture of diastereomers, dr ≈ 6:1): ^{13}C NMR (101 MHz, $CDCl_3$)

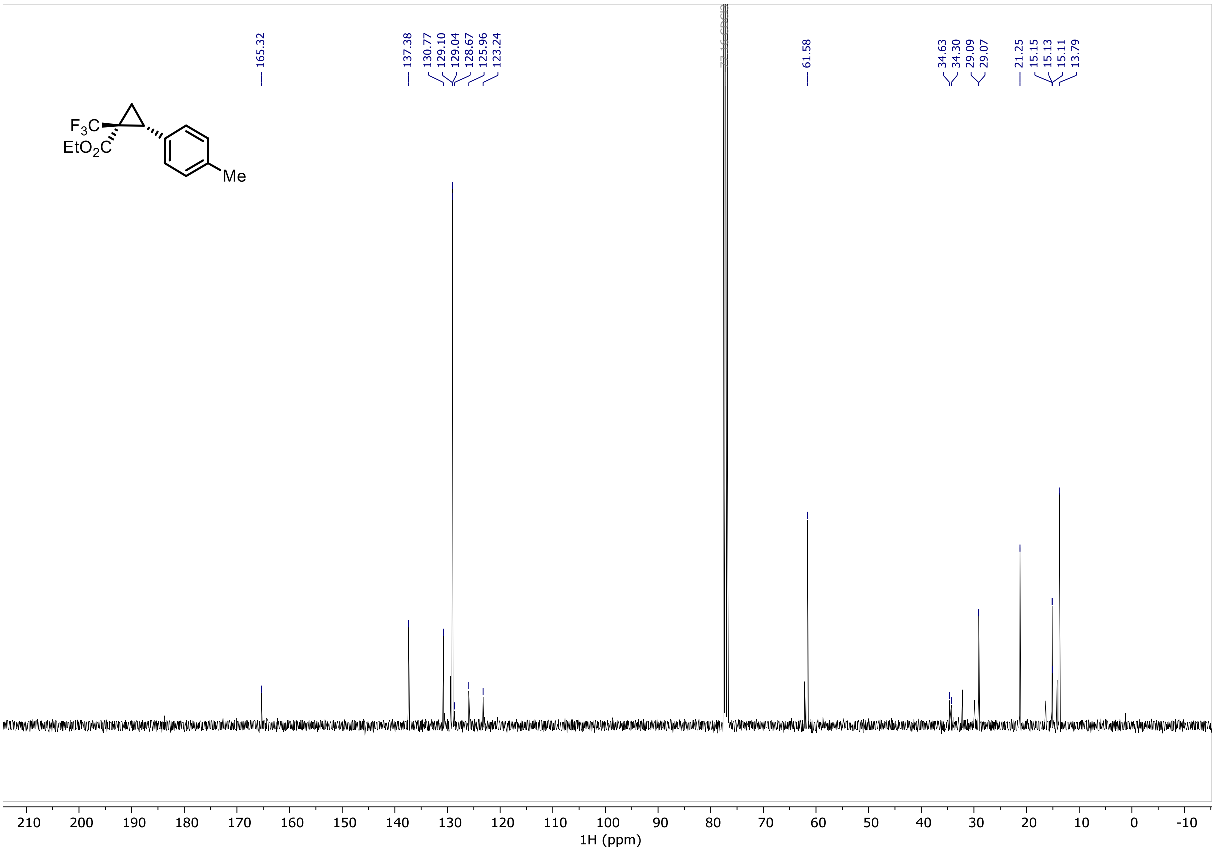

4d (mixture of diastereomers, dr ≈ 6:1): ^{19}F NMR (376 MHz, $CDCl_3$)

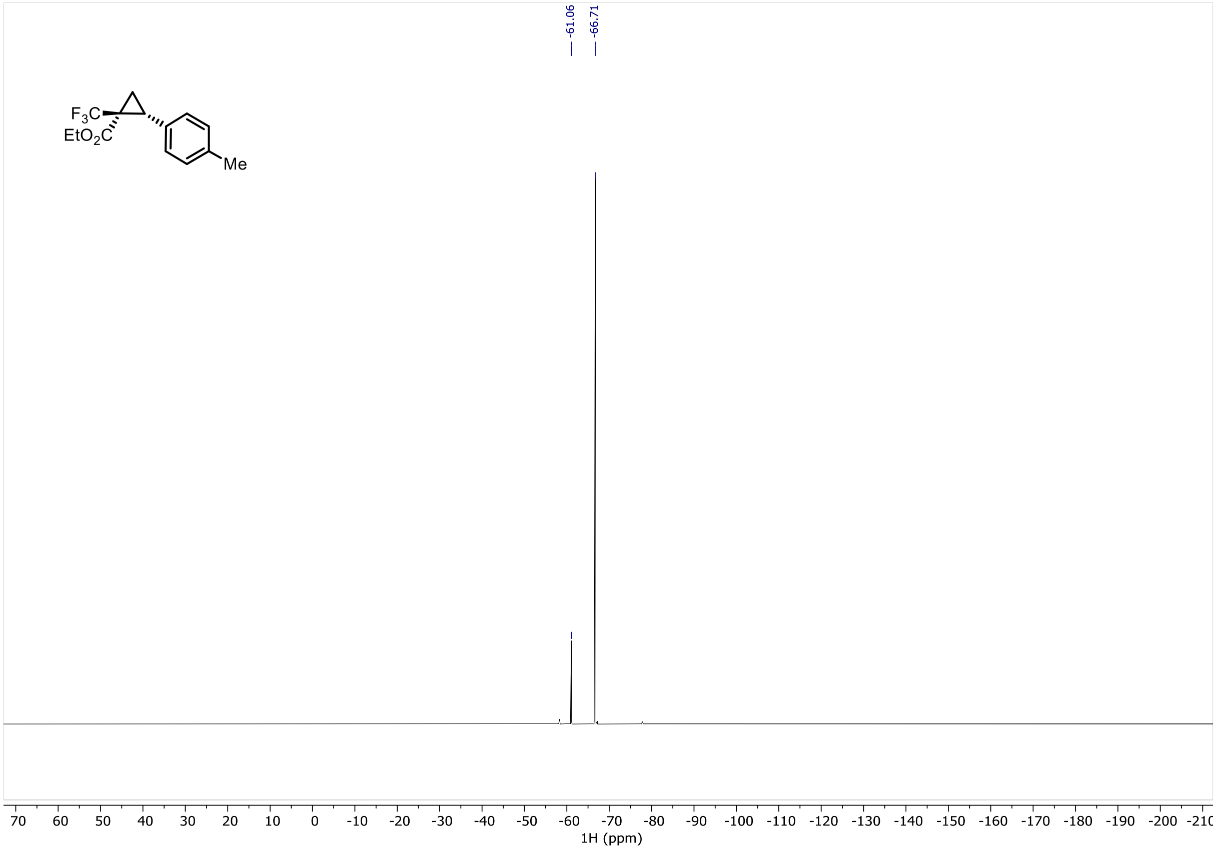

4e: ^{1}H NMR (400 MHz, $CDCl_3$)

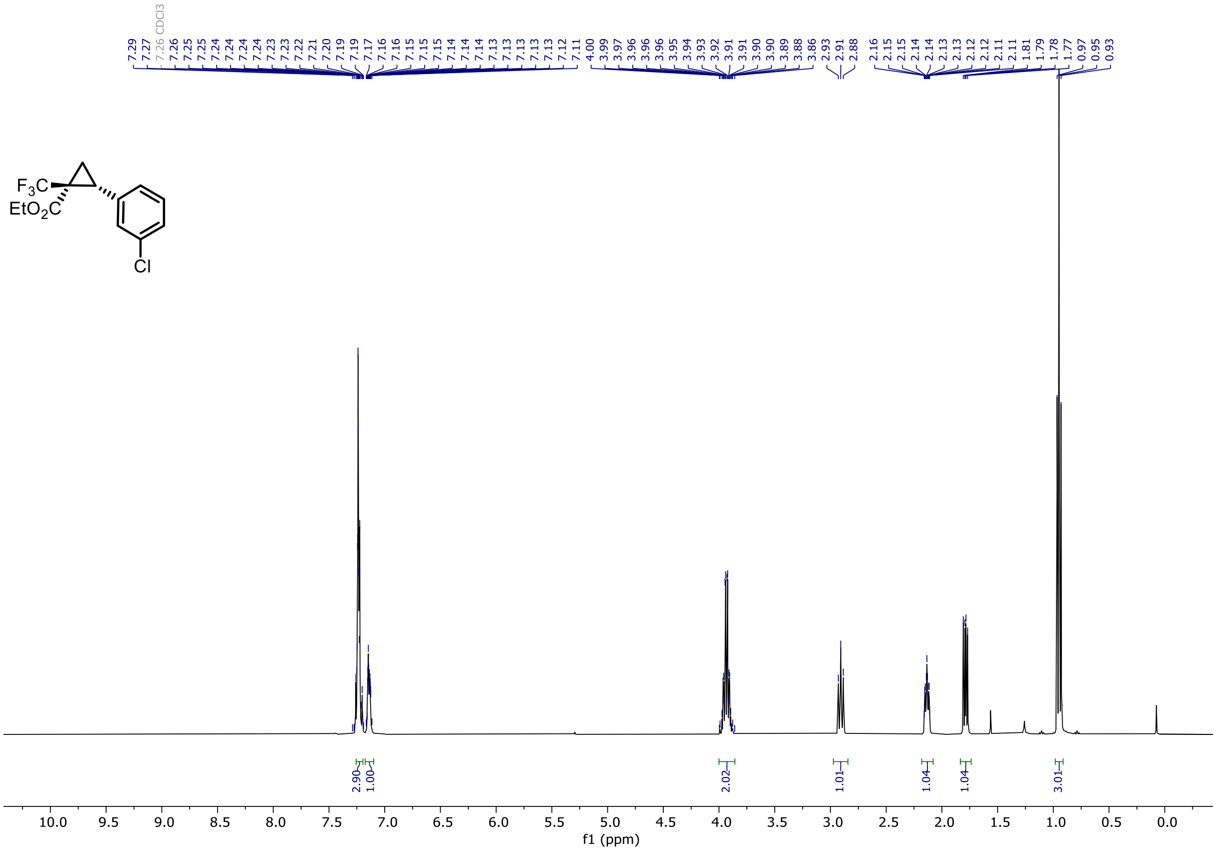

4e: ^{13}C NMR (101 MHz, $CDCl_3$)

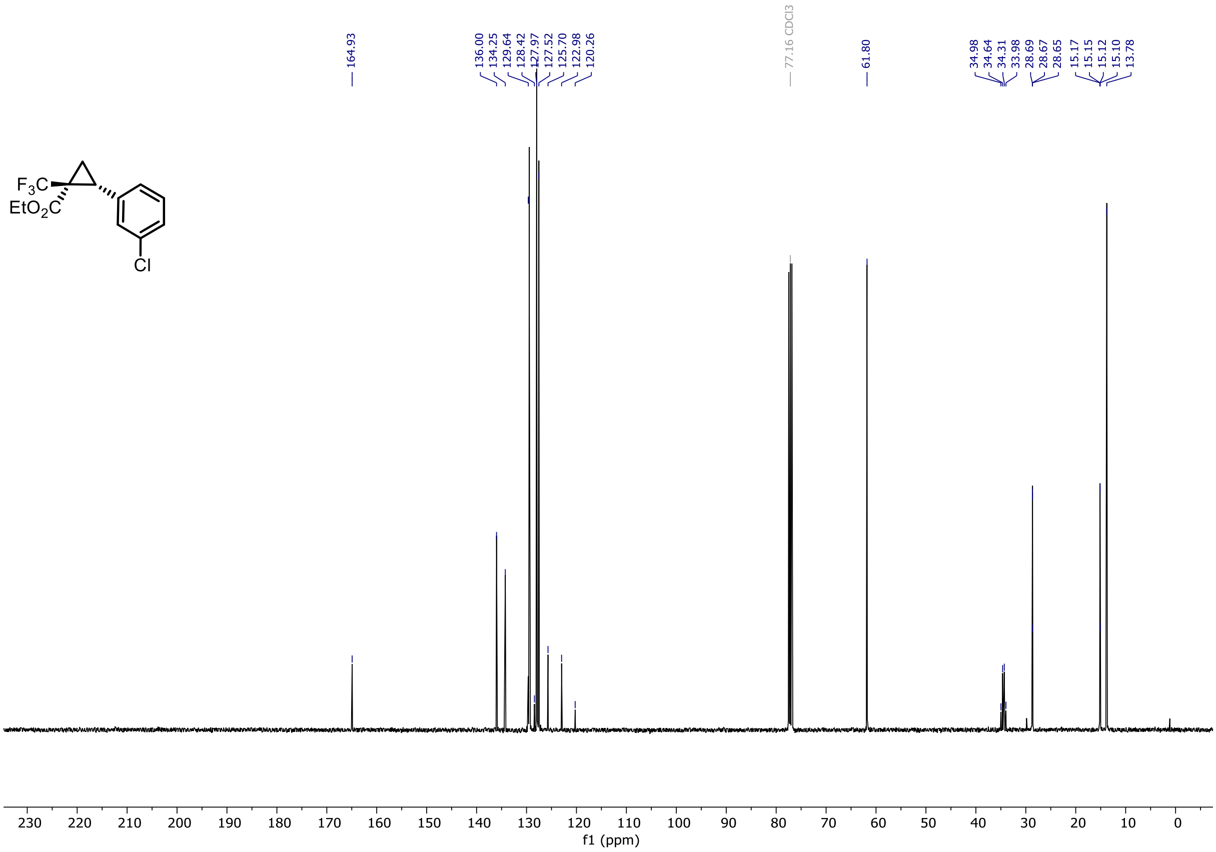

4e: ^{19}F NMR (282 MHz, $CDCl_3$)

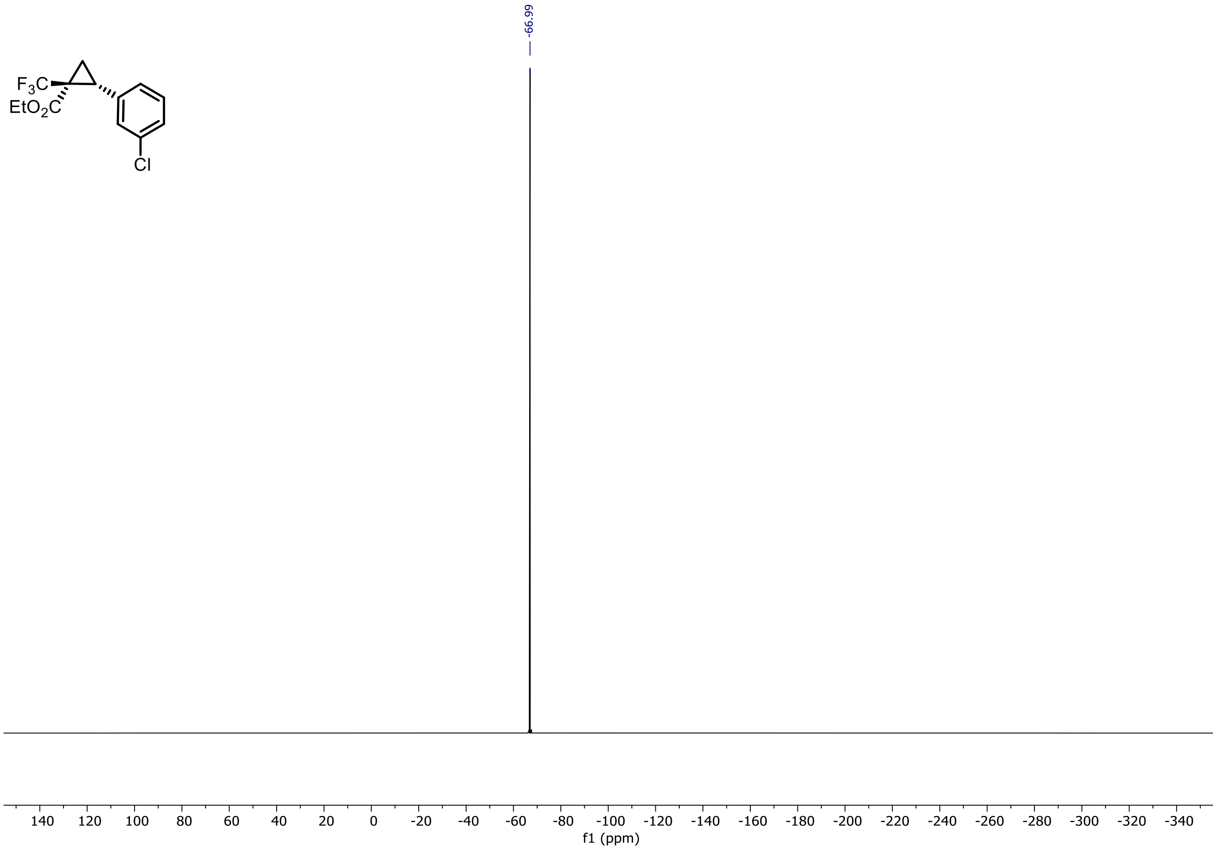

4f: ^{1}H NMR (400 MHz, $CDCl_3$)

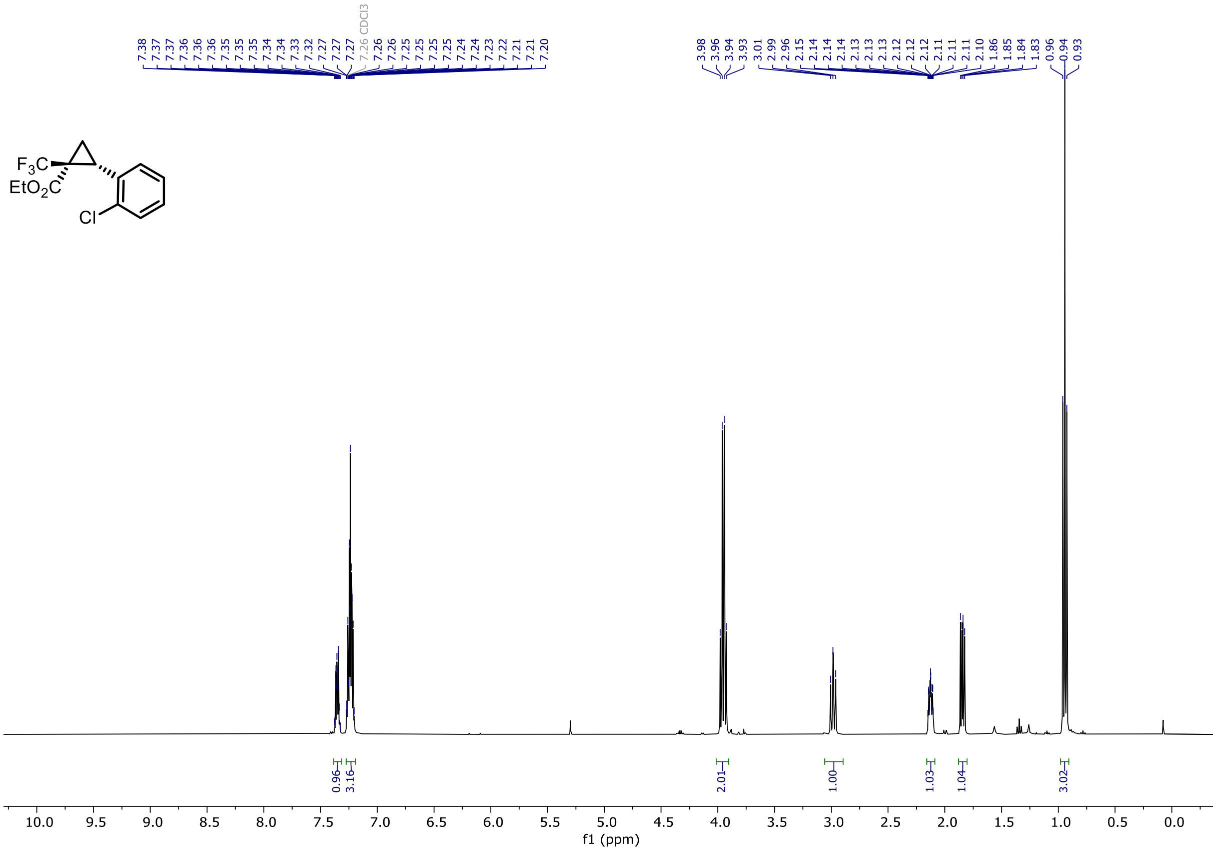

4f: ^{13}C NMR (101 MHz, $CDCl_3$)

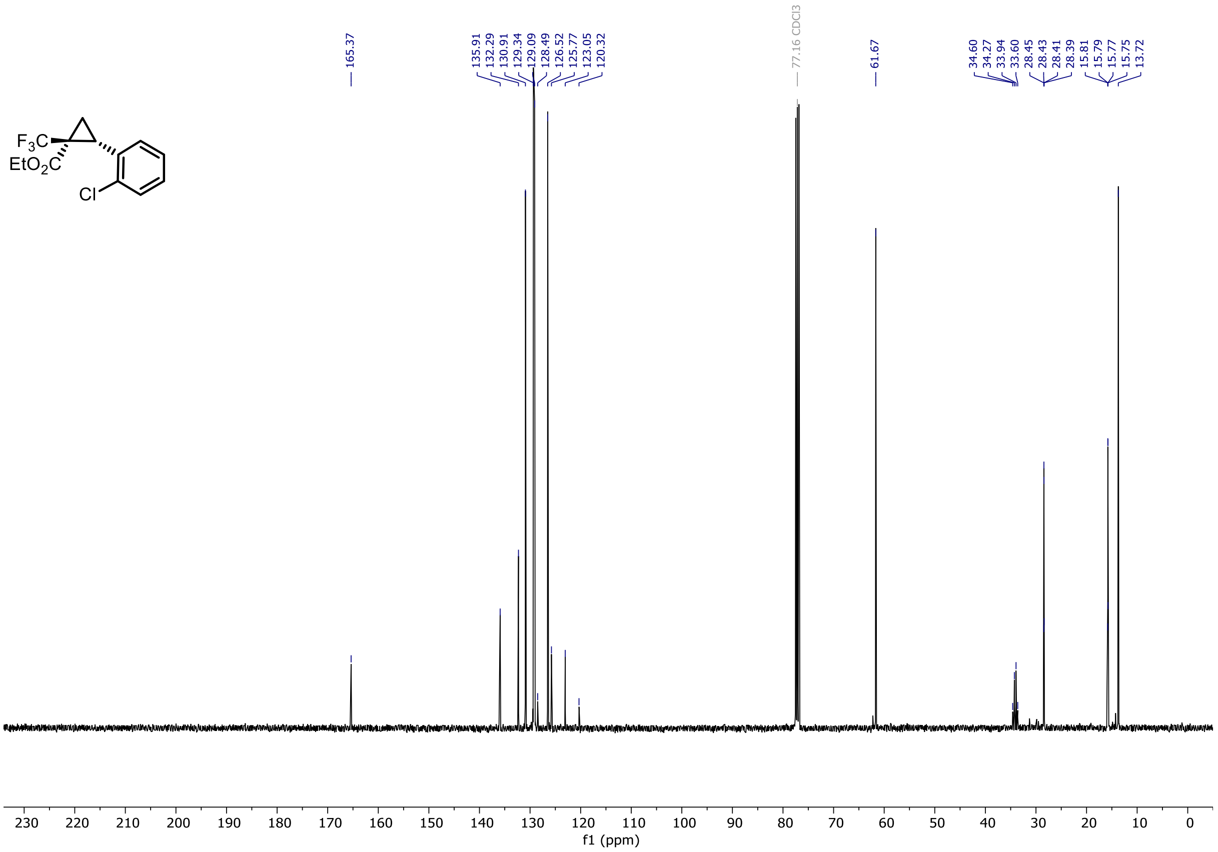

4f: ^{19}F NMR (282 MHz, $CDCl_3$)

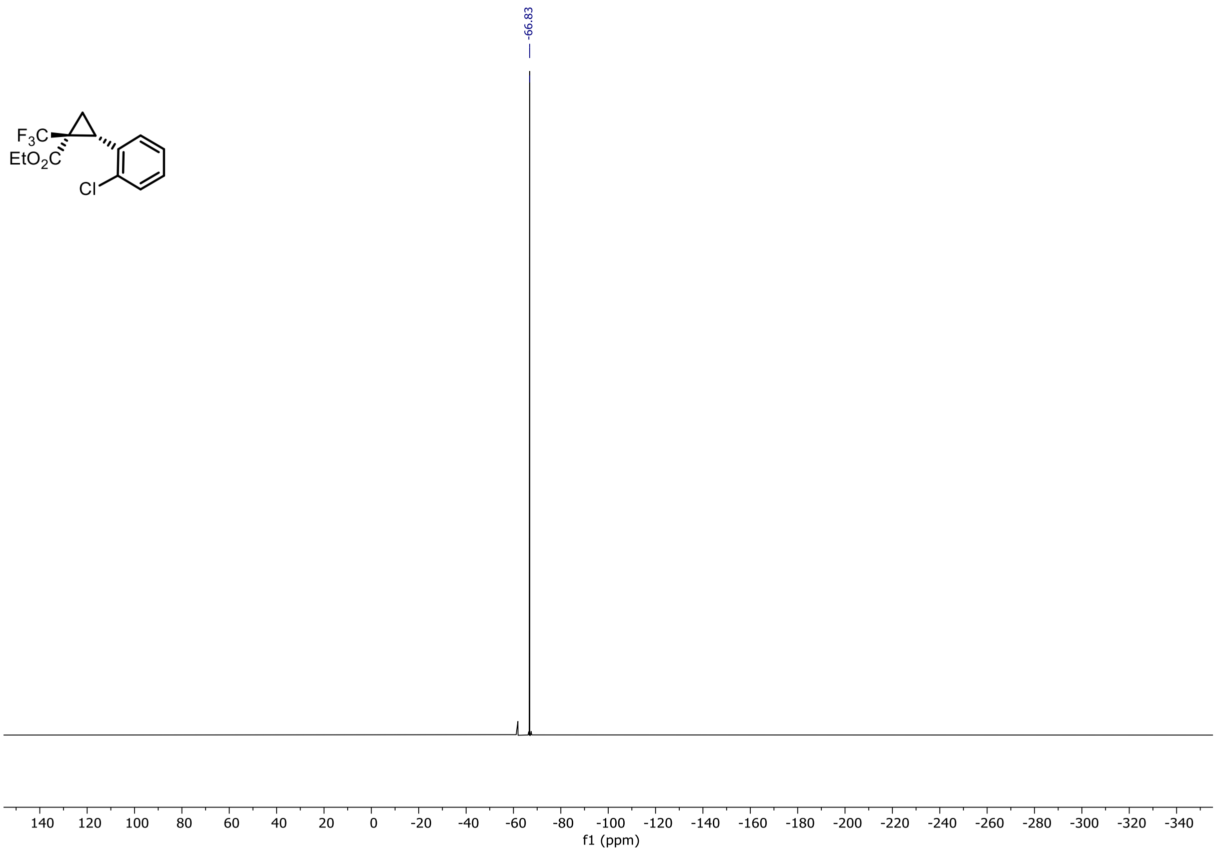

4g: ^{1}H NMR (400 MHz, $CDCl_3$)

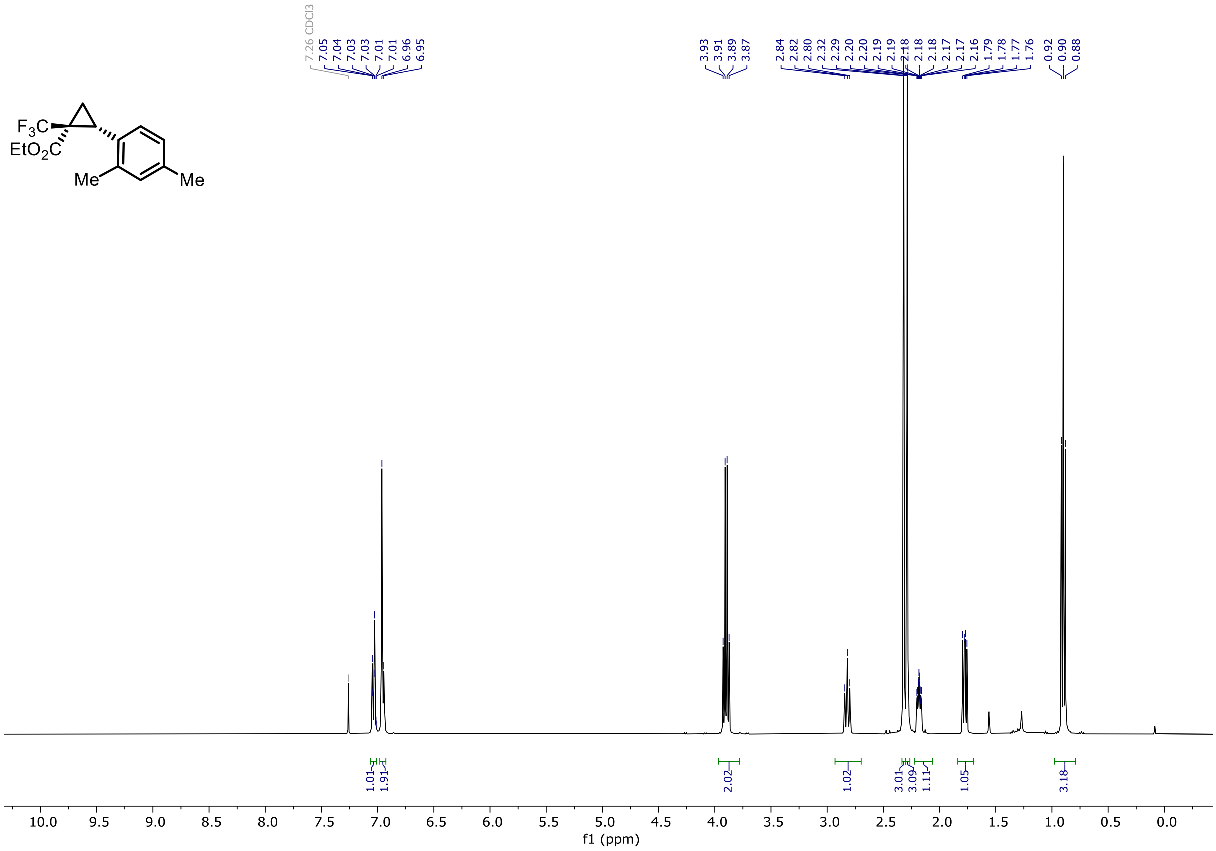

4g: ^{13}C NMR (101 MHz, $CDCl_3$)

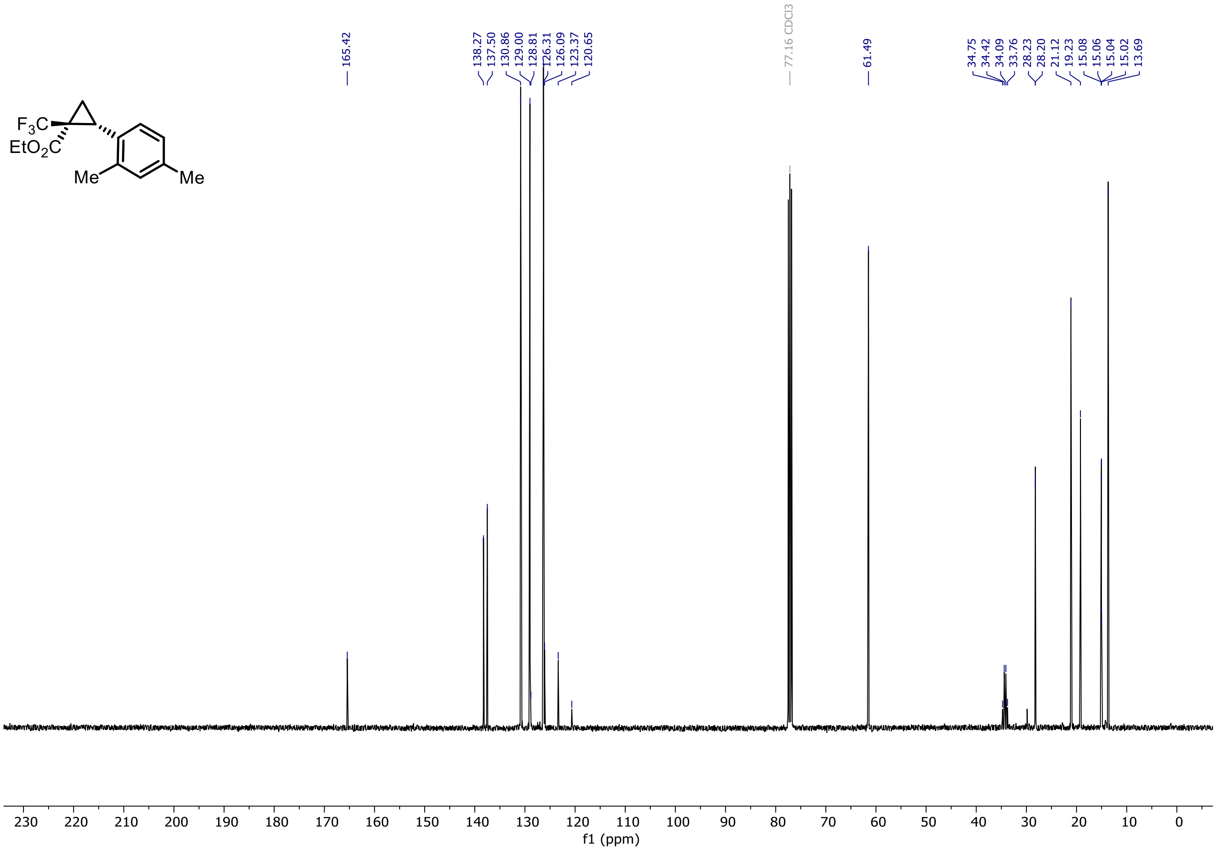

4g: ^{19}F NMR (282 MHz, $CDCl_3$)

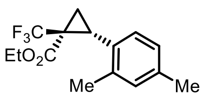

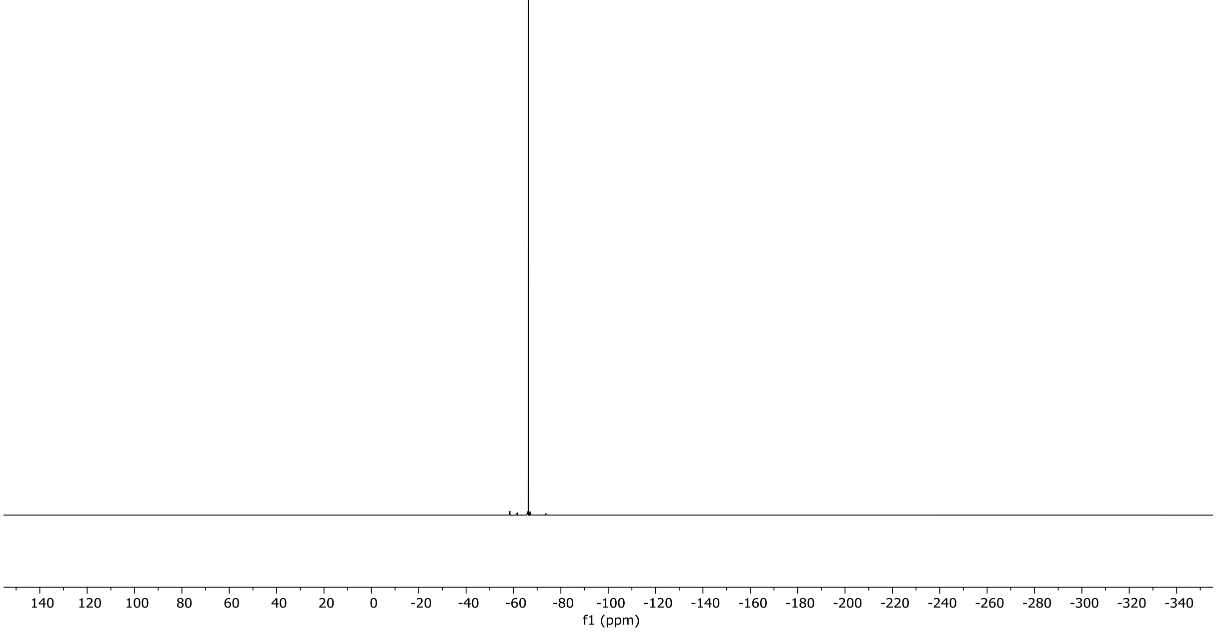

4h: ^{1}H NMR (600 MHz, $CDCl_3$)

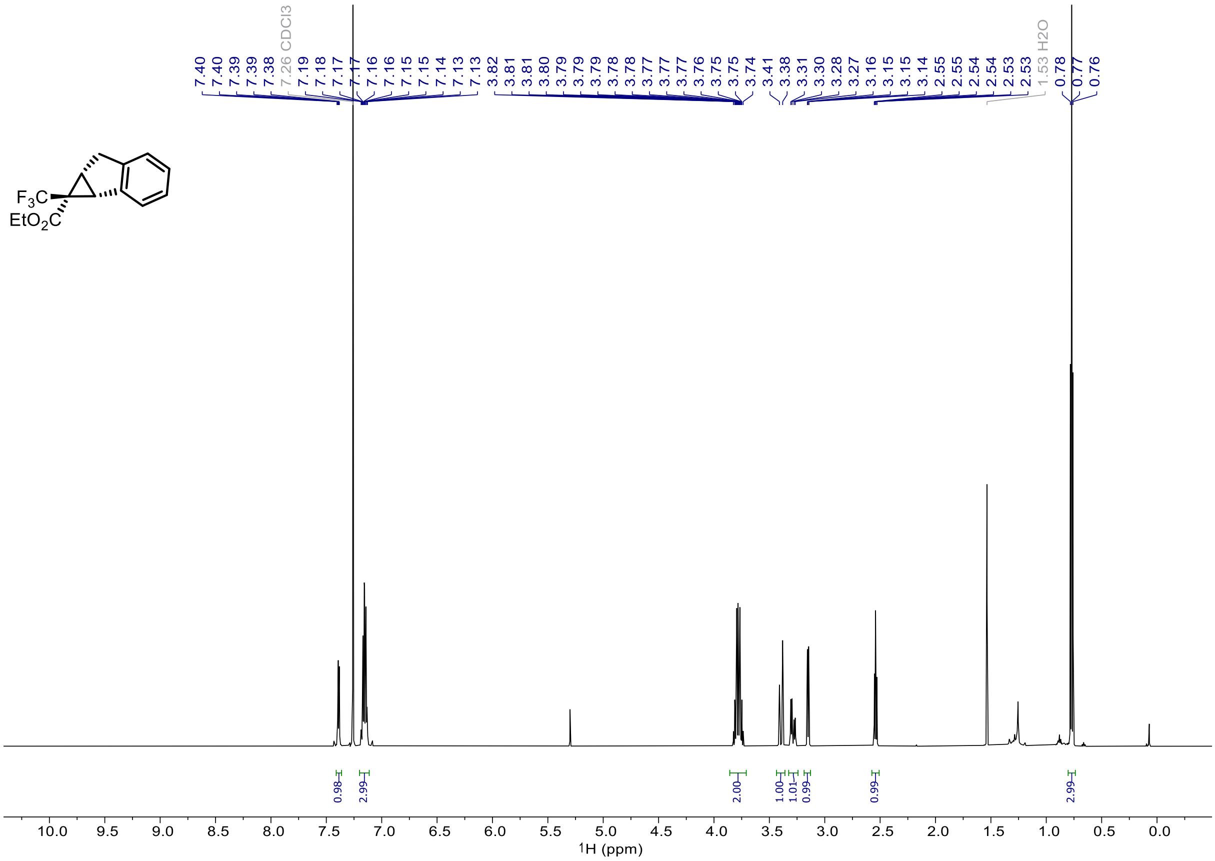

4h: ^{13}C NMR (151 MHz, $CDCl_3$)

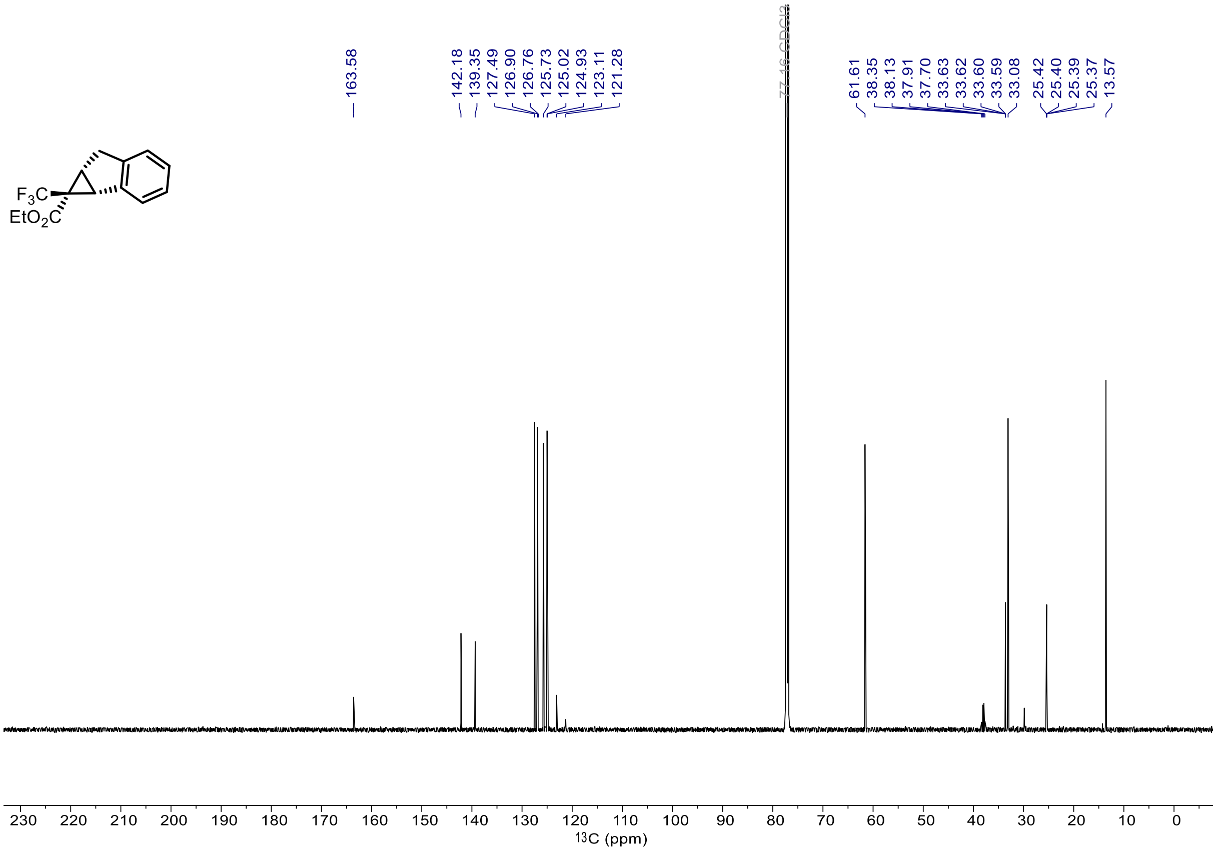

4h: ^{19}F NMR (565 MHz, $CDCl_3$)

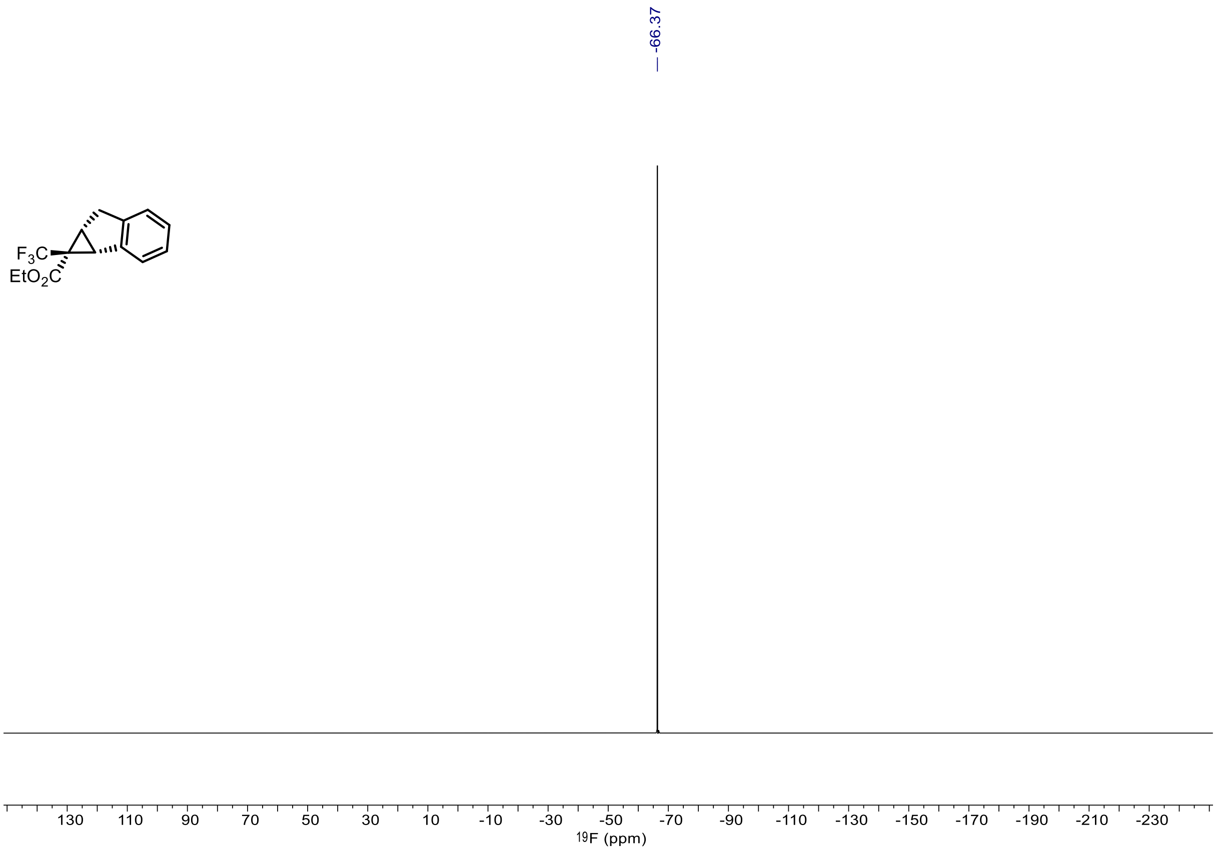

4h: ^{1}H-^{19}F HOESY ($CDCl_3$)

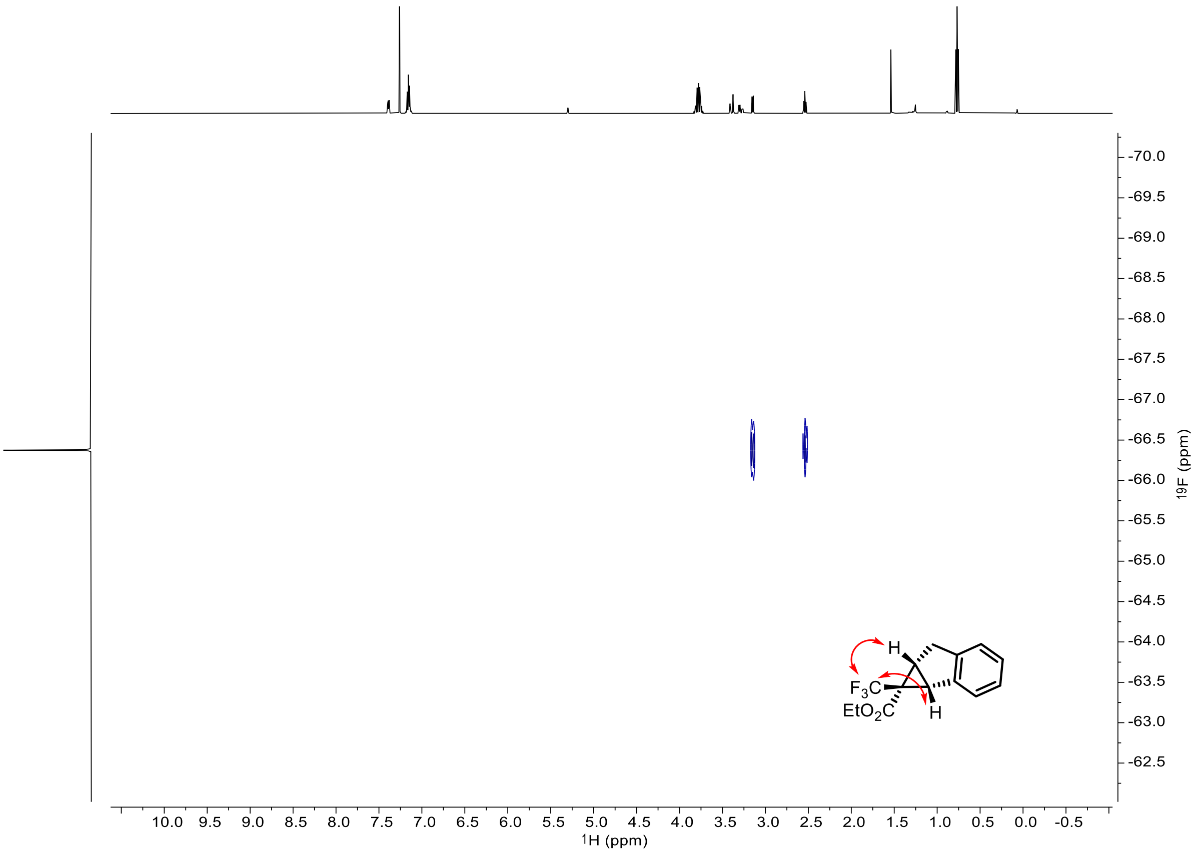

4i: ^{1}H NMR (600 MHz, [D$_6$]-DMSO, 383 K)

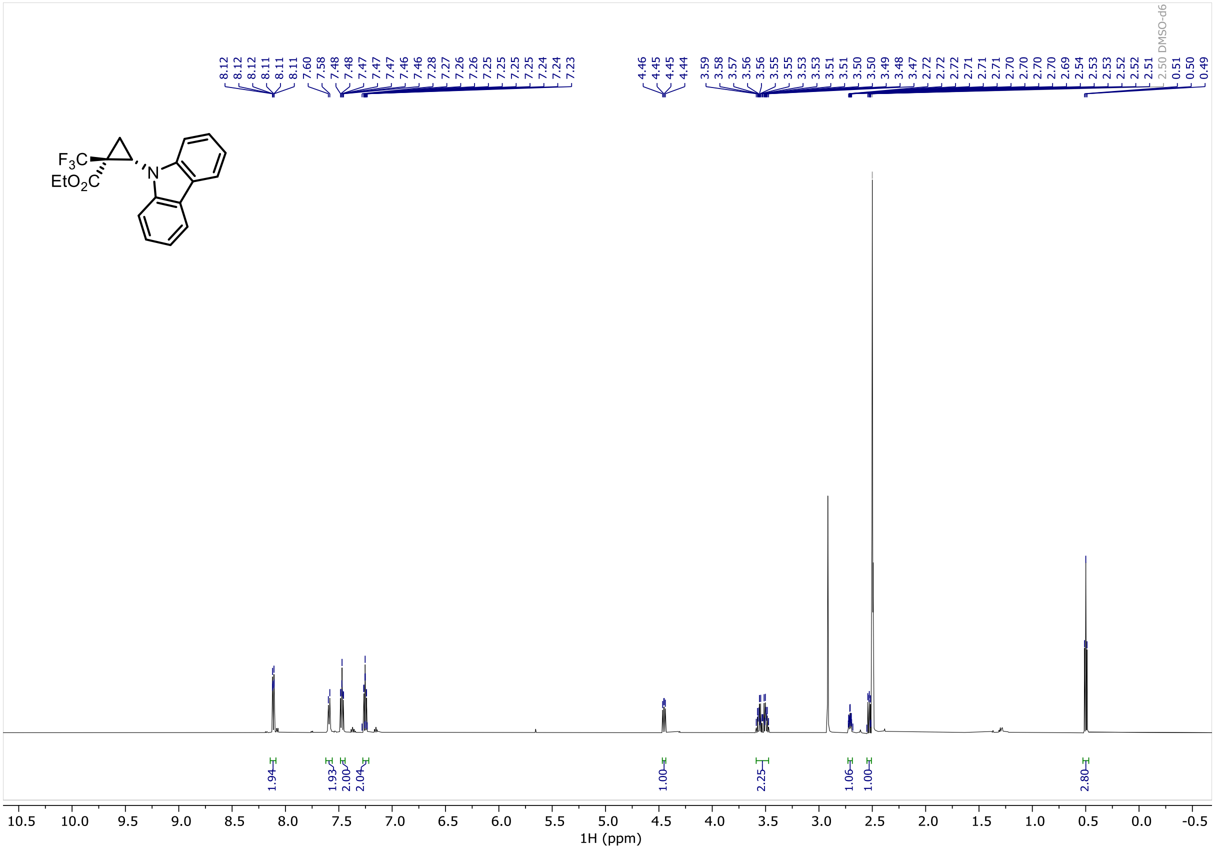

4i: ^{13}C NMR (151 MHz, [D$_6$]-DMSO, 383 K)

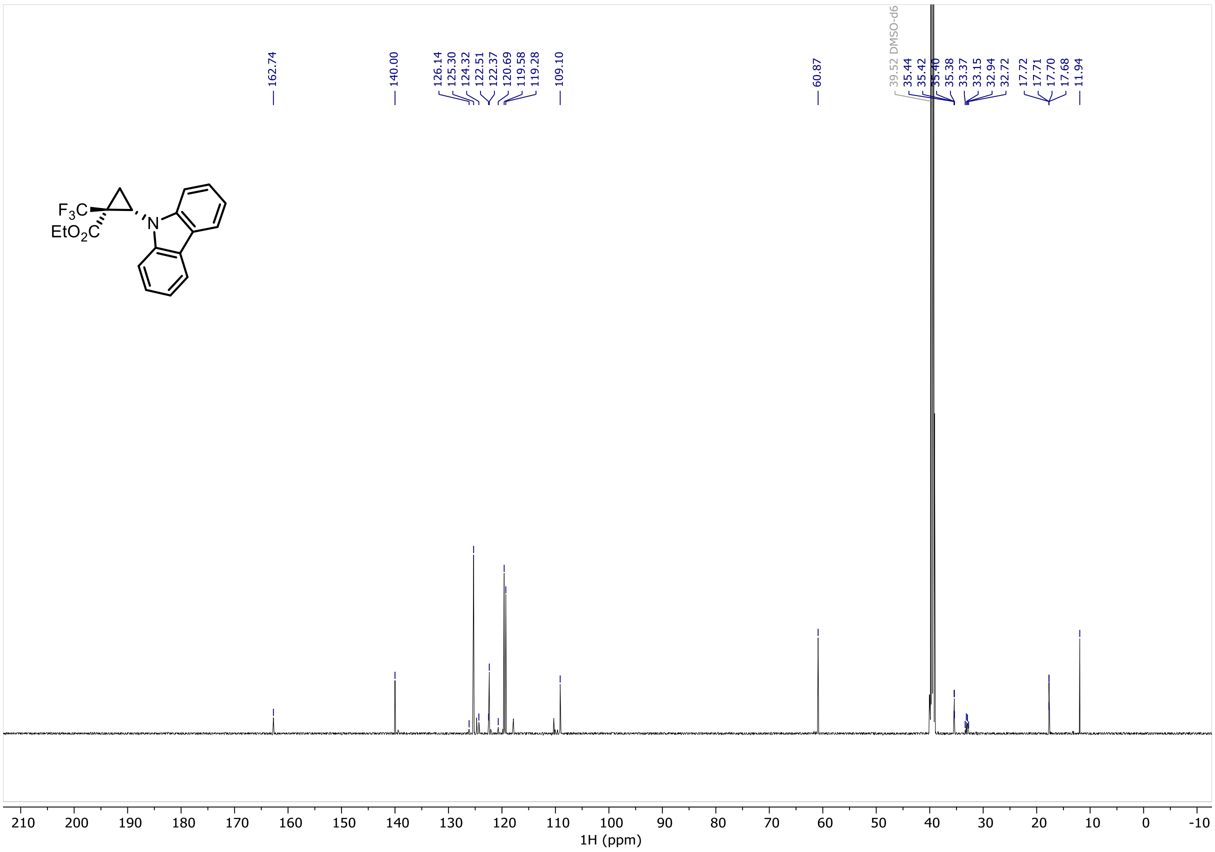

4i: ^{19}F NMR (565 MHz, [D$_6$]-DMSO, 383 K)

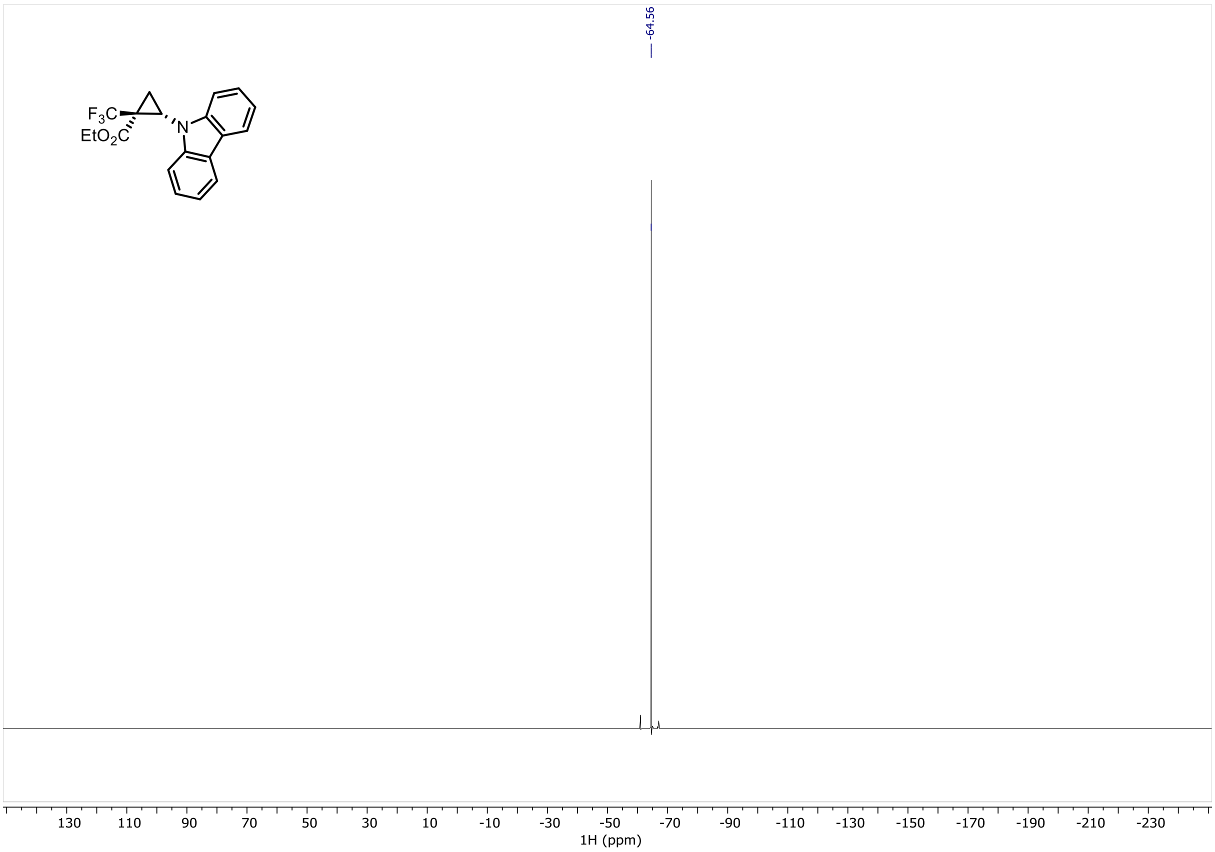

4j: ^{1}H NMR (400 MHz, $CDCl_3$)

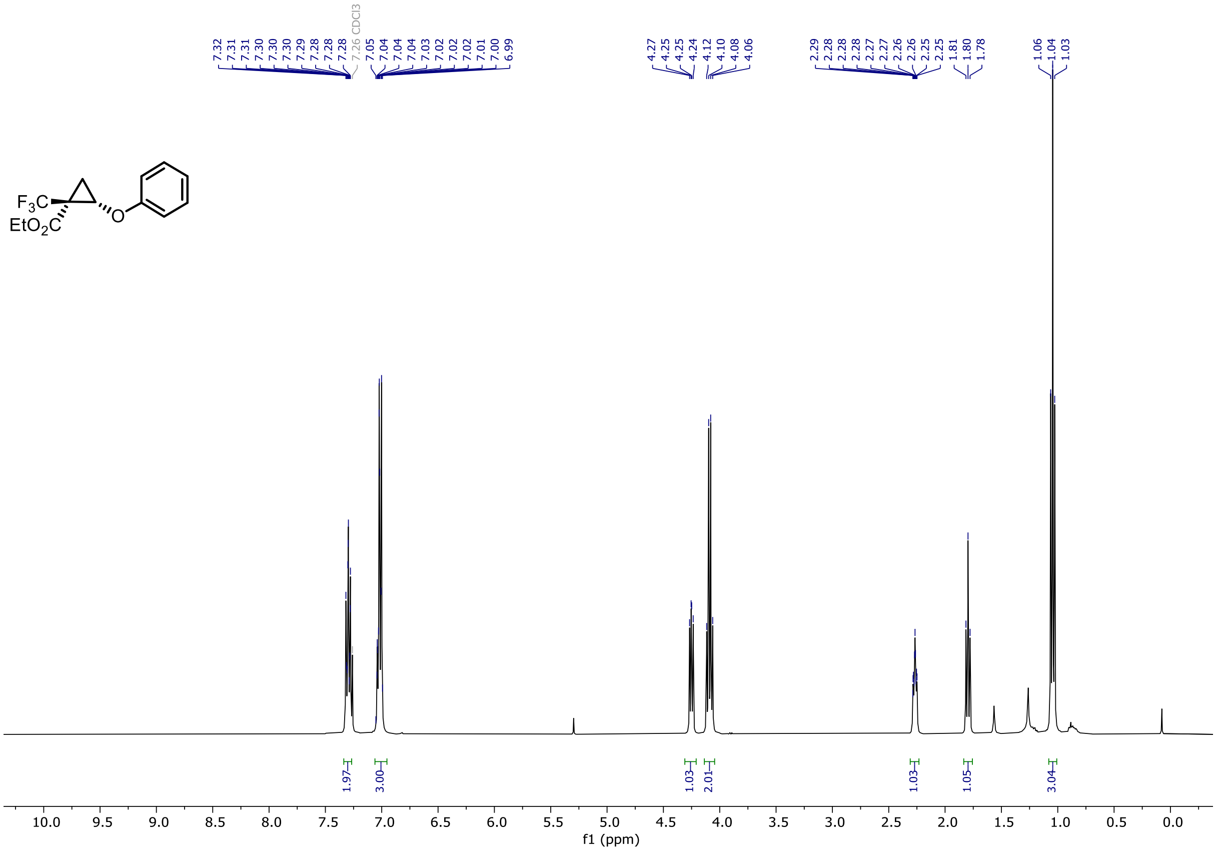

4j: ^{13}C NMR (101 MHz, $CDCl_3$)

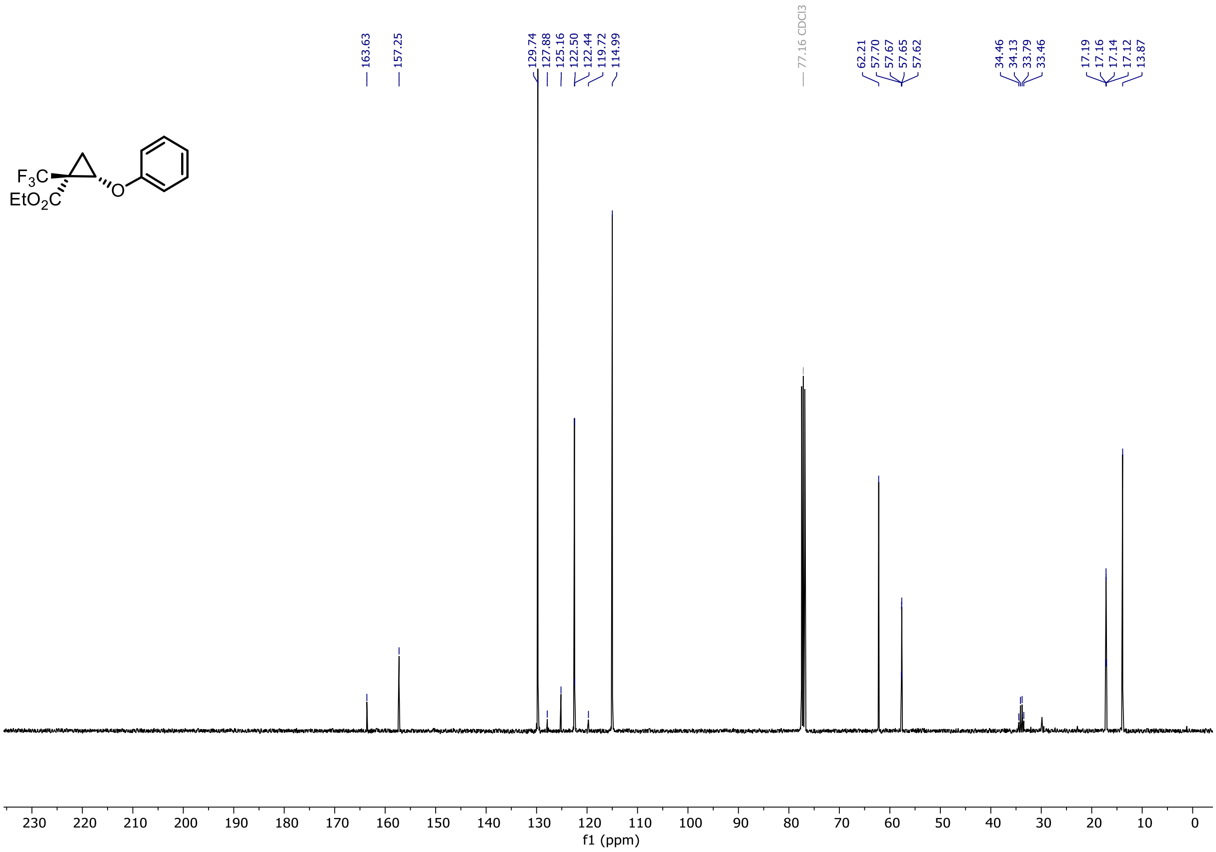

4j: ^{19}F NMR (282 MHz, $CDCl_3$)

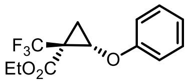

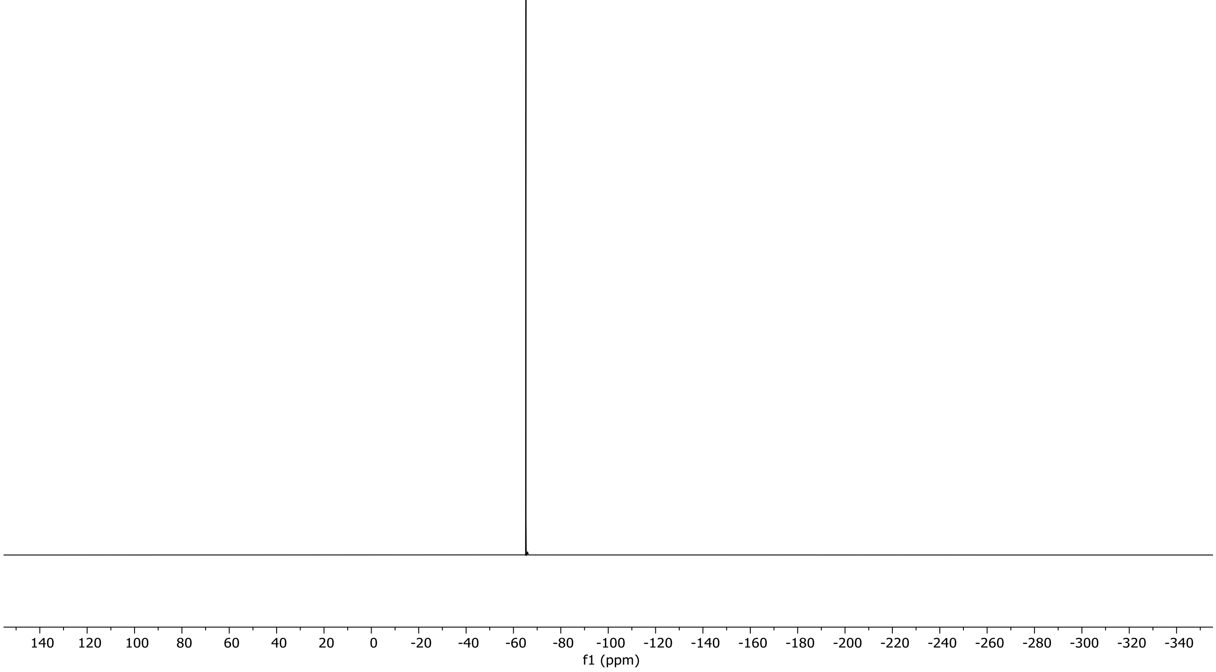

4k (mixture of diastereomers, dr ≈ 4.5:1): ^{1}H NMR (400 MHz, $CDCl_3$)

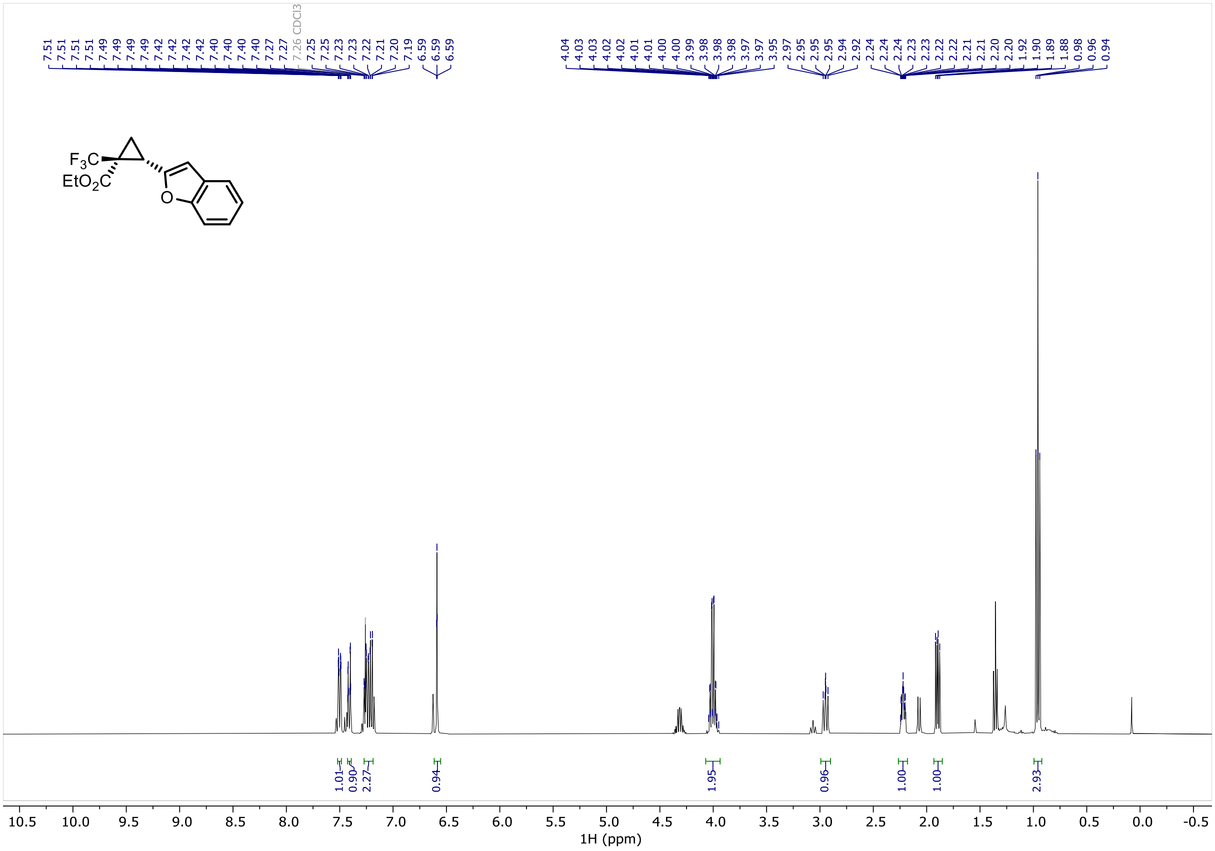

4k (mixture of diastereomers, dr ≈ 4.5:1): ^{13}C NMR (101 MHz, $CDCl_3$)

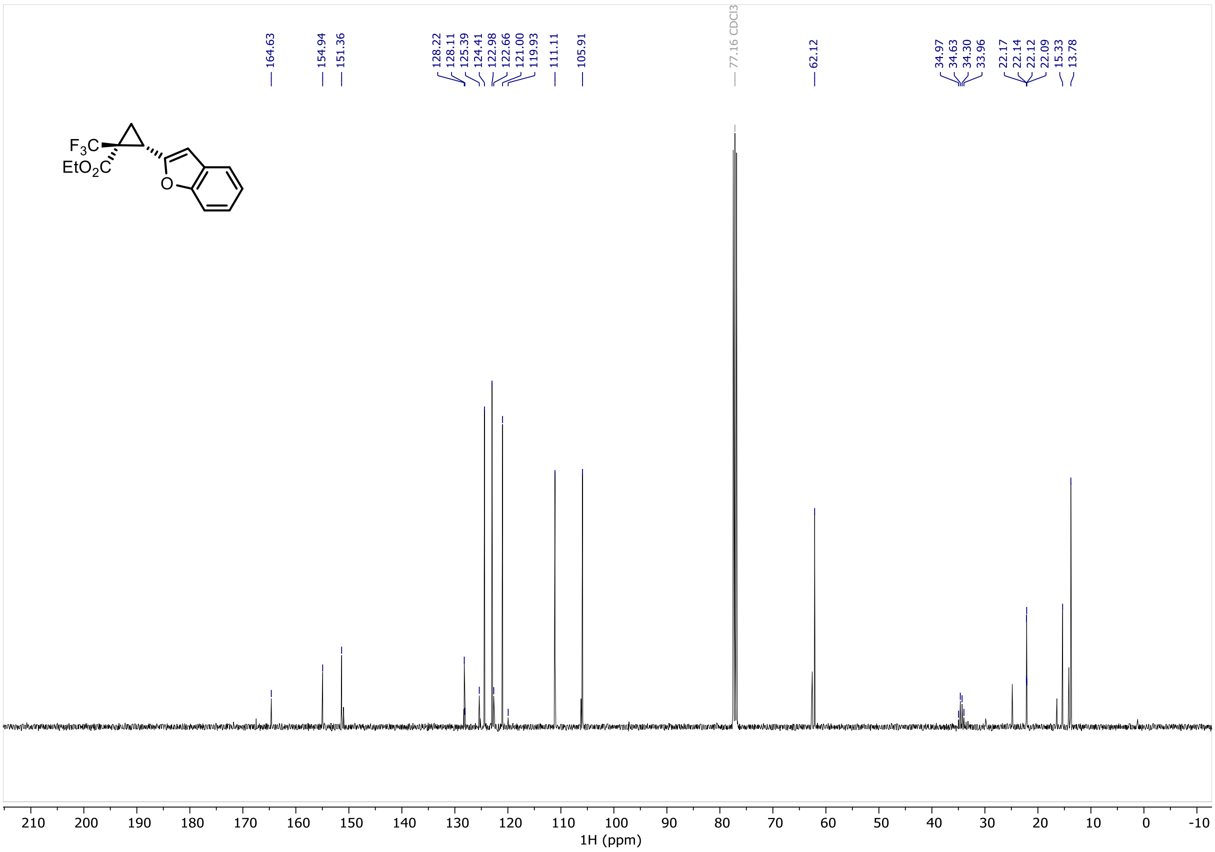

4k: (mixture of diastereomers, dr ≈ 4.5:1): ^{19}F NMR (376 MHz, $CDCl_3$)

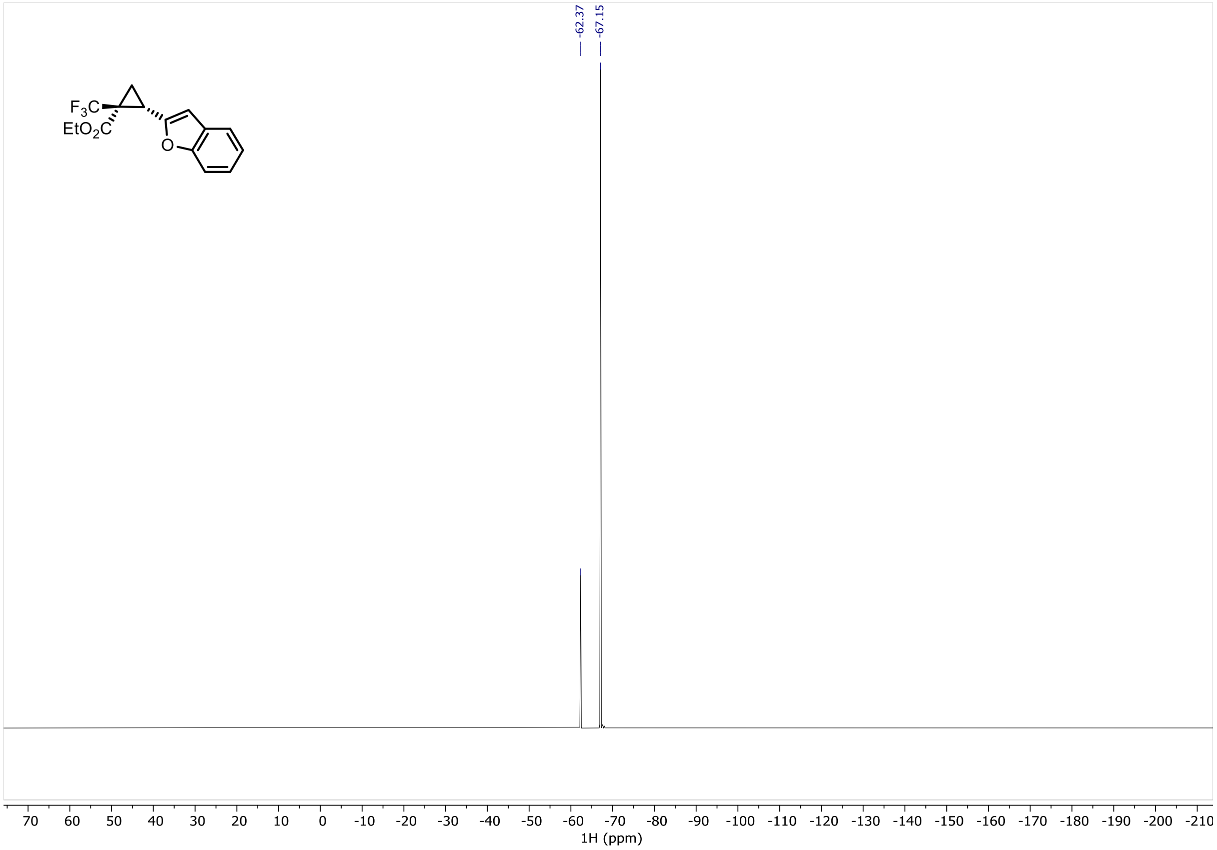

4l: ^1H NMR (400 MHz, $CDCl_3$)

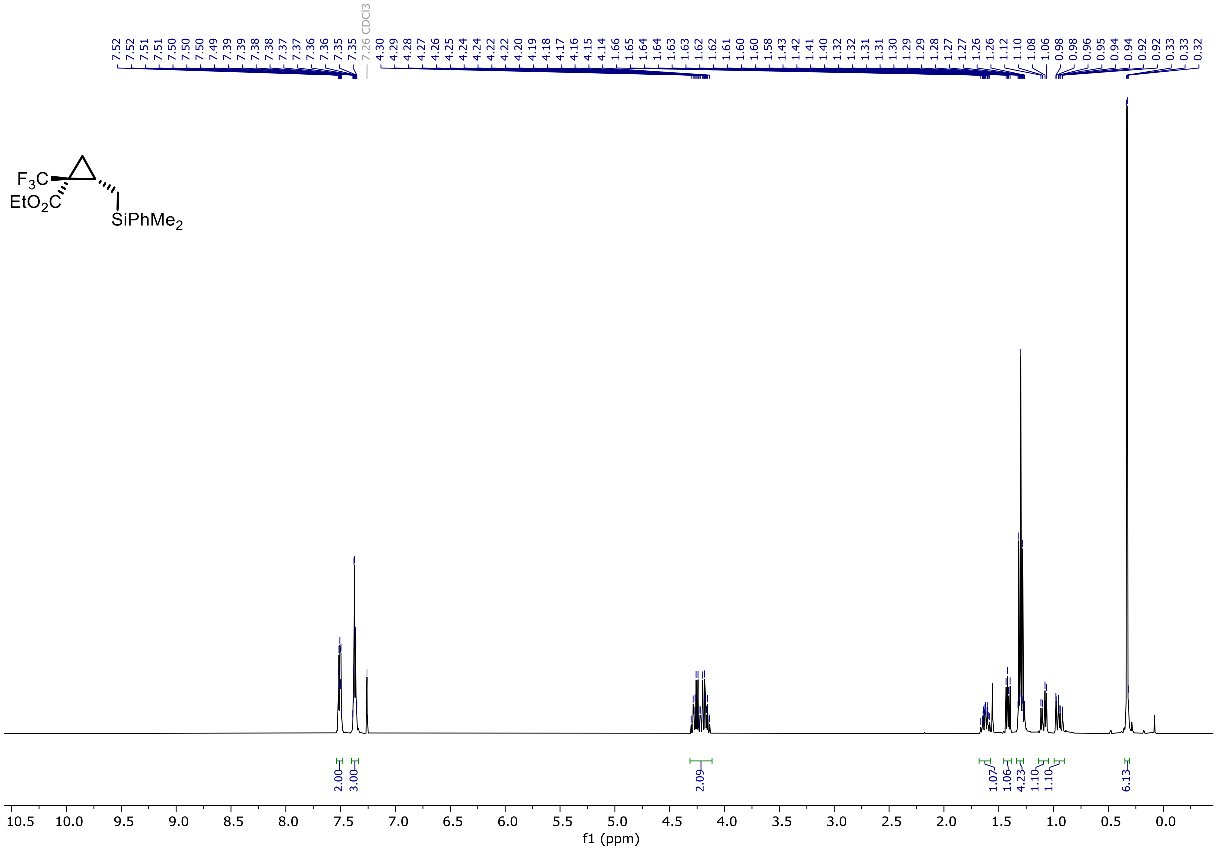

4l: ^{13}C NMR (101 MHz, $CDCl_3$)

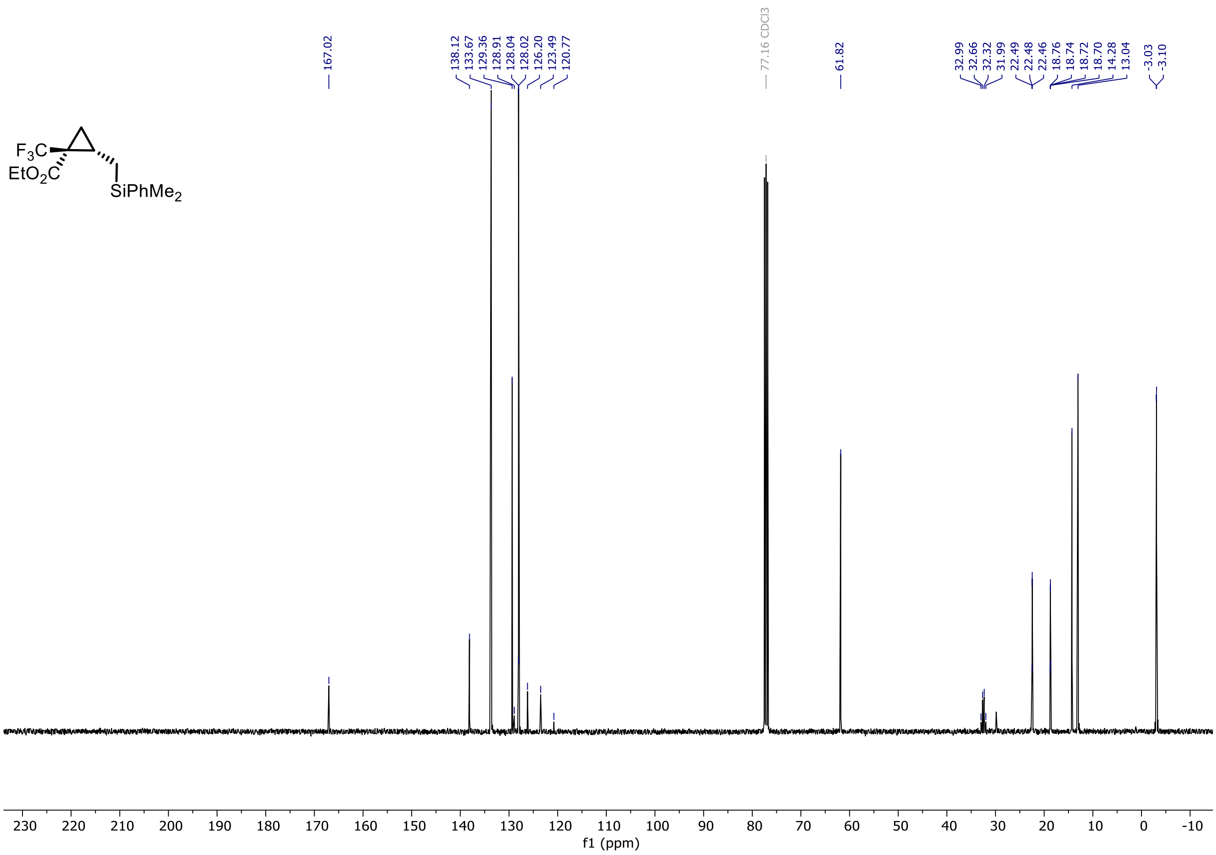

4l: ^{19}F NMR (282 MHz, $CDCl_3$)

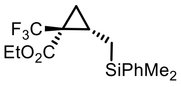

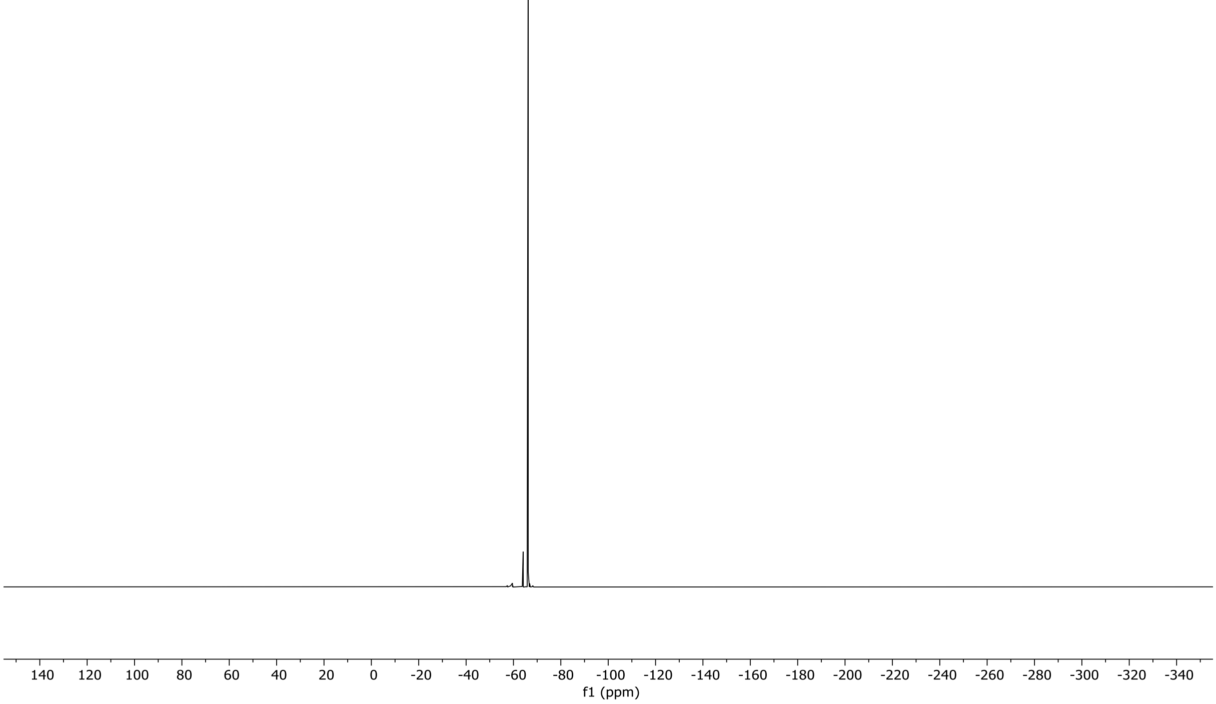

4m: ^{1}H NMR (400 MHz, $CDCl_3$)

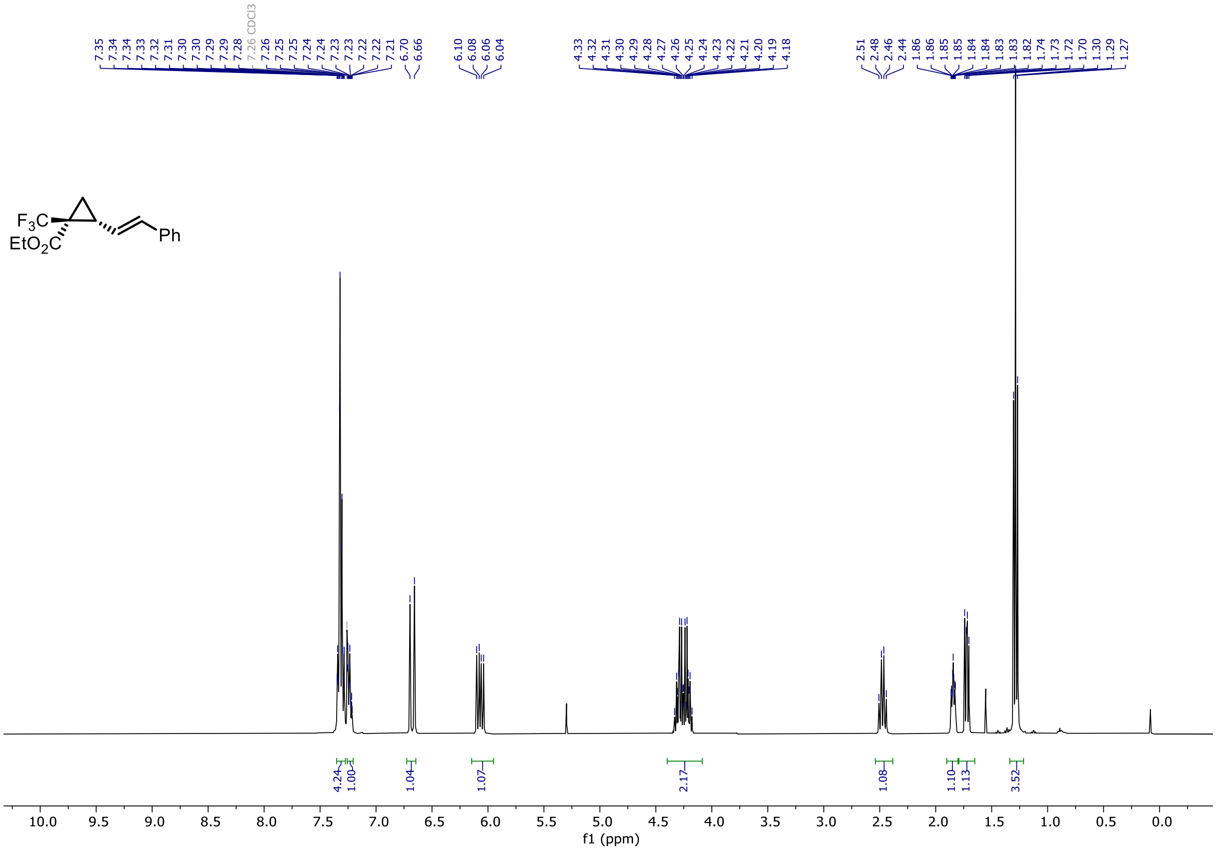

4m: ^{13}C NMR (101 MHz, $CDCl_3$)

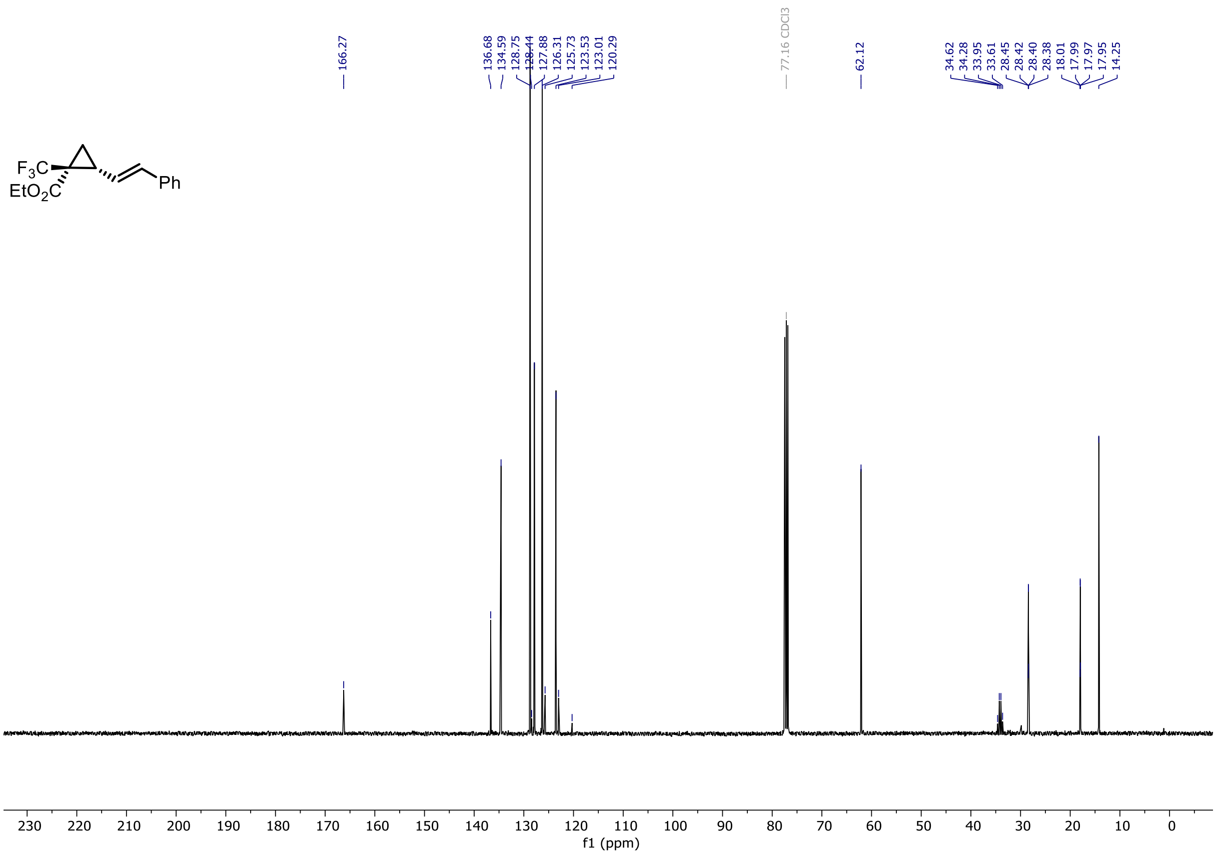

4m: ^{19}F NMR (282 MHz, $CDCl_3$)

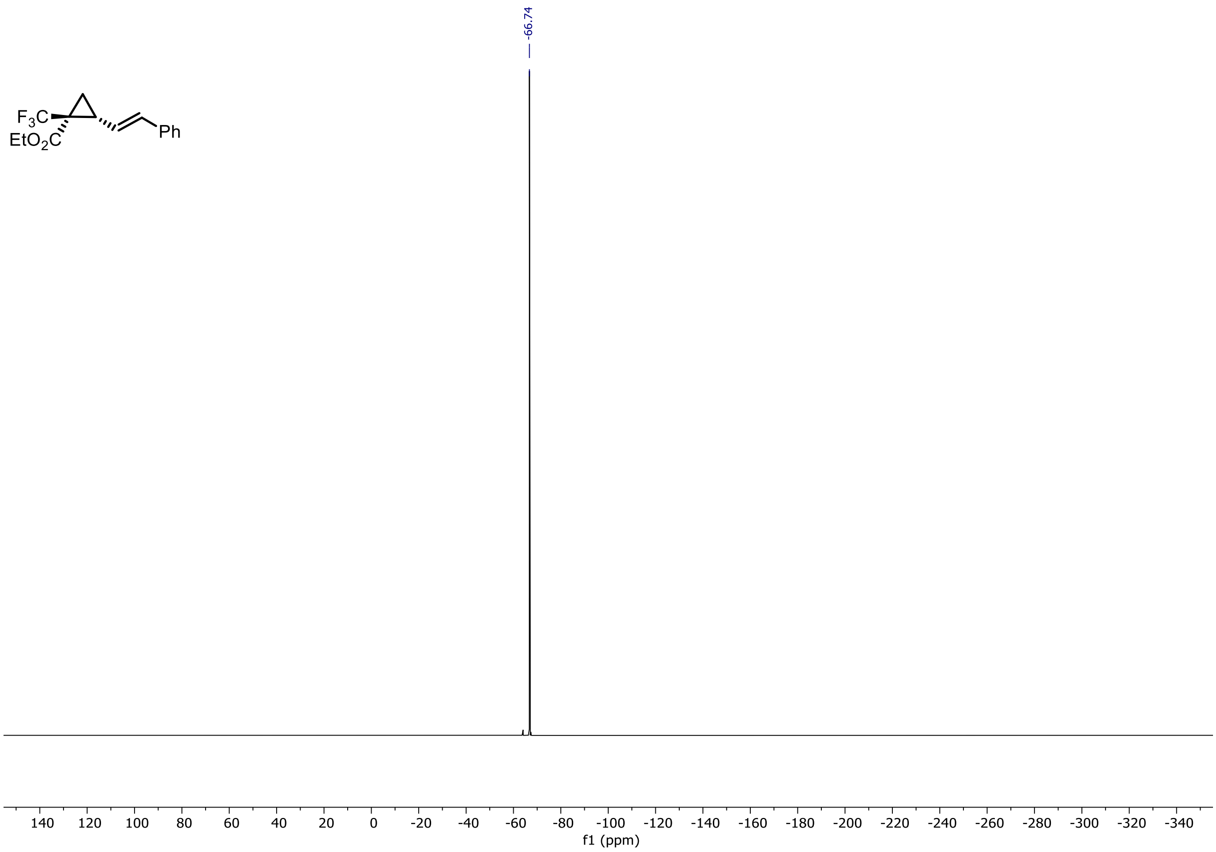

4n (mixture of diastereomers, dr ≈ 4:1): ^{1}H NMR (600 MHz, $CDCl_3$)

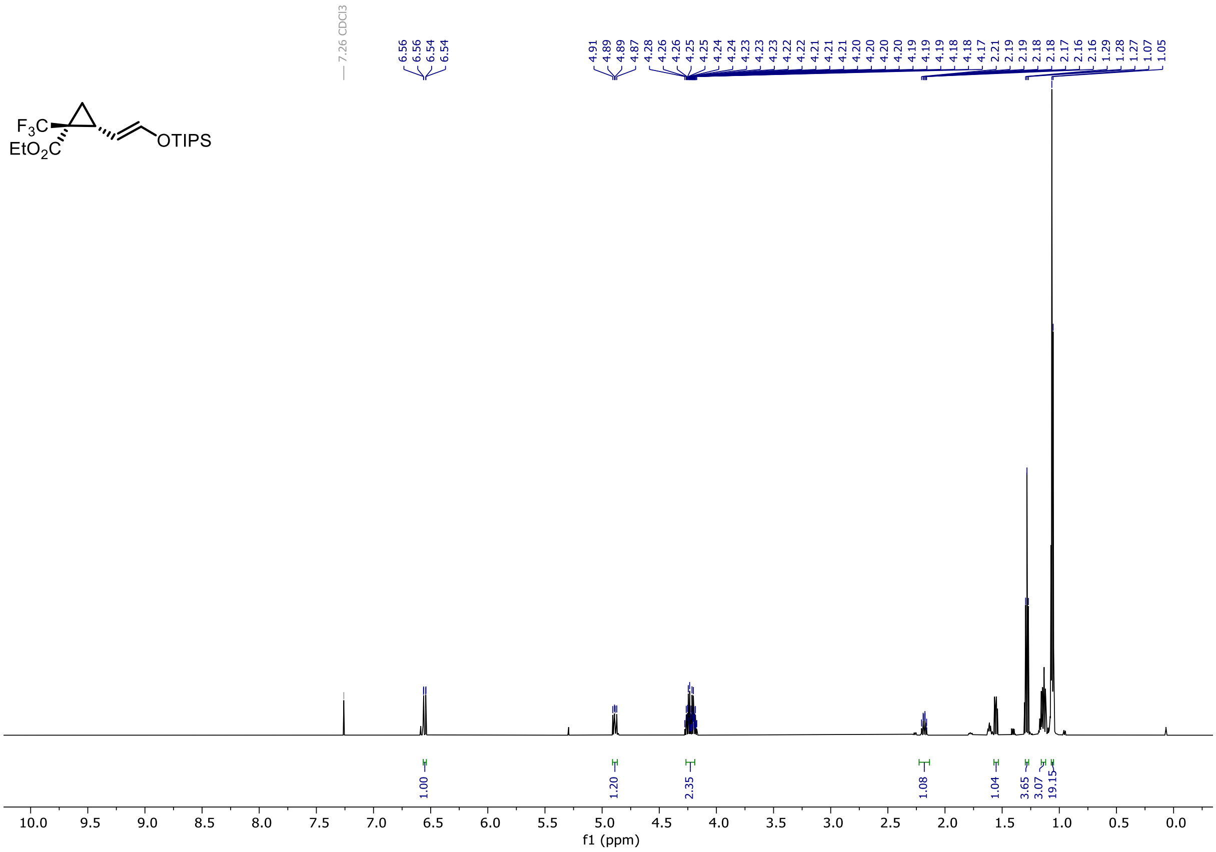

4n (mixture of diastereomers, dr ≈ 4:1): ^{13}C NMR (151 MHz, $CDCl_3$)

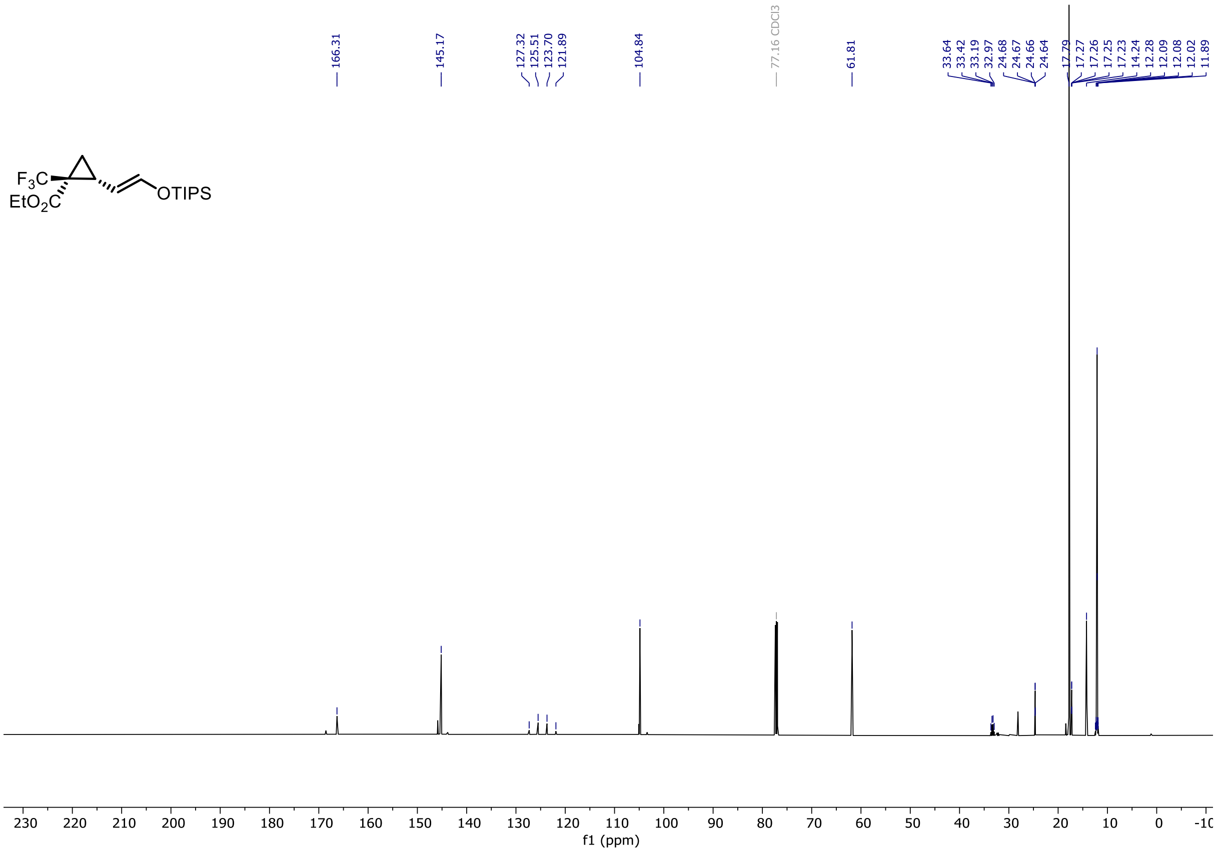

4n (mixture of diastereomers, dr ≈ 4:1): ^{19}F NMR (565 MHz, $CDCl_3$)

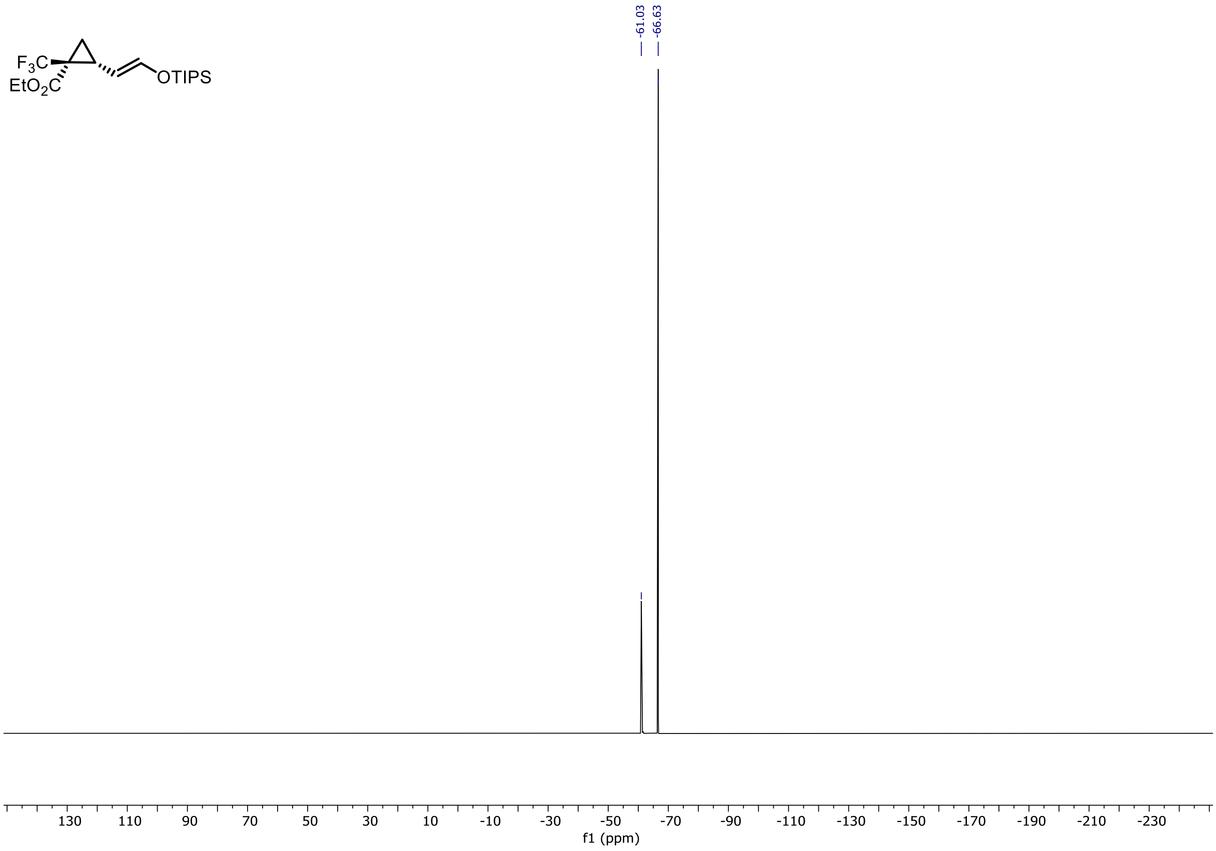

4n (mixture of diastereomers, dr ≈ 4:1): ^{1}H-^{1}H NOESY ($CDCl_3$)

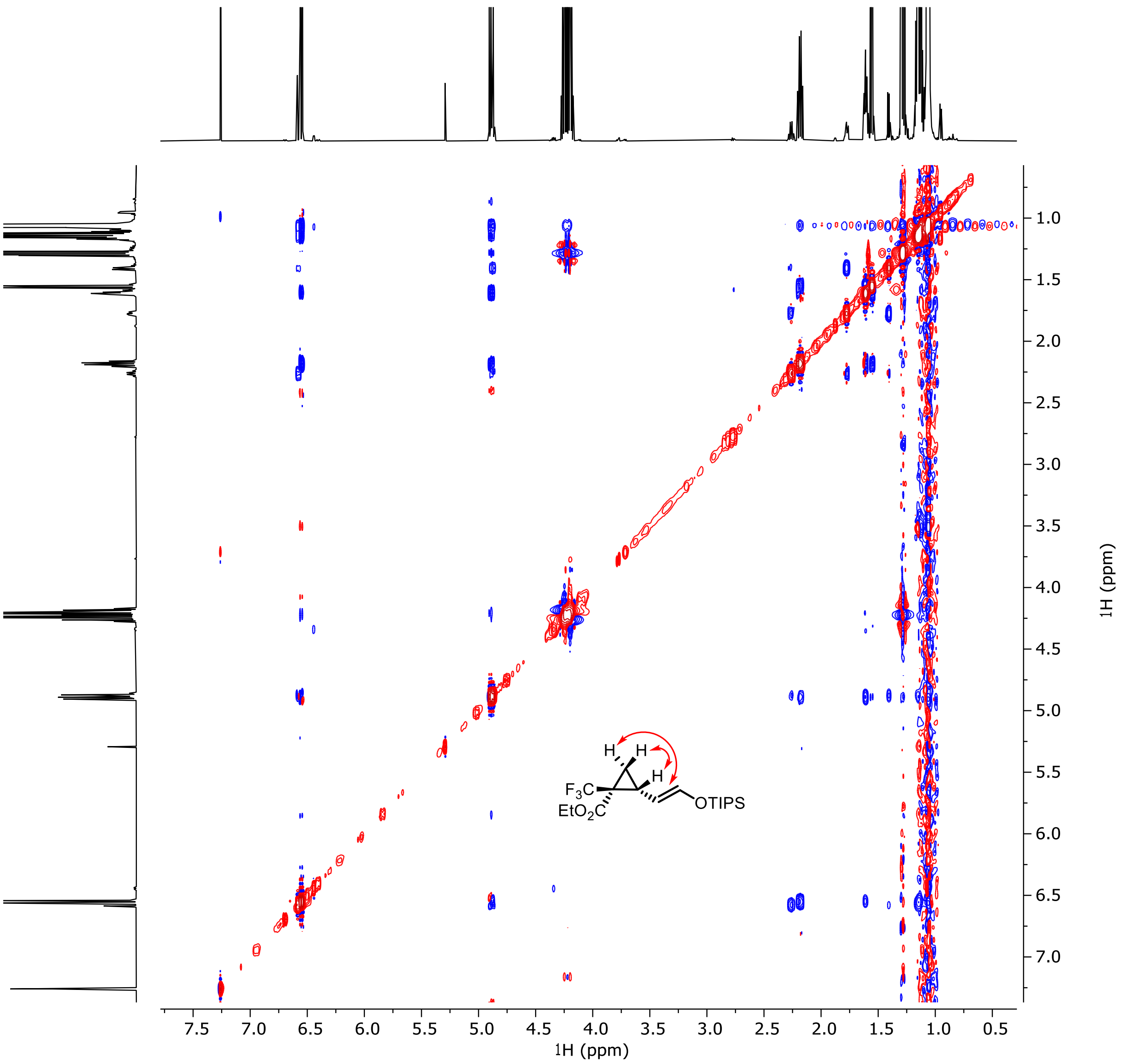

4o (mixture of diastereomers, dr ≈ 6:1): ^{1}H NMR (400 MHz, $CDCl_3$)

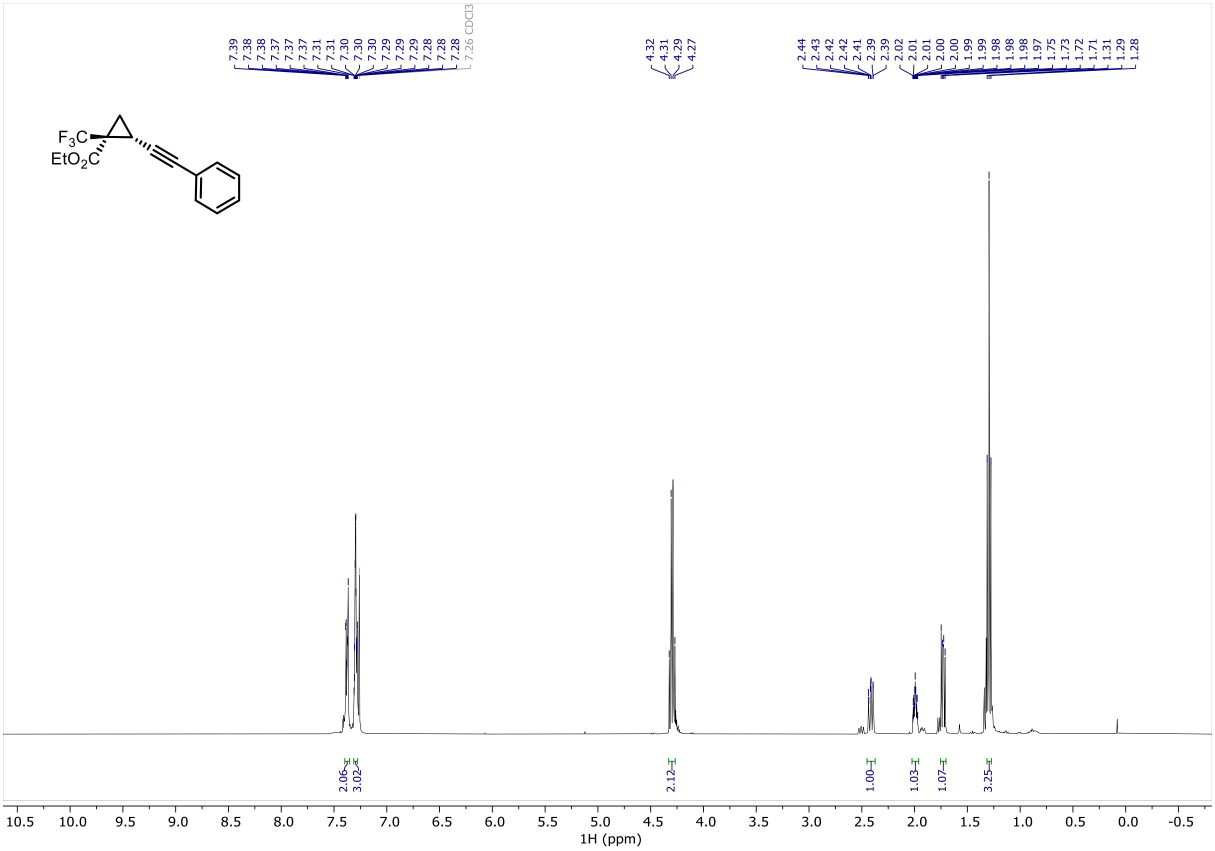

4o (mixture of diastereomers, dr ≈ 6:1): ^{13}C NMR (101 MHz, $CDCl_3$)

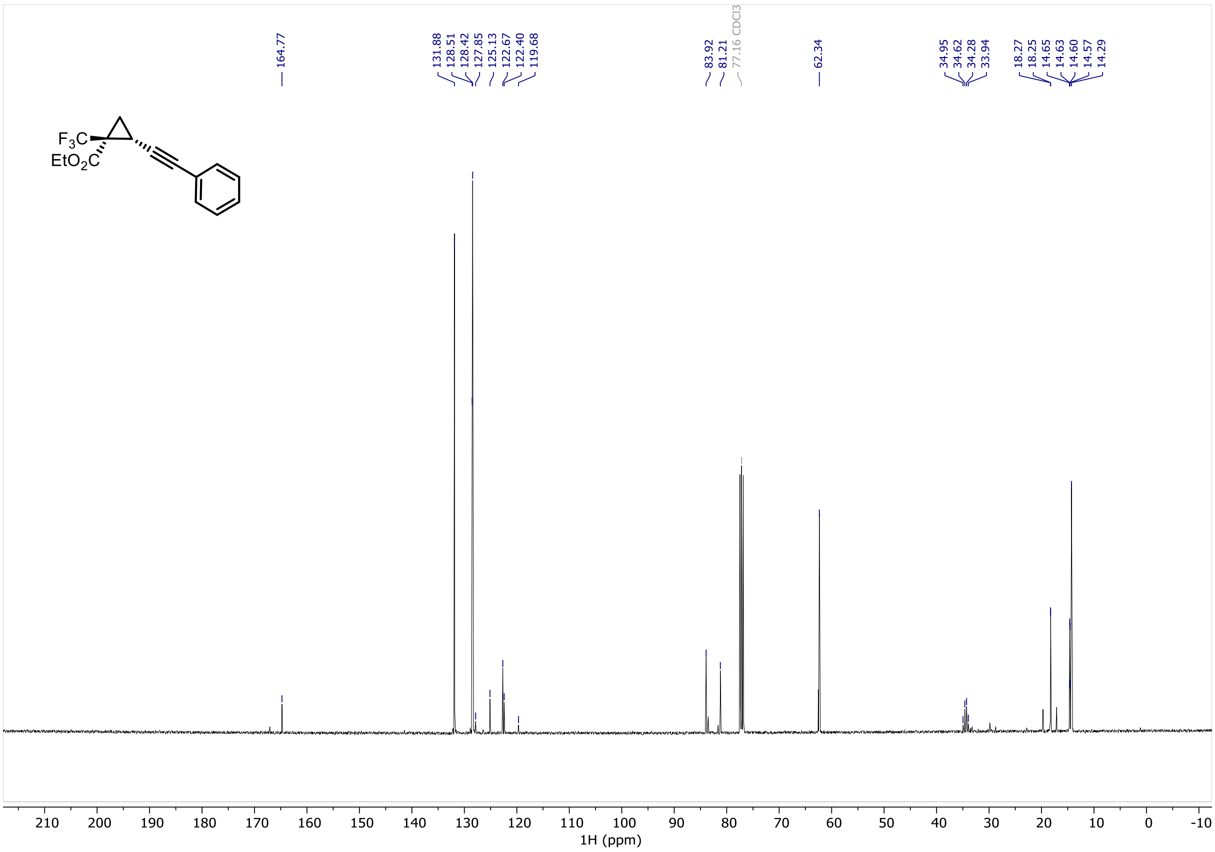

4o (mixture of diastereomers, dr ≈ 6:1): ^{19}F NMR (376 MHz, $CDCl_3$)

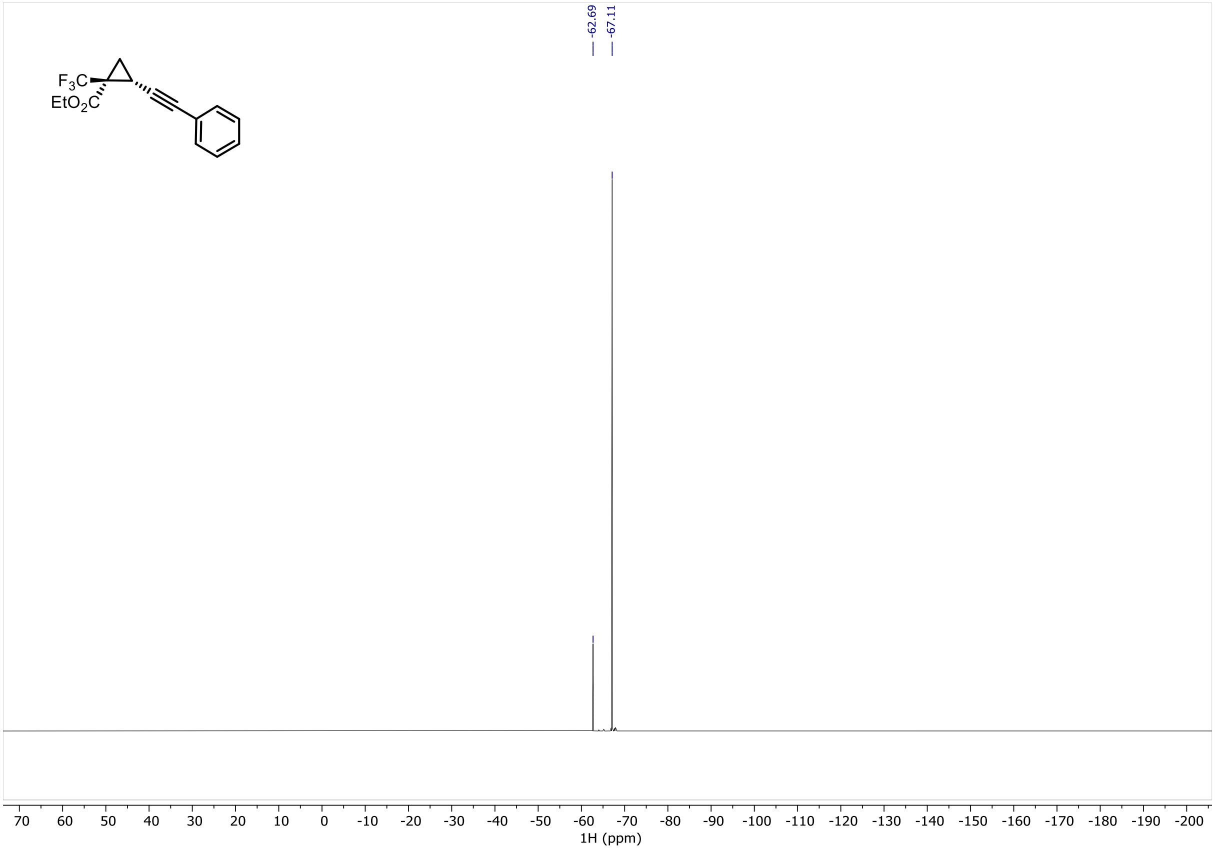

4p: ^1H NMR (400 MHz, $CDCl_3$)

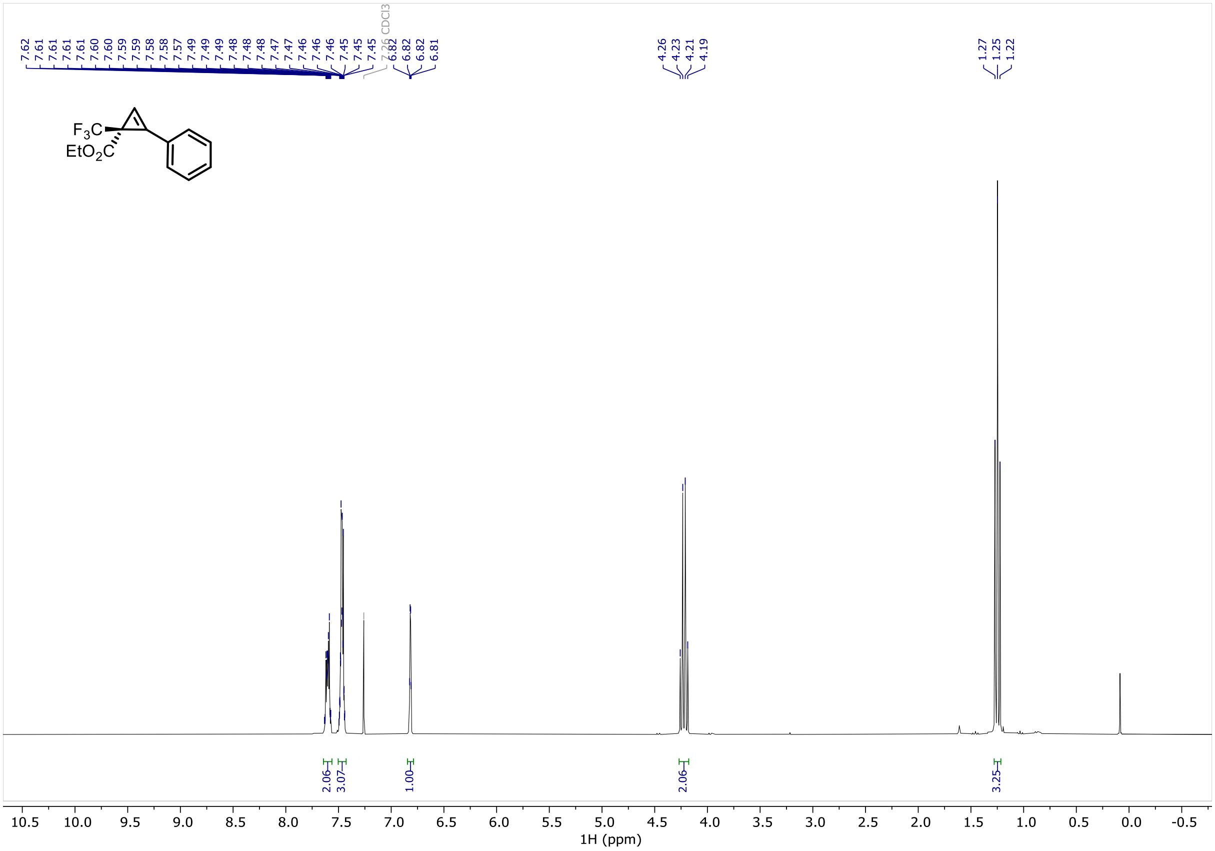

4p: ^{13}C NMR (101 MHz, $CDCl_3$)

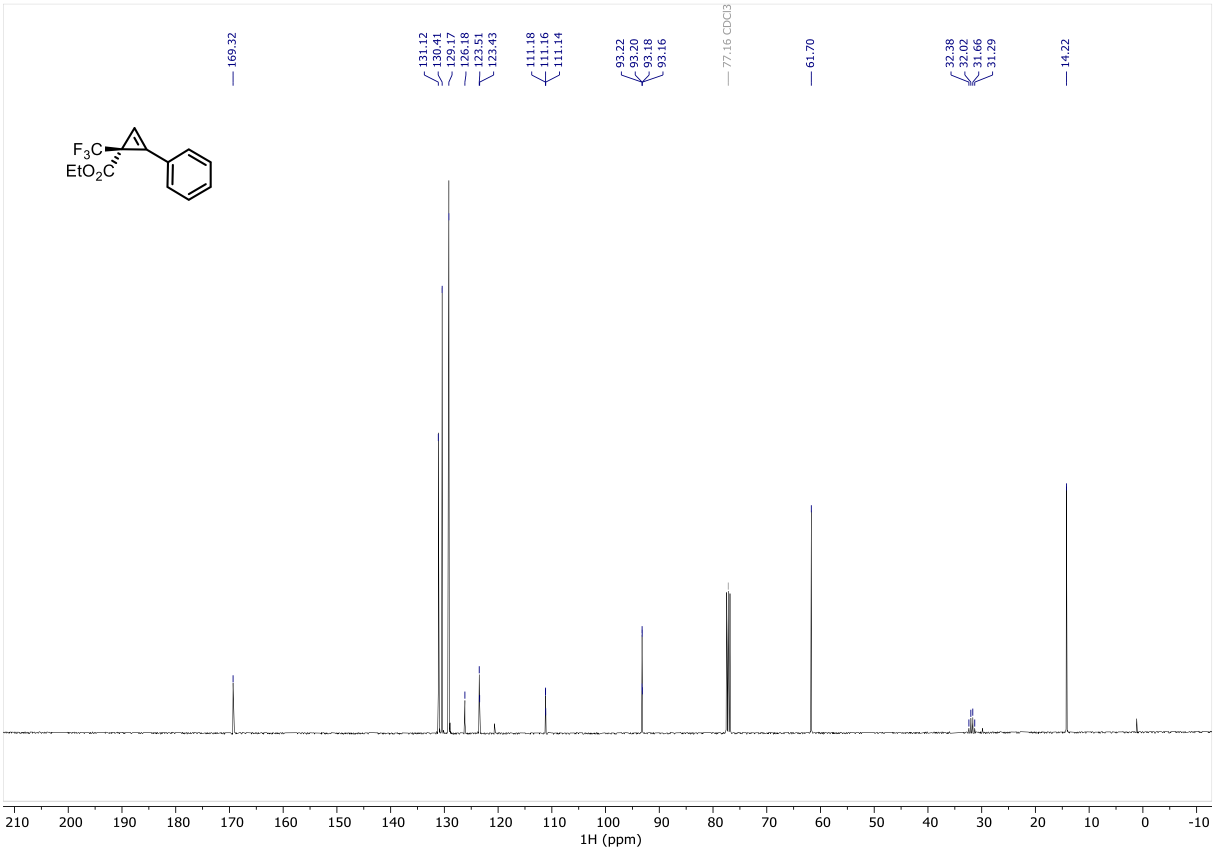

4p: ^{19}F NMR (282 MHz, $CDCl_3$)

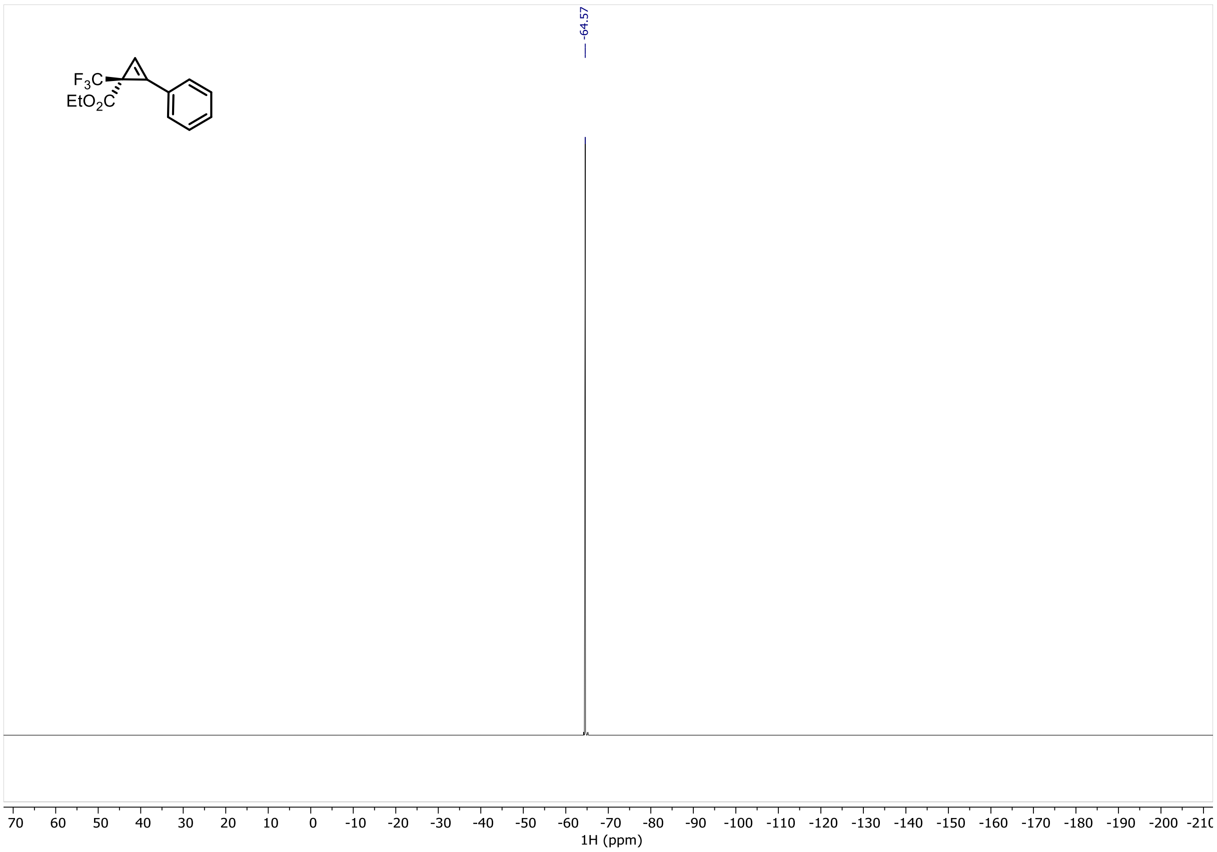

5a: ^{1}H NMR (400 MHz, $CDCl_3$)

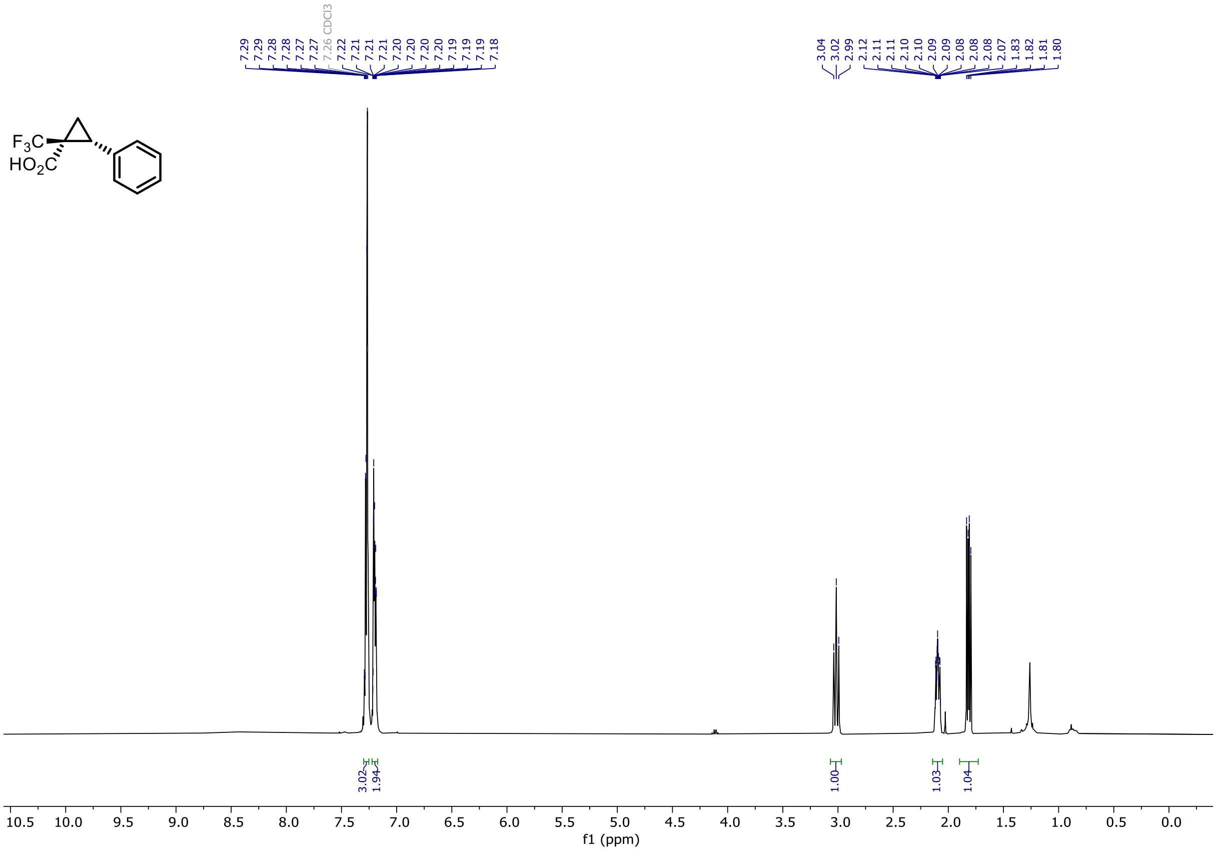

5a: ^{13}C NMR (101 MHz, $CDCl_3$)

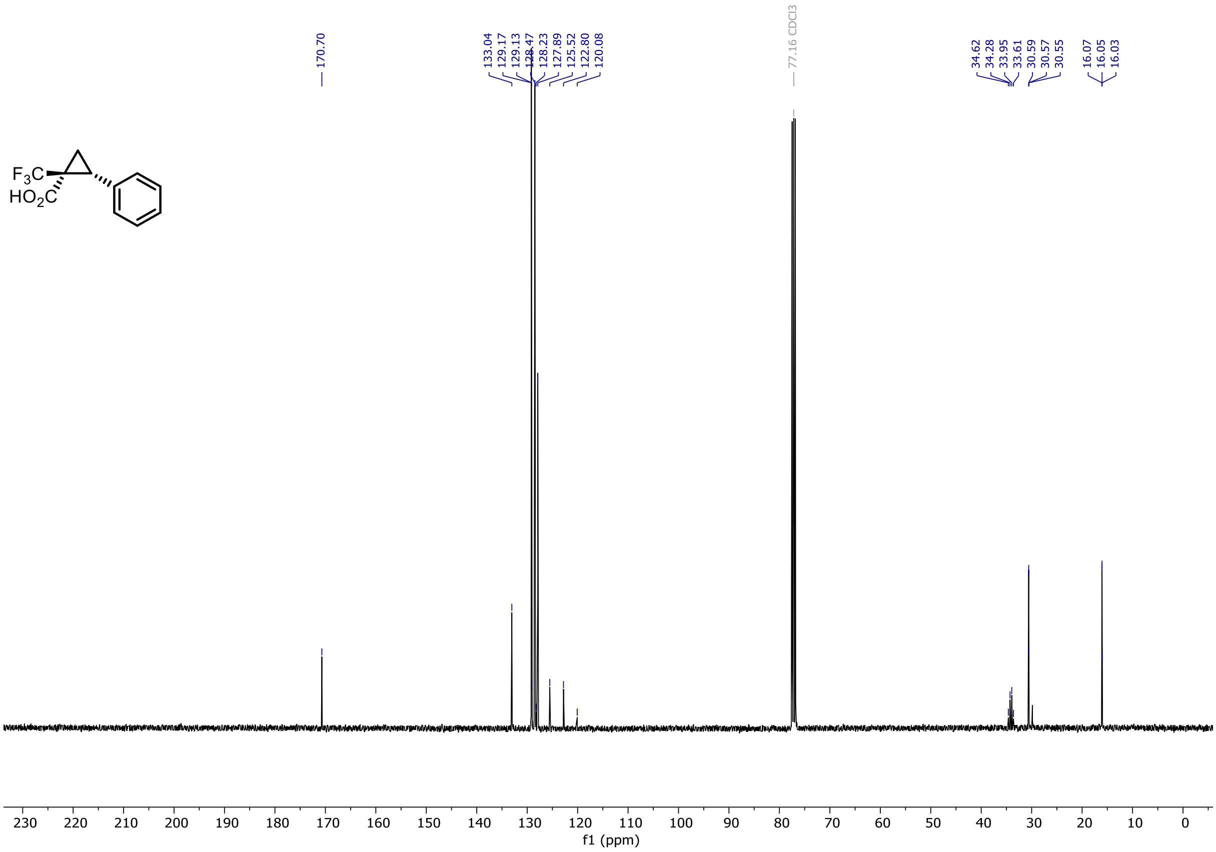

9a: ^{1}H NMR (500 MHz, $CDCl_3$)

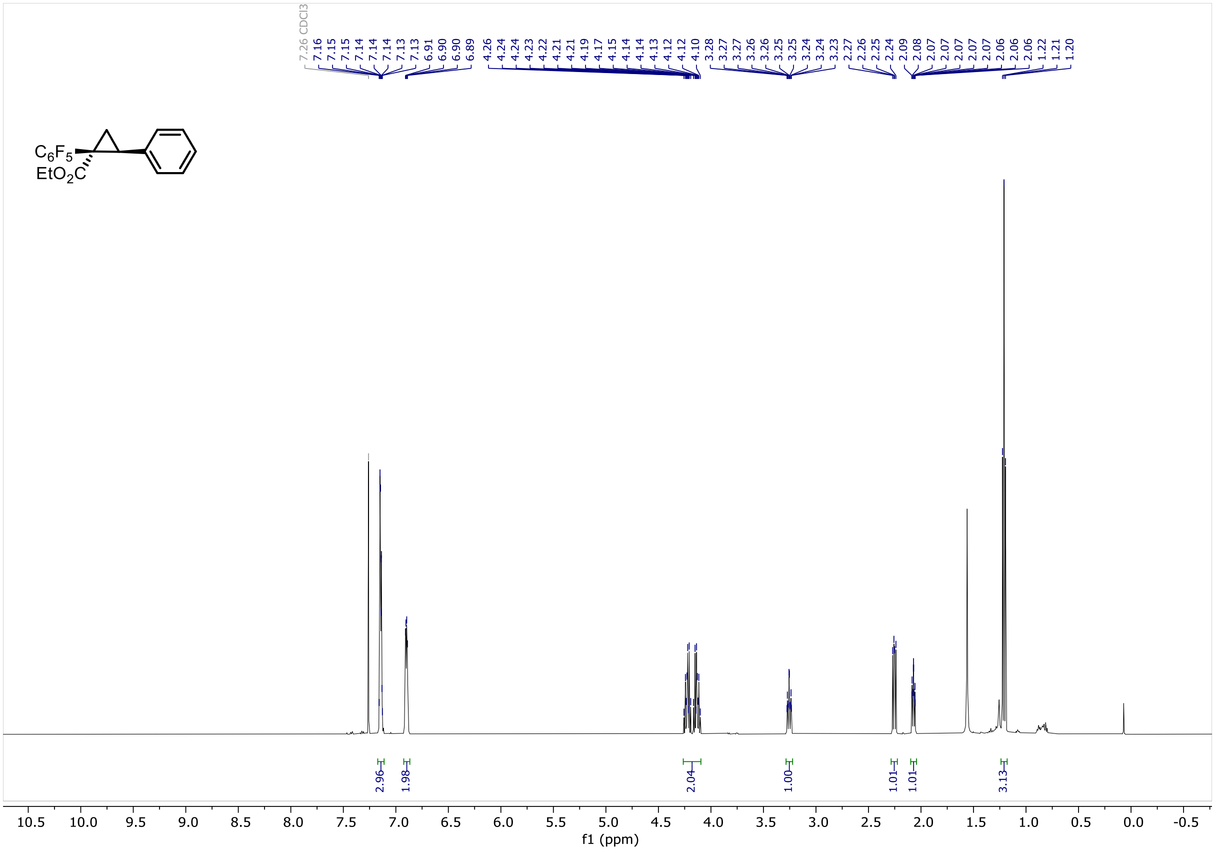

9a: ^{13}C NMR (126 MHz, $CDCl_3$)

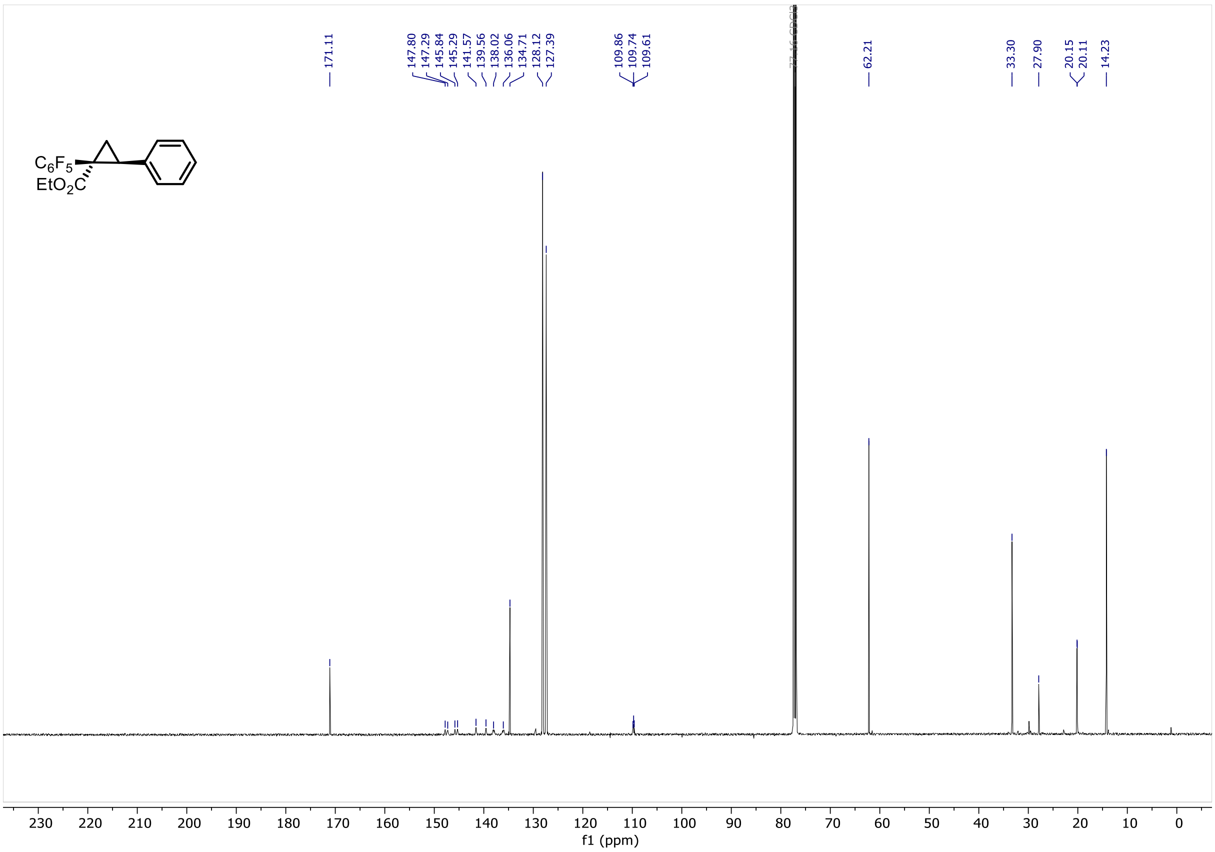

9a: ^{19}F NMR (470 MHz, $CDCl_3$)

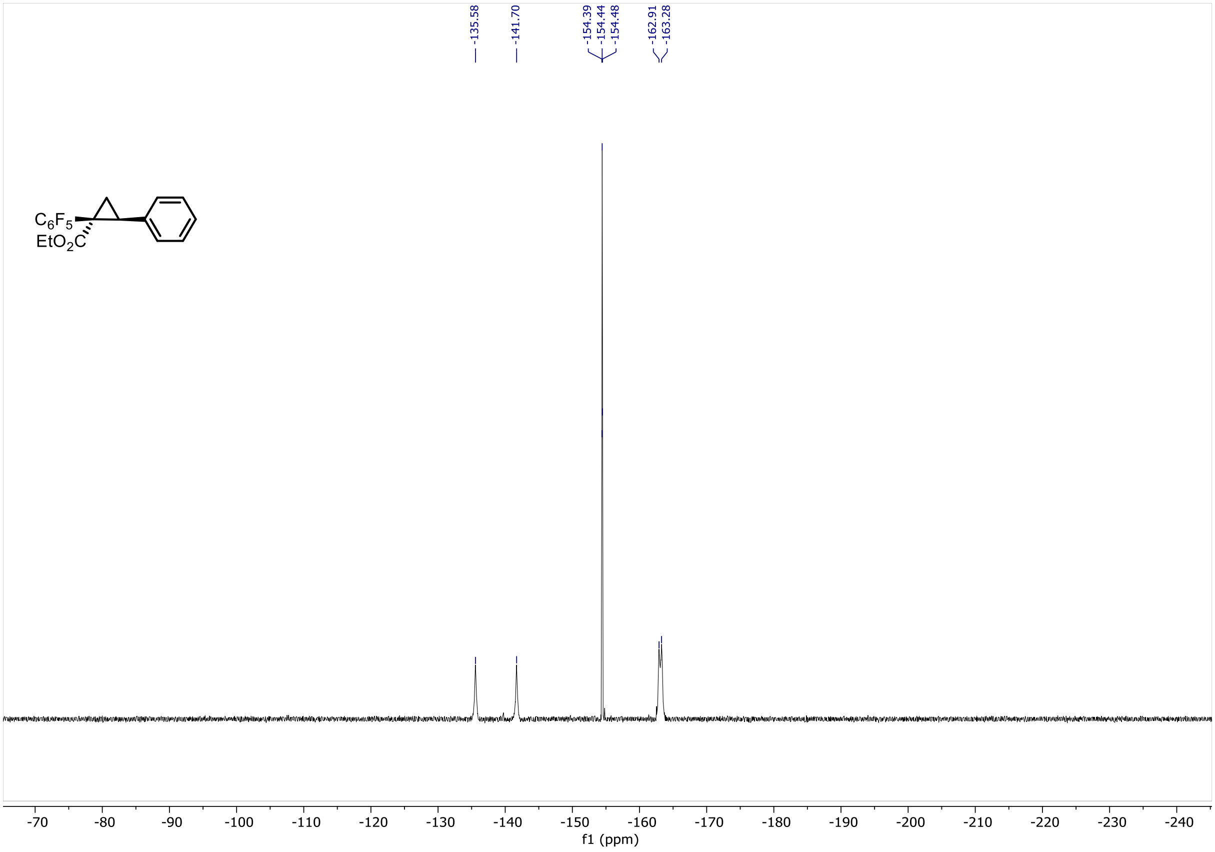

9a: ^{1}H-^{19}F HOESY ($CDCl_3$):

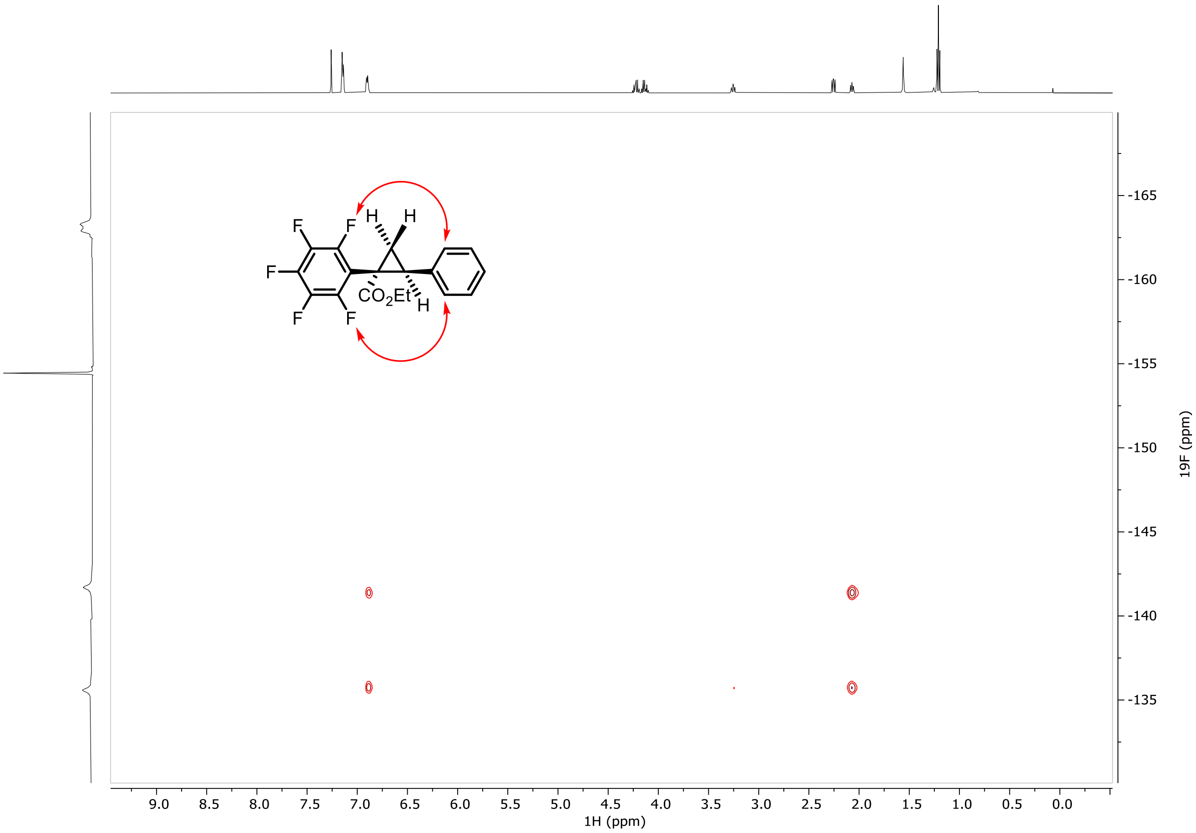

9a: ^{1}H-^{1}H NOESY ($CDCl_3$):

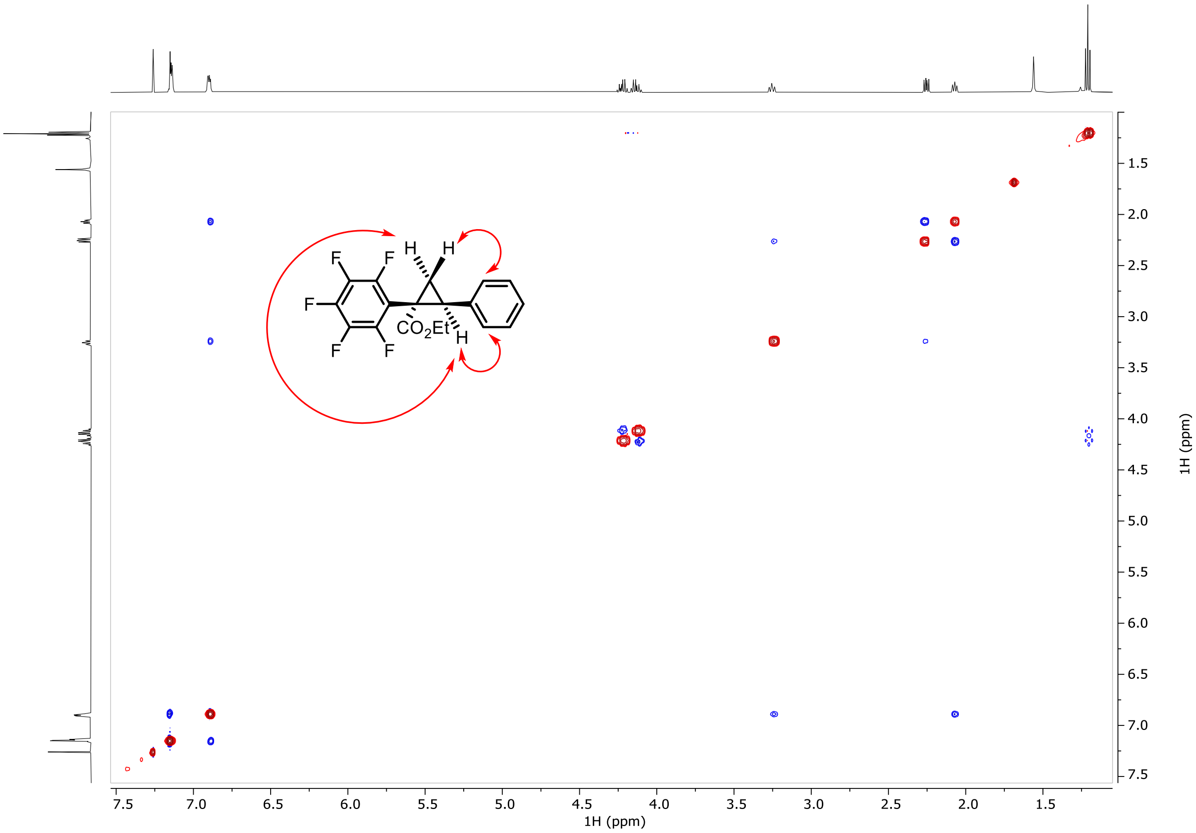

9b: ^{1}H NMR (400 MHz, $CDCl_3$)

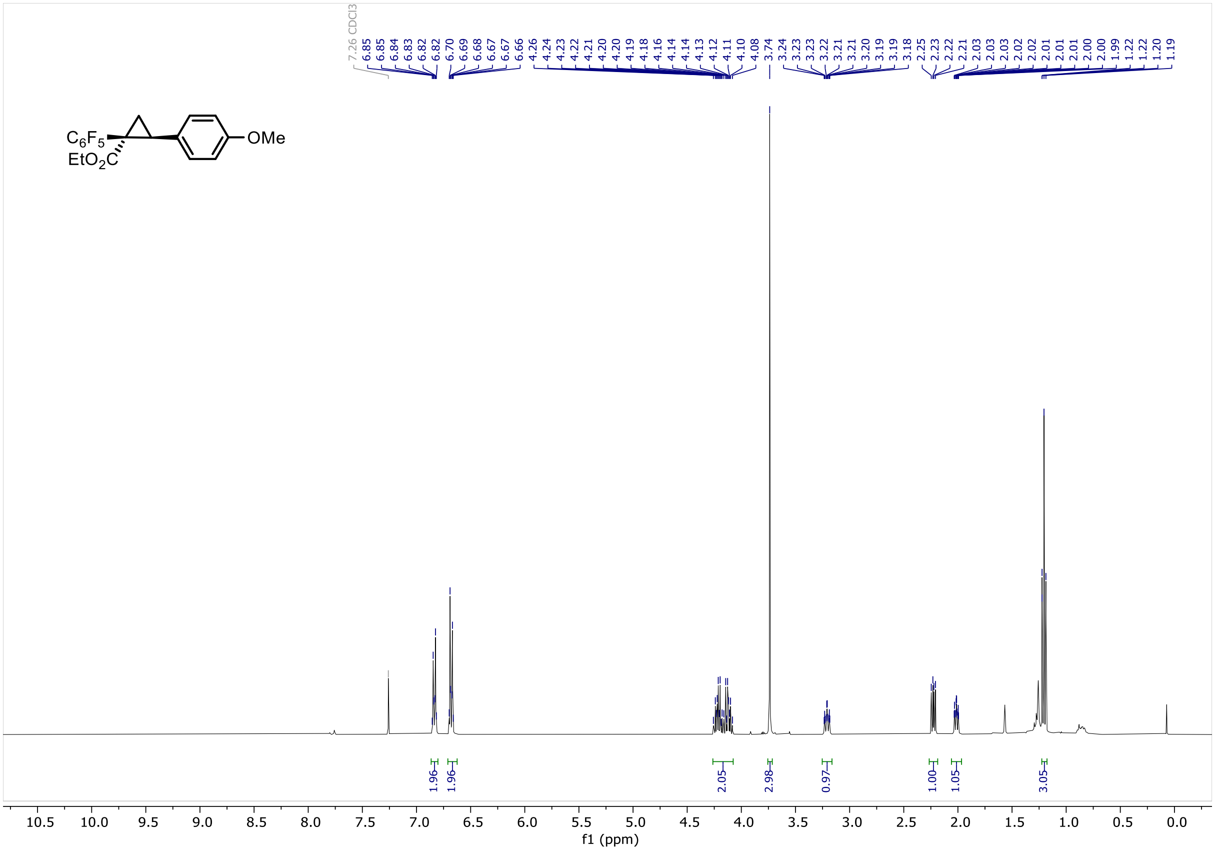

9b: ^{13}C NMR (101 MHz, $CDCl_3$)

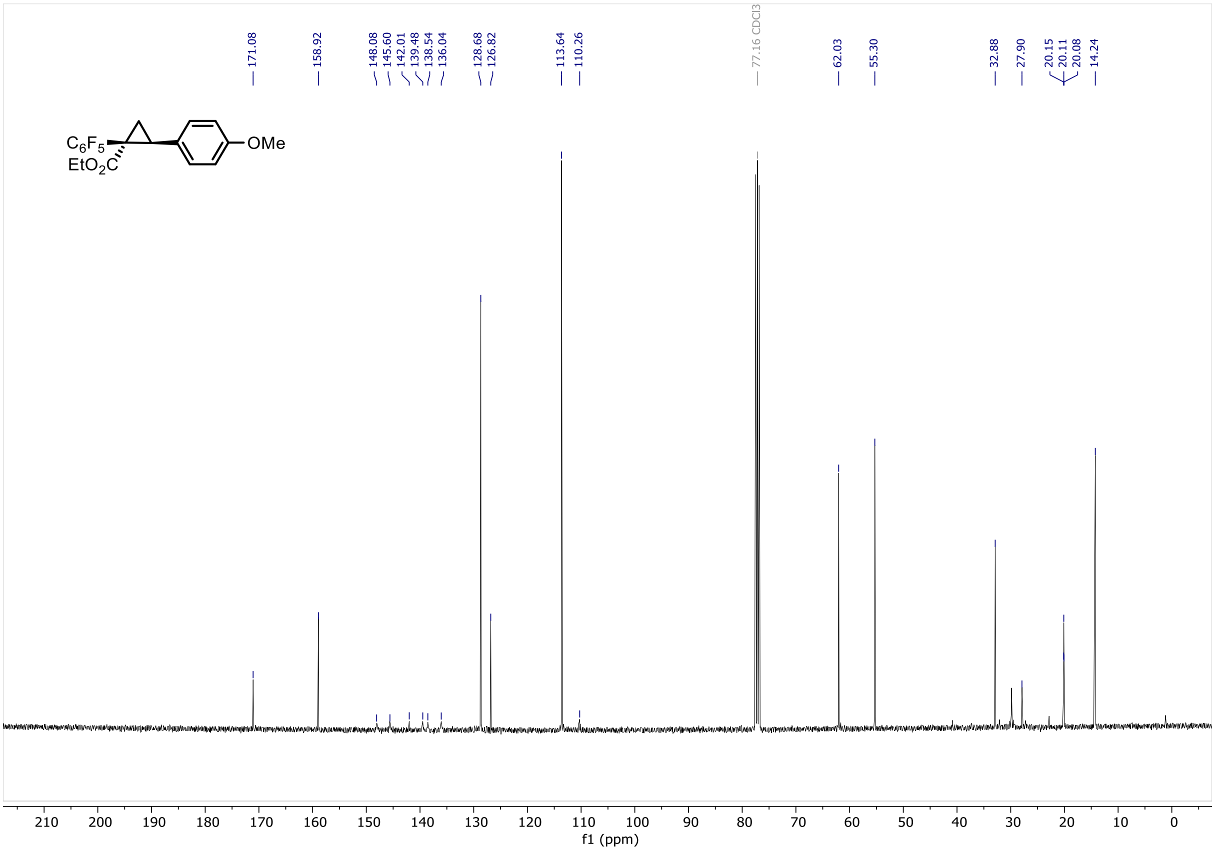

9b: ^{19}F NMR (282 MHz, $CDCl_3$)

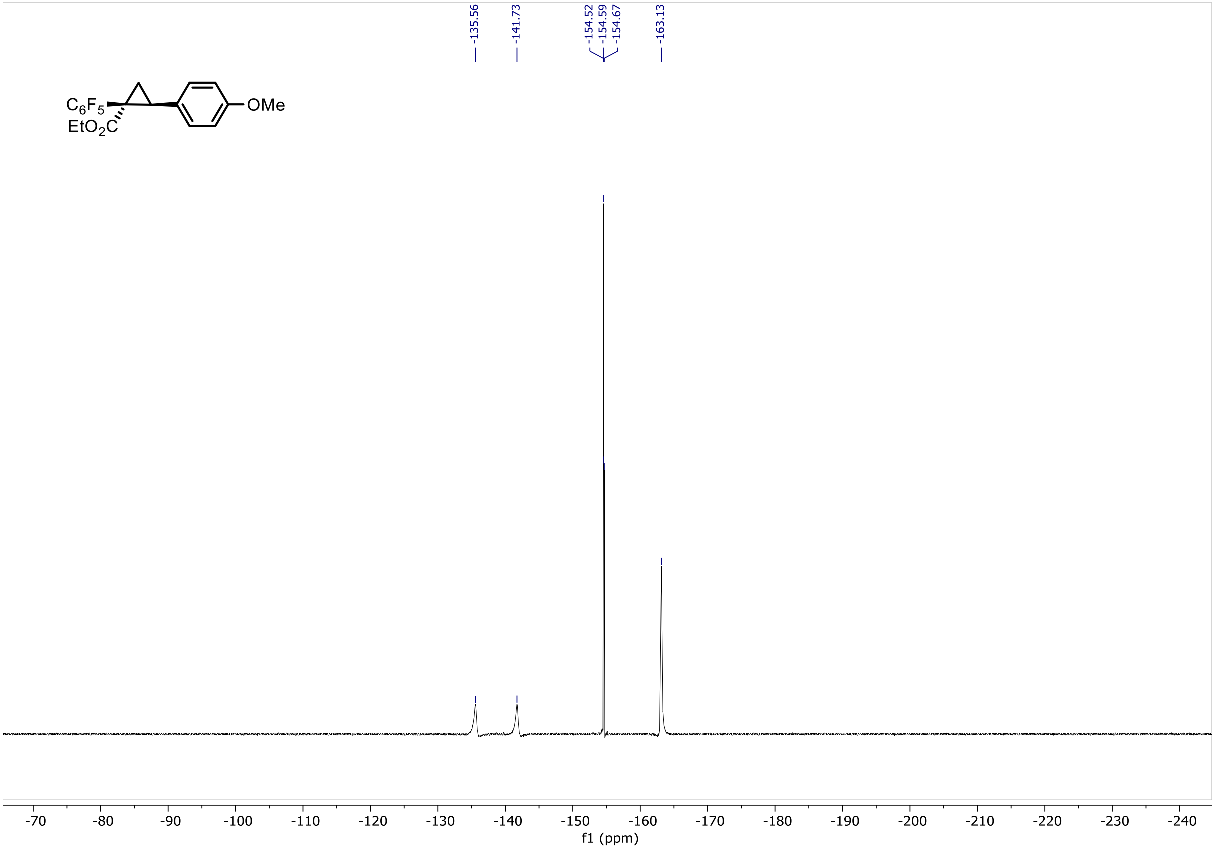

9c: ^{1}H NMR (400 MHz, $CDCl_3$)

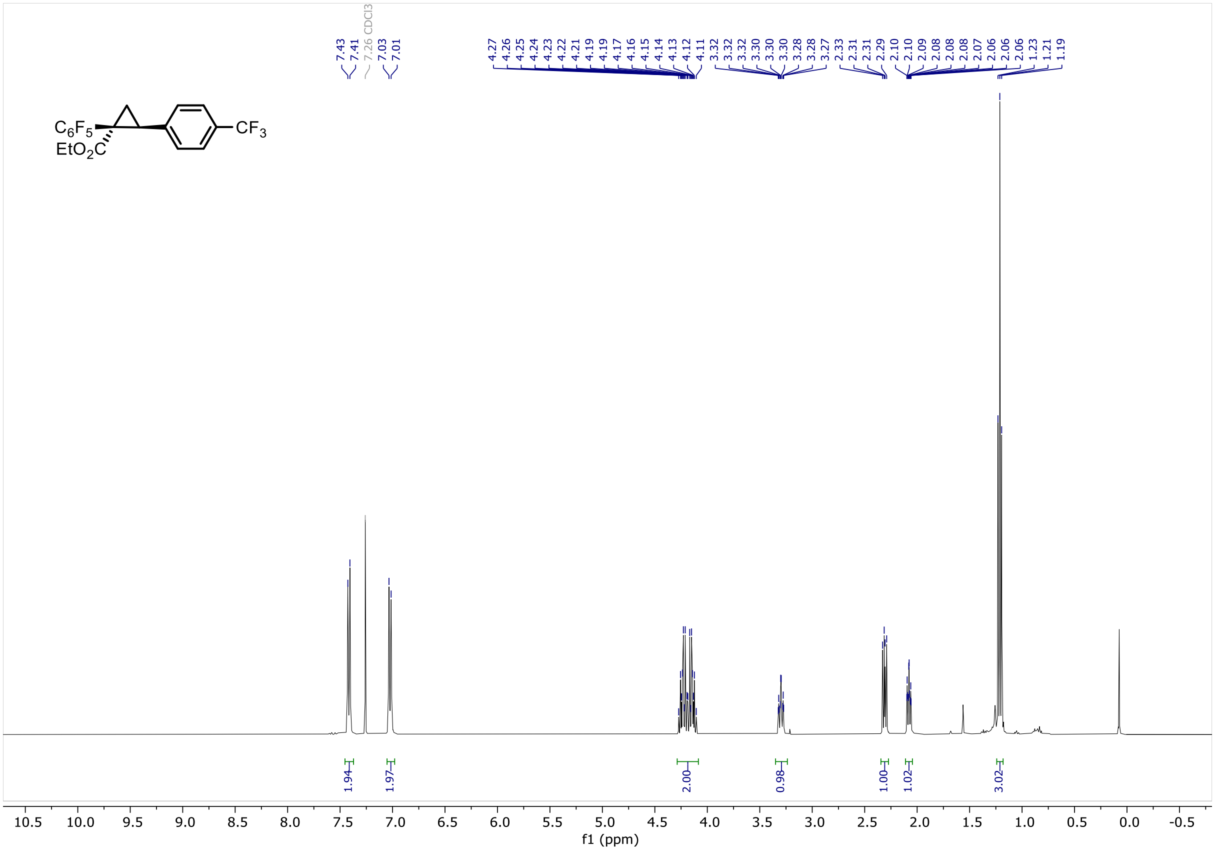

9c: ^{13}C NMR (101 MHz, $CDCl_3$)

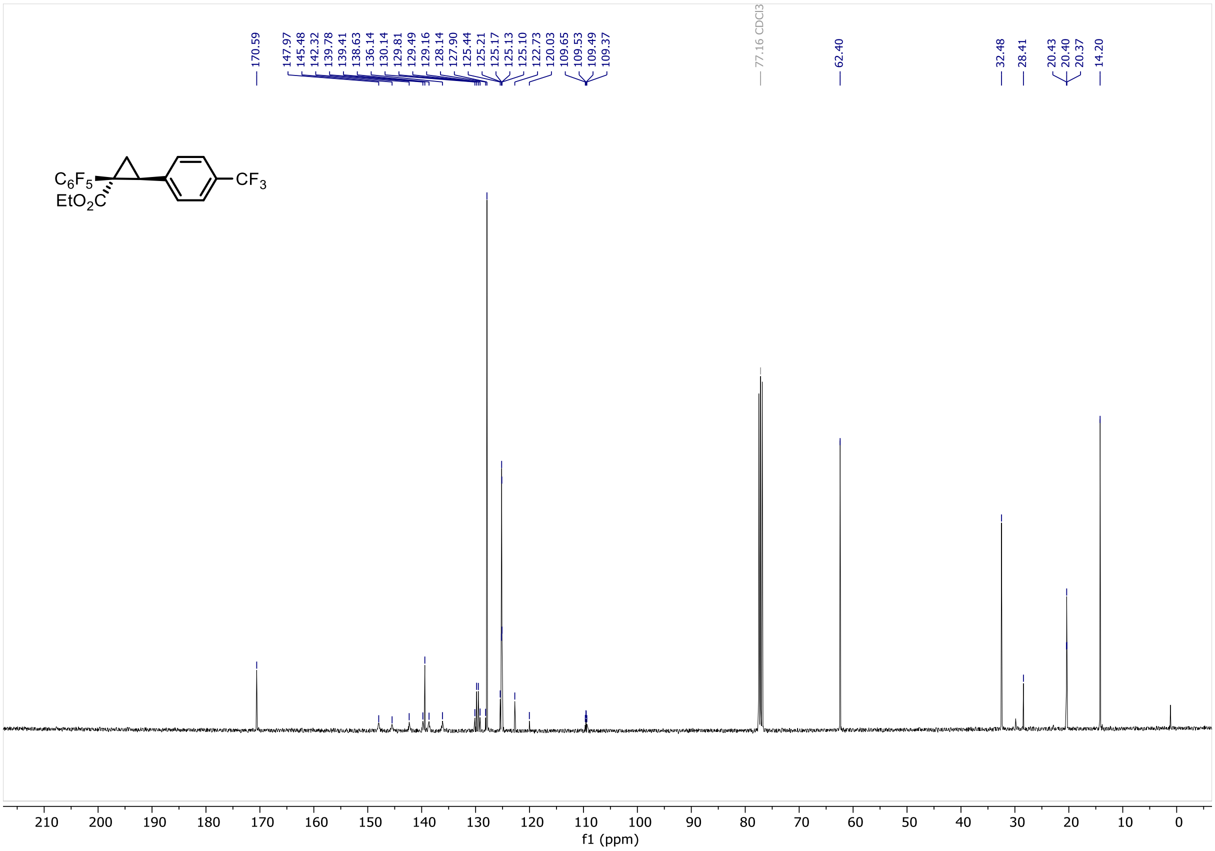

9c: ^{19}F NMR (565 MHz, $CDCl_3$)

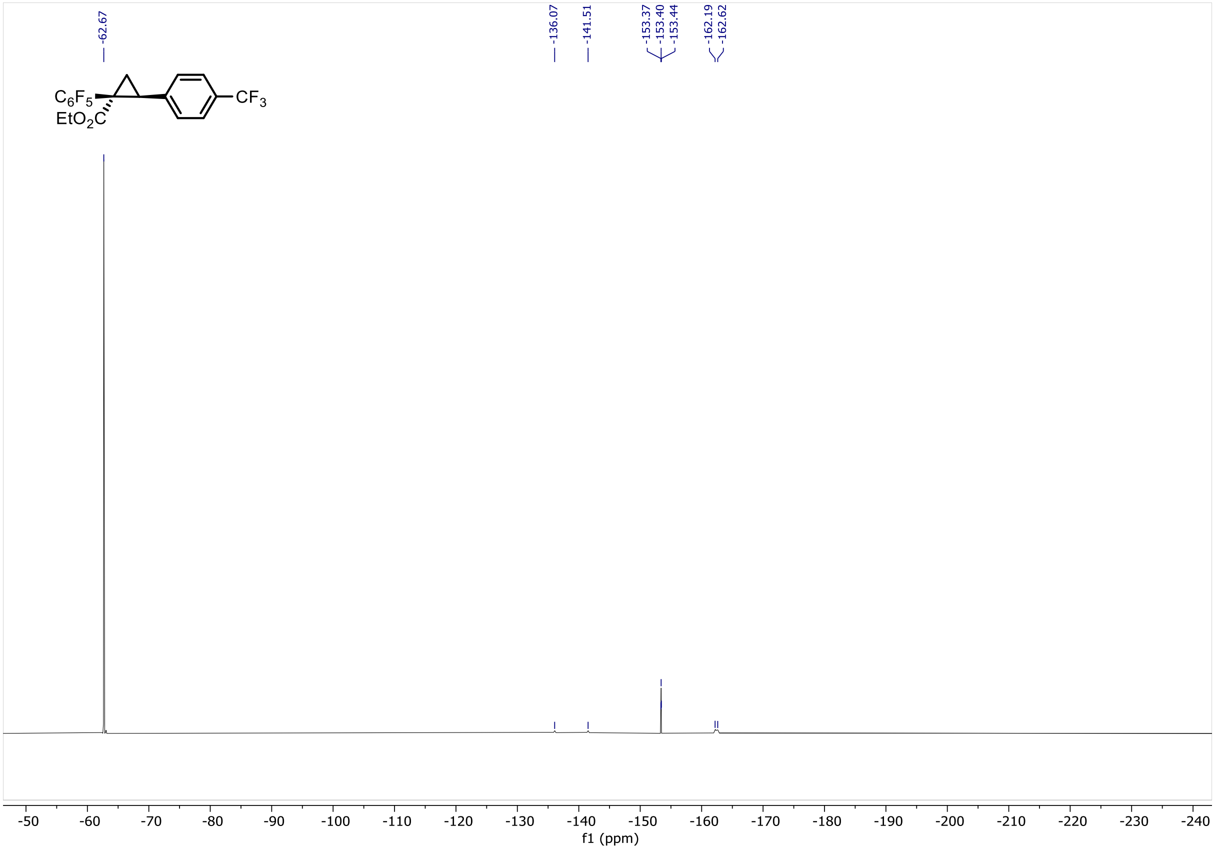

9d: ^{1}H NMR (400 MHz, $CDCl_3$)

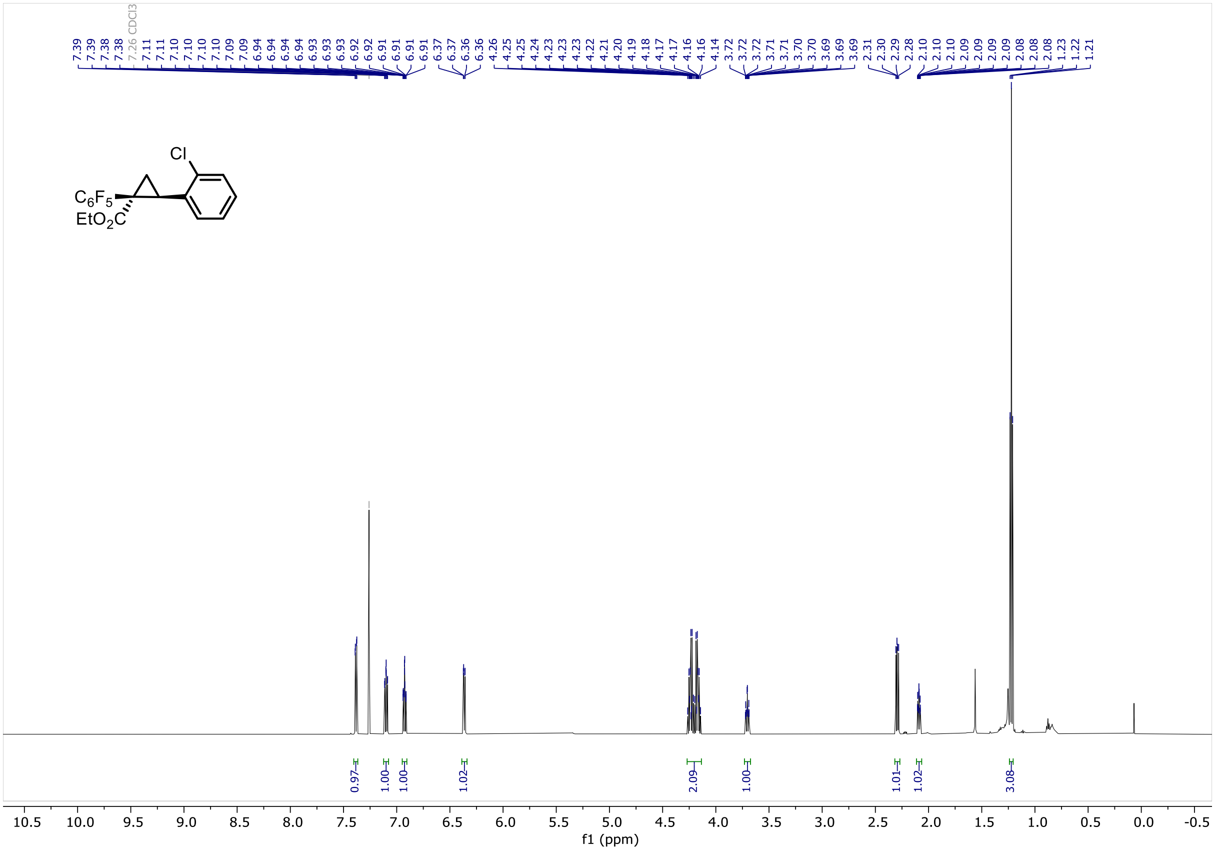

9d: ^{13}C NMR (151 MHz, $CDCl_3$)

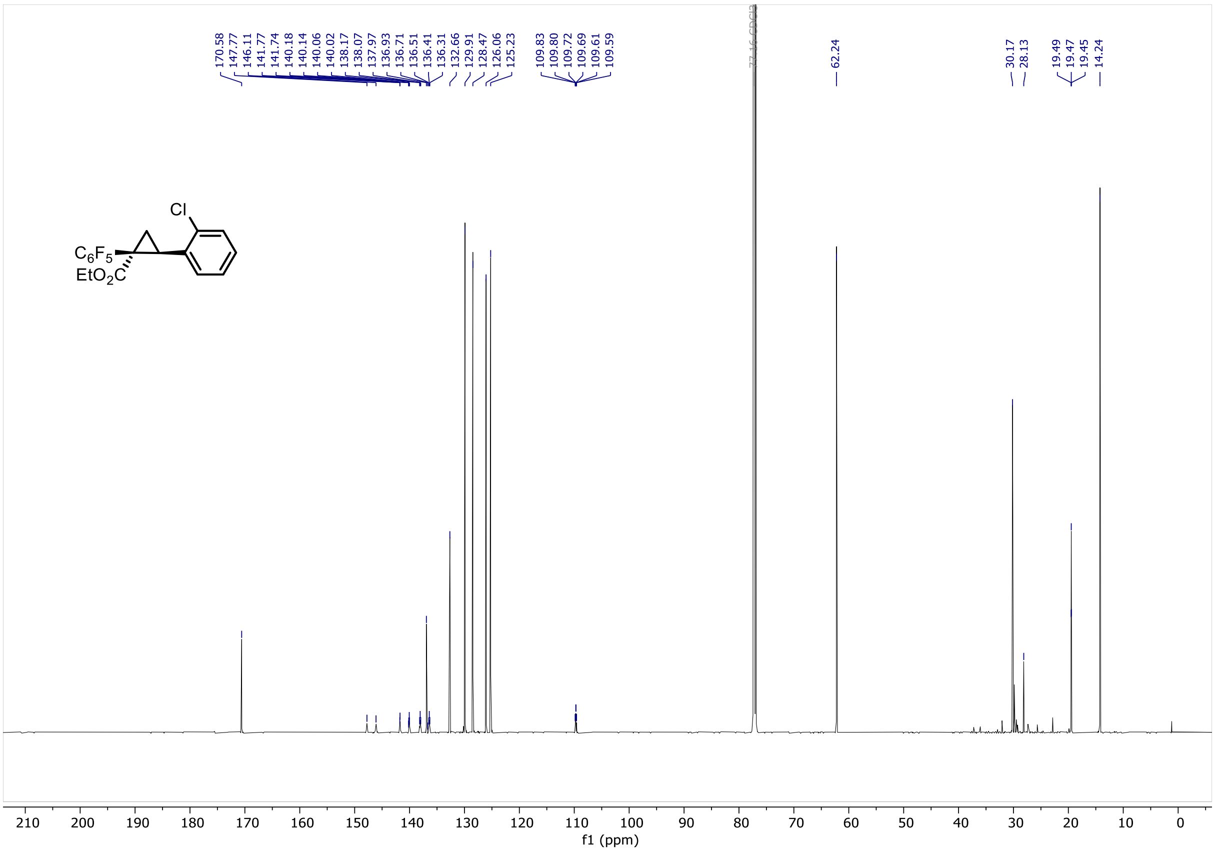

9d: ^{19}F NMR (376 MHz, $CDCl_3$)

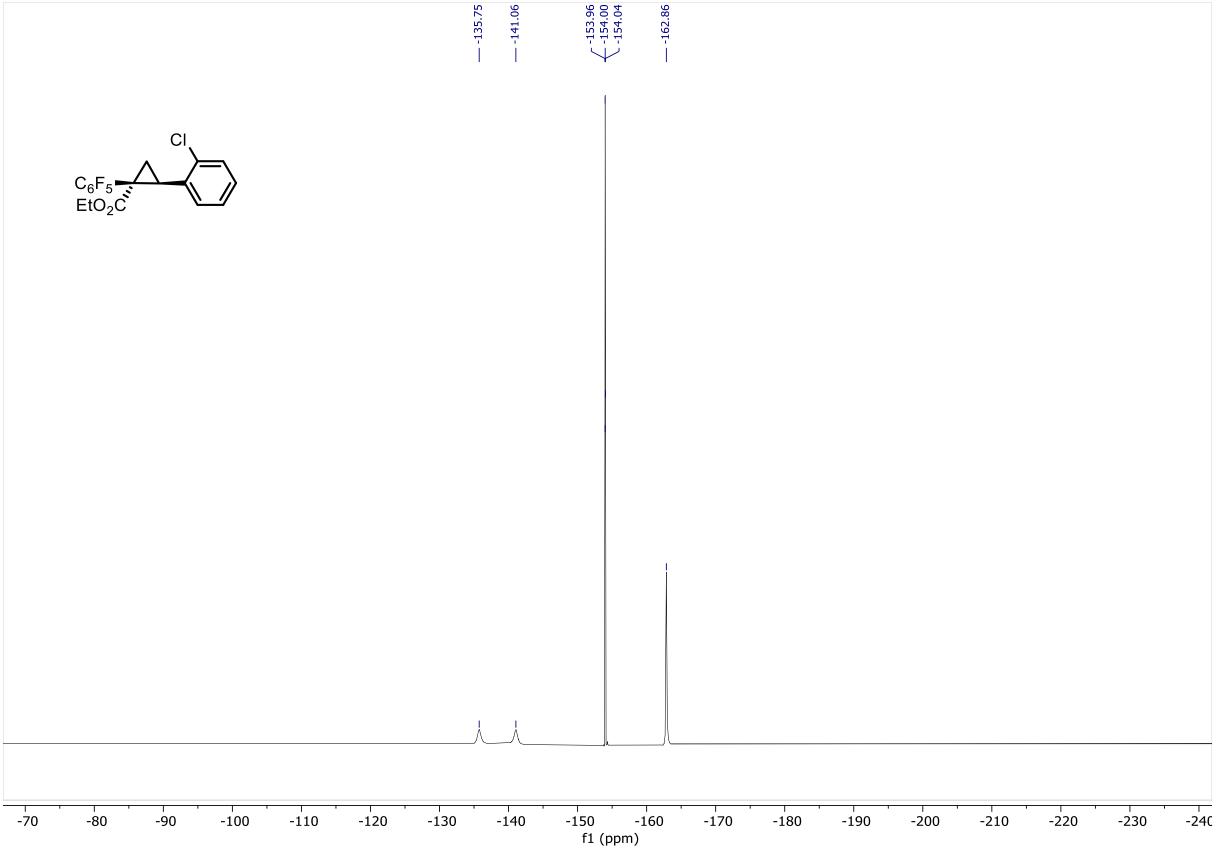

9e: ^{1}H NMR (400 MHz, $CDCl_3$)

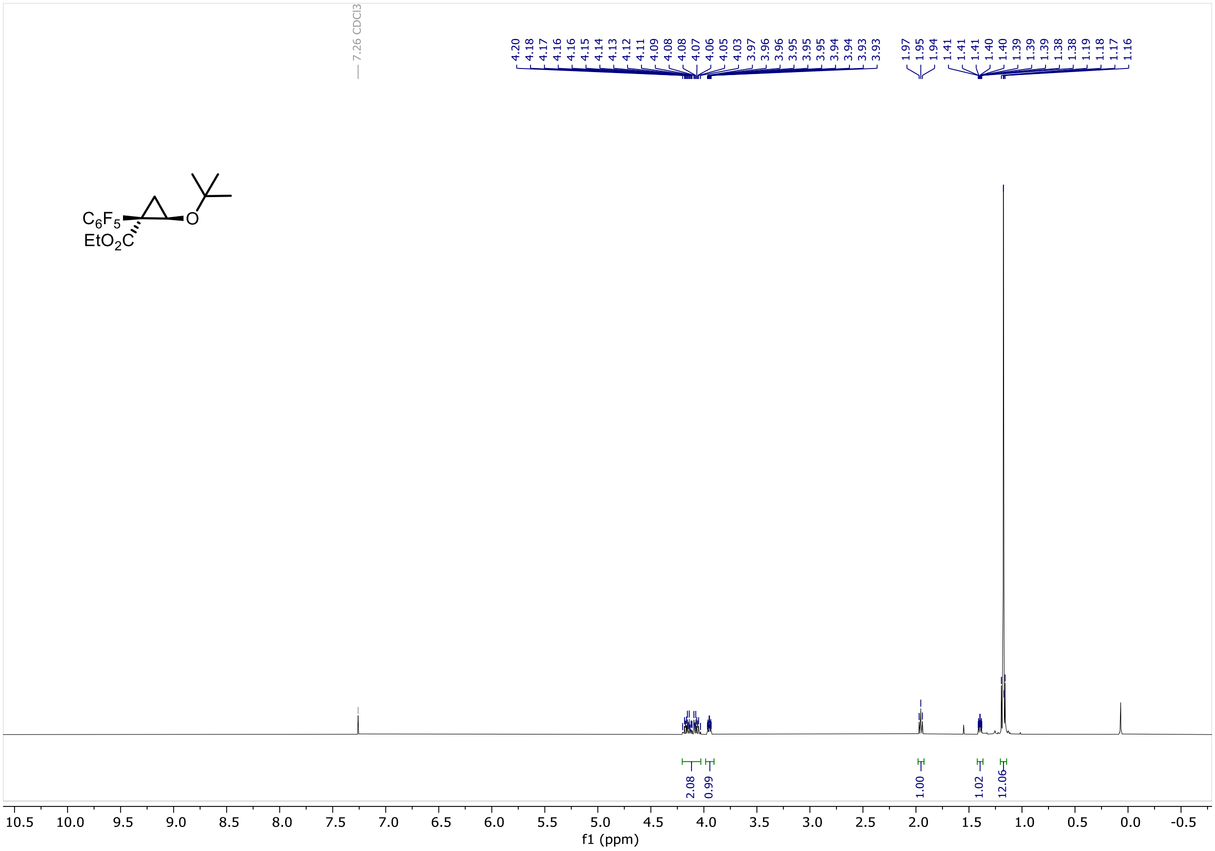

9e: ^{13}C NMR (101 MHz, $CDCl_3$)

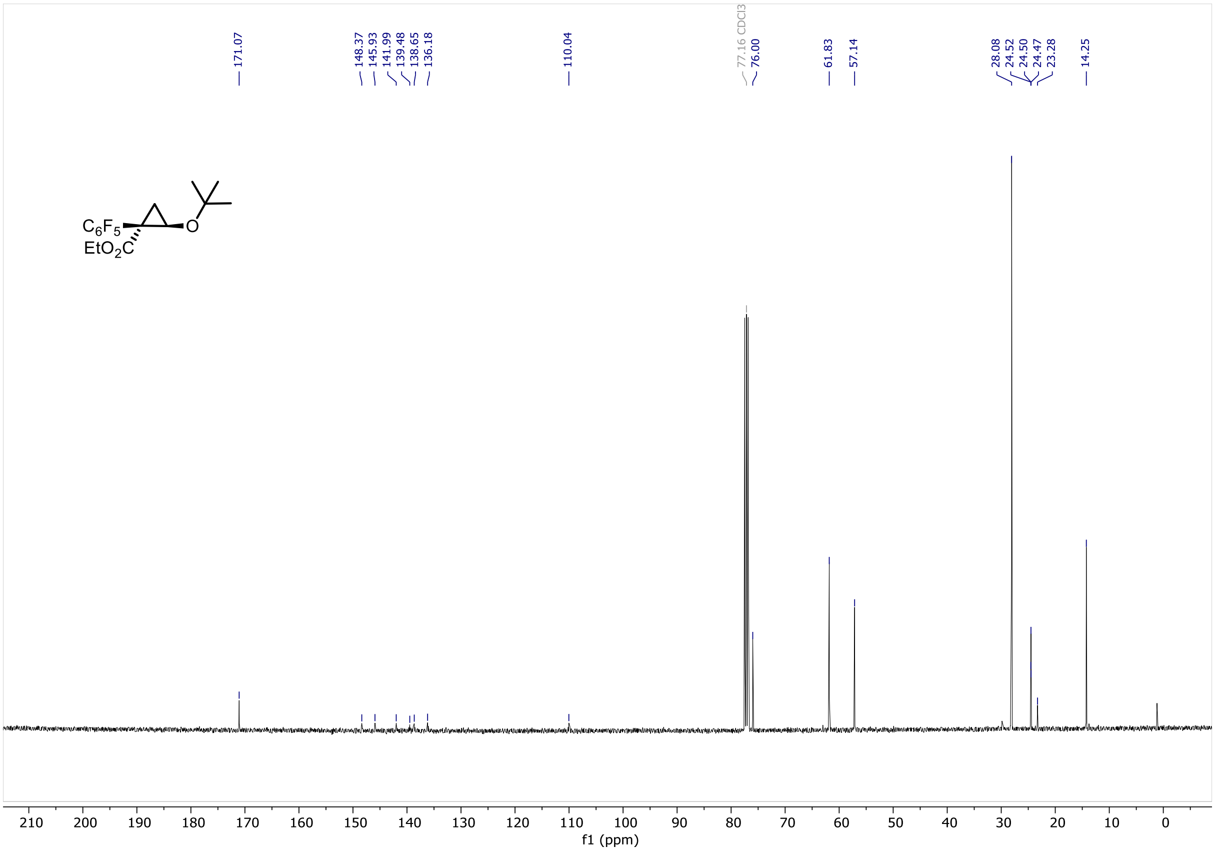

9e: ^{19}F NMR (376 MHz, $CDCl_3$)

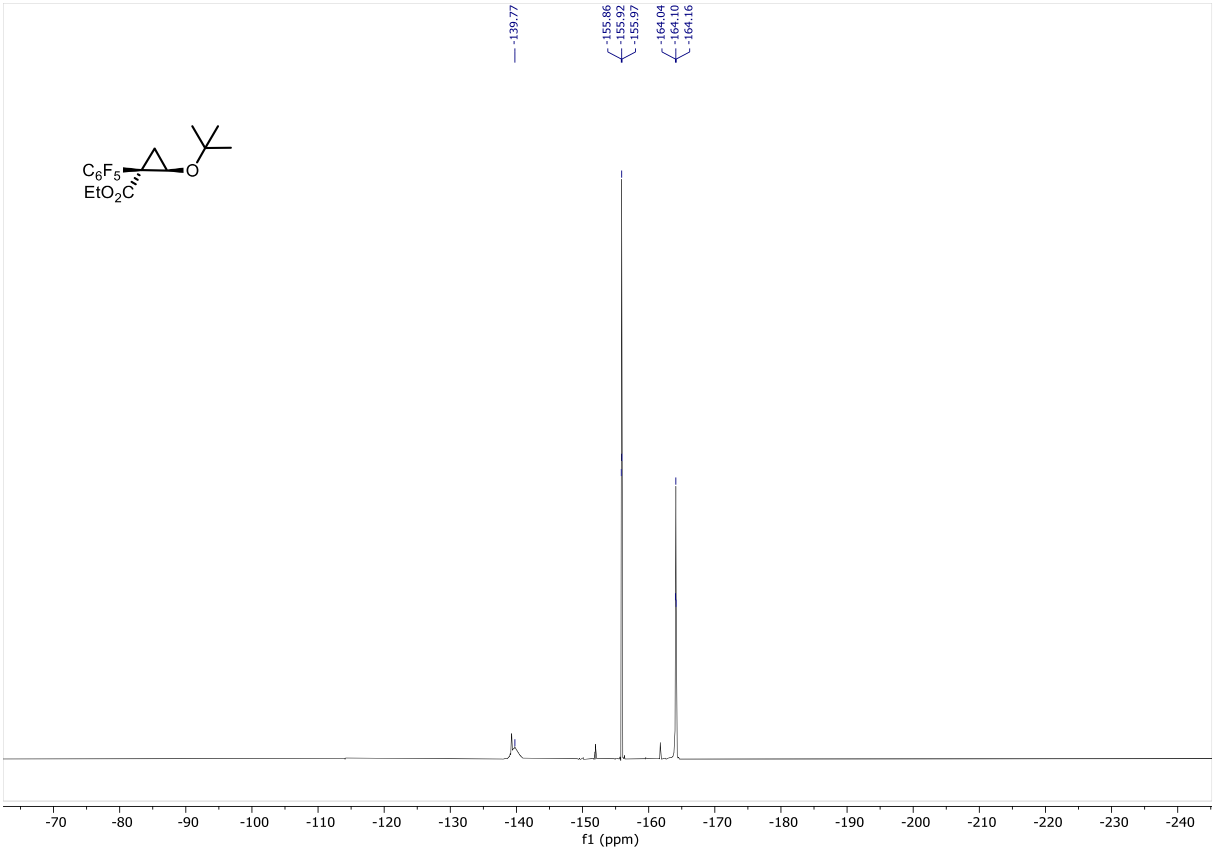

9f: ^{1}H NMR (400 MHz, $CDCl_3$)

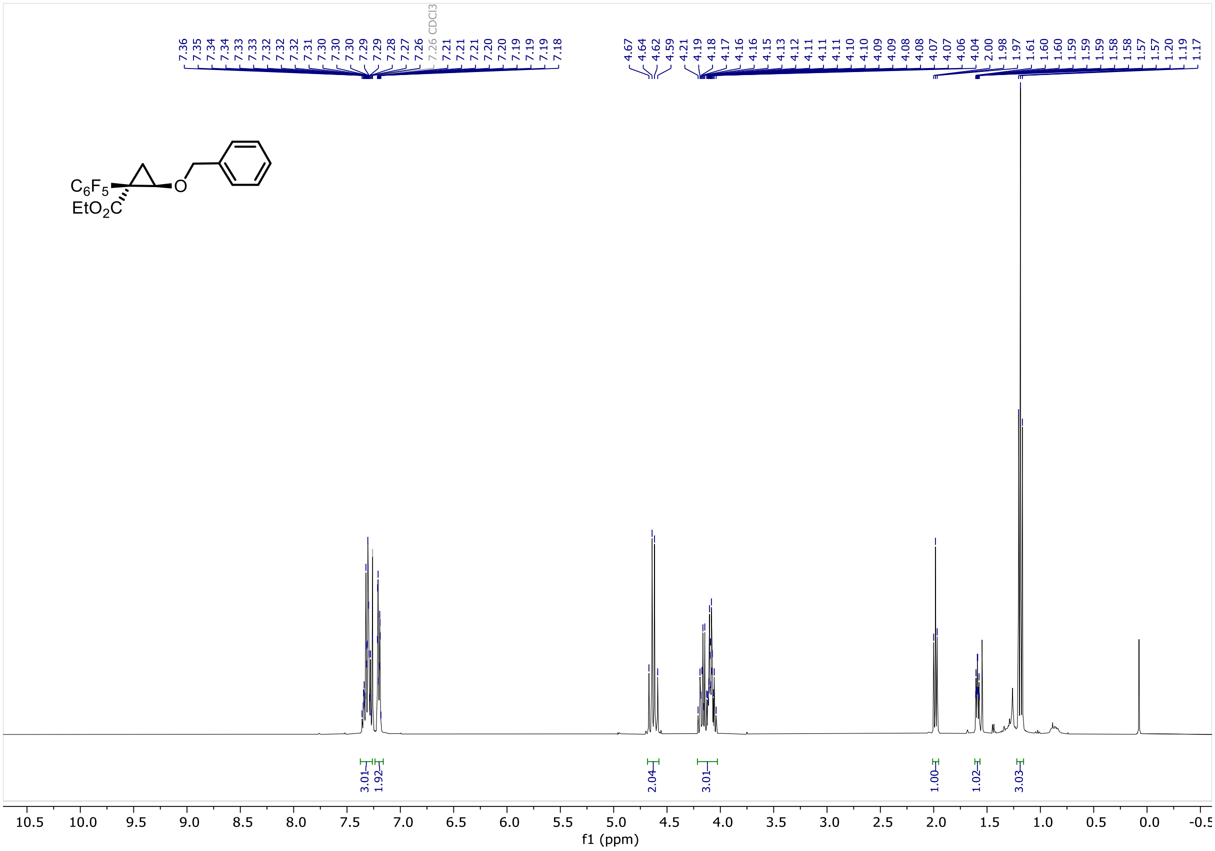

9f: ^{13}C NMR (101 MHz, $CDCl_3$)

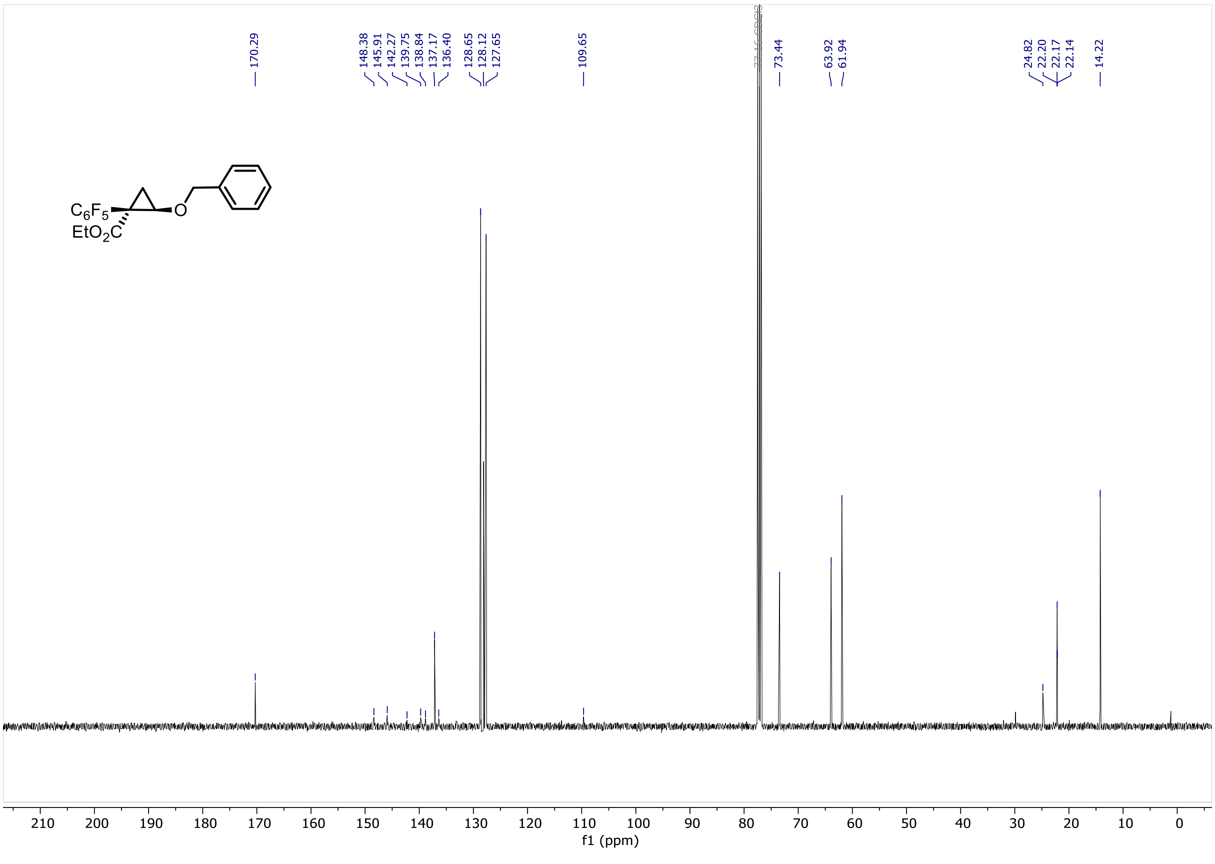

9f: ^{19}F NMR (565 MHz, $CDCl_3$)

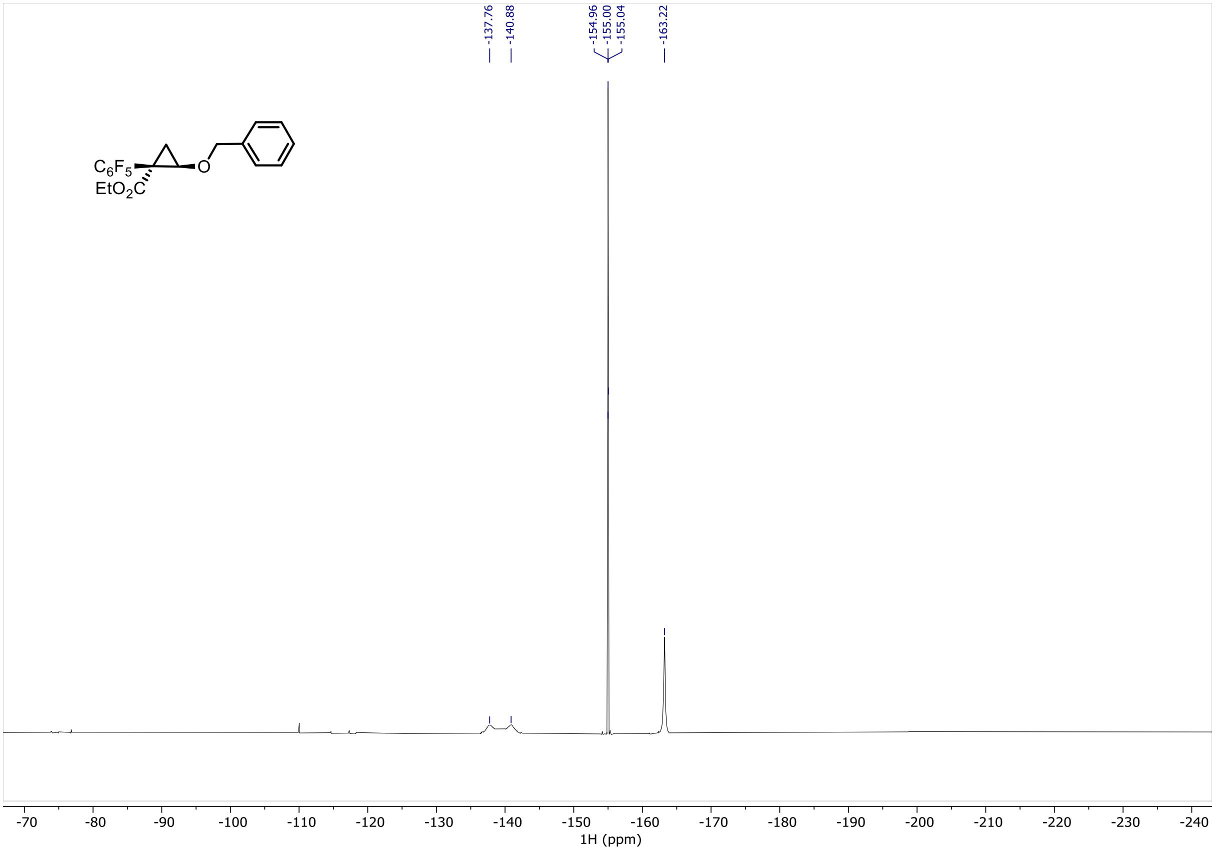

9g: ^{1}H NMR (400 MHz, $CDCl_3$)

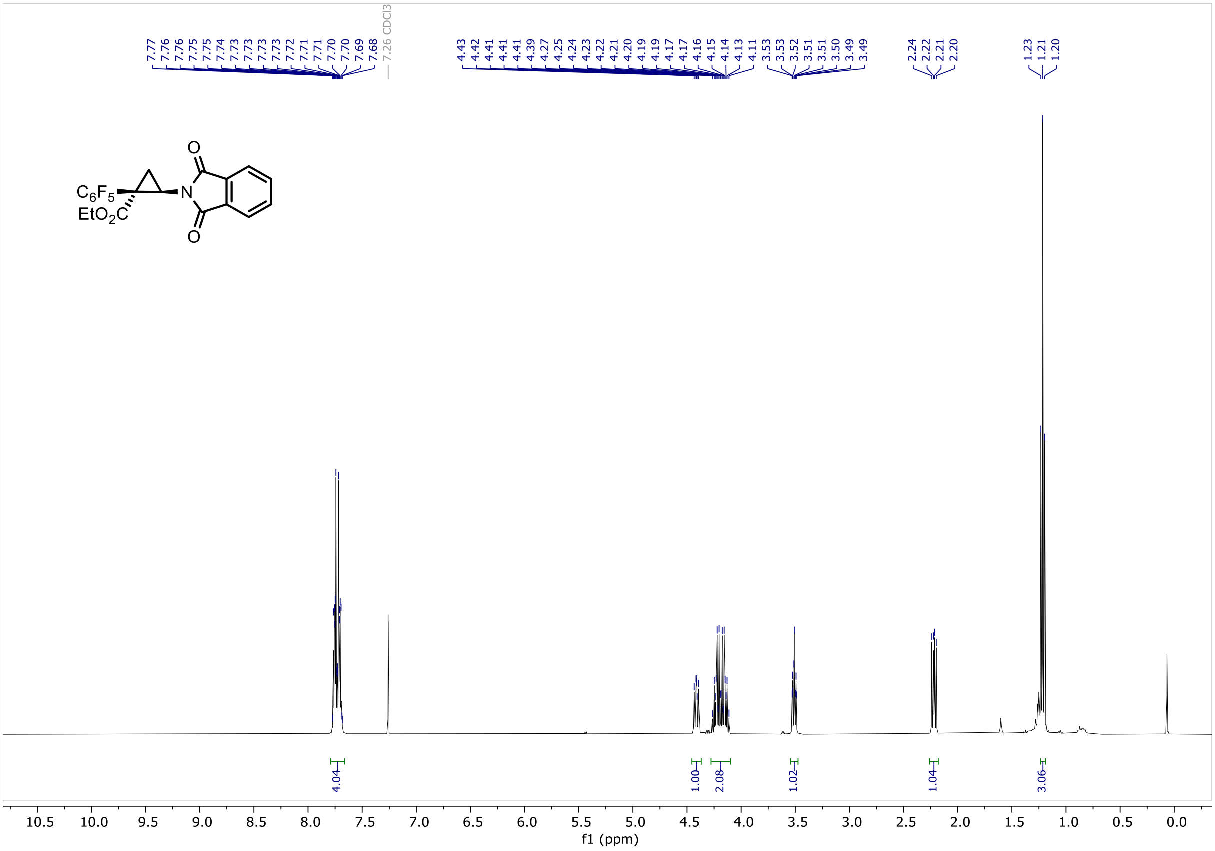

9g: ^{13}C NMR (101 MHz, $CDCl_3$)

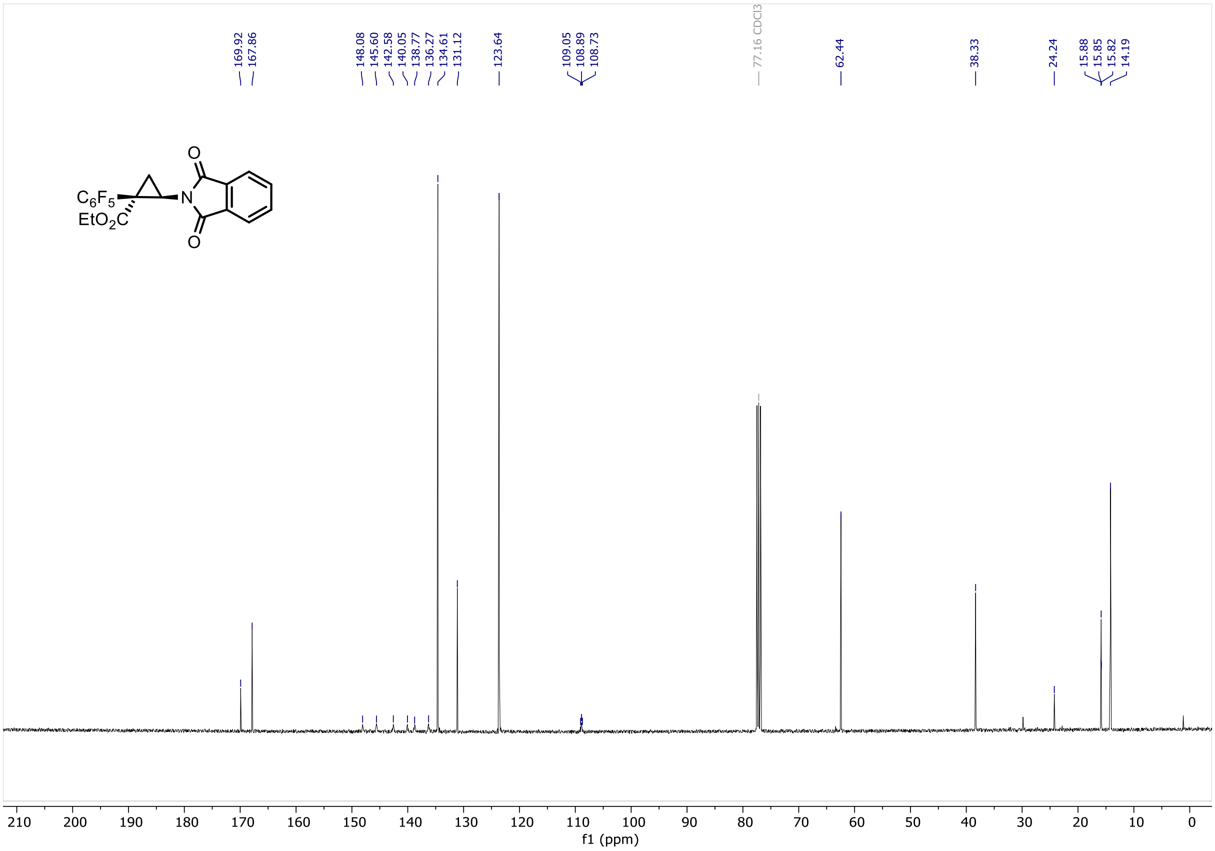

9g: ^{19}F NMR (376 MHz, $CDCl_3$)

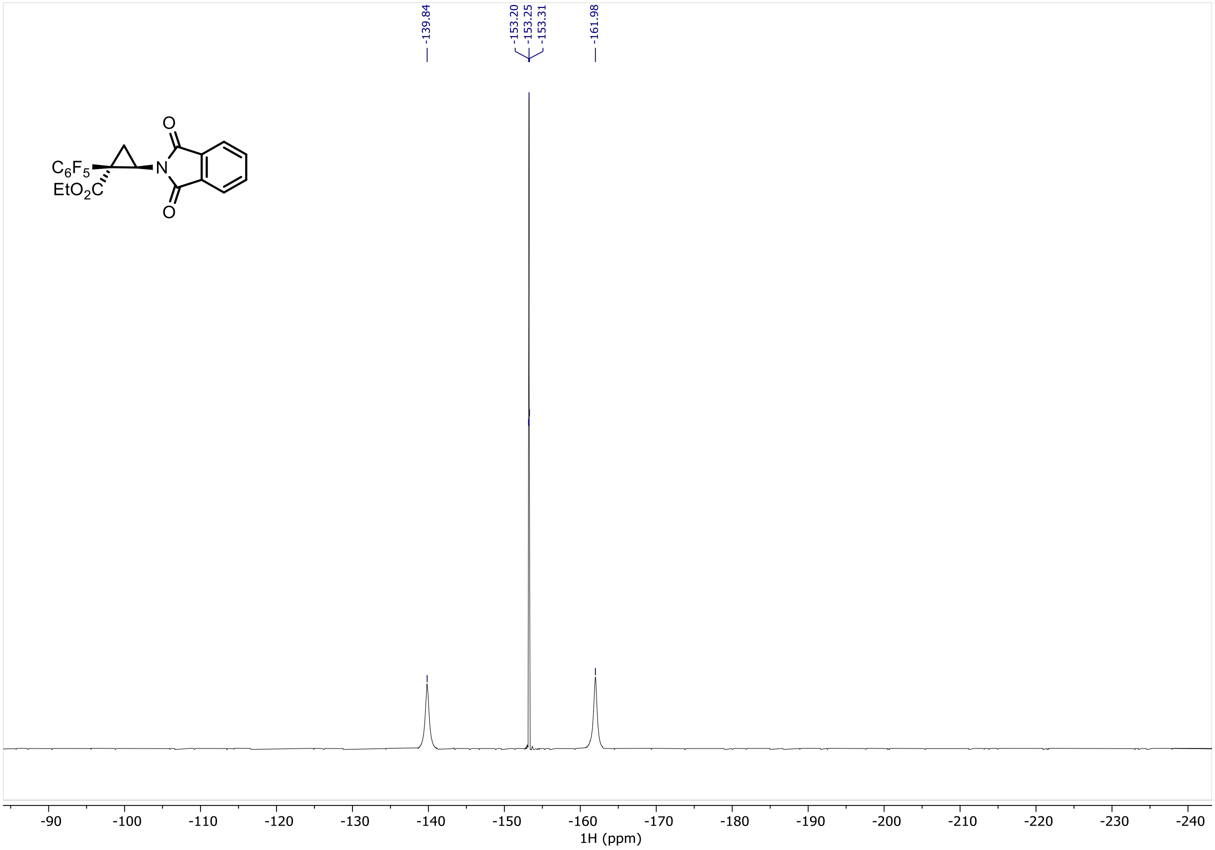

9h: ^{1}H NMR (400 MHz, $CDCl_3$)

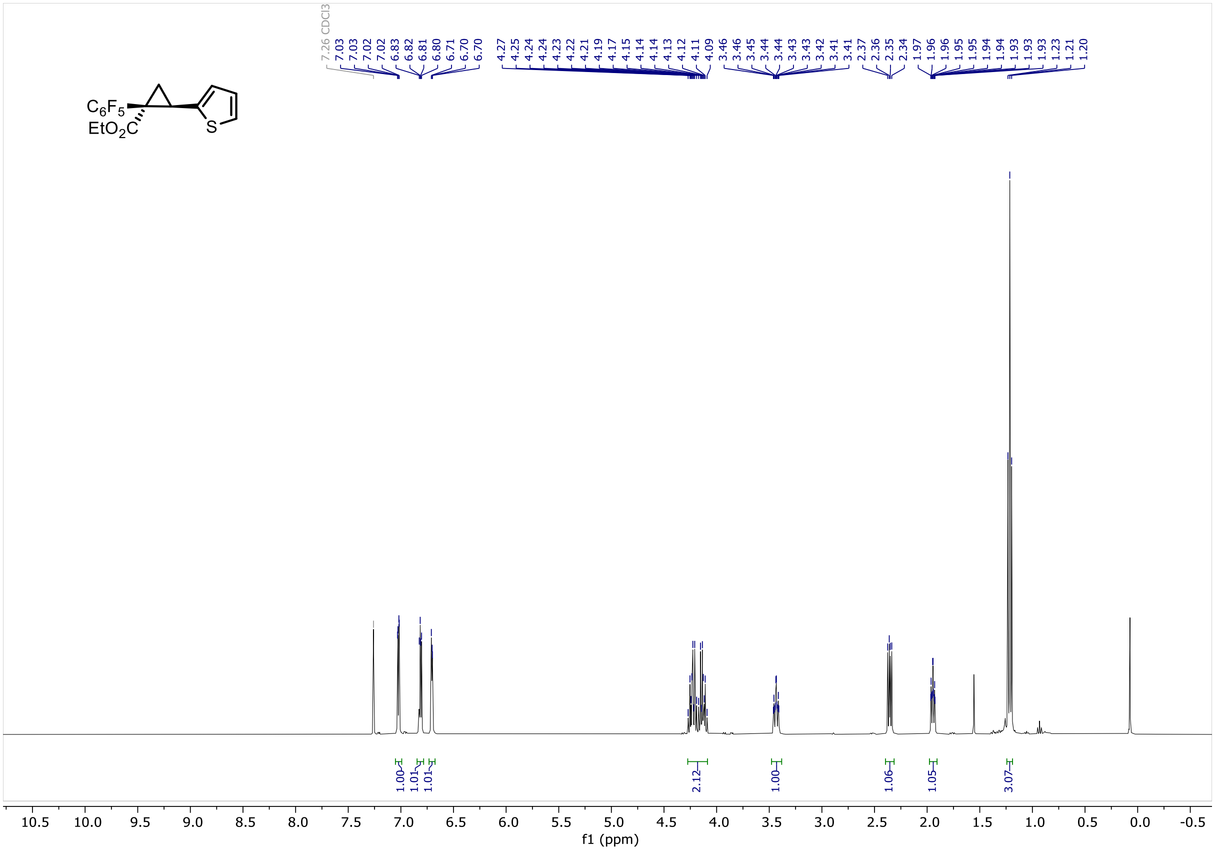

9h: ^{13}C NMR (101 MHz, $CDCl_3$)

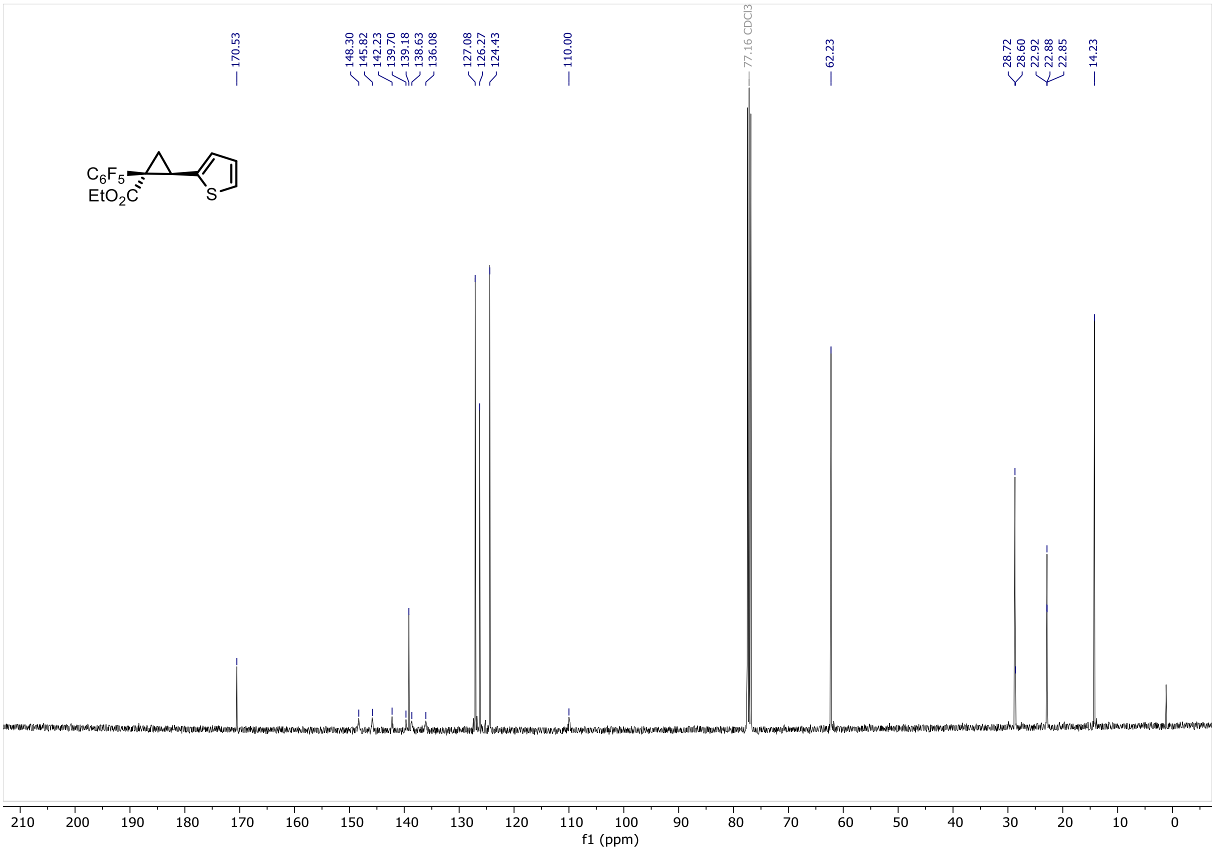

9h: ^{19}F NMR (376 MHz, $CDCl_3$)

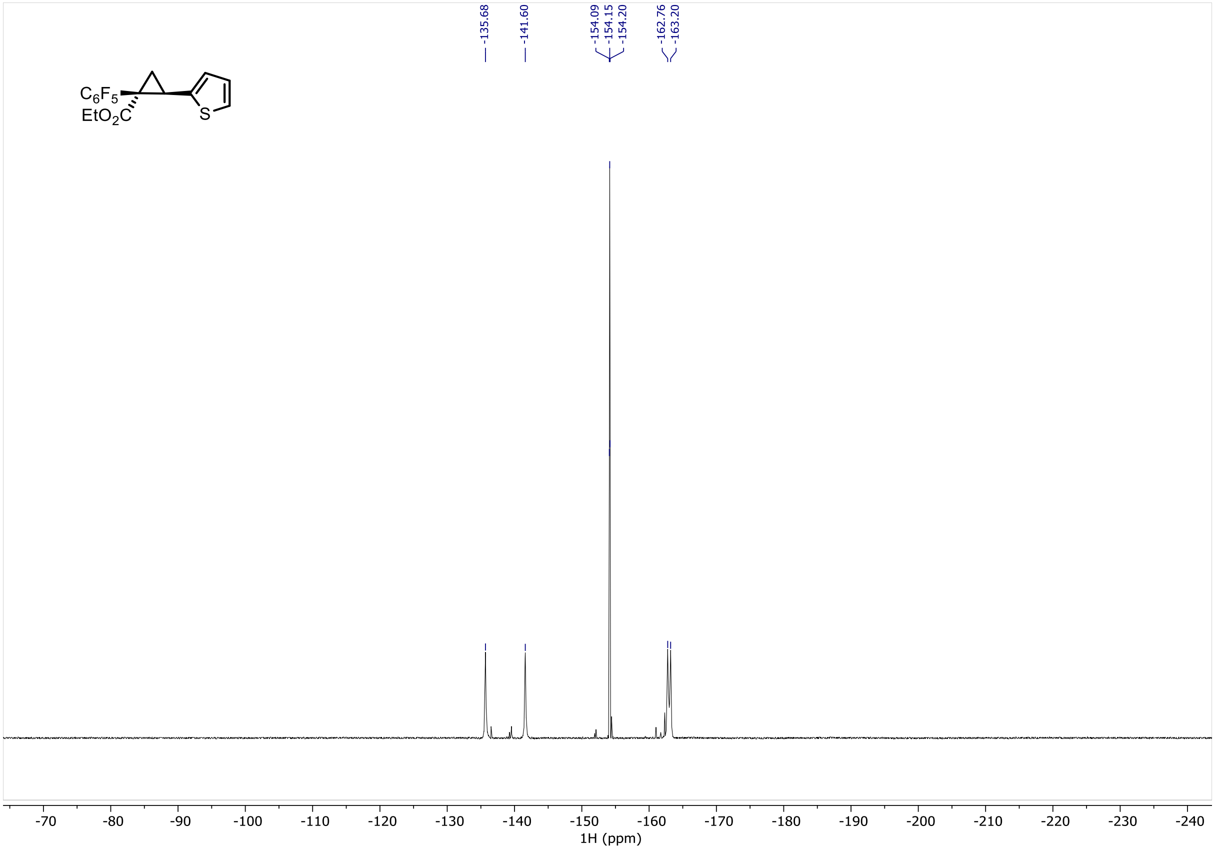

10: ^{1}H NMR (400 MHz, $CDCl_3$)

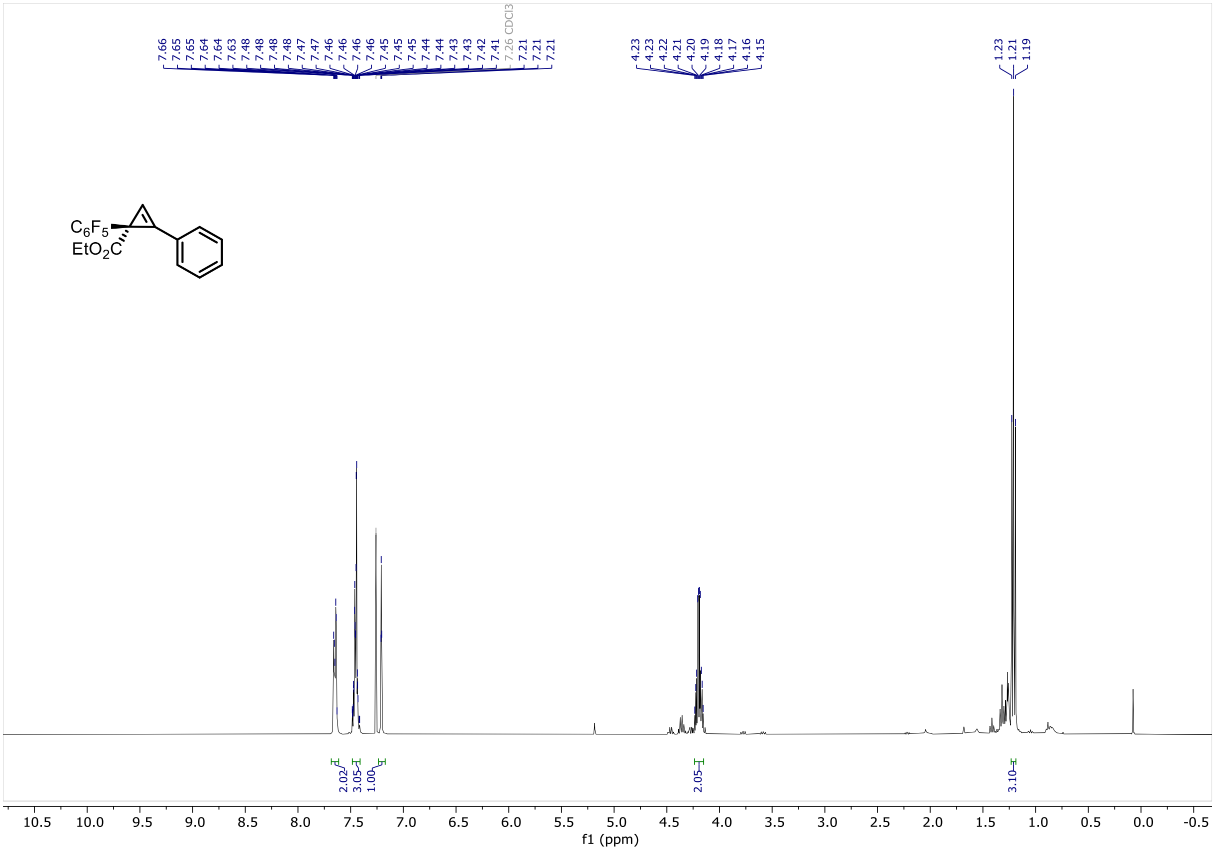

10: ^{13}C NMR (101 MHz, $CDCl_3$)

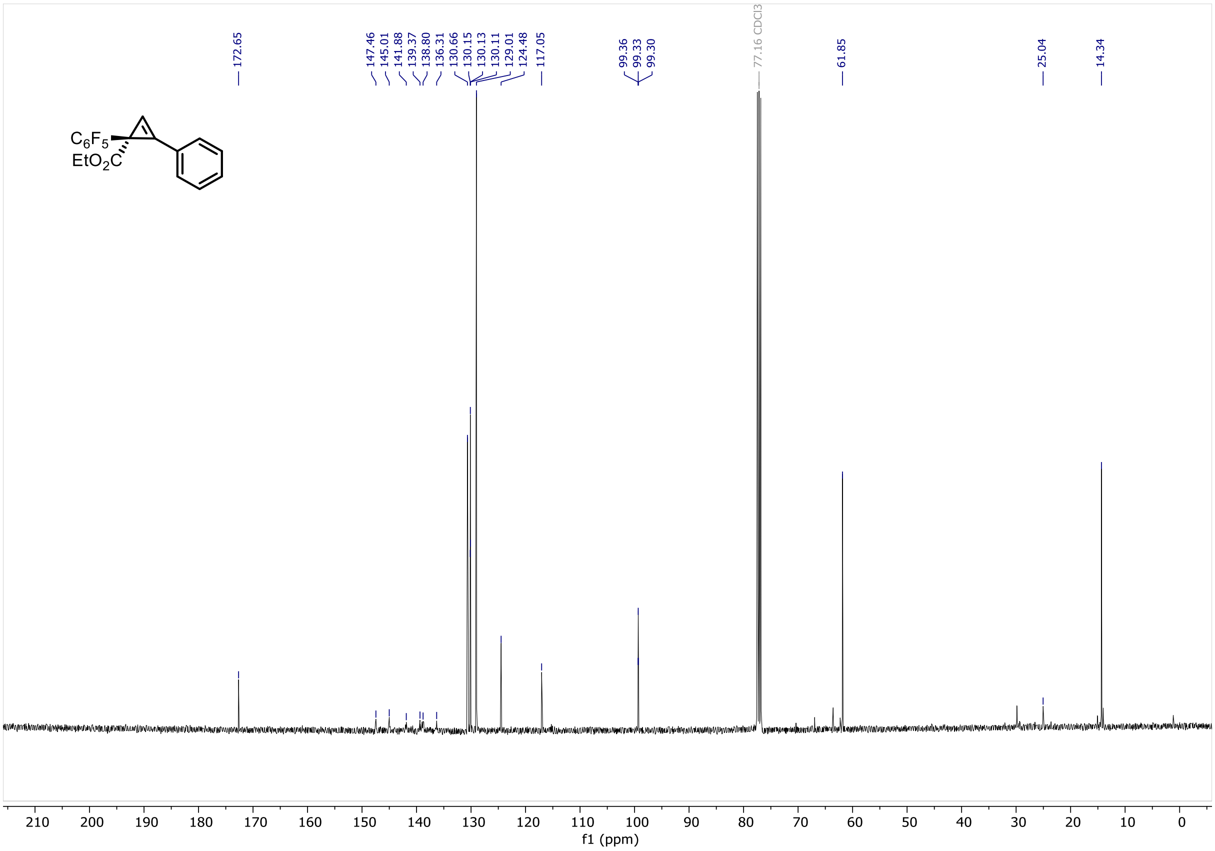

10: ^{19}F NMR (376 MHz, $CDCl_3$)

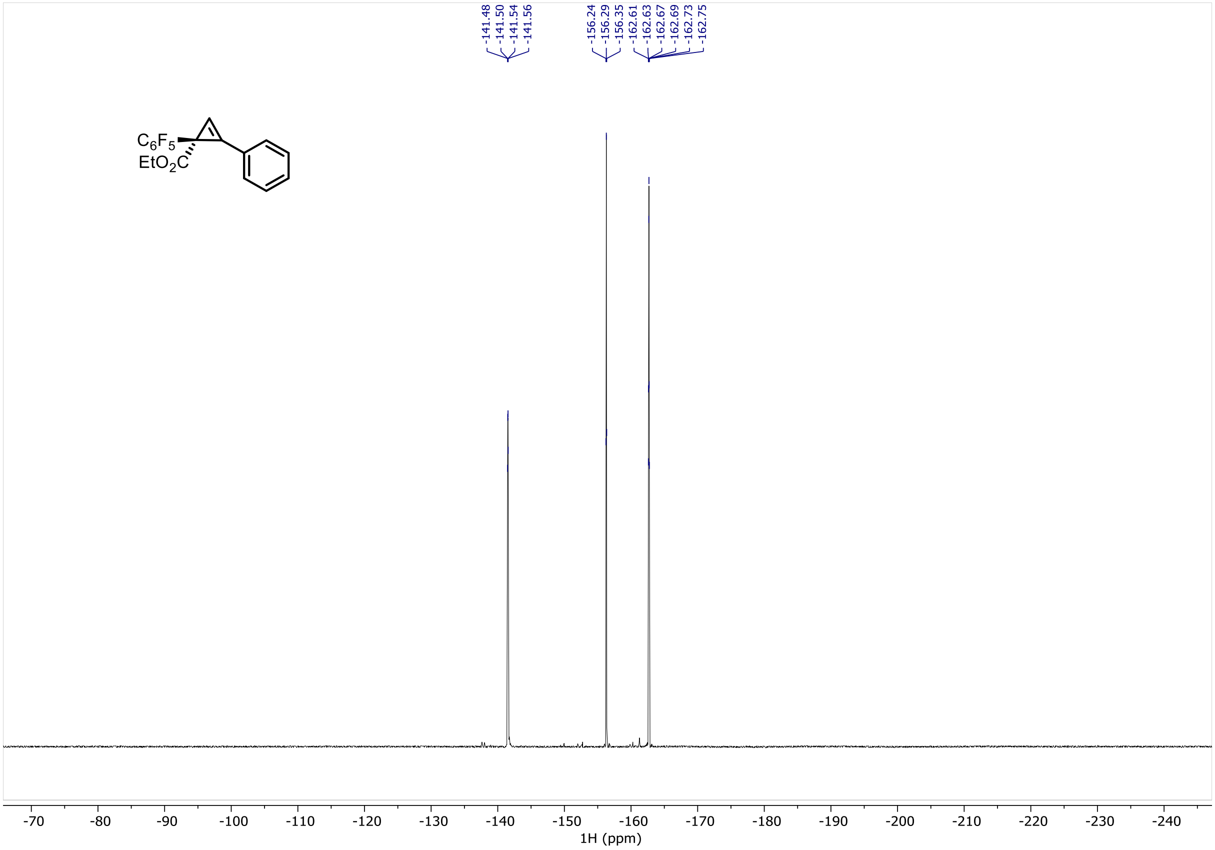

11: ¹H NMR (400 MHz, $CDCl_3$)

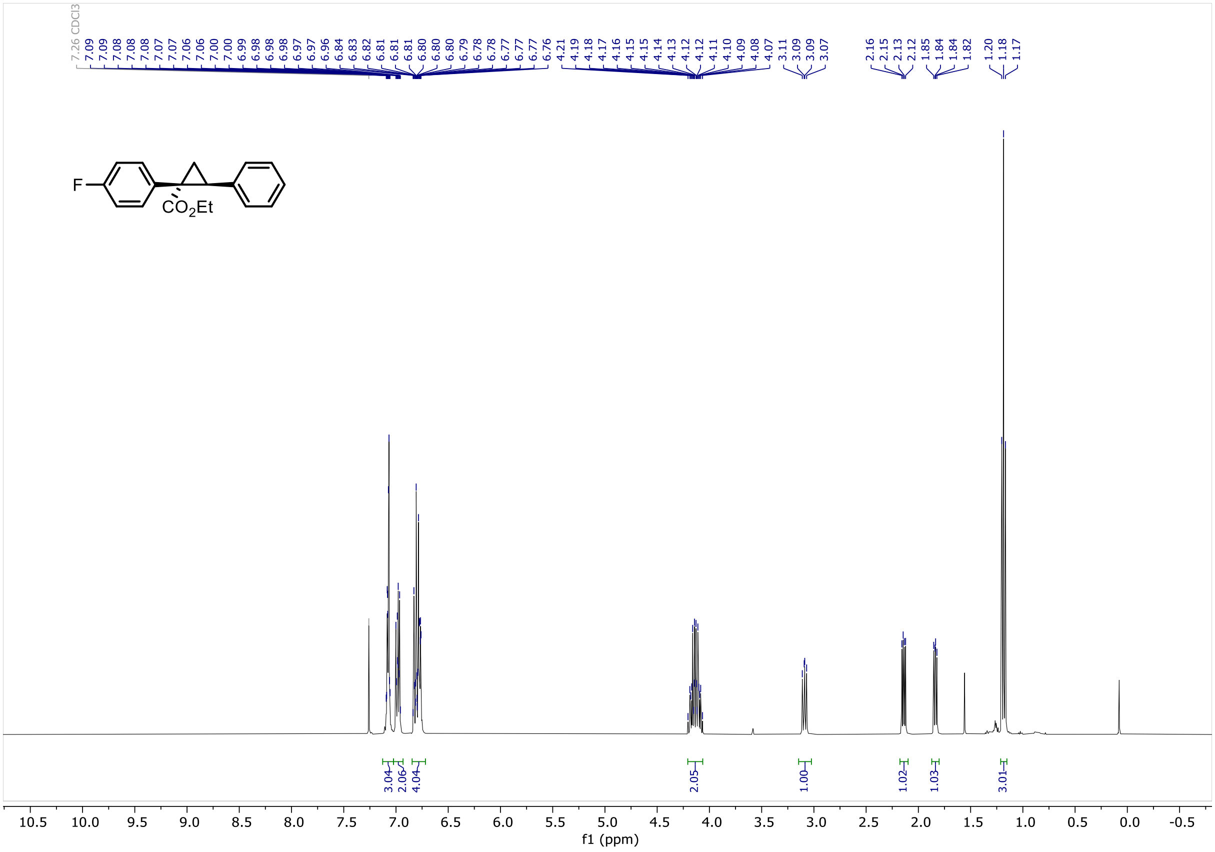

11: ¹³C NMR (101 MHz, $CDCl_3$)

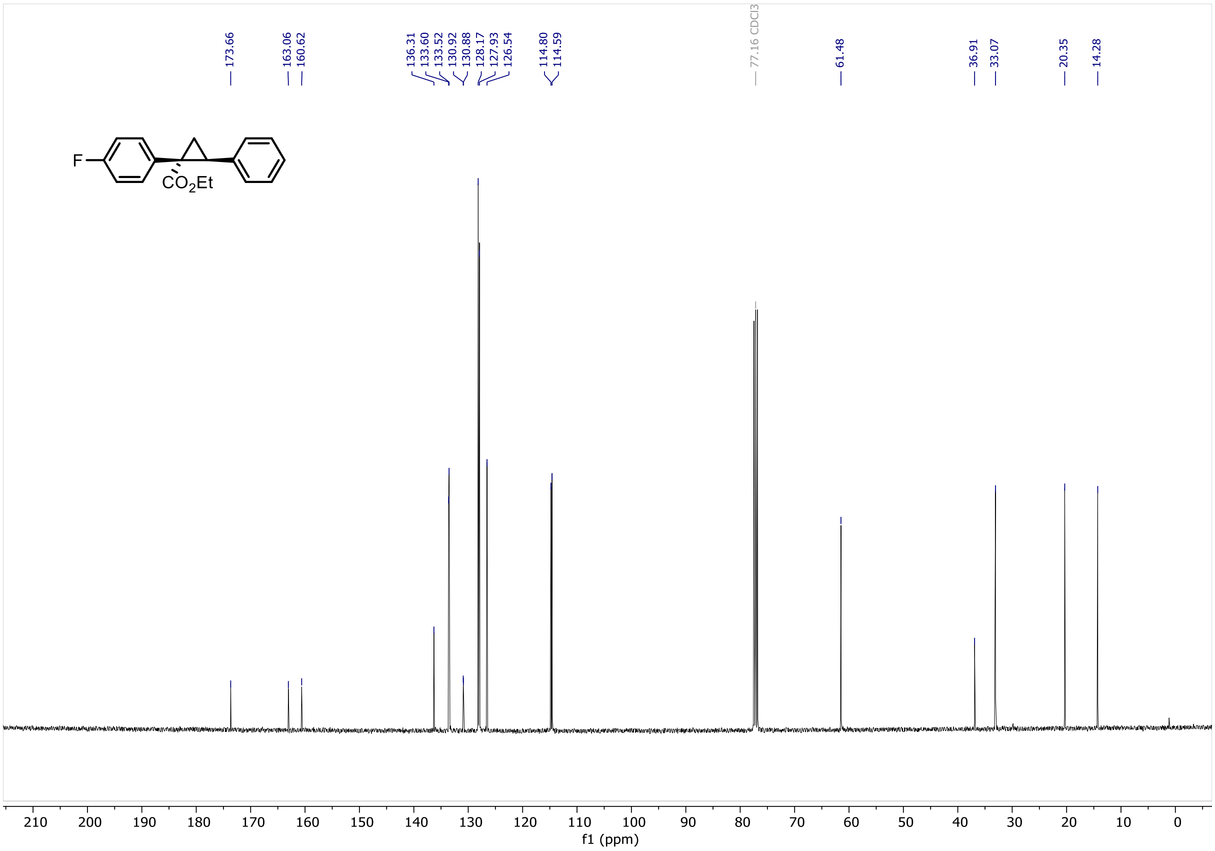

11: ^{19}F NMR (282 MHz, $CDCl_3$)

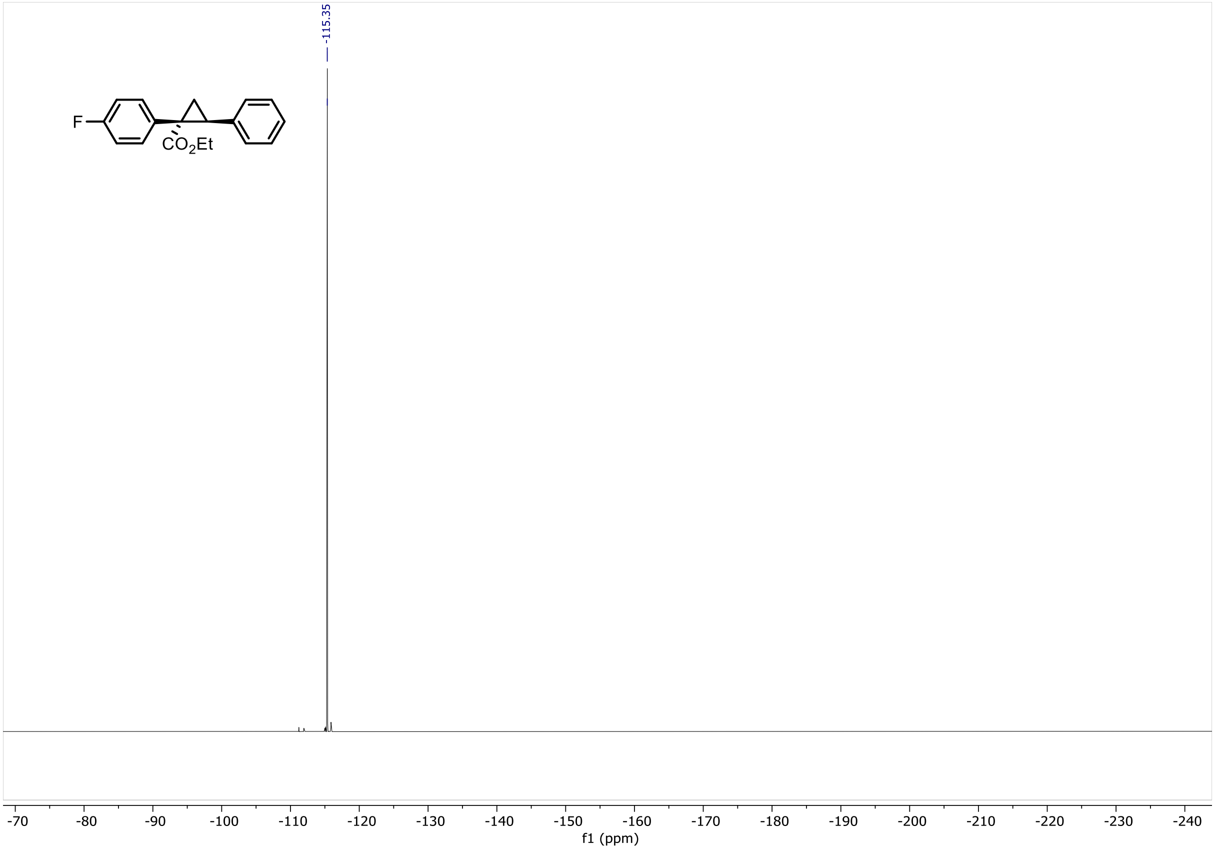

12: ^{1}H NMR (400 MHz, $CDCl_3$)

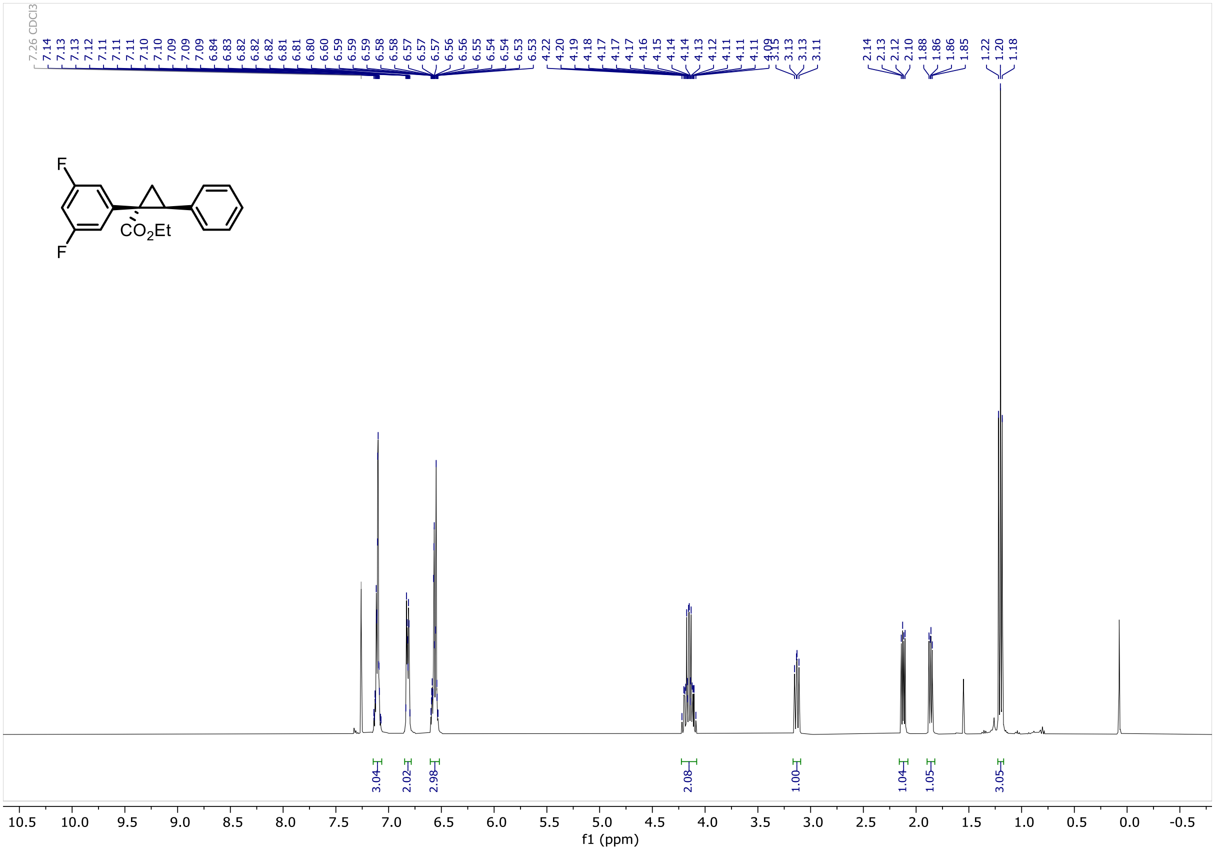

12: ^{13}C NMR (101 MHz, $CDCl_3$)

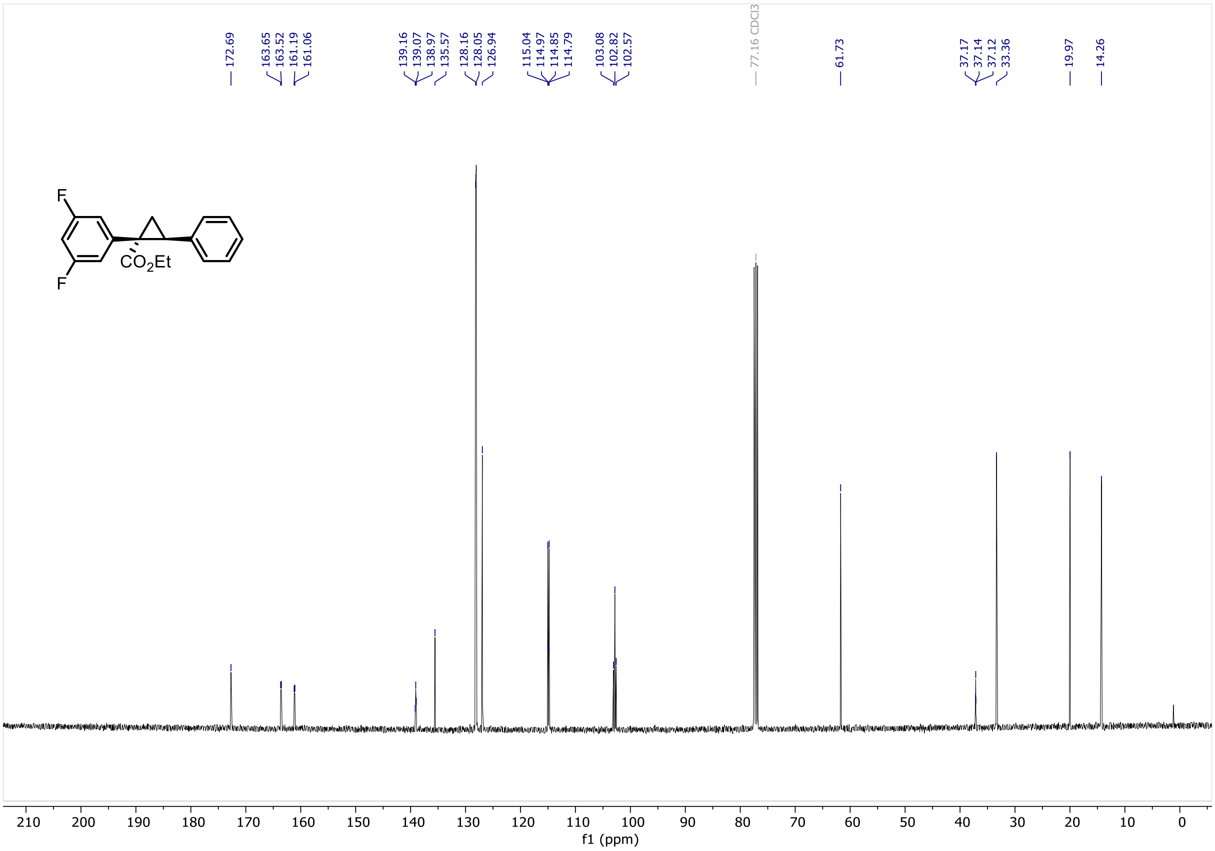

12: ^{19}F NMR (282 MHz, $CDCl_3$)

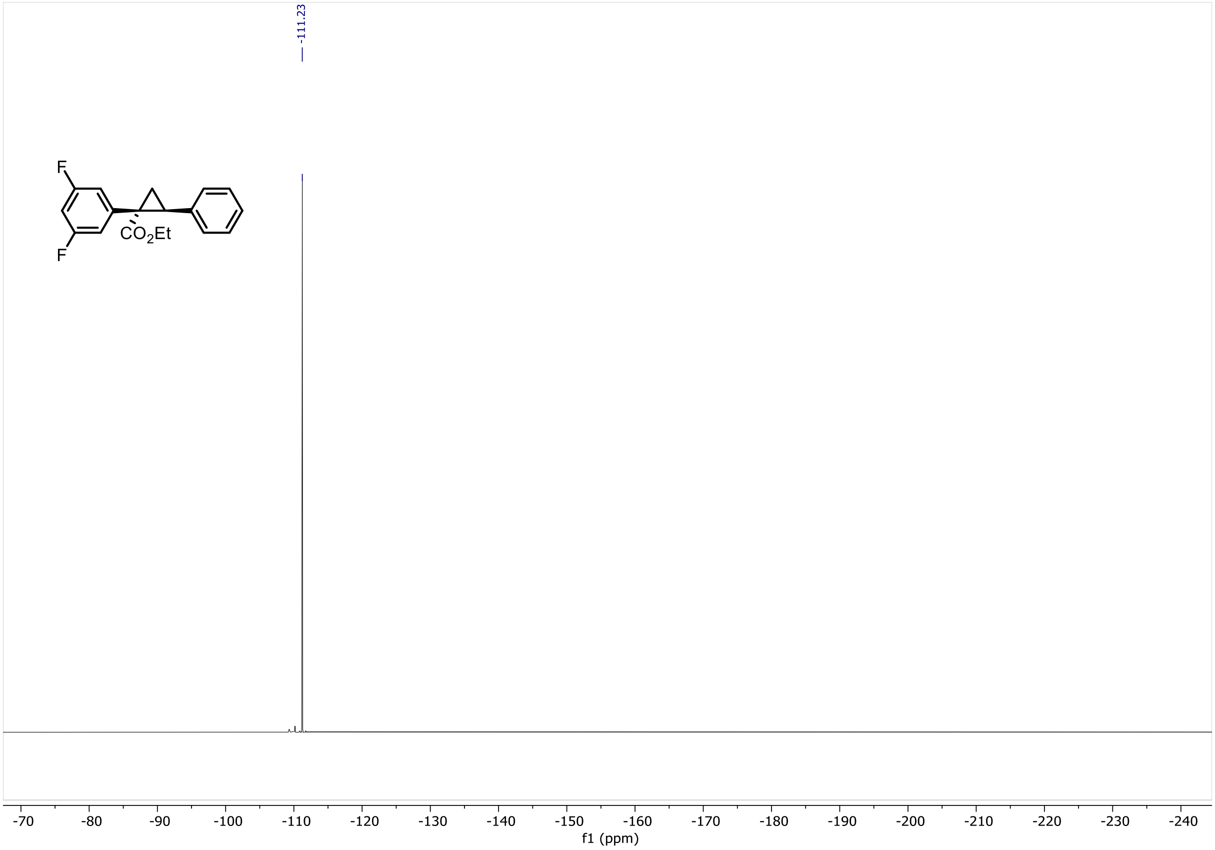

13: ^{1}H NMR (400 MHz, $CDCl_3$)

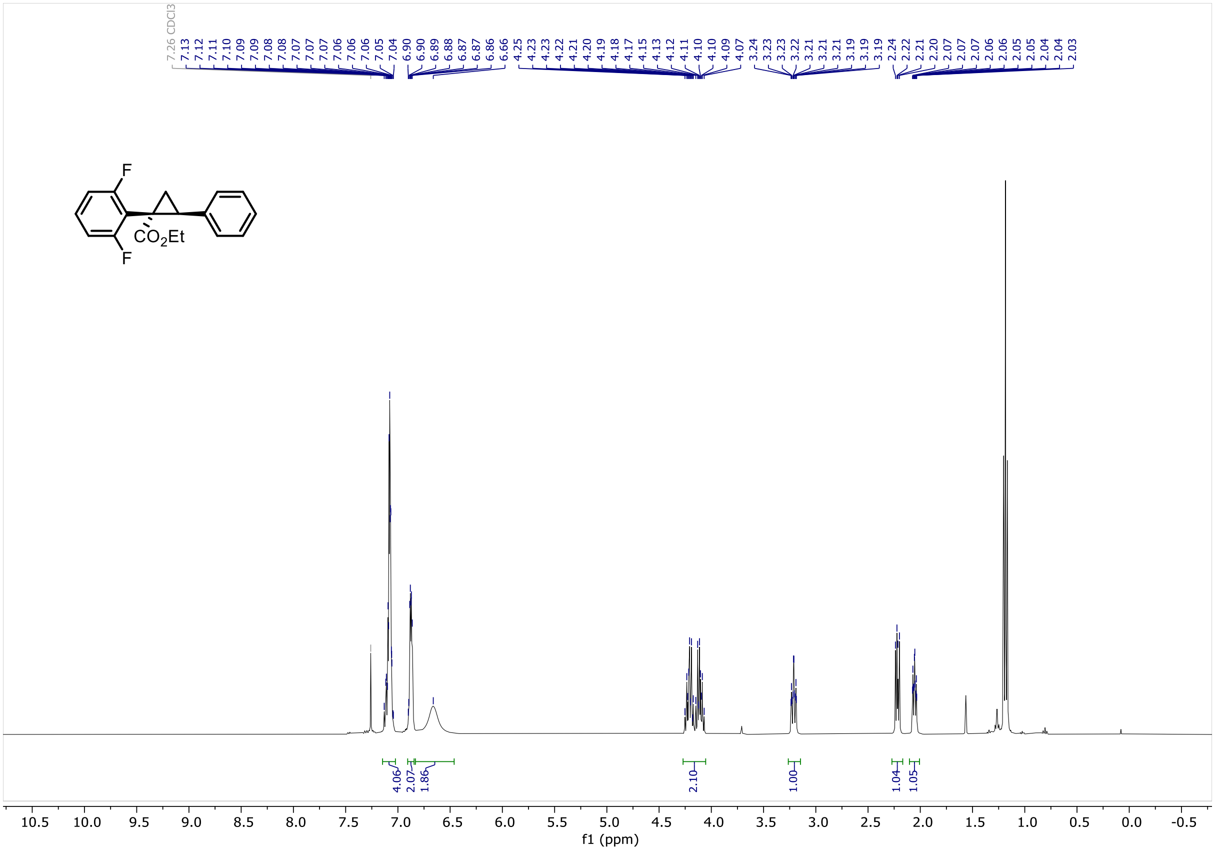

13: ^{13}C NMR (101 MHz, $CDCl_3$)

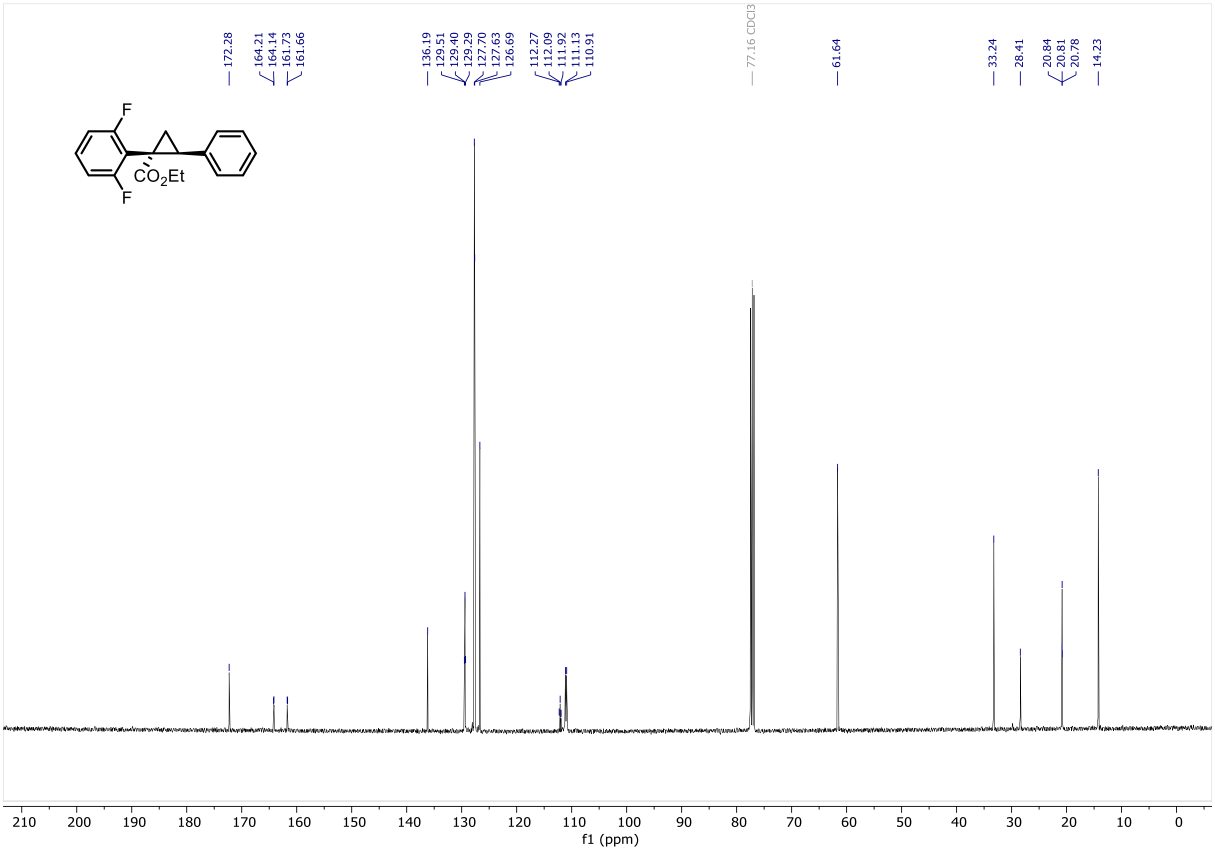

13: ^{19}F NMR (565 MHz, $CDCl_3$)

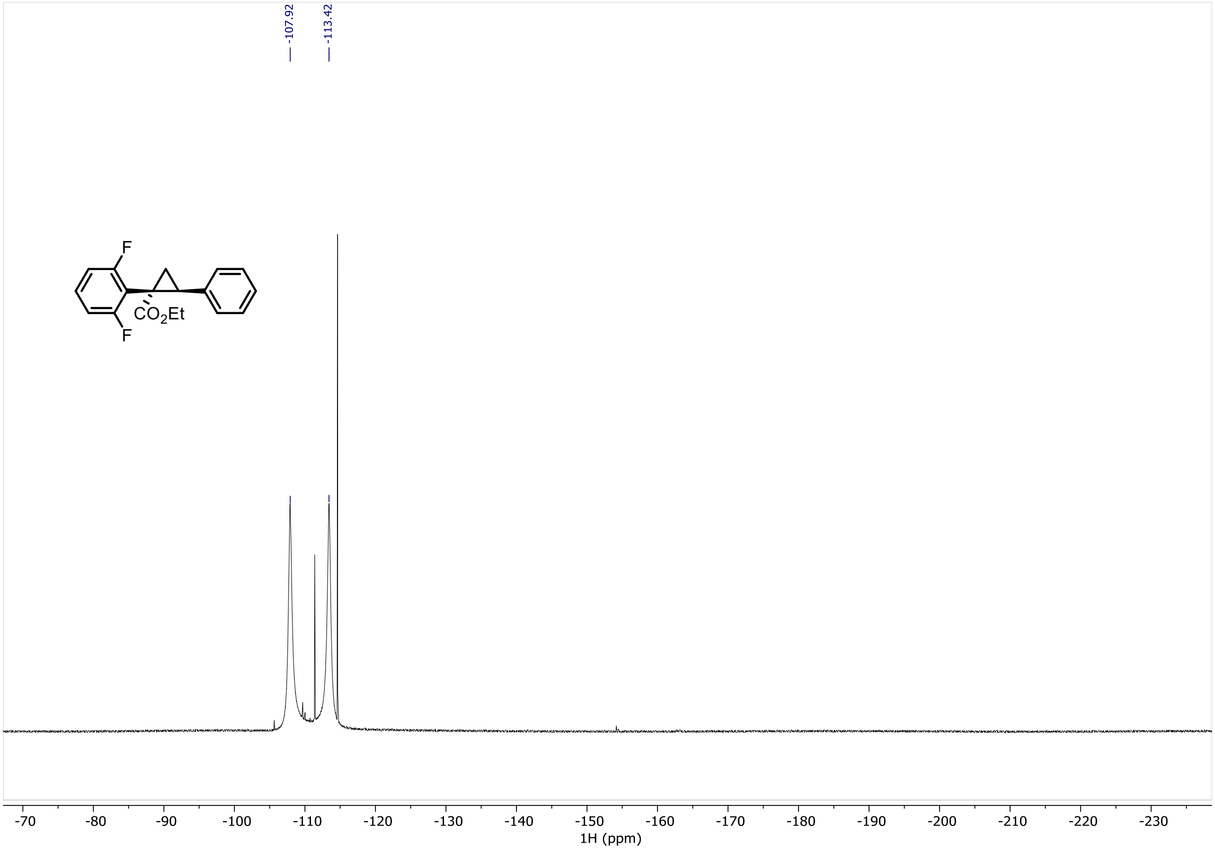

References

[1] G. R. Fulmer, A. J. M. Miller, N. H. Sherden, H. E. Gottlieb, A. Nudelman, B. M. Stoltz, J. E. Bercaw, K. I. Goldberg, *Organometallics* **2010**, *29*, 2176-2179.

[2] M. Peeters, L. Baldinelli, S. Lerda, G. Bistoni, A. Fürstner, *J. Am. Chem. Soc.* **2025**, *147*, 12418-12424.

[3] P. Sreedevi, J. B. Nair, P. Preethanuj, B. S. Jeeja, C. H. Suresh, K. K. Maiti, R. L. Varma, *Analyt. Chem.* **2018**, *90*, 7148-7153.

[4] A. Pedrini, J. Perego, S. Bracco, C. X. Bezuidenhout, P. Sozzani, A. Comotti, *J. Mat. Chem. A* **2021**, *9*, 27353-27360.

[5] M. Larsen, M. Jørgensen, *J. Org. Chem.* **1996**, *61*, 6651-6655.

[6] M. Peeters, J. Decaens, A. Fürstner, *Angew. Chem. Int. Ed.* **2023**, *62*, e202311598.

[7] S. Singha, M. Buchsteiner, G. Bistoni, R. Goddard, A. Fürstner, *J. Am. Chem. Soc.* **2021**, *143*, 5666-5673.

[8] J.-D. Chai, M. Head-Gordon, *Phys. Chem. Chem. Phys.* **2008**, *10*, 6615-6620.

[9] F. Weigend, R. Ahlrichs, *Phys. Chem. Chem. Phys.* **2005**, *7*, 3297.

[10] K. Eichkorn, O. Treutler, H. Öhm, M. Häser, R. Ahlrichs, *Chem. Phys. Lett.* **1995**, *240*, 283-290.

[11] F. Neese, *J. Comput. Chem.* **2003**, *24*, 1740-1747.

[12] F. Weigend, *Phys. Chem. Chem. Phys.* **2006**, *8*, 1057-1065.

[13] V. Barone, M. Cossi, *J. Phys. Chem. A* **1998**, *102*, 1995-2001.

[14] M. Cossi, N. Rega, G. Scalmani, V. Barone, *J. Comput. Chem.* **2003**, *24*, 669-681.

[15] P. Pracht, F. Bohle, S. Grimme, *Phys. Chem. Chem. Phys.* **2020**, *22*, 7169-7192.

[16] S. Spicher, S. Grimme, *Angew. Chem. Int. Ed.* **2020**, *59*, 15665-15673.

[17] A. D. Becke, *J. Chem. Phys.* **1993**, *98*, 1372-1377.

[18] C. Lee, W. Yang, R. G. Parr, *Phys. Rev. B: Condens. Matter Mater. Phys.* **1988**, *37*, 785-789.

[19] S. H. Vosko, L. Wilk, M. Nusair, *Can. J. Phys.* **1980**, *58*, 1200-1211.

[20] P. J. Stephens, F. J. Devlin, C. F. Chabalowski, M. J. Frisch, *J. Phys. Chem.* **1994**, *98*, 11623-11627.

[21] S. Grimme, J. Antony, S. Ehrlich, H. Krieg, *J. Chem. Phys.* **2010**, *132*, 154104.

[22] S. Grimme, S. Ehrlich, L. Goerigk, *J. Comput. Chem.* **2011**, *32*, 1456.

[23] B. de Souza, *Angew. Chem. Int. Ed.* **2025**, *64*, e202500393.

[24] G. Regni, L. Baldinelli, G. Bistoni, *ACS Cent. Sci.* **2025**, *11*, 890-898.

[25] R. L. Sutar, S. M. Huber, *ACS Catal.* **2019**, *9*, 9622-9639.

[26] J. Bamberger, F. Ostler, O. G. Mancheño, *ChemCatChem* **2019**, *11*, 5198-5211.
